# 41st International Symposium on Intensive Care and Emergency Medicine

**DOI:** 10.1186/s13054-022-03927-z

**Published:** 2022-03-25

## P001

### Sex-related differences in patients’ characteristics, provided care, and outcomes following spontaneous intracerebral hemorrhage

#### SW Wang^1^, SYB Bögli^2^, SW Wildbolz^1^, NM Nierobisch^1^, EK Keller^1^, GB Brandi^1^

##### ^1^UniversitätsSpital Zürich, Zürich, Switzerland, ^2^UniversitätsSpital Zürich, Intensive Care Medicine, Zürich, Switzerland

*Critical Care* 2022, **26(Suppl 1):** P001

**Introduction:** Sex-related differences in patients with hemorrhagic stroke due to spontaneous intracerebral hemorrhage (ICH) are poorly investigated. This study elucidates whether sex-related differences exist in particular with regard to provided care, while also taking into account the patients’ characteristics and outcomes in patients with ICH admitted to the neurocritical care unit.

**Methods:** This retrospective single center study includes all consecutive patients with spontaneous ICH admitted to the Neurocritical Care Unit in a 10-year period. Patients’ demographics, comorbidities, symptoms at presentation, radiological findings, surgical and non-surgical provided care, and ICU- and 12-month mortality were compared between men and women.

**Results:** A total of 398 patients were included (male = 198 and female = 200). No differences in demographics, Charlson Comorbidity Index, symptoms at presentation, ICU- and 12-month mortality were observed among men and women. Men received an external ventricular drain (EVD) for hydrocephalus-therapy significantly more often than women did. In the multivariate analysis, EVD insertion was independently associated with male gender (OR 2.82, 95%-CI 1.61–4.95, *p* value < 0.001) irrespective of demographic or radiological features. Functional outcome after ICH as assessed by mRS was more favorable for women (*p* = 0.044).

**Conclusions:** Sex-related differences in patients with ICH with regard to the surgically provided care exist. We provide evidence that insertion of EVD is associated with male gender disregarding clear reasoning. We suggest that a gender-bias as well as social factors play a significant role in decision-making for the insertion of an EVD.

## P002

### Connection between possible and proved predictors of the post-neurosurgical meningitis 4-hydroxyphenyllactic acid and lactate in cerebrospinal fluid

#### A Pautova, A Meglei, E Chernevskaya, N Beloborodova

##### Federal Research and Clinical Center of Intensive Care Medicine and Rehabilitology, Moscow, Russian Federation

*Critical Care* 2022, **26(Suppl 1):** P002

**Introduction:** The cerebrospinal fluid (CSF) concentration of the bacterial 4-hydroxyphenyllactic acid (*p-*HPhLA) was recently shown to be a possible one-parameter criterion for the diagnosis of the post-neurosurgical bacterial meningitis (PNBM) with cut-off value of 0.9 µmol/l [1]. CSF lactate in known to be a PNBM marker with cut-off value of 4 mmol/l [2]. The goal of this study was to determine if the CSF content of these parameters correlate with each other in neurosurgical patients.

**Methods:** The residues of CSF samples from neurosurgical patients (n = 84) were obtained after diagnostic lumbar puncture. Concentration of CSF *p-*HPhLA was measured by gas chromatography–mass spectrometry, CSF lactate level was obtained from medical records of patients. CSF *p-*HPhLA and lactate were studied in dynamics in some patients (n = 5).

**Results:** *p*-HPhLA is known to correlate with the serum lactate [3]. Moderate positive statistically significant Spearman’s rank correlation was revealed between CSF *p*-HPhLA and lactate (r = 0.55, *p* = 0.01 2-tailed). Some patients were studied in dynamics to illustrate the revealed correlation. Patient 5 had no signs of PNBM; lactate and *p*-HPhLA were less than cut-off values (Fig. 1). Patients 1–4 had signs of PNBM according to specific criterium of positive CSF culture (patients 1 and 3) and non-specific criteria of neutrophilic pleocytosis, high CSF protein and lactate (patients 2 and 4). *p*-HPhLA was higher than cut-off value in all patients with signs of PNBM, while lactate levels were lower than cut-off values in patients 3–4. Detailed analysis of the patients’ medical records explained the revealed differences.

**Conclusions:** The correlation between CSF lactate and *p-*HPhLA indicates the diagnostic significance and pathophysiological role of *p-*HPhLA in the development of PNBM**.**

**References**

1. Beloborodova N et al. Crit Care 25(Suppl 1):P108, 2021.
2. Maskin L et al. Clin Neurol Neurosurg 115:1820–5, 2013.
3. Beloborodova N et al. Shock 50:273–9, 2018.

**Fig. 1 (abstract P002):**
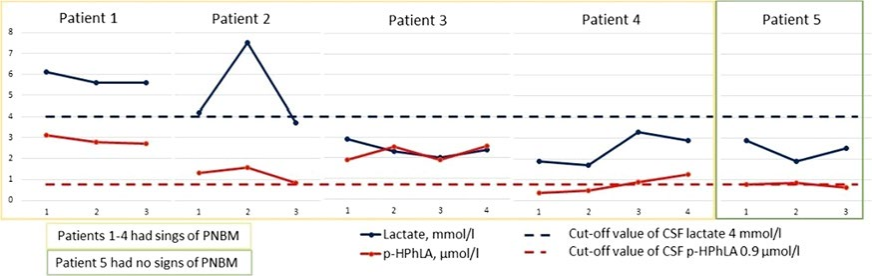
Dynamics of CSF lactate and p-HPhLA in post-neurosurgical patients with (patients 1-4) and without (patient 5) signs of PNBM.

## P003

### Assessing outcome in ischemic stroke patients with automated pupillometry

#### E Marinangeli, A Marudi, G Bettelli, G Melegari, C Dallai, S Rinaldi, L Pietropaoli, G Branchetti, E Bertellini

##### Azienda Ospedaliero Universitaria Policlinico di Modena, Anestesia e Rianimazione, Modena, Italy

*Critical Care* 2022, **26(Suppl 1):** P003

**Introduction:** The assessment of pupillary light reflex (PLR) is essential in critical care. Automated pupillary is now available and provides a precise measure of the Constriction Velocity (CV), latency, dilation velocity, size, percent change, and Neurological Pupil index (NPi) [1,2]. The purpose of this study is to find a correlation between NPi, CV and unfavorable outcome in patients with ischemic stroke.

**Methods:** We included patients with ischemic stroke admitted to NeuroIntensive Care Unit in Modena Hospital from August 2019 to August 2020. We collected NPi and CV in the first 72 h of hospitalization, selecting the worst measurement as significant. We used the Neuroptics NPi-200 pupillometer: NPi values < 3 and CV < 0.8 mm/sec were considered abnormal. Finally we collected the GCS at the discharge of our patients, evaluating as GCS ≤ 8 as an unfavorable outcome. A Fisher’s exact test was added to the analysis to lend reliability to the significance of the stratified binary variables. We performed the statistical analysis with SPSS statistic version 25.

**Results:** We included 18 patients, 12 male, the median of age is 71.5; in the first 72 h of admission 2 patients had left NPi < 3 and 9 patients left CV < 0.8 mm/sec, 3 patients right NPi < 3 and 6 patients right CV < 0.8 mm/sec. At the discharge from ICU 7 patients had GCS ≤ 8.2 patients had left NPi < 3 and GCS at the discharge ≤ 8; 2 patients had right NPi < 3 and GCS at the discharge ≤ 8; 5 patients had left CV < 0.8 mm/sec and GCS at the discharge ≤ 8; 3 patients had right CV < 0.8 mm/sec and GCS at the discharge ≤ 8. There was no statistically difference between each group.

**Conclusions:** CV and NPi evaluate two different aspects of the PLR; NPi and CV are both unlinked to ischemic stroke outcome at discharge, maybe due to the sample size. Further studies are required to the determine the utility of CV and NPi in the prognostication of ischemic stroke outcome.

**References**

1. Shoyombo et al. Sci Rep 8:6992, 2018
2. Jahns et al. Crit Care 23:155, 2019

## P004

### Automated quantitative pupillometry to predict neurologic outcome in SAH patients: a pilot study

#### A Blandino Ortiz^1^, J Higuera Lucas^1^, G Alonso Salinas^2^, C Soriano^1^, S Saez^1^, R De Pablo^1^

##### ^1^Ramón y Cajal University Hospital, Department of Intensive Care, Madrid, Spain, ^2^Complejo Hospitalario de Navarra, Department of Cardiology, Pamplona, Spain

*Critical Care* 2022, **26(Suppl 1):** P004

**Introduction:** The pupillary examination is a fundamental part of the neurological assessment in neurocritical patients, in whom pupillary abnormalities are associated with poor outcome. Traditionally pupillary examination is based on subjective, and inaccurate estimation, nevertheless, in the past years, the use of automated quantitative pupillometry (AQP) has change our way to assess the pupillary function as it allows us to detect subtle changes.

**Methods:** We included all consecutive patients with aneurysmatic subarachnoid hemorrhage (a-SAH) admitted in our ICU from September 2019-March 2020 (n = 21). We recorded demographics data, clinical characteristics, pupillometry measurements during ICU stay, therapies, complications, neurological outcomes at ICU discharge and 3 months.

**Results:** Of 21 patients (age 60 [31–82] years), 16 were aneurysm of anterior circulation (Fig. 1), 9 (42.8%) developed intracerebral hematoma. 81% had endovascular therapy, among these, 28% intra-arterial vasodilators, and 14.2% angioplasty. 57% needed external ventricular drainage, 33% and 28% require osmotic agents and barbiturates respectively. The ICU mortality was 24% (n = 5), the mean GOS at ICU discharge was 3, 62% had unfavorable neurological outcome (UO) at ICU discharge, favorable neurological outcome (FO) improved in 52.3% (n = 11) at 3 months. Regarding AQP, abnormal NPi values was associated with UO at ICU discharge (RR 2.4 [95% IC 3.4 to 1.4], *p* = 0.0001) and at 3 months (RR 2.1 [95% IC 0.9 to 3.2], *p* = 0.001). On the contrary NPi > 3 was associated with FO at ICU discharge (mean diff GOS -2.07; [95% IC − 1.4 to − 2.7], *p* = 0.0001), as well at 3 months (mean diff GOS 2.4; [95% IC − 2.2 to − 3.7], *p* = 0.001).

**Conclusions:** AQP seems to be a useful tool to predict neurological outcome (GOS) at ICU discharge and at 3 months in a-SAH patients. We haven’t found a significant association with abnormal pupillometry values and ICU mortality; however, this could be explained for the small sample of patients.

**Fig. 1 (abstract P004):**
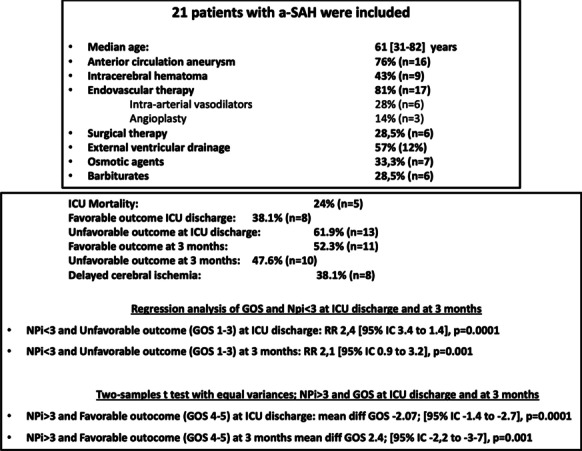
Results.

## P005

### Determination of the average diameter of the optic nerve in Bogota, Colombia

#### EE Rodríguez, JA Carrizosa, C Perez

##### Fundación Santa Fe de Bogotá, Intensive Care Department, Bogotá, Colombia

*Critical Care* 2022, **26(Suppl 1):** P005

**Introduction:** Noninvasive measurement of intracranial pressure (ICP) has become an important issue in neurocritical care. Ultrasound measures with transcranial Doppler and optic nerve sheath diameter (ONSD) estimation can be made at the bedside [1]. We present preliminary results of a study in healthy volunteers (work team members and students) to find the ONSD average in non-hospitalized patients, which we defined as normal population, with the hypothesis that it will be different from the value reported in other populations [2]. This will be the first study in a healthy Colombian population and the first that measures the ONSD using a standardized technique with the enforcement of the CLOSED protocol and the adjustment with the transverse eyeball diameter (ED) [3,4].

**Methods:** A total of 246 subjects are needed in this Metexploratory observational study with a cross-sectional design to estimate the ONSD average value measured according to the CLOSED protocol, the ratio of ONSD/ED, and determine if it is affected by variables such as sex, age, and comorbidities.

**Results:** Preliminary results of 86 subjects showed an ONSD average around 0.35 cm ± 0.12 cm with extreme data up to 0.55 cm. Ratio of ONSD/ED is around 0.17 cm ± 0.03 cm, including values of 0.31 as reported in other populations [2]. There is no effect of sex or comorbidities in our study. The few cases where we could not apply the CLOSED protocol were excluded from the analysis.

**Conclusions:** The first preliminary analysis showed that the ONSD average seems lower than we thought. Even if there are many patients with an expected value around 0.5 cm, our race-plural population may benefit from establishing a lower cut point to suspect a raised ICP. Nevertheless, this is a preliminary report, and ultimately measures must be done before concluding a cut-off.

**References**

1. Robba C et al. Echography and Doppler of the Brain. 1ed. 2021
2. Kim DH et al. Sci Rep 7:15,906, 2017
3. Aspide R et al. Neurocrit Care 32:327–332, 2020
4. Du J et al. Neurocrit Care 32:478–485, 2020

## P006

### Infectious extracranial complications in TBI hospitalized in ICU: a retrospective analysis in a trauma center

#### A Sica, E Russo, C Dell’Amore, A Cittadini, DP Santonastaso, D Bellantonio, G Scognamiglio, C Turrini, E Gamberini, V Agnoletti

##### Ospedale Maurizio Bufalini, Anestesia e Rianimazione, Cesena, Italy

*Critical Care* 2022, **26(Suppl 1):** P006

**Introduction:** Aim of the study is to evaluate the frequency of extracranial infectious complications in TBI and the association with various degrees of severity of TBI. TBI is frequently associated with extracranial complications and adversely affects outcome [1]. iVACs causing increased ICU days and duration of MV; uncertain is the impact on survival. The clinical benefit of early aggressive antibiotic treatment has not been demonstrated [2]. There is no indication for antibiotic prophylaxis but vigilant monitoring of the development of iVACs are recommended.

**Methods:** A retrospective analysis was conducted on clinical data from patients admitted with diagnosis of polytrauma with TBI, from 1/1/19 to 31/12/19, to the Department Major Trauma AUSL Romagna at the Hospital "M. Bufalini" of Cesena. We collected AIS and ISS (criteria of CENTER-TBI[3]), GCS, presence of MV, percutaneous tracheotomy, development of AKI/CRRT, application of empiric/targeted antimicrobial therapy. We defined the incidence of iVACs and assessed the significant association with clinical score and trauma severity. For statistical analysis Independent Student's t test, Mann Whitney's U test and χ2 test were used.

**Results:** During the observation period 264 patients were admitted. 16.6% of patients developed an infectious process; iVAC in 13.8% (the only ones that have allowed statistical inferential analysis [4]), peritonitis in 1.13% and skin and soft tissue infections in 1.51%. GCS value and AIS head/ISS were lower and higher, respectively, in iVAC group compared to the no-iVAC group; they reached statistically significant differences (Table 1). Not the same was observed for AIS thorax between the 2 groups.

**Conclusions:** The severity of head injury, quantified using the value of GCS, head AIS, and ISS, has an association with the development of iVAC.

**References**

1. Robba C et al. Curr Opin Crit Care 26:137–146, 2020
2. Chieregato A et al. Minerva Anest 83:553–562, 2017
3. Steyerberg EW et al. Lancet Neu 18:923–934, 2019
4. Esnault P et al. Neurocrit Care 27:187–198, 2017

**Table 1 (abstract P006):** Results

		NO iVACs	iVACs	*p* Value
GCS tot.	Average (s.d.)	11.58 (4.15)	8.97 (4.93)	–
	Median (IQR)	14 (7)	7.5 (11.5)	0.003
AIS head	Average (s.d.)	2.5 (2.1)	3.4 (1.9)	0.001
	Median (IQR)	3 (4)	4 (2)	
ISS	Average (s.d.)	32.10 (16.2)	37.47 (10.59)	0.01
	Median (IQR)	29 (16)	36 (11.5)	
AIS thorax	Average (s.d.)/Median (IQR)	2.1 (1.8)/3 (4)	2.4 (1.9)/ 3 (4)	0.436

## P007

### Safety of 5% sodium chloride bolus administration via peripheral venous access in neurocritical care patients

#### AL Brask, TS Lam, JT Jancik

##### Hennepin County Medical Center, Clinical Pharmacy, Minneapolis, USA

*Critical Care* 2022, **26(Suppl 1):** P007

**Introduction:** Hypertonic saline (HTS) is effective at lowering intracranial pressure in patients with acute brain injury [1]. When administered via peripheral venous access, HTS may result in extravasation, pain, and tissue injury [2]. Institutions may restrict 5% HTS administration to central vascular access. The aim of this study was to evaluate the safety of administration of 5% sodium chloride (NaCl) bolus via peripheral venous access.

**Methods:** This single center, retrospective study evaluated adult neurocritical care patients who received 5% NaCl over 20 min via peripheral venous access between January 2015 and February 2019. The primary outcome was occurrence of infusion-related complications such as extravasation, phlebitis, or soft tissue injury. The secondary outcome was the incidence of hyperchloremia (serum chloride > 110 mEq/l) or hypernatremia (serum sodium > 160 mEq/l).

**Results:** Of 514 peripheral administrations of 5% NaCl, 7 (1.4%) were associated with infusion-related complications. Among these, 6 cases resulted in extravasation requiring intervention, and 1 case resulted in a skin tear requiring intervention. None of the complications required surgical intervention. Five of the cases were documented as such that alternative causes of the patients’ symptoms could not be ruled out. Electrolyte abnormalities occurred with 181 (35.2%) administrations, all of which involved hyperchloremia. Among these, hypernatremia also occurred with 5 (2.8%) administrations.

**Conclusions:** Peripheral venous administration of 5% NaCl bolus appears safe, as demonstrated by a low incidence of infusion-related complications in the study population.

**References**

1. Cooper DJ et al. JAMA 291:1350–1357, 2004.
2. Jones GM et al. Am J Crit Care 26:37–42, 2016.

## P008

### Protocol-driven detection of potential organ donors in a German emergency department

#### B Schmid^1^, CN Lang^2^, HJ Busch^1^, G Neitzke^3^, KM Lücking^4^

##### ^1^Medical Center – University of Freiburg, Department of Emergency Medicine, Freiburg im Br., Germany, ^2^Medical Center – University of Freiburg, Department of Medicine III (Interdisciplinary Medical Intensive Care), Freiburg im Br., Germany, ^3^Medical School Hannover, Institute for History, Ethics and Philosophy of Medicine, Hannover, Germany, ^4^Medical Center – University of Freiburg, Coordinator for transplantation, Freiburg im Br., Germany

*Critical Care* 2022, **26(Suppl 1):** P008

**Introduction:** The detection of potential organ donors is a fundamental step to meet patients’ wishes in end-of-life-care as well as the demand for transplantable organs. Organ donation (OD) after neurologic determination of death is the only accepted legal option in Germany. A guideline recently issued by the German Medical Association encourages ICU staff to check for clinical imminent brain death in patients and evaluate patients’ wishes in this regard. International literature suggests a protocol-driven approach immediately in the emergency department (ED) to increase the detection of potential organ donors.

**Methods:** A written protocol was implemented in the ED of a tertiary university hospital. The protocol was developed by an interdisciplinary team consisting of emergency medicine, neurology, medical ethics, and the local organ donation coordination. The protocol included a pathway for the identification of potential donors, consultations of relatives, pathways for initiation of treatment.

**Results:** About 50,000 patients (primarily adults) seek help in the ED per year. About 2000 of those are critically ill and have to be treated in the resuscitation area. The protocol was implemented in April 2021. During the following 6 months (04–10/2021) 2 additional patients were recognized by using this protocol. Intensive care support was established in these patients in the ED following patients’ wishes in favor of organ donation. The resulting 2 successful OD contributed to the hospital’s overall 12 OD during the observed time period (17%).

**Conclusions:** ED staff plays a central role in the detection of patients who are at imminent risk to develop brain death. Evaluating patients’ wishes early in the ED and subsequent introduction of intensive care means can relevantly contribute to the number of successful OD and organ transplantations.

## P009

### Super-refractory status epilepticus in a neurocritical care unit

#### D Gomes, D Correia, I Moniz, JM Ribeiro

##### Centro Hospitalar Universitário Lisboa Norte, Serviço de Medicina Intensiva, Lisboa, Portugal

*Critical Care* 2022, **26(Suppl 1):** P009

**Introduction:** Super-refractory status epilepticus (SRSE) is defined as status epilepticus (SE) that continues or recurs 24 h or more after the onset of anaesthetic therapy. Treatment is based on case reports and expert opinion, and reported outcome is generally ominous. We aimed to analyse the incidence, clinical characteristics and mortality of patients with the diagnosis of SRSE admitted to a neurocritical care unit (NICU).

**Methods:** Retrospective cohort study. Population consisted of all patients admitted to our NICU with a diagnosis of SRSE, from January 2018 to July 2021. Protocoled-based data were retrieved from our electronic data base. Statistical analysis was performed using T-test and Chi-square for comparing variables between deceased and survival patients. Variables who were statistically significant were introduced in a binary logistic regression model to predict mortality.

**Results:** Of all eighty-two patients with a diagnosis of SE, 29 (35%) fulfilled criteria for SRSE. Mean age was 61-year-old, predominantly females (84.6%); mean SAPS II was 48 and mean admission SOFA was 7. The majority had acute symptomatic aetiology (65.5%). NICU mortality was 44.8%, further 20% patients died in hospital after ICU discharge and another 3.4% after hospital discharge, with a cumulative mortality of 69%. 82.8% were treated with at least 2 anaesthetics and 89.7% with at least 4 conventional antiepileptics. Patients who died where older (66 vs 43 years, *p* < 0.05), mostly male (R = 4.15, *p* < 0.05), were submitted to neurosurgery (R = 8.62, *p* < 0.05) and had nonconvulsive status epilepticus (R = 5.73, *p* < 0.05). Of those variables, only age and need for neurosurgery predict mortality.

**Conclusions:** Despite comprehensive protocol-based treatment in a dedicated neurocritical centre [1], mortality of patients with diagnosis of SRSE remain very high. Better understanding of underlying causes, pathophysiology and higher evidence-based treatment protocols are urgently needed.

**Reference**

1. Gomes D et al. Acta Médica Portuguesa 31:598–605, 2018

## P010

### Paroxysmal sympathetic hyperactivity in patients with chronic disorders of consciousness.

#### E Kondratyeva^1^, S Kondratev^2^, G Rybakov^3^

##### ^1^Almazov Medical Research Centre, Minimally Conscious Research Group, St Petersburg, Russian Federation, ^2^Almazov Medical Research Centre, Intensive Care Department, St Petersburg, Russian Federation, ^3^Almazov Medical Research Centre, ICU department, St Petersburg, Russian Federation

*Critical Care* 2022, **26(Suppl 1):** P010

**Introduction:** Paroxysmal sympathetic hyperactivity (PSH) is often observed in patients with disorders of consciousness (DOC) aggravating the course of the disease and slowing down the recovery processes. The objective of the study was to investigate PSH severity and therapy approaches in DOC patients.

**Methods:** The study was performed in 54 DOC patients, group 1—1 (CRS-R score from 0 to 5)—16 patients, group 2 (CRS-R score from 6 to 8)—19, group 3 CRS-R score from 9 to 23—19 patients were monitored daily with a bedside monitor of the following parameters: blood pressure, heart rate, respiratory rate, oxygen saturation (Sat O2), axillary and rectal temperature. Degree of vegetative dysregulation was assessed by the modified PSH scale [1].

**Results:** PSH was treated according to the following scheme: mild degree of PSH (1–5 points PSH scale), a combination of a beta-blocker (bisoprolol 5–10 mg per day) with diphenine (100–200 mg per day), moderate PSH degree (6–7 points) —clonidine (0.2–1 mcg / kg / h), benzodiazepines or clonazepam (2–6 mg/day), severe PSH (8 or more points), combination of an opioid analgesic (fentanyl 0.2–1.4 mcg/kg/h) with clonidine (0.3–1 mcg/kg/h). Decrease in the overall score on the PSH scale, Kerdo index approaching zero were used to evaluate the efficacy. PSH in response to TBI is one of the manifestations of a stress reaction, which, sometimes becomes fixed, loses its adaptive qualities and passes into a "stable pathological state" and was found in all groups, more severe in group 1 (Fig. 1).

**Conclusions:** An important point in the treatment of DOC patients is the timely correction PSH with a differentiated approach depending on its severity.

**Funding:** The reported study was funded by RFBR project number 19–29-01,066.

**Reference**

1. Baguley IJ et al. J Neurotrauma 31:1515–20, 2014

**Fig. 1 (abstract P010):**
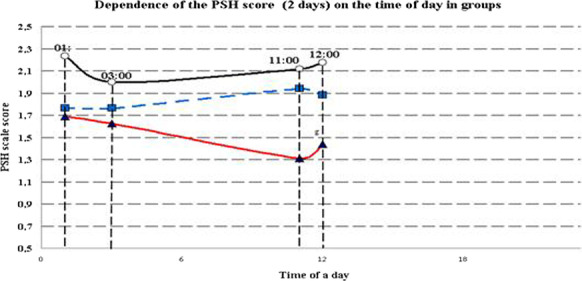
Dependence of the PSH on the time of a day in 3 groups.

## P011

### Behavior of systemic inflammatory markers in patients undergoing craniotomy for tumor resection

#### GA Madrid^1^, MC Niño^2^, D Cohen^2^, J Mercado^2^, J Cortés^1^, D Arias^3^, A Ordoñez^3^, EE Rodríguez^3^

##### ^1^Fundación Santa Fe de Bogotá, Anesthesiology Department, Bogotá, Colombia, ^2^Fundación Santa Fe de Bogotá, Neuroanesthesia Service, Bogotá, Colombia, ^3^Fundación Santa Fe de Bogotá, Intensive Care Department, Bogotá, Colombia

*Critical Care* 2022, **26(Suppl 1):** P011

**Introduction:** Neoplasms generate an inflammatory response associated with poor survival. Neutrophil/lymphocyte ratio (NLR) and platelet/lymphocyte ratio (PLR) are widely available, inexpensive, and their elevation has been associated with decreased survival in solid tumors [1]. We aimed to describe the behavior of these inflammatory markers in patients scheduled for intracranial tumor resection.

**Methods:** A retrospective cohort study was conducted. Patients at least 18 years old undergoing craniotomy for tumor resection between January 2015 and March 2019 were included. We excluded patients if programmed for reintervention, presented sepsis, or had previous corticosteroid use. Demographic and tumor characteristics were recorded, in addition to preoperatory NLR and PLR. Inflammation was defined as NLR > 3 and PLR > 200. Descriptive statistics and bivariate analysis were used as appropriate.

**Results:** A sample of 322 patients was analyzed, of which 53% had benign tumors and 47% had malignant pathology. Preoperative inflammation defined by NLR was present in 44% of patients, whereas 22% had inflammation according to PLR. Demographic variables did not differ between patients with and without inflammation. Inflammation by NLR was more common in patients with supra and infratentorial tumors, than in patients with pituitary tumors (*p* = 0.003). NLR > 3 was also associated with preoperative cerebral edema (*p* = 0.01), hypertension (*p* = 0.05), heart failure (*p* = 0.047), and dementia (*p* = 0.037). PLR was not associated with the evaluated variables.

**Conclusions:** Preoperative inflammation defined as NLR > 3 was associated with supratentorial and infratentorial tumors, as well as comorbidities such as cerebral edema, hypertension, heart failure and dementia. PLR was not associated with preoperative variables.

**Reference**

1. Templeton AJ et al. J Natl Cancer Inst 106:dju124, 2014.

## P012

### Blood metabolomic profiling of chronic disorders of consciousness.

#### E Kondratyeva^1^, A Orlova^2^, Y Dubrovskii^3^, N Dryagina^4^, E Verbitskaya^5^, S Kondratyev^6^

##### ^1^Almazov Medical Research Centre, Minimally Conscious Research Group, St Petersburg, Russian Federation, ^2^Saint Petersburg State Chemical Pharmaceutical University (SPCPA, Researcher of the Department of Science and Training of Scientific and Pedagogical Personnel, Assistant of the Department of Pharmacognosy, Saint Petersburg, Russian Federation, ^3^Almazov Medical Research Centre, Metabolomic research centre, St Petersburg, Russian Federation, ^4^Almazov Medical Research Centre, Department of laboratory Diagnostic, St Petersburg, Russian Federation, ^5^Pavlov University, Associate Professor of the Department of Clinical Pharmacology, Head of the Department of Biomedical Statistics, Saint Petersburg, Russian Federation, ^6^Almazov Medical Research Centre, Intensive Care Department, St Petersburg, Russian Federation

*Critical Care* 2022, **26(Suppl 1):** P012

**Introduction:** Aim of the study was to investigate the main differences in metabolic disorders in DOC patients (VS/UWS, MCS) and to identify changes in the metabolome depending on the phase of sleep or wakefulness.

**Methods:** Metabolomic plasma profile of 10 VS/UWS (group 1), 6 MCS (group 2). Etiology: group 1 (TBI—2, hypoxia—8), group 2 (TBI—5, hypoxia -1). Jugular vein was catheterized in all patients, blood sampling was carried out in a waking state during the daytime for 2 days. Aliquots of pooled plasma samples were purified from protein components and analyzed by high-performance liquid chromatography in two modes—reversed-phase and hydrophilic. Mass spectrometric detection was carried out in full ion current scanning mode: registration of positively charged ions in the m/z range from 50 to 1300 a.e.m. Data alignment and normalization were performed using MS-DIAL ver software. 4.70 Statistical analysis -MetaboAnalyst 5.0 software.

**Results:** A non-target metabolomic analysis of patient groups in VS/UWS and MCS, on the reversed-phase column four metabolites (with VIP > 0.5), the level of which is most modulated depending on the group under consideration, were identified: 4 (m/z 124.0867, Rt = 17.67, *p* < 0.01), 33 (m/z 782.5722, Rt = 17.69, *p* < 0.01), 6 (m/z 125.0904, Rt = 18.43, *p* < 0.01) and 1 (m/z 463.2304, Rt = 15.78, *p* < 0.01), and there were no any significant differences between daytime and nighttime blood sampling. Analysis on a hydrophilic column showed significant quantitative differences of 3 metabolites in groups.

**Conclusions:** In the course of the study, a set of metabolites was established—the use of biomarkers for the differential diagnosis of VS/UWS and MCS—they are 4, 33, 6, 1 for an experiment on a reversed-phase column and 14, 35, 41, 48 for an experiment on a hydrophilic column, based on their significant contribution to the manifestation of intergroup and intragroup differences.

**Acknowledgement:** The study was carried out with the financial support of the RFBR project No. 19–29-01,066.

## P013

### The use of 100% oxygen during non-effective cardiopulmonary resuscitation improves brain oxygenation compared to 50% oxygen

#### A Nelskylä^1^, J Humaloja^1^, E Litonius^2^, P Pekkarinen^2^, G Babini^1^, T Mäki-Aho^1^, J Heinonen^2^, MB Skrifvars^1^

##### ^1^University of Helsinki and Helsinki University Hospital, Department of Emergency Medicine and Services, Helsinki, Finland, ^2^University of Helsinki and Helsinki University Hospital, Department of Anesthesia, Intensive Care, and Pain Medicine, Helsinki, Finland

*Critical Care* 2022, **26(Suppl 1):** P013

**Introduction:** Guidelines recommend 100% oxygen during cardiopulmonary resuscitation (CPR) but studies show that during effective mechanical chest compressions 50% oxygen may result in comparable brain oxygen levels [1]. Lower FiO_2_ might reduce reperfusion injury but could cause hypoxia during poor quality CPR. We compared 100% and 50% oxygen during poor quality manual chest compressions.

**Methods:** After Finnish National Animal Experiment Board (ESAVI/15067/2018, ESAVI/35183/2019) approval, ventricular fibrillation (VF) was induced electrically in anaesthetized pigs and left untreated for 5 min, followed by randomization to poor quality chest compressions with manual ventilation with 50% or 100% oxygen. Defibrillation was performed at 10 min and CPR continued with mechanical chest compressions (LUCASTM) and defibrillation every 2 min until 36 min or ROSC. Cerebral oxygenation was measured with near-infrared spectroscopy (rSO_2_) and invasive brain tissue oxygen (PbO_2_). Cerebral oxygenation was compared between groups with Mann–Whitney *U* tests.

**Results:** Twenty-eight pigs were included in the study with 14 cases in each group. With a median time of 15 min, 9 pigs achieved ROSC in the 50% group and 8 pigs in the 100% group (*p* = 0.699). During non-effective CPR PbO_2_ (*p* = 0.001) was higher with FiO_2_ 100%, but rSO_2_ showed no difference (*p* = 0.070). During mechanical chest compressions, there was no difference in rSO_2_ (0.085) and PbO_2_ (0.970) between groups. After ROSC the rSO_2_ and PbO_2_ increased significantly in both groups.

**Conclusions:** The use of 100% oxygen during non-effective CPR improves brain oxygenation.

**Reference**

1. Nelskylä A et al. Resuscitation 116:1–7, 2017

## P014

### Centralising cardiac arrest care: a single centre retrospective observational study

#### R Pugh^1^, M Papadopoullos^2^, J Scanlon^1^, R Craddock^1^

##### ^1^Glan Clwyd Hospital, Department of Anaesthetics, Bodelwyddan, UK, ^2^Cardiff University, School of Medicine, Cardiff, UK

*Critical Care* 2022, **26(Suppl 1):** P014

**Introduction:** Glan Clwyd Hospital (GCH) has offered a 24/7 Percutaneous Coronary Intervention (PCI) service in North Wales (population approx. 690,000) since 2017 and has been designated one of three Welsh Cardiac Arrest Centres. The aim of the study was to evaluate the impact of this development upon resource requirements and outcomes.

**Methods:** Retrospective review of the ICU Ward Watcher database to identify patients undergoing CPR in the 24 h prior to admission April 2013–April 2021. Patients likely to have sustained Out-of-Hospital Cardiac Arrest (OOHCA) of primary cardiac aetiology (OOHCA-C) were identified from primary/secondary diagnoses and free text entry. Data were subsequently analysed using Excel and SPSS. The project was registered as a service evaluation.

**Results:** There were 367 ICU admissions following cardiac arrest; 245 were OOHCA, of which 189 were considered OOHCA-C. Annual OOHCA admissions increased through the study period from 12 (2013–2014) to 50 (2019–2020) before decreasing to 29 during COVID-19 pandemic (2020–2021). OOHCA bed days increased from 38 in 2013–2014 to 215 in 2019–2020, falling to 169 in 2020–2021. Proportions of OOHCA-C patients undergoing pre-ICU PCI increased with time (33% in 2013–2014 to 47% in 2020–2021). Hospital mortality following OOHCA was 61.2% and OOHCA-C was 59.7%; temporal trends did not reach statistical significance. Main factors from first 24 h of ICU admission associated with hospital mortality are presented below. On logistic regression, only lactate, central temperature and lack of pre-ICU PCI significantly predicted hospital mortality (*p* < 0.001) (Table 1).

**Conclusions:** Centralising cardiac arrest care has led to an appreciable rise in ICU bed occupancy. Although overall hospital mortality for OOHCA-C remains high and appreciating potential selection bias, a significant association between PCI and survival to hospital discharge appears to support clinical pathways enabling PCI access following OOHA-C [1].

**Reference**

1. Nolan et al. Intensive Care Med 47:369–421, 2021.

**Table 1 (abstract P014):** Hospital mortality following OOHCA-C

Factor (categorical or median)	Lived (n = 76)	Died (n = 113)	All (n = 189)	*p* Value
Age (years)	61	67	64	0.007
APACHE II	14	19	17	< 0.001
Pre-ICU PCI	47 (62%)	35 (31%)	82 (43%)	< 0.001
Lowest P:F ratio (kPa)	26.9	22.0	23.5	0.003
Lowest systolic blood pressure (mmHg)	89	84	85	0.029
Highest lactate (mmol/l)	2.5	3.8	3.2	< 0.001
Highest temperature (C)	37.0	36.5	36.9	< 0.001

## P015

### The simple predictive indicators of outcome related to oxidative stress after out-of-hospital cardiac arrest

#### O Shigemitsu, R Takenaka

##### Oita University, Emergency Medicine, Faculty of Medicine, Oita, Japan

*Critical Care* 2022, **26(Suppl 1):** P015

**Introduction:** Out-of-hospital cardiac arrest (OHCA) occurs annually in 250,000–300,000 patients worldwide. The management of cardiac arrest is progressing including cardiopulmonary resuscitation (CPR) and the therapy of post cardiac arrest syndrome (PCAS). However, the social recovery rate remains low even now. Knowing accurate prognosis of PCAS and good neurological outcome is very important for utilizing limited medical resources. In recent, there are various cardiac arrest prognostic scores using multiple factors. The minimum indicators to know these are desirable to be able to obtain just after coming to hospital.

**Methods:** Blood samples of OHCA patients were corrected immediately after visited to our hospital between September 2016 to May 2019. Biological anti-oxidant potential (BAP) and derivatives-of-reactive oxygen metabolites (d-ROM) as oxidative stress indicators were measured with Free carrio Duo™ (Wismerll, Italy), 8-hydroxy-2’-deoxyguanosine (8-OHdG), carbonyl protein (CP), and High-mobility group box 1 (HMGB1) were measured with ELISA kit. These indicators values from 18 health volunteers (HV) were used as controls.

**Results:** Overall, 33 OHCA patients were included. 25 patients achieved ROSC (ROSC group), 8 patients did not achieve ROSC (ROSC- group). ROSC- group had significantly higher BAP than HV and ROSC group (*p* < 0.05). Conversely ROSC- group had lower CP than HV and ROSC group. The receiver operating characteristics (ROC) curve of -BAP for predicting survival 28 days after OHCA was 0.724. ROC curve of CP of that was 0.720. BAP of CPC 3–5 group was higher than that of CPC1-2.

**Conclusions:** The oxidative stress (BAP and CP) were strong correlate with 28-day survival and good neurological condition (BAP only) despite single indicator.

## P016

### Prognostic capabilities of inflammatory markers after out-of-hospital cardiac arrest: a systematic review

#### AMJ Seppä^1^, MB Skrifvars^2^, PT Pekkarinen^1^

##### ^1^University of Helsinki and Helsinki University Hospital, Division of Intensive Care, Department of Anaesthesiology, Intensive Care and Pain Medicine, Helsinki, Finland, ^2^University of Helsinki and Helsinki University Hospital, Department of Emergency Care and Services, Helsinki, Finland

*Critical Care* 2022, **26(Suppl 1):** P016

**Introduction:** Out-of-hospital cardiac arrest (OHCA) survivors often develop a post-cardiac arrest syndrome in which systemic inflammation plays an important role [1]. We conducted a systematic review to summarize current evidence regarding whether inflammatory marker levels are useful for prognostic assessment and guidance of treatment in the intensive care unit setting.

**Methods:** We conducted a systematic search from PubMed database using search terms (“inflammation” OR “cytokines”) AND “out-of-hospital cardiac arrest”. Afterwards, each inflammatory marker found was combined with “out-of-hospital cardiac arrest” with AND function to find further relevant studies. We included original articles where inflammatory markers were measured from adult OHCA patients, and their prognostic capabilities were assessed regarding mortality, neurological outcome, or severity of organ failure.

**Results:** Forty-seven studies met the inclusion criteria. Elevated procalcitonin (PCT), interleukin-6 (IL-6) and CRP were associated with outcome in 10/11 (independently in 3), 10/11 (independently in 4) and 7/10 (independently in 2) studies, respectively. PCT had strongest association 1 day after OHCA, IL-6 at ICU admission, and CRP at later timepoints. In general, the inflammatory marker association with outcome was stronger in studies with more severely ill patient populations. Spearman r between AUC and poor outcome proportion was 0.82 for PCT (Fig. 1) and 0.53 for IL-6. Studies reported conflicting results regarding marker association with organ failure severity. Numerous other inflammatory markers were assessed, mostly in single studies.

**Conclusions:** Inflammatory markers are potentially useful for early risk stratification after OHCA. PCT and IL-6 are the most studied markers and reported to have prognostic value during the first 24 h of ICU stay. Prognostic value of the inflammatory markers is dependent on the case mix.

**Reference**

1. Adrie C et al. Circulation 106:562–568, 2002

**Fig. 1 (abstract P016):**
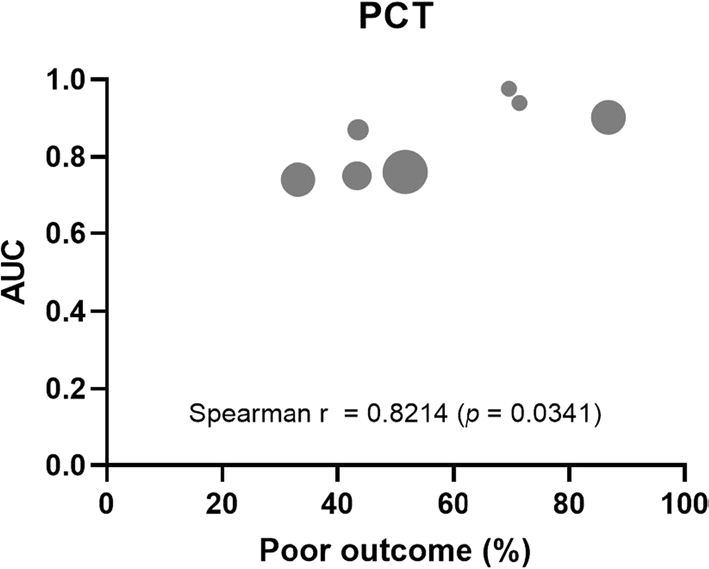
Spearman correlation between procalcitonin’s (PCT) area under receiver operating characteristic curve (AUC) and proportion of patients with poor outcome. Sample size of each study is represented by point size.

## P017

### Fever after OHCA: a post hoc analysis of the FINNRESUSCI study

#### A Holm^1^, M Reinikainen^2^, J Kurola^3^, J Vaahersalo^4^, M Tiainen^5^, T Varpula^4^, J Hästbacka^4^, M Skrifvars^6^

##### ^1^University of Helsinki and Helsinki University hospital, Faculty of Medicine and Department of Emergency Care and Services, Helsinki, Finland, ^2^University of Eastern Finland and Kuopio University Hospital, Kuopio, Finland, ^3^Centre of Prehospital Emergency Care, Kuopio University Hospital, Kuopio, Finland, ^4^Department of Anaesthesiology, Intensive Care and Pain Medicine, Helsinki University Hospital and University of Helsinki, Department of Anaesthesiology, Intensive Care and Pain Medicine, Helsinki University Hospital and University of Helsinki, Helsinki, Finland, ^5^Department of Neurology, Helsinki University Hospital and University of Helsinki, Department of Neurology, Helsinki University Hospital and University of Helsinki, Helsinki, Finland, Helsinki, Finland, ^6^Department of Emergency Care and Services, University of Helsinki and Helsinki University Hospital, Department of Emergency Care and Services, University of Helsinki and Helsinki University Hospital, Helsinki, Finland

*Critical Care* 2022, **26(Suppl 1):** P017

**Introduction:** Fever may occur after out-of-hospital cardiac arrest (OHCA) and some previous data suggests it is associated with outcome. Recent studies show that targeted temperature management (TTM) targeting 33 °C as a mean to avoid fever does not improve functional outcome (FO) [1]. We aimed to assess the prevalence of fever among those not treated with TTM and study associations between fever and FO.

**Methods:** A post hoc analysis of patients who were included in the FINNRESUSCI study but not treated with TTM [2]. The FINNRESUSCI study was an observational cohort study including all patients treated in Finnish intensive care units (ICU) following OHCA in 2010–2011. We defined fever as at least one temperature measurement of ≥ 37.8 °C within 72 h of ICU admission [3]. The primary outcome was favourable FO at 12 months defined as cerebral performance category (CPC) of 1 or 2.

**Results:** This study included 67,428 temperature measurements from 192 patients of whom 87 (45%) experienced fever. Twelve-month CPC was missing in seven patients and 51(28%) patients had favourable FO at 12 months. Neither time in minutes nor area (minutes times degree over threshold) over 37 °C, 37.5 °C, 38 °C, 38.5 °C, 39 °C, 39.5 °C or 40 °C were significantly different in those with favourable FO compared to those with unfavourable FO within the first 24, 48 or 72 h from ICU admission. Fever was not associated with favourable FO at 12 months in a binary logistic regression model (odds ratio (OR) 0.65, 95% confidence intervals (CI) 0.34–1.24, *p* = 0.19) or in a multivariable binary regression model including initial rhythm, witnessed arrest, bystander CPR and delay to return of spontaneous circulation (OR 0.90, 95% CI 0.44–1.84, *p* = 0.77).

**Conclusions:** Half of the patients not treated with TTM develop fever. We found no association between fever and outcome.

**References**

1. Dankiewicz J et al. N Engl J Med 385:1341–1342, 2021
2. Vaahersalo J et al. Intensive Care Med 39:826–37, 2013
3. Obermeyer Z et al. Br Med J 359:j5468, 2017.

## P018

### Mortality risk estimation for peri-operative cardiac arrest and 30-day mortality in preterm infants requiring non-cardiac surgery

#### G Jansen^1^, L Irmscher^1^, T May^2^, R Borgstedt^1^, K Thies^2^, S Rehberg^2^, S Scholz^1^

##### ^1^Protestant Hospital of the Bethel Foundation, University Hospital OWL, University of Bielefeld, Department of Anaesthesiology, Intensive Care, Emergency Medicine, Transfusion Medicine, and Pain Therapy, Bielefeld, Germany, ^2^Protestant Hospital of the Bethel Foundation, University Hospital OWL, University of Bielefeld, Bielefeld, Germany

*Critical Care* 2022, **26(Suppl 1):** P018

**Introduction:** The aim of this study was to develop a risk calculation for 30-day mortality in the context of perioperative anaesthesiological care of preterm infants in non-cardiac surgery.

**Methods:** Monocentric follow-up study of 22,650 paediatric anaesthesias at a German university hospital and level one perinatal center between 2007–2020. Inclusion criteria were age < 37 gestational weeks at the time of surgery. The primary endpoint was 30-day mortality. The data collected included weight at time of surgery, time of surgery and the need for catecholamine therapy. For statistical analysis, univariate and multivariate logistic regressions were used.

**Results:** Between 2007 and 2020, a total of 268 preterm infants underwent surgery. The 30-day mortality was 10.5% (27/268; CI95%: 6.7–14.3) with weight at time of surgery (≥ 2000 g: 1.1%; 1999–1000 g: 10.1%; 999–750 g: 18.2%; < 750 g: 30.3%), time of surgery (7:01–15:00: 7.2%; 15:01–22:00: 11.4%; 22:01–7: 00:30.8%) and the need for catecholamine therapy (22.3%) as significant predictors in the multivariate regression analysis. Table 1 shows an overview of the identified risk factors and the predicted (multivariate regression model) vs. observed mortality (*p* = 0.27).

**Conclusions:** Perioperative 30-day mortality of preterm infants during non-cardiac surgery is higher than previously thought. The risk calculation from the easily ascertainable factors (i.e. low body weight at the time of surgery, time of the surgical intervention and catecholamine therapy) could be a valuable tool for estimating 30-day perioperative mortality in preterm infants and should be validated in larger populations.

**Table 1 (abstract P018):** Predicted and observed perioperative mortality of preterm infants undergoing non-cardiac surgery

Weight (g)	Catecholamine therapy	Time	Predicted Mortality (%)	Observed Mortality (%)
≥2.000	No	7:00-22:00	0.8	1.3
1.000-1.999	No	7:00-22:00	5.4	3.0
	No	22:01-7:00	20.7	16.7
	Yes	7:00-22:00	15.1	22.7
	Yes	22:01-7:00	44.9	50.0
750-999	No	7:00-22:00	8.5	13.3
	No	22:01-7:00	29.6	40.0

## P019

### Right ventricular pressure monitoring in acute ischemic right ventricular dysfunction: an animal model

#### EJ Couture^1^, K Moses^2^, MI Monte Garcia^3^, C Potes^2^, F Haddad^4^, L Gronlykke^5^, F Garcia^2^, E Paster^2^, A Denault^6^

##### ^1^Institut Universitaire de Cardiologie et de Pneumologie de Québec, Anesthesiology and Intensive Care Medicine, Quebec, Canada, ^2^Edwards Lifesciences, Irvine, USA, ^3^Hospital Universitario SAS de Jerez, Intensive Care Medicine, Jerez de la Frontera, Spain, ^4^Stanford University, Cardiovascular Medicine, Stanford, USA, ^5^Copenhagen University Hospital, Anesthesiology, Copenhagen, Denmark, ^6^Montreal Heart Institute, Université de Montréal, Anesthesiology, Montreal, Canada

*Critical Care* 2022, **26(Suppl 1):** P019

**Introduction:** Right ventricular dysfunction is a major cause of morbidity and mortality in intensive care and cardiac surgery. Early detection of right ventricular dysfunction may be facilitated by continuous right ventricular monitoring strategies. The objective is to evaluate the relationship between hemodynamic parameters derived from the right ventricular pressure monitoring and the right ventricular end-systolic elastance (Ees) in a right ventricular ischemic model.

**Methods:** Acute ischemic right ventricular dysfunction was induced in 10 anesthetized pigs by progressive embolization of microsphere in the right coronary artery. Right ventricular hemodynamic performance was assessed using the Ees from a right ventricular conductance catheter during inferior vena cava occlusion.

**Results:** Acute ischemia resulted in a significant reduction in right ventricular Ees from 0.26 (interquartile range: 0.16 to 0.32) to 0.14 (0.11 to 0.19) mmHg/ml, *p* < 0.010), cardiac output from 6.3 (5.7 to 7) to 4.5 (3.9 to 5.2) l/min, *p* = 0.007, mean systemic arterial pressure from 72 (66 to 74) to 51 (46 to 56) mmHg, *p* < 0.001, and SvO_2_ from 65 (57 to 72) to 41 (35 to 45) %, *p* < 0.001). Linear mixed-effect model analysis was used for assessing the relationship between Ees and right ventricular pressure derived parameters. The reduction in right ventricular Ees best correlated with a reduction in dP/dtmax and single-beat Ees. Normalizing dP/dtmax by heart rate resulted in an improved surrogate of Ees.

**Conclusions:** Stepwise decreases in right ventricular Ees during acute ischemic right ventricular dysfunction were tracked by dP/dtmax derived from the right ventricular pressure waveform.

## P020

### Acute cor pulmonale and mortality in mechanically ventilated patients with COVID-19 acute respiratory distress syndrome

#### ED Valenzuela espinoza^1^, P Mercado^2^, R Pairumani^3^, N Medel^4^, E Petruska^3^, D Ugalde^4^, F Morales^3^, J Montoya^4^, D Eisen^4^, C Araya^3^

##### ^1^Hospital Clínico Pontificia Universidad Católica de Chile, Intensive Care Medicine, Santiago, Chile, ^2^Clínica Alemana de Santiago, Departamento de Paciente Crítico, Santiago, Chile, ^3^Hospital Barros Luco Trudeau, Departamento de Paciente Crítico, Santiago, Chile, ^4^Hospital Clínico Universidad de Chile, Departamento de Paciente Crítico, Santiago, Chile

*Critical Care* 2022, **26(Suppl 1):** P020

**Introduction:** Although COVID-19 affects primarily the respiratory system, several studies have shown evidence of cardiovascular alterations and right ventricular dysfunction. Our aim was to evaluated cardiac function and its association with lung function, hemodynamic compromise and mortality.

**Methods:** Prospective, cross-sectional multicenter study in four university-affiliated hospitals in Chile. All consecutive patients with COVID-19 ARDS on mechanical ventilation admitted between April and July 2020 were included. Transthoracic echocardiography was performed within the first 24 h of intubation.

**Results:** Consecutive 140 patients on mechanical ventilation with COVID-19 ARDS were included in the study, the mean age was 57 ± 11 years, PaO2/FiO2 ratio was 155 [IQR 107–177], cardiac output was 5.1 L/min [IQR 4.5–6.2] and 86% of the patients required norepinephrine. ICU mortality was 29% (40 patients). Fifty-four patients (39%) exhibited right ventricle dilation and 20 of them (37%) exhibited acute cor pulmonale (ACP). Eight of twenty (40%) patients with ACP exhibited pulmonary embolism. Patients with ACP had higher norepinephrine requirement, lower stroke volume, tachycardia, prolonged capillary refill time and higher lactate levels. In addition, acute cor pulmonale patients presented lower compliance, higher driving pressure and the presence of respiratory acidosis. Left ventricular systolic function was normal or hyperkinetic in most cases and only thirteen patients (9%) exhibited left ventricular systolic dysfunction (ejection fraction < 45%). In the multivariate analysis acute core pulmonale, PaO2/FiO2 ratio and pH were independent predictors of mortality (Table 1).

**Conclusions:** Right ventricular dilation is highly prevalent in mechanically ventilated patients with COVID-19 ARDS. The presence of acute cor pulmonale is associated with poorer lung function, but only in 40% of patients it was associated to pulmonary embolism. Acute cor pulmonale is an independent risk factor for mortality in the ICU.

**Table 1 (abstract P020):** Principal differences among patients with normal right ventricle, right ventricle dilation without acute cor pulmonale (ACP) and ACP

	All n = 140	Normal RV n = 86	RV dilation n = 34	ACP n = 20
RS compliance, ml/cmH_2_O	33 [26-40]	35 [27-40]	32 [26-42]	28 [20-37]*
PCO_2_, mmHg	43 [39-56]	43 [39-53]	45 [38-57]	55 [43-65]*
pH	7.33 [7.24-7.38]	7.33 [7.26-7.38]	7.35 [7.24-7.40]	7.24 [7.18-7.32]*
NE, mcg/kg/min	0.05 [0.03-0.14]	0.05 [0.03-0.12]	0.04 [0.01-0.08]	0.20 [0.05-0.30]*
LVOT VTI, cm	20 [16-24]	21 [17-24]	18 [16-21] #	16 [14-20]*
TAPSE, mm	20 [18-23]	21 [18-23]	22 [19-24]	16 [13-20]*
ICU mortality	40 (29%)	23 (27%)	3 (9%)	14 (70%)*

## P021

### Right ventricular outflow tract analysis in the critically ill with sepsis

#### E Bowcock^1^, L Schramko^1^, B Gerhardy^2^, S Orde^1^

##### ^1^Nepean Hospital, Intensive Care, Kingswood, Australia, ^2^Nepean Hospital, Respiratory Medicine, Kingswood, Australia

*Critical Care* 2022, **26(Suppl 1):** P021

**Introduction:** Right ventricular (RV)-pulmonary circuit interaction is important to consider in sepsis given the propensity for right ventricular dysfunction (RVD) and pulmonary hypertension (PH). RVD is associated with increased mortality. In PH a pulmonary valve acceleration time (PAT) of < 90 ms and PAT/pulmonary artery systolic pressure (PASP) ratio of < 2 show strong correlation with pulmonary vascular resistance (PVR) of > 3 wood units (WU). Mid-systolic notching predicts worse outcomes in this group. The therapeutic and prognostic significance of altered RVOT flow profiles in sepsis is unknown. Targeted individualised treatment strategies aimed at minimising insult to the pulmonary vasculature and pressure sensitive RV may be of benefit [1]. Analysis of the RVOT flow profile may alert the intensivist to early RVD in sepsis [2].

**Methods:** Single centre, retrospective analysis of a 110 patients with sepsis who had a transthroacic echo (TTE) with RVOT pulsed wave Doppler (PWD) over a 3-year period. Flow profiles were categorised as no notch (NN) versus notched (N). PAT, RVOT velocity time integral and ejection time were measured.

**Results:** PAT was lower in non-survivors (79.2 vs 91.6 ms, p = 0.02) (Fig. 1). A lower PAT/PASP ratio was found amongst non-survivors. Receiver operating curve for PAT/ PASP ratio showed an AUC 0.78 for a cut off value of 1.6.

**Conclusions:** RVOT Doppler flow analysis may be useful in identifying pulmonary vascular dysfunction in sepsis. Further prospective studies are needed to assess its diagnostic and theraputic benefit.

**References**

1. Price LC et al. Crit Care 14:R169, 2010
2. Takahama H et al. JACC Cardiovasc Imaging 10:1268–77, 2017

**Fig. 1 (abstract P021):**
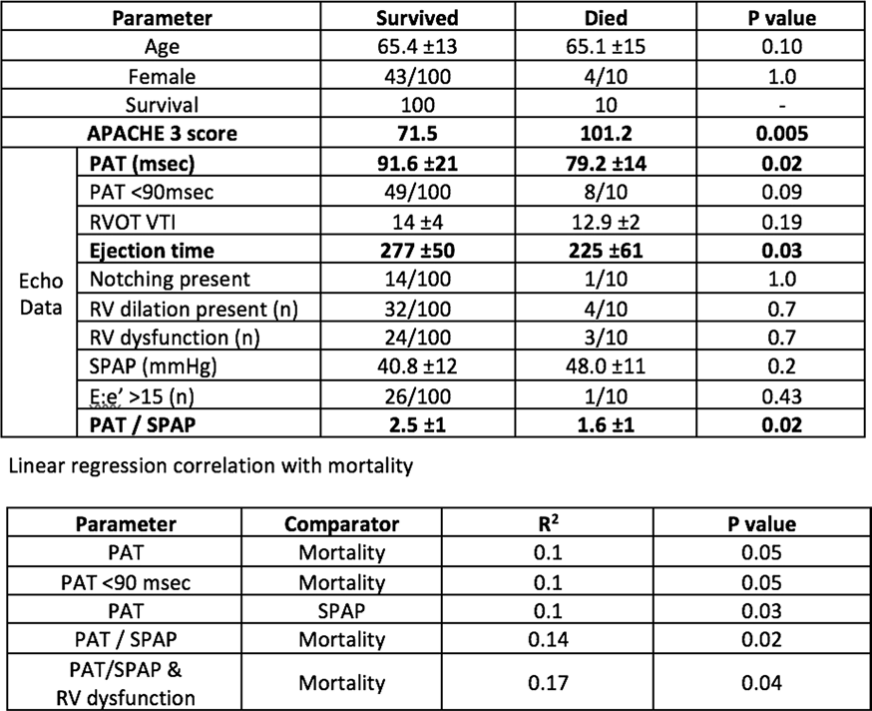
Demographic and echocardiographic parameters in survivors and non-survivors in sepsis

## P022

### Pulmonary artery systolic pressure at admission predicts ICU mortality in elderly critically ill with severe COVID-19 pneumonia

#### M Kurnik, H Božič, P Kolar, A Vindišar, M Podbregar, CE COVID-19 Study Group

##### General Hospital Celje, Department of Internal Intensive Medicine, Celje, Slovenia

*Critical Care* 2022, **26(Suppl 1):** P022

**Introduction:** Point-of-care ultrasound (POCUS) is a useful diagnostic tool in noninvasive assessment of critically ill patients. Mortality of especially elderly patients with COVID-19 pneumonia is high and there is still scarcity of definitive predictors. Aim of our study was to assess the prediction value of combined lung and heart POCUS data on mortality of elderly critically ill patients with severe COVID-19 pneumonia.

**Methods:** Data of patients older than 70 years, with severe COVID-19 pneumonia admitted to 22-bed mixed ICU, level 3, was analysed retrospectively. POCUS was performed on all admitted patients; our parameters of interest were pulmonary artery systolic pressure (PAPs) and diffuse B-line lung pattern (B-pattern).

**Results:** A total of 116 patients (average age 77 ± 5 years) were included. Average length of ICU stay was 10.7 ± 8.9 days. High-flow oxygenation, non-invasive ventilation and invasive mechanical ventilation were at some point used to support 36/116, 39/116 and 75/116 patients respectively. ICU mortality was 59/116 (50.9%). ICU stay was shorter in survivors (8.8 ± 8.3 vs 12.6 ± 9.3 days, *p* = 0.02). PAPs was lower in ICU survivors (32.5 ± 9.8 vs. 40.4 ± 14.3 mmHg, *p* = 0.024) (Table 1). B-pattern was more often detected in non-survivals (35/24 (59%) vs. 19/38 (33%), *p* = 0.005). PAPs and B-pattern were both univariate predictors of mortality. PAPs was an independent predictor of ICU mortality (OR 1.0683, 95%CI: 1.0108–1.1291, *p* = 0.02).

**Conclusions:** PAPs at admission is an independent predictor of ICU mortality of elderly patients with severe COVID-19 pneumonia.

**Table 1 (abstract P022):** B-pattern: diffuse B-line lung pattern

Variable	All (n = 116)	ICU survivors (n = 58)	ICU non-survivors (n = 59)	*p* Value
PAPs, mmHg	36.7±12.9	32.5±9.8	40.4±14.3	0.024
B-pattern, n (%)	54 (47)	19 (33)	35 (59)	0.005
**Logistic regression**	**OR**	**95%CI**	***p* Value**	
PAPs—univariate	1.0680	1.0071-1.1326	0.028	
B-pattern—univariate	2.9167	1.3681-6.2182	0.006	
PAPs—multivariate	1.0683	1.0108-1.1291	0.02	
B-pattern—multivariate	2.8125	0.8258-9.5788	0.10	

CI: confidence interval, ICU: Intensive care unit, OR: Odds ratio, PAPs: pulmonary artery systolic pressure

## P023

### Moderate elevations in positive end expiratory pressure (PEEP) in a patient with ARDS and severe systolic heart failure can decrease oxygen delivery: a case report

#### C David^1^, C Pierrakos^2^, R Attou^2^, K Kaefer^2^, D Velissaris^3^, PM Honore^2^, D De Bels^2^

##### ^1^Institut Jules Bordet, Intensive Care, Brussels, Belgium, ^2^CHU Brugmann, Intensive Care, Brussels, Belgium,^3^University Hospital of Patras, Intensive Care, Pio, Greece

*Critical Care* 2022, **26(Suppl 1):** P023

**Introduction:** Positive end-expiratory pressure (PEEP) may improve oxygenation and left ventricular (LV) function in patients with acute respiratory distress (ARDS). The aim of this case study was to assess the hemodynamic and oxygenation effects of moderate PEEP elevation in an invasively ventilated patient with ARDS and severe systolic LV dysfunction. We hypothesized that moderate PEEP elevations may improve oxygenation, LV function and oxygen delivery (DO_2_).

**Methods:** A 72-year-old patient with severe heart failure and severe ARDS (PaO_2_/FiO_2_: 60) due to *Legionella* infection was assessed. The patient was submitted to invasive ventilation (tidal volume of 5.6 ml/kg of predicted body weight and PEEP of 3 cm H_2_O). PEEP was progressively increased up to 15 cm H_2_O for improving oxygenation. Echocardiographic and hemodynamic variables from Swan-Ganz catheter were obtained before and 20 min after each PEEP modification.

**Results:** At baseline, the patient had a LV ejection fraction of 10%, tricuspid annular plane systolic excursion (TAPSE) of 19 mm and cardiac index of 2.5 l/min/m^2^. PEEP elevation increased oxygen saturation (from 91 to 95%), mixed venous saturation (60% to 72%) and driving pressure (11 to 15 cmH_2_O) but DO_2_ decreased (329 to 311 ml/min/m^2^) (Fig. 1). LV function improved as indicated by left ventricular global longitudinal strain (− 9% to − 14%) and LV myocardial performance index (LV-MPI 0.55 to 0.38). In contrast, right ventricular (RV) function deteriorated as indicated by RV myocardial performance (RV-MPI 0.31 to 0.58). TAPSE/systolic pulmonary artery pressure ratio decreased from 0.32 to 0.11.

**Conclusions:** This case suggests that in a patient with severe systolic heart dysfunction and ARDS, moderate PEEP elevations may deteriorate oxygen delivery despite left ventricular function and oxygenation improvement. RV–pulmonary vascular coupling assessment can be clinical relevant parameter for PEEP choice.

**Consent to Publish**: Written informed consent was obtained from the next of kin.

**Fig. 1 (abstract P023):**
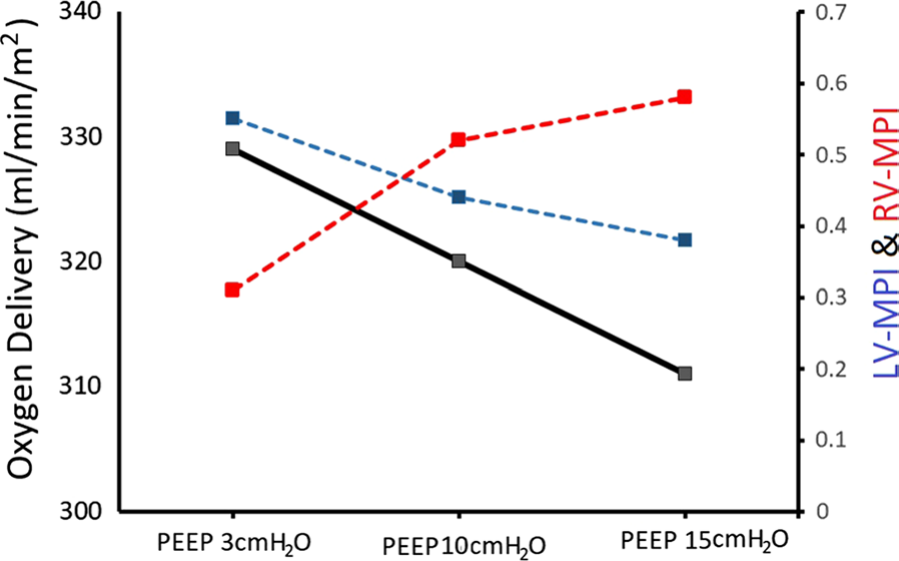
Evolution of cardiovascular and oxygen delivery parameters according to changes in positive end-expiratory pressure (PEEP)

## P024

### Portal vein waveform may predict outcome in patients undergoing corrective surgery for tetralogy of Fallot

#### H Aggarwal^1^, G D Puri^2^, R Ganesan^3^, B Mandal^3^, RM Kumar^4^, SK Thingnam^5^

##### ^1^Sector 12, Cardiac Anaesthesia, Chandigarh, India, ^2^Sector 12, Department of Anaesthesia and Intensive Care, Chandigarh, India, ^3^Sector 12, Anaesthesia and Intensive Care, Chandigarh, India, ^4^Sector 12, Cardiology, Chandigarh, India, ^5^Sector 12, Cardiothoracic and vascular surgery, Chandigarh, India

*Critical Care* 2022, **26(Suppl 1):** P024

**Introduction:** Right ventricle (RV) failure is common after corrective surgery for Tetralogy of Fallot (TOF) and is associated with significant morbidity and mortality. We aimed to determine whether an increased portal vein flow pulsatility fraction (PVPF) was associated with worse clinical outcome.

**Methods:** We conducted a prospective single-centre observational study in patients of all ages undergoing corrective surgery for TOF. PVPF and other commonly used parameters of RV function were assessed at 6 timepoints: intraoperatively: before and after bypass, postoperatively: days 1,2, at extubation and at intensive care unit (ICU) discharge (timepoints 1–6 respectively). PVPFmax was defined as the maximum PVPF obtained in a patient at any time point. Correlation was tested between PVPFmax and mechanical ventilation duration, prolonged ICU stay and mortality.

**Results:** The study included 54 patients of age 3 [2.7] years (median [IQR]) and mortality was in 3 patients. PVPF measurement was feasible in 92.6% of the examinations. Mean values of PVPF were 34.87 ± 13.53, 47.24 ± 18.66, 49.92 ± 21.72, 43.07 ± 19.14, 40.52 ± 16.86 and 28.9 ± 12.8 at time points 1 to 6 respectively. There was weak correlation of PVPFmax with duration of mechanical ventilation and ICU stay (r = 0.286, p = 0.036 and r = 0.296, *p* = 0.030 respectively) and no correlation with mortality. There was a moderate negative correlation of PVPF with RV fractional area change and RV Strain (r =  − 0.488, *p* < 0.001 and r =  − 0.457, *p* < 0.001 respectively) and a strong positive correlation with abnormal hepatic vein waveform(rho = 0.686, *p* < 0.001).

**Conclusions:** PVPF measurement is feasible in the pediatric cardiac surgery patient and an increased PVPF may be associated with right ventricular dysfunction and worse clinical outcome.

## P025

### Portal vein Doppler in high-risk cardiac surgery patients: a multicenter prospective cohort study

#### A Denault^1^, EJ Couture^2^, É De Medicis^3^, JK Shim^4^, M Mazzeffi^5^, RA Henderson^5^, S Langevin^2^, R Dhawan^6^, M Michaud^7^, W Beaubien-Souligny^8^

##### ^1^Montreal Heart Institute, Université de Montréal, Anesthesiology, Montreal, Canada, ^2^Institut Universitaire de Cardiologie et de Pneumologie de Québec, Anesthesiology, Quebec, Canada, ^3^Centre Hospitalier de l’Université de Sherbrooke, Anesthesiology, Sherbrooke, Canada, ^4^Yonsei University College of Medicine, Anesthesiology and Pain Medicine, Seoul, South Korea, ^5^University of Maryland, Anesthesiology, Baltimore, USA, ^6^University of Chicago Medicine, Anesthesiology, Chicago, USA, ^7^Centre Hospitalier de l’Université de Montréal, Anesthesiology, Montreal, Canada, ^8^Centre Hospitalier de l’Université de Montréal, Nephrology, Montreal, Canada

*Critical Care* 2022, **26(Suppl 1):** P025

**Introduction:** Portal vein Doppler pulsatility measured by transesophageal echocardiography is a promising imaging ultrasound biomarker of the hemodynamic impact of right ventricular failure in cardiac surgery. The objective of the study was to determine whether the presence of abnormal portal vein flow pulsatility is associated with a longer duration of invasive life support and adverse post-operative outcomes after cardiac surgery in high-risk patients.

**Methods:** Multicenter cohort study using pulsed-wave Doppler assessments of the portal vein flow before initiation of cardiopulmonary bypass (CPB) (T1) and after CPB separation (T2). Abnormal pulsatility was defined as a portal pulsatility fraction (PPF) of ≥ 50% (PPF50). The primary outcome studied was the cumulative time in perioperative organ dysfunction (T_POD_) requiring invasive life support during the first 28 days after surgery. Secondary outcomes included major post-operative complications.

**Results:** A total of 373 patients were included in the study. PPF50 was present in 22.0% of assessments at T1 and in 24.9% at T2. PPF50 was associated with a longer T_POD_ (T1: median 27 [IQR, 11–72] versus 19 h [IQR, 8.5–42], *p* = 0.02; T2: median 27 [IQR, 11–61] versus 20 h [IQR, 8–42], *p* = 0.006). After adjusting for confounders, only PPF50 at T1 showed significant association with T_POD_. The detection of PPF50 at T2 was associated with a higher rate of major post-operative complications (36.4% versus 20.3%, p = 0.006). Moreover, *de* *novo* PPF50 at T2 was associated with the highest rate of major post-operative complications (40.5%).

**Conclusions:** The presence of PPF50 in cardiac surgery is associated to have a longer duration of life support therapy and complications after cardiac surgery in high-risk patients.

**Consent to Publish**:

Study protocol was approved by the research and ethics committee of each participating institution. Written informed consent was obtained from all subjects.

**Funding**: CARF.

**Acknowledgement:** Study’s Other Collaborators: DPG, DB, JME, CEG, CR, DL, YL, FD, AD, GD.

## P026

### Phenotyping intraoperative hypotension using artificial intelligence in patients having major abdominal surgery

#### K Kouz^1^, L Brockmann^1^, LM Timmermann^1^, A Bergholz^1^, M Flick^1^, L Krause^2^, B Saugel^1^

##### ^1^University Medical Center Hamburg-Eppendorf, Department of Anesthesiology, Hamburg, Germany, ^2^University Medical Center Hamburg-Eppendorf, Department of Medical Biometry and Epidemiology, Hamburg, Germany

*Critical Care* 2022, **26(Suppl 1):** P026

**Introduction:** Intraoperative hypotension is associated with postoperative myocardial injury, acute kidney injury, and death and thus should be avoided. In clinical practice, specific causes of intraoperative hypotension are often neglected. A detailed understanding of underlying hemodynamic alterations would allow treating intraoperative hypotension causally. We sought to use artificial intelligence to identify intraoperative hypotension phenotypes characterized by different underlying hemodynamic alterations in major abdominal surgery patients. We hypothesized that artificial intelligence can identify intraoperative hypotension phenotypes.

**Methods:** We conducted a secondary analysis of intraoperative hemodynamic measurements from a prospective observational study in 100 patients who had major abdominal surgery with general anesthesia including stroke volume index, heart rate, cardiac index, systemic vascular resistance index, and pulse pressure variation measurements. We defined intraoperative hypotension as mean arterial pressure (MAP) ≤ 65 mmHg or MAP between 66 and 75 mmHg when the norepinephrine infusion rate exceeded 0.1 µg/kg/min. To identify intraoperative hypotension phenotypes we used an artificial intelligence algorithm—specifically, hierarchical clustering and then applied the algorithm to pairwise Euclidean distances using Ward’s minimum variance method.

**Results:** There were 615 episodes of intraoperative hypotension in 82 patients. Artificial intelligence revealed six as the optimal number of intraoperative hypotension phenotypes. Based on their clinical characteristics, we labeled the phenotypes as (1) myocardial depression, (2) bradycardia, (3) vasodilation with CI increase, (4) vasodilation without CI increase, (5) hypovolemia, and (6) mixed type (Fig. 1).

**Conclusions:** Artificial intelligence identified six intraoperative hypotension phenotypes. Considering these phenotypes may allow treating intraoperative hypotension causally with specific therapeutic interventions.

**Fig. 1 (abstract P026):**
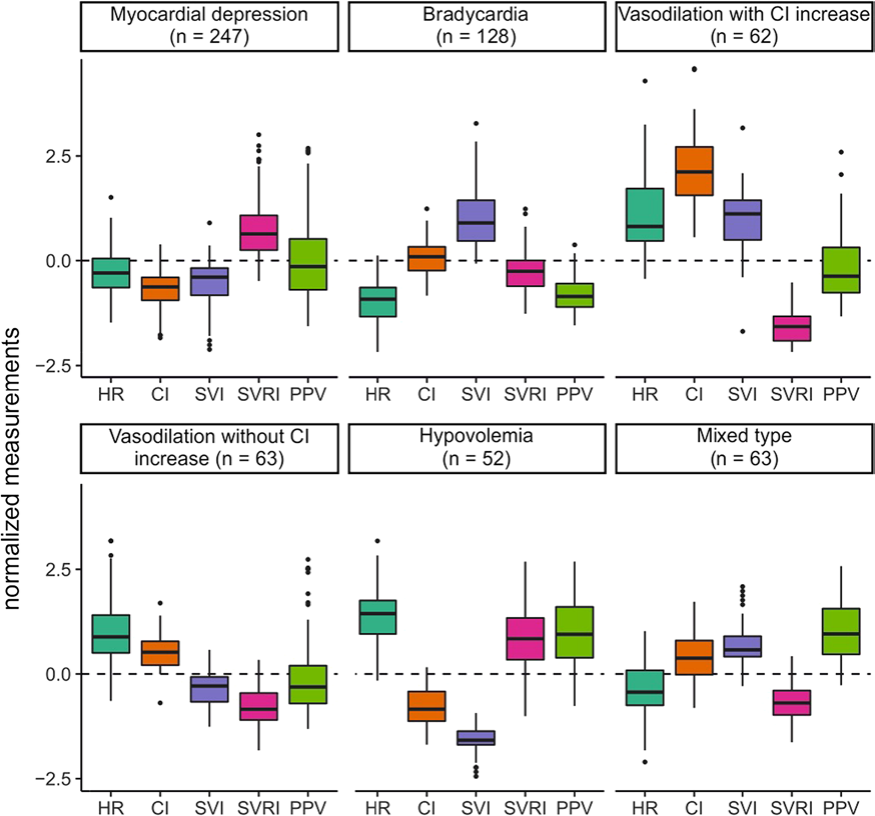
Boxplots showing the normalized mean and standard deviation of stroke volume index (SVI), heart rate (HR), cardiac index (CI), systemic vascular resistance index (SVRI), and pulse pressure variation (PPV) for each phenotype. Data are normalized to a mean of zero (dashed horizontal line) and a standard deviation of one. Boxes represent 25th and 75th percentiles and the range between them is the interquartile range. Inside the boxes, bold horizontal lines represent medians. The whiskers (extensions from the box) indicate the lowest and highest value no further than 1.5 times the interquartile range. Outliers are shown as dots.

## P027

### MRI as research tool for cuff-based physiological measurements

#### L Bogatu^1^, J Hoppenbrouwers^2^, H van den Bosch^2^, S Turco^3^, M Mischi^3^, J Muehlsteff^4^, L Schmitt^4^, P Woerlee^3^, H Korsten^2^, RA Bouwman^2^

##### ^1^Eindhoven University of Technology, Philips Research, Biomedical Diagnostics, Patient Care and Measurements, Eindhoven, Netherlands, ^2^Catharina Ziekenhuis, Eindhoven, Netherlands, ^3^Eindhoven University of Technology, Eindhoven, Netherlands, ^4^Philips Research, Eindhoven, Netherlands

*Critical Care* 2022, **26(Suppl 1):** P027

**Introduction:** Cuff devices offer ample possibilities to modulate blood flow and pulse propagation. Vasculature response to occlusion perturbations may enable measurement of arterial compliance, peripheral resistance, and beat-to-beat BP calibration [1]. However, in standard practice the cuff is still only used for intermittent, largely inaccurate BP measurements. Strong assumptions are required to explain vascular occlusion mechanisms. Additional research modalities are needed for further development of cuff measurements.

**Methods:** In this study, we employed MRI to provide new insights over the influence of the cuff on arterial pulsations. We performed MRI scans on 10 healthy participants to observe vasculature, tissue, cuff interaction. Written informed consent was obtained from the participants.

**Results:** The images provide insights into several assumptions. Unpredictable cuff folding occurs during inflation; compression of the arm is not isotropic (Fig. 1). This effect possibly hinders accurate modulation of arterial transmural pressure. The artery location is subject dependent; oscillations of superficial arteries are likely expressed differently than oscillations of arteries located within subcutaneous fat. Complex tissue compression/displacement occurs under the cuff; arterial volume pulsations might not be equivalent to arm volume pulsations. Artery size is quantified revealing non-linear collapse characteristics and non-uniform collapse across the length of the cuff. No significant changes in arterial properties were detected during two consecutive inflations.

**Conclusions:** These results are useful for improving existing BP measurements and enabling measurement of arterial compliance, peripheral resistance and beat-to-beat BP. The cuff interaction with the vasculature is oversimplified by existing models. MRI is an essential research tool for further development of cuff-based physiological measurements.

**Acknowledgement:** The data collection was registered with MEC-U as nWMO W20.090.

**Reference**

1. Bogatu et al. Sensors 21:5593, 2021

**Fig. 1 (abstract P027):**
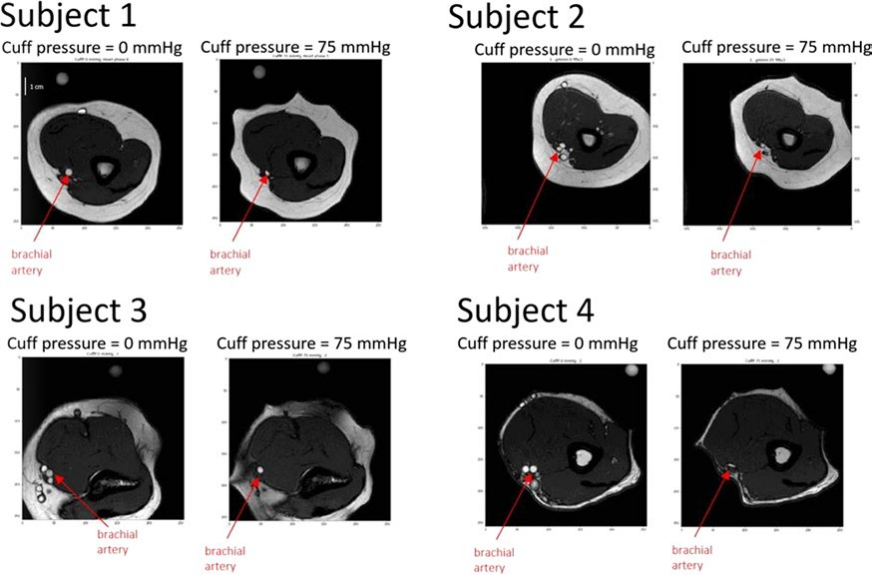
Cross-sectional view of the upper arm during cuff inflation.

## P028

### Norepinephrine infusion titration at the early phase of septic shock: relevance of a transcranial Doppler based protocol

#### C Ben Miled^1^, A Ben Souissi^1^, M Sboui^1^, E Langar^1^, W Fguiri^1^, S Yamoun^1^, A Gharbi^1^, I Saddem^1^, J Hafedh^1^, MS Mebazaa^2^

##### ^1^Mongi Slim University Hospital, Anesthesiology and ICU Department, La Marsa, Tunisia, ^2^Mongi Slim University Hospital, Anesthesiolgy and ICU, La Marsa, Tunisia

*Critical Care* 2022, **26(Suppl 1):** P028

**Introduction:** In septic shock, the surviving sepsis Campaign (SSC) guidelines give clear recommendations about the initial mean arterial pressure (MAP) goals but remain incomplete regarding further goals. The cerebral circulation is concerned by the blood flow redistribution during shock and can be assessed by transcranial Doppler (TCD). The purpose of this study was to assess the contribution of TCD in hemodynamic management during the early phase of septic shock by comparing a personalized TCD-guided hemodynamic goals to the SSC recommendations.

**Methods:** Fifty patients meeting the Sepsis-3 consensus criteria were enrolled and equally randomized into 2 groups. Concerning norepinephrine infusion, we aimed in the standard group to maintain a MAP ≥ 65 mmHg with negative blood lactate level during 72 h after shock onset. For the TCD-guided group, MAP goals and Norepinephrine infusion rate were determined according to TCD measurements to achieve a pulsatility index (PI) < 1.3. Sepsis associated encephalopathy (SAE) was diagnosed using CAM-ICU score > 3 or a GCS deterioration. The main outcome was 28 days mortality.

**Results:** The 2 groups were comparable regarding demographic and initial severity scores. We noticed an increased mortality (*p* = 0.031), higher incidence of SAE (*p* < 10^−3^) and cerebral hypoperfusion (*p* < 10^−3^) in the standard group. In TCD-group, TCD measurements were improved with lower PI (*p* < 10^−4^). MAP was higher in the TCD-group (*p* < 10^−4^) along the study period with no difference in norepinephrine mean infusion rate (*p* = 0.497). No difference in final SOFA scores, duration of mechanical ventilation, ICU length of stay and duration of Norepinephrine infusion were recorded.

**Conclusions:** Monitoring of the cerebral perfusion using TCD is useful in personalizing hemodynamic goals during septic shock and thus improves mortality and neurological outcome.

## P029

### Norepinephrine dose variation related effects on mean arterial pressure: preliminary results from the NOVAMAP study

#### F Moretto^1^, R Shi^2^, JL Teboul^2^, A Pavot^2^, C Lai^2^, N Fage^2^, S Ayed^2^, I Adda^2^, T Pham^2^, X Monnet^2^

##### ^1^Université Paris-Saclay, Service de Médecine Intensive-Réanimation, Hôpital de Bicêtre, Le Kremlin-Bicêtre, France, ^2^Université Paris-Saclay, Le Kremlin-Bicêtre, France

*Critical Care* 2022, **26(Suppl 1):** P029

**Introduction:** Responsiveness to norepinephrine dose variation in terms of mean arterial pressure (MAP) change is highly variable in acute circulatory failure. This preliminary study aimed to investigate the influencing factors of the pharmacodynamic effect of norepinephrine on MAP in critically ill patients.

**Methods:** Monocentric, observational, prospective study conducted at the intensive care unit of Bicêtre Hospital, Paris. Patients with diagnosis of circulatory failure requiring norepinephrine and invasive pressure monitoring were included. To characterize MAP responsiveness, the maximal amplitude of MAP change over the amplitude of norepinephrine dose change (Emax/∆NE) was defined.

**Results:** From January to July 2021, 29 patients presenting 86 episodes of norepinephrine dose change, 55 increases and 31 decreases, were included. The main origin of shock was sepsis in 59 episodes, followed by hypovolemic/hemorrhagic shock in 16 episodes and non-septic vasoplegia in 11 episodes. Septic shock was characterized by lower baseline values of mean (66, 58–86 vs 82, 71–103 mmHg, respectively) and diastolic arterial pressure (50, 44–64 vs 64, 55–78 mmHg, respectively) and a larger amplitude of norepinephrine dose change (0.08, 0.05–0.12 vs 0.04, 0.03–0.06 µg/kg/min, respectively). Emax/∆NE was significantly lower in septic shock (315, 161–590 vs 575, 401–776 vs 446, 336–1119 mmHg/µg/kg/min, respectively, *p* = 0.03, Fig. 1). At multiple logistic regression analysis, preexisting hypertension, body temperature at the episode time and shock etiology were associated with Emax/∆NE (*p* = 0.002), and body temperature and C-reactive protein influenced Emax/∆NE in septic shock episodes (*p* = 0.003).

**Conclusions:** Septic shock seems to be characterized by lower vascular reactivity compared to other shock etiologies and pressure responsiveness is not identical to other distributive shocks.

**Fig. 1 (abstract P029):**
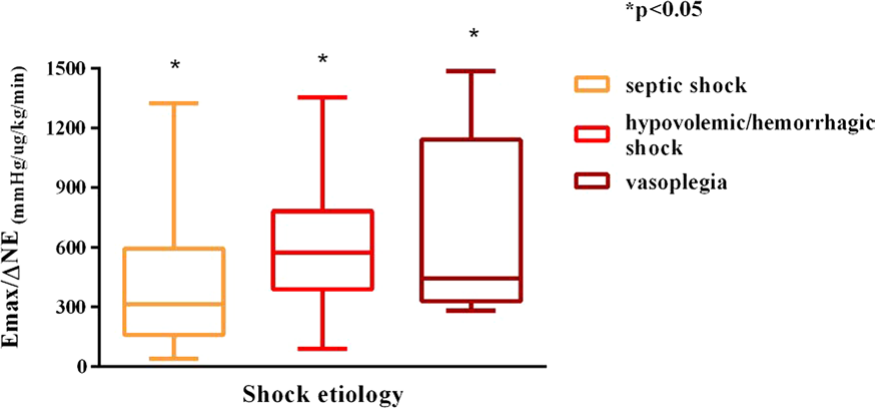
Emax indexed on norepinephrine dose variation (Emax/∆NE), based on shock etiology.

## P030

### Impact of levosimendan use on survival of patients on veno-arterial extracorporeal membrane oxygenation (VA-ECMO) support

#### WS Ng, KB Tang, HP Shum

##### Pamela Youde Nethersole Eastern Hospital, Department of Intensive Care, Chai Wan, Hong Kong, SAR China

*Critical Care* 2022, **26(Suppl 1):** P030

**Introduction:** In refractory cardiogenic shock, VA-ECMO may be employed to maintain end organ perfusion. Measures to hasten weaning from VA-ECMO support are sought to minimize complications. Levosimendan is an inodilator which is shown to facilitate weaning of VA-ECMO in post cardiac surgery patients. Its benefit on patients without cardiac surgery is uncertain. We aim to study the effect of levosimendan on survival of ICU patients receiving VA-ECMO support.

**Methods:** This is a retrospective cohort study carried out in the 24-bed mixed medical/surgical ICU of a regional hospital in Hong Kong from January 1, 2015, to July 31, 2021. Patients admitted for refractory cardiogenic shock or post cardiac arrest requiring peripheral VA-ECMO support were recruited.

**Results:** A total of 51 patients receiving peripheral VA-ECMO support were included. Among them, 19 patients received levosimendan (LEVO), while 32 did not (control). Demographics including age, sex, medical co-morbidities, and the APACHE IV score were not significantly different between groups. Acute myocardial infarction, as the indication for ECMO (84.2% vs 46.9%, *p* = 0.016), and the concomitant use of intra-aortic balloon pump (IABP) (78.9% vs 37.5%, *p* = 0.008) were more commonly found in the LEVO group than the control group. The primary endpoint was 30-day mortality, which was significantly lower in the LEVO group (36.8% vs 68.8%, *p* = 0.026). Kaplan–Meier plots showed improvement of 30-day (*p* = 0.022) and 90-day mortality (*p* = 0.039) for patients who received levosimendan. Statistically significant secondary outcomes included ICU mortality (31.6% vs 62.5%, p = 0.033) and ICU length of stay (6.1 vs 3.3 days, *p* = 0.032). Low total levosimendan dose, defined as less than 140mcg/kg, predicted ICU mortality (AUROC 0.885, 95% CI 0.691–1.000, *p* = 0.009).

**Conclusions:** Levosimendan use was associated with improved 30-day mortality in patients suffering from refractory cardiogenic shock or post cardiac arrest treated with peripheral VA-ECMO.

## P031

### Cardiac output change predicts patient outcome

#### J Sahatjian^1^, MJ Javed^2^, HL Latham^3^, JR Rickelman^4^, DM Hansell^5^

##### ^1^Baxter Healthcare, Newton Center, USA, ^2^Mercy Hospital, St, Louis, USA, ^3^Kansas University Hospital, Kansas City, USA, ^4^Blessing Hospital, Quincy, USA, ^5^Massachusetts General Hospital, Boston, USA

*Critical Care* 2022, **26(Suppl 1):** P031

**Introduction:** Cardiac function is known to be negatively impacted by sepsis. Monitoring Cardiac Output (CO) trends over the course of treatment may provide insight into cardiac function and may be used to predict patient outcome. The goal of this study was to explore the relationship between the change in stroke volume and outcome in critically ill patients.

**Methods:** The Starling Registry study is an observational registry study evaluating trends in CO and SV (Stroke Volume) over time as related to patient outcome (NCT04648293). Patients that exhibited an overall improvement in CO (first CO measurement compared to last CO measurement) were compared to those who did not exhibit improvement.

**Results:** A total of 229 critical care patients received hemodynamic monitoring during their ICU stay across three different hospitals. 48% were female, and the average age was 64 years. 64% of the patients had sepsis, and 17% of patients were positive for COVID. Notably, patients who exhibited an overall improvement in CO exhibited a decrease need for mechanical ventilation (4.8% vs 15%, *p* = 0.041) and a trend toward a decrease in mortality (16.4%) compared to those who did not improve (28.0%, *p* = 0.080) (Fig. 1).

**Conclusions:** We have previously shown that patients who show an improvement in CO in response to the resuscitation exhibited improved outcome. Trending cardiac output over a 1–3 day monitoring period revealed additional usefulness in predicting patients with improved outcome. These results highlight the importance of trending hemodynamics in therapy.

**Fig. 1 (abstract P031):**
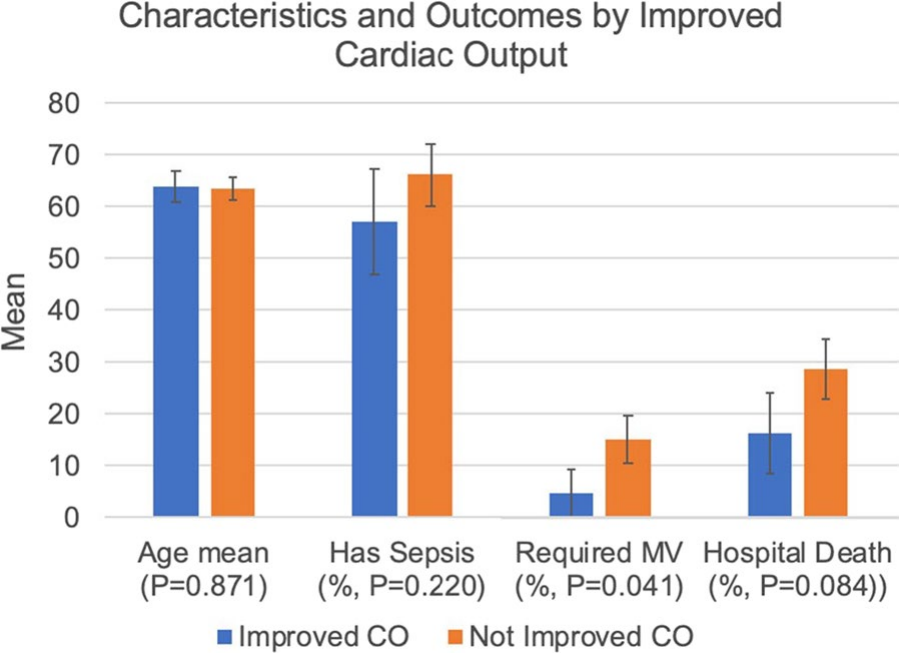
Cardiac output and patient outcome

## P032

### Comparison of cardiac index measurement using continuous wave versus pulsed wave echo-Doppler compared to pulse contour cardiac output

#### J Powys-Lybbe^1^, P Parulekar^1^, P Bassett^2^, S Roques^2^, M Snazelle^2^, T Harris^2^

##### ^1^William Harvey Hospital, Intensive Care Unit, Ashford, UK, ^2^William Harvey Hospital, Ashford, UK

*Critical Care* 2022, **26(Suppl 1):** P032

**Introduction:** This study assesses the accuracy of cardiac output (CO) derived from pulsed wave (PW) velocity time integral (VTI) at the left ventricular outflow tract (LVOT) [1] versus continuous (CW) VTI at the aortic valve (AV) using calibrated PiCCO CO as a reference standard. CO assessments can be used in critical care to aid definition of shock status and guide resuscitation. Stoke volume (SV) and CO measurements can be obtained via transthoracic echocardiography (TTE) as can other methods via thermodilution techniques [2, 3]. CO is measured using the product of cross-sectional area (CSA) and VTI: typically using PW VTI at left LVOT.

**Methods:** We performed a single centre, prospective, observational study in a 15-bed intensive care unit in a UK hospital. Patients had simultaneous measurements of cardiac index (CI) by PiCCO, TTE LVOT PW VTI and TTE AV CW VTI. The mean difference between modalities were measured, with Bland–Altman (B-A) limits of agreement (LOA), and percentage error (PE) calculations performed.

**Results:** Fifty-five patients were assessed with 3 excluded due to AV disease therefore 52 results for all measurements. AV CI mean 2.7 l/min/m^2^ (range 0.78–5.11, s.d. 0.92). LVOT CI mean 2.33 l/min/m^2^ (range 0.77–5.40, s.d. 0.90). PICCO CI mean 2.86 l/min/m^2^ (range 1.50–5.56, s.d. 0.93). AV CW VTI and PICCO mean difference was − 0.16 l/min/m^2^ PE 43.5% (Table 1). LVOT PW VTI and PICCO had a mean difference of − 0.54 PE 38.6%. AV CW VTI and LVOT PW VTI had a mean difference of 0.38 l/min/m^2^ PE 46.0%. There was a non-significant difference between these all these modalities.

**Conclusions:** This study shows that CI derived from both AV CW-VTI and LVOT PW-VTI methods underestimate CI compared to PiCCO, with the AV method having closer values overall to the PICCO method. AV CW-VTI may offer a more accurate assessment of SV.

**References**

1. Evangelista A et al. J Am Coll Cardiol 25:710–6, 1995
2. Critchley LA et al. J Clin Monit Comput 15: 85–91, 1999
3. Zhang Y et al. PLoS ONE 14: e0222105, 2019

**Table 1 (abstract P032):** Table to show Bland-Altman limit of agreement results

Measurements	Mean difference l/min/m^2^	SD difference	95% B-A LOA	Percentage error
AV CI & PICCO CI	−0.16	0.62	(−1.37, 1.05)	1.96 × 0.62/0.5 × (2.70 + 2.86) = 43.5%
LVOT CI & PICCO CI	−0.54	0.51	(−1.53, 0.46)	1.96 × 0.51/0.5 × (2.33 + 2.86) = 38.6%
AV CI & LVOT CI	0.38	0.59	(−0.77, 1.52)	1.96 × 0.59/0.5 × (2.33 + 2.70) = 46.0%

## P033

### Microcirculatory tissue perfusion during general anaesthesia and non-cardiac surgery: an observational study using incident dark field imaging with automated video analysis

#### MF Flick^1^, THS Schreiber^2^, JM Montomoli^3^, LK Krause^4^, HDDB De Boer^5^, KK Kouz^1^, TWLS Scheeren^6^, CI Ince^7^, MH Hilty^8^, BS Saugel^1^

##### ^1^University Medical Center Hamburg-Eppendorf, Department of Anesthesiology, Hamburg, Germany, ^2^University Medical Center Hamburg-Eppendorf, Hamburg, Germany, ^3^Marche Politechnic University, Department of Biomedical Sciences and Public Health, Ancona, Italy, ^4^University Medical Center Hamburg-Eppendorf, Department of Medical Biometry and Epidemiology, Hamburg, Germany, ^5^Martini General Hospital Groningen, Department of Anesthesiology, Groningen, Netherlands, ^6^University Medical Center Groningen, Department of Anesthesiology, Groningen, Netherlands, ^7^Erasmus MC University Medical Center, Department of Intensive Care, Rotterdam, Netherlands, ^8^University Hospital of Zurich, Institute of Intensive Care Medicine, Zürich, Switzerland

*Critical Care* 2022, **26(Suppl 1):** P033

**Introduction:** Handheld vital microscopy allows direct observation of red blood cells within the sublingual microcirculation. Automated analysis allows quantifying microcirculatory tissue perfusion variables—including tissue red blood cell perfusion (tRBCp). We aimed to (1) describe baseline microcirculatory tissue perfusion in patients presenting for elective non-cardiac surgery and (2) test that microcirculatory tissue perfusion is preserved during general anaesthesia and non-cardiac surgery.

**Methods:** In this prospective observational study, we measured sublingual microcirculation using incident dark field imaging with automated analysis at baseline before induction of general anaesthesia, under general anaesthesia before surgical incision, and every 30 min during surgery in 120 elective non-cardiac surgery patients (major abdominal, orthopaedic or trauma, and minor urologic surgery). We also performed measurements in young healthy volunteers.

**Results:** We automatically analysed 3687 microcirculation video sequences. Microcirculatory tissue perfusion variables varied substantially between individuals—but ranges were similar between patients and volunteers. Under general anaesthesia before surgical incision, there were no important changes in tRBCp, functional capillary density, and capillary haematocrit compared to pre-induction baseline. However, total vessel density was higher and red blood cell velocity and the proportion of perfused vessels were lower under general anaesthesia. There were no important changes in any microcirculatory tissue perfusion variables during surgery (Fig. 1).

**Conclusions:** In patients presenting for elective non-cardiac surgery, baseline microcirculatory tissue perfusion variables vary substantially between individuals—but ranges are similar to those in young healthy volunteers. Microcirculatory tissue perfusion is preserved during general anaesthesia and non-cardiac surgery—when macrocirculatory haemodynamics are maintained.

**Fig. 1 (abstract P033):**
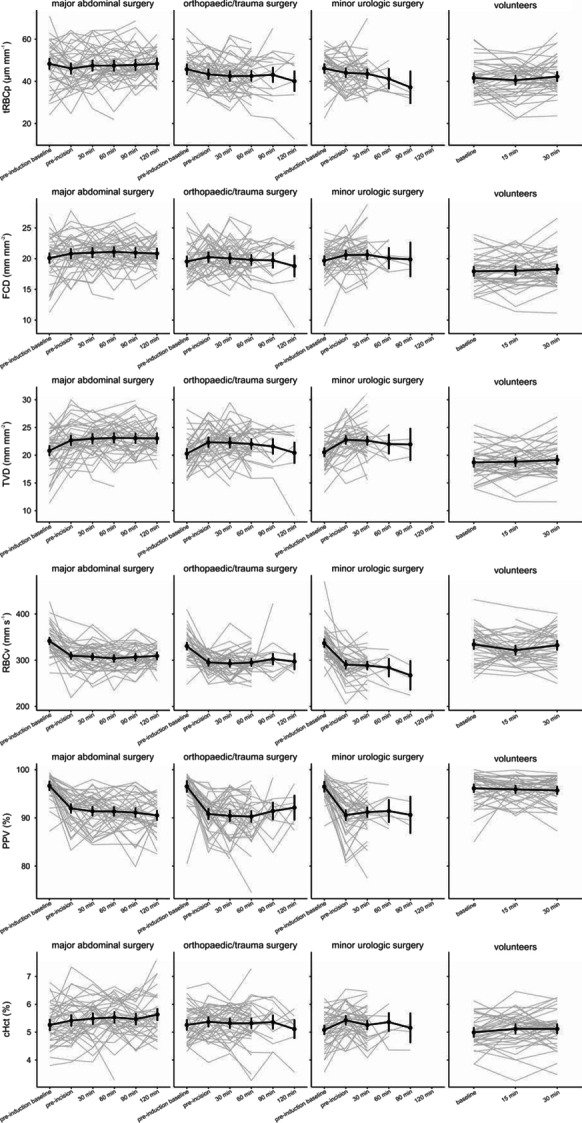
Changes in microcirculatory tissue perfusion variables over time are shown as spaghetti plots for individual patients (gray) and as expected marginal means (black dot) and corresponding 95%-confidence intervals (black vertical lines) of the fixed effects. tissue red blood cell perfusion (tRBCp); functional capillary density (FCD); total vessel density (TVD); proportion of perfused vessels (PVD); red blood cell velocity (RBCv); capillary hematocrit (cHct).

## P034

### Mechanical complications after central venous catheterisation in the ultrasound-guided era: a prospective multicentre cohort study of 12 667 procedures

#### M Adrian^1^, O Borgquist^1^, T Kröger^2^, E Linné^3^, P Bentzer^3^, M Spångfors^4^, A Holmström^5^, J Åkeson^5^, R Linnér^1^, T Kander^5^

##### ^1^Skåne University Hospital, Cardiothoracic Surgery, Anaesthesia and Intensive Care, Lund, Sweden, ^2^Lund University, Medical Faculty, Lund, Sweden, ^3^Helsingborg Hospital, Anaesthesia and Intensive Care, Helsingborg, Sweden, ^4^Kristianstad Hospital, Anaesthesia and Intensive Care, Kristianstad, Sweden, ^5^Skåne University Hospital, Anaesthesia and Intensive Care, Malmö, Sweden

*Critical Care* 2022, **26(Suppl 1):** P034

**Introduction:** We aimed to determine the incidence of mechanical complications and to identify associated independent risk factors in a healthcare system where real-time ultrasound guidance is clinical practice for central venous access.

**Methods:** All central venous catheter insertions in patients ≥ 16 years at four emergency care hospitals between 2 March 2019 and 31 Dec 2020 were eligible for inclusion. During the study period, dedicated collaborators at each study site continuously reviewed the insertion records and chest X-rays. The primary outcome measures were mechanical complications that occurred within 24 h after catheterisation. Multivariable logistic regression analysis was used to determine associations between independent variables and major mechanical complications defined as pneumothorax, arterial catheterisation, major bleeding, serious cardiac arrhythmia and persisting nerve injury. The study was registered at clinicaltrials.gov (NCT03782324) and the study protocol was published in September 2019 [1].

**Results:** In total, 12 667 central venous catheter insertions were prospectively included. The incidence of mechanical complications was 7.7% (95% exact binomial confidence interval [CI] 7.3–8.2), out of which 0.4% (95% CI 0.3–0.5) were major mechanical complications. Patient BMI < 20 kg/m^2^ (OR 2.63 [95% CI 1.20–5.32]), male operator sex (OR 2.65 [95% CI 1.36–5.57]), limited operator experience (OR 3.12 [95% CI 1.71–5.60]) and number of skin punctures (OR 2.11 [95% CI 1.58–2.72]) were associated with a higher risk for major mechanical complications (Table 1).

**Conclusions:** In a healthcare system where real-time ultrasound guidance is clinical practice for central venous access, the incidence of major mechanical complications was found to be low. Patient BMI < 20 kg/m^2^, male operator sex, limited operator experience, and more than one skin puncture were identified as independent risk factors of major mechanical complications.

**Reference**

1. Adrian M et al. BMJ Open 9: e029301, 2019

**Table 1 (abstract P034):** Results. (1) Compared to patients with BMI 20-30 kg/m^2^, (2) Compared to operators that had performed > 100 CVC insertions at the beginning of the study period

Independent variables	Odds ratios	95% Confidence intervals	*p* Values
BMI < 20 (1)	2.63	1.20–5.32	0.010
BMI ≥30 (1)	0.77	0.34–1.57	0.488
Positive pressure ventilation	0.75	0.41–1.35	0.330
Male operator sex	2.65	1.36–5.57	0.007
Limited operator experience (2)	3.12	1.71–5.60	< 0.001
No. of skin punctures	2.11	1.58–2.72	< 0.001
Observations = 10 634			

## P035

### Role of N-terminal pro-B-type natriuretic peptide in early surgery of infective endocarditis due to acute heart failure

#### H Koltunova

##### Amosov National Institute of Cardiovascular Surgery National Academy of Medical Sciences of Ukraine, Anesthesiology, Kyiv, Ukraine

*Critical Care* 2022, **26(Suppl 1):** P035

**Introduction:** We assessed the role of N-terminal pro-B-type natriuretic peptide (NT-proBNP) levels in patients hospitalized for infective endocarditis (IE) for emergent valve surgery due to acute heart failure (AHF).

**Methods:** The study enrolled 100 patients, who underwent valve surgery for IE between 2019 and 2021. All patients were divided into groups according to the degree of preoperative heart failure by NYHA classification. For each group the limit values of NT-proBNP were determined. The predictors of AHF were analyzed, and clinical results of patients with non-AHF (n = 44) were evaluated and compared.

**Results:** Inflammatory pathology of the lungs (odds ratio [OR] 3,37; *p* = 0.003), aortic valve infective endocarditis (OR 2.97; *p* = 0.003), nosocomial infective endocarditis (OR 2,14; *p* = 0.049), vancomycin resistance (OR 2,25; *p* = 0.032), linezolid resistance (OR 2,34; *p* = 0.026) were risk factors for preoperative AHF in patients with IE. Limit values of NT-proBNP in the preoperative period in patients with IE depending on the class of NYHA: group I (n = 20)—less than 300 pg/ml, group II (n = 24)—351–1500 pg/ml, group III (n = 31)—1501–6500 pg/ml, group IV (n = 25)—more than 6500 pg/ml. Clinical signs of AHF were detected in 11 (35%) patients in group III with an average value of NT-proBNP—3190.4 ± 280.8 pg/ml. In group IV with an average value of NT-proBNP—20,150.3 ± 1961.2 pg/ml—preoperative AHF had 16 (64.0%) patients. The correlation analysis revealed a significant relationship between the degree of preoperative AHF and hospital mortality: in group III hospital mortality was—2 (6.5%) cases, in group IV—3 (12%) cases (X^2^ = 18.42, *p* < 0.001).

**Conclusions:** Estimation of the NT-proBNP level allows timely hospitalization and assessment of patients with infective endocarditis, offers adequate preoperative preparation in the intensive care unit with consequent emergent cardiac surgery.

## P036

### Implementation of serum soluble ST2 in pediatric cardiology practice

#### A Bidzhiev^1^, R Tepaev^1^, Y Savluk^1^, V Lastovka^2^, E Basargina^3^, N Aliabeva^4^

##### ^1^National Medical Research Center for Children’s Health, Pediatric Intensive Care Unit, Moscow, Russian Federation, ^2^Morozov Children’s Hospital, Urgent Cardiac Surgery Department, Russian Federation, ^3^National Medical Research Center for Children’s Health, Cardiology Department, Moscow, Russian Federation, ^4^National Medical Research Center for Children’s Health, Clinical Laboratory, Moscow, Russian Federation

*Critical Care* 2022, **26(Suppl 1):** P036

**Introduction:** There are still lack of usefulness biomarkers of heart failure in pediatric cardiology practice. Soluble ST2 is one of the most promising laboratory marker of heart failure. It’s already widely used for diagnostic and prognostic goals in adults [1], but it’s not much investigated in children [2]. We suppose serum soluble ST2 is able to become a new cardiac biomarker of cardiac muscle injury, remodeling and fibrosis, and heart failure outcomes stratification in pediatric population.

**Methods:** Research was performed on the base of Pediatric Intensive Care Unit and Cardiology department of National Medical Research Center for Children’s Health, Moscow. Sera from 44 patients, 20 female and 24 male, in age from 8 months up to 17 years and 11 months (median age 7 years and 2 months old) with confirmed dilated and hypertrophic cardiomyopathy were taken. For all patients levels of soluble ST2, pro-BNP were determined and ECHO was performed for ejection fraction (EF) assessing.

**Results:** Both pro-BNP and ST2 showed direct link with severity class of Chronic Heart Failure assessed by NYHA classification. However, moderate negative correlation (r =  − 0.61, *p* value = 0.00019) between ST2 and EF was proofed by Pearson’s correlation test, meanwhile there was no strong correlation between pro-BNP plasma level and ECHO results.

**Conclusions:** Significantly higher plasma level of serum soluble ST2 were found in patients with reduced EF (< 55%). In pediatric patients with DCMP and HCMP ST2 plasma level seems could be used as cardiac function marker. For assessing diagnostic value of ST2 in children with cardiac pathology as a marker of patient response to therapy, future investigations should be done.

**References**

1. Villacorta H et al. Arq Bras Cardio 106: 145–152, 2016
2. Meeusen JW et al. Clin Biochem 48: 1337–1340, 2015.

## P037

### Factors affecting intensive care unit readmission among patients with ventricular assist device: ten years’ experience

#### BM Yurtsever^1^, N Akovalı^1^, H Şahintürk^1^, A Torgay^1^, P Zeyneloglu^2^

##### ^1^Baskent University Faculty of Medicine, Anesthesiology and Critical Care, Ankara, Turkey, ^2^Baskent University Faculty of Medicine, Anesthesiology, Ankara, Turkey

*Critical Care* 2022, **26(Suppl 1):** P037

**Introduction:** Mechanical support systems are needed due to insufficient number of donor hearts and patients are admitted to ICU after ventricular assist device (VAD) implantation. We aimed to analyze factors associated with ICU readmission among patients with VAD.

**Methods:** After Ethics Committee approval, perioperative data of patients who underwent VAD between November 2008 and December 2018 at Baskent University Faculty of Medicine were analyzed retrospectively. The reasons for ICU readmission and association of perioperative factors with mortality were evaluated.

**Results:** Eighty-nine patients underwent VAD implantation. Out of the 89 patients, 76 were male with a mean age of 46.1 ± 16.5 years. After discharge from ICU, 53 (59.6%) patients were readmitted to hospital, and 44 (49.4%) were readmitted to ICU due to complications. The mean time between readmission to ICU after discharge was 221.1 ± 207.5 days. Out of 89 patients, 23.6% of them were readmitted to ICU due to device-related problems, 14% due to bleeding and 11.4% due to infection. At ICU admission the mean APACHE II score was 10.4 ± 8.9, and the SOFA score was 4.2 ± 3.8. The need for mechanical ventilation (MV) was observed in 14 (31.8%) patients and the mean duration of MV was 4.4 ± 14.5 days. The incidence of acute kidney injury (AKI) was 25% and 22.7% of them needed renal replacement therapy (RRT). In multivariate regression analysis, need for prolonged postoperative mechanical ventilation (PPMV) (OR: 11.439, CI: 1.046–125.096, *p* = 0.046); need for RRT before VAD implantation (OR: 0.36, CI: 0.001–0.911, *p* = 0.044) were found to be risk factors for ICU readmission. Need for PPMV was associated with mortality (OR: 4.882, CI: 1.334–17.873, *p* = 0.017). 30-day survival was 76.4% and 1-year survival was 62%.

**Conclusions:** ICU readmission after VAD implantation occurred almost half of the patients. Requirement for RRT before VAD implantation and PPMV were associated with ICU readmission.

## P038

### Echocardiographic technics for optimal atrio-ventricular (AV) interval election in dual-chamber pacemaker

#### O Moreno Romero^1^, M Muñoz Garach^2^, MT Cruces Moreno^1^, P Fernandez Morales^1^

##### ^1^Hospital Universitario Clínico San Cecilio, Intensive Care Unit, Granada, Spain, ^2^Hospital Universitario Clínico San Cecilio, Critical Care, Granada, Spain

*Critical Care* 2022, **26(Suppl 1):** P038

**Introduction:** Dual-chamber pacemaker allows the physician to ensure the best diastolic function and filling time by calculating the optimal atrioventricular interval to achieve a right systolic function for improving the clinical status of the patients with AV disorders.

**Methods:** Prospective study in a two-month period (1 May-30 June 2019) in which we registered all patients with acute AV block admitted in our ICU of 20 beds and with an indication of dual chamber pacemaker implantation. A routinary echocardiography was performed by our cardiac estimulation unit physicians (intensivists) in the next 6 h after the implantation (max). Two techniques were applied:

- Calculating the AV by the Ritter's Method, using a short-AV interval of 50 ms and a long-AV interval of 200 ms. In all patient we assured using the mitral Doppler that there was neither E-A fusion nor truncated A wave.
- Measuring the Time Velocity Integral (TVI) of the Left Ventriclular Outflow Tract (LVOT) at different AV intervals selected (100, 150, 180 and 200 ms), making the average of 3 cardiac cycles of each interval. The average time in applying both methods was noted.

**Results:** Thirty-one patients with cardiac arrhythmia were admitted in the study period. 19 (61.3%) with AV disorders 14 (45.2%) with dual-chamber pacemaker implantation. Only 7 (22.6%) patients fullfill the inclusión criteria (AV block and US in next 6 hoursmaximmum) and were admitted in our study. 5 male (71.4%), mean age 72 ± 8.5 years. Average times applied: with Ritter's method, 7.5 min (IC 95:5.2–9.8) and with TVI method, 9.7 min (IC95:7.5–11.9).Complete results on Fig. 1.

**Conclusions:** We demonstrate the high variability of AV interval amongst patients. The use of bedside echocardiography in ICU in these patients is an assumable procedure, technically and temporarily, and it may facilitate the selection of the optimal AV interval after a dual chamber pacing implantation.

**Fig. 1 (abstract P038):**
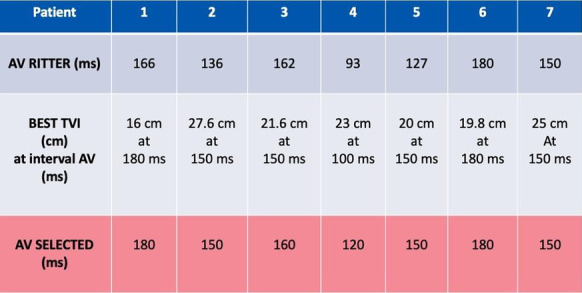
Results

## P039

### CO_2_ derived parameters from central venous blood sampling: correlation with severity and prognosis. a case series of 22 patients in a general ICU.

#### A Casazza, P Cornara, E Bellazzi, F Bonomi, D Ciprandi, R Preda, R Vanzino, MP Storti

##### ASST Pavia, Anaesthesia and Intensive Care Vigevano, Vigevano, Italy

*Critical Care* 2022, **26(Suppl 1):** P039

**Introduction:** DpCO_2_ (venous to arterial pCO_2_ difference) is suggested to be a CO adequacy marker in patients affected by circulatory shock; is still debated, instead, if DpCO_2_/Ca-vO_2_ (DpCO_2_ to O_2_ arterio-venous content difference ratio) is a RQ surrogate and a global anaerobic metabolism marker [1].

**Methods:** We calculated ΔpCO_2_ andΔpCO_2_/Ca-cvO_2_ (ΔpCO_2_ to arterio-central venous O_2_ content difference ratio) at admission using data from arterial and central vein blood sampling and we analyzed relationship with SOFA score and arterial lactate level at admission, Catecholamine Index (C.I.) during ICU stay and ICU mortality. We used linear regression analysis and ROC curve to study relationship.

**Results:** ΔpCO_2_ showed strong correlation with SOFA score (R^2^ 0.59, *p* < 0.0001) and C.I. (R^2^ 0.63, *p* < 0.0001) and was found to be an accurate test in predicting SOFA score > 6 (AUC 0.89, best cut-off value 8 mmHg, sensitivity 77% and specificity 100%) and C.I. > 10 (AUC 0.89, best cut-off value 7 mmHg, sensitivity 86% and specificity 87%). ΔpCO_2_/Ca-cvO_2_ showed good correlation with SOFA score (R^2^ 0.26 *p* < 0.05) and with C.I. (R^2^ 0.47 and *p* < 0.05). The test had moderate accuracy in predicting SOFA score > 6 (AUC 0.80, best cut-off value of 1.95 mmHg/ml with sensitivity 69% and specificity 89%) and better accuracy in predicting lactate level > 2 mmol/l (AUC 0.82, best cut-off value of 1,75 mmHg/ml, sensitivity 100% and specificity 71.4%).

**Conclusions:** ΔpCO_2_ is strongly related with circulatory impairment; ΔpCO2/Ca-cvO2 can be considered, if not a RQ surrogate at all, a decoupling index between macro-circulation and O_2_ extraction. Both parameters are statistically significant higher in patients died in ICU. In our series best cut-off values are higher than those suggested in previous study, probably due to the use of central rather than mixed venous blood sampling [2].

**References**

1. Gavelli F et al. J Thorac Dis S11:1528–37, 2019
2. Cavaliere F et al. Minerva Anestesiol 85:1308–14, 2019

## P040

### Central venous-to-arterial carbon dioxide tension in critically ill COVID-19 patients

#### K Kaefer^1^, C David^2^, D De Bels^1^, C Pierrakos^1^, S Fratino^3^, A Cudia^3^, D De Backer^4^

##### ^1^CHU Brugmann, ICU, Laeken, Belgium, ^2^Institut Jules Bordet, ICU, Brussels, Belgium, ^3^CHIREC—Hôpital Delta, ICU, Brussels, Belgium, ^4^CHIREC Hospitals—Université Libre de Bruxelles, ICU, Brussels, Belgium

*Critical Care* 2022, **26(Suppl 1):** P040

**Introduction:** Critically ill patients with coronavirus disease 2019 (COVID–19) may present severe tissue perfusion abnormalities. The mixed venous-to-arterial carbon dioxide tension difference (P_va_CO_2_) is an easily derived parameter identifying insufficient tissue perfusion. The purpose of this study was to evaluate the clinical relevance of high values of P_va_CO_2_ in COVID − 19 patients early after their admission to intensive care unit (ICU). We speculated that high P_va_CO_2_ values might be associated with poor outcome in critically ill COVID–19 patients.

**Methods:** This was a retrospective study conducted in two independent centers of Belgium. We included patients treated in the first wave of the national outbreak with available P_va_CO_2_ within 3 days of admission and without severe hypercapnia (PCO_2_ > 75 mmHg). The highest value was registered. Normal values were considered ≤ 6 mmHg, moderate elevations 7 − 9 mmHg, and high elevations > 9 mmHg. The primary outcome was ICU discharge alive and secondary outcome mortality at 28 days.

**Results:** Seventy-three patients were included with median age of 60 years (IQR: 52 to 68), and simplified acute physiology score II (SAPS II) 26.1 (IQR: 19.8 to 29.5). Fifty-three (76%) patients needed invasive ventilation within 24 h after ICU admission (12 h (IQR: 12 to 24). The worst ratio of partial pressure arterial oxygen to the fraction of inspired oxygen (PaO_2_/FiO_2_) within 3 days after admission was 151 (IQR: 86 to 243) and P_va_CO_2_ 6 mmHg (IQR: 5 to 9). P_va_CO_2_ > 9 mmHg was associated with longer ICU stay (ICU free days: 0 days (0 − 0) vs 1 day (0 − 18), *p* = 0.02), independently of PaO_2_/FiO_2_ and SAPS II score (HR: 0.21, 95% − CI: 0.05 − 0.92, *p* = 0.04) (Fig. 1, Panel A), but not to 28 days mortality (HR:1.71, 95% − CI: 0.48 − 5.91, *p* = 0.41) (Fig. 1, Panel B).

**Conclusions:** In COVID-19 patients, high P_va_CO_2_ values early after ICU admission are associated with prolonged ICU stay independently of hypoxemia and disease severity.

**Fig. 1 (abstract P040):**
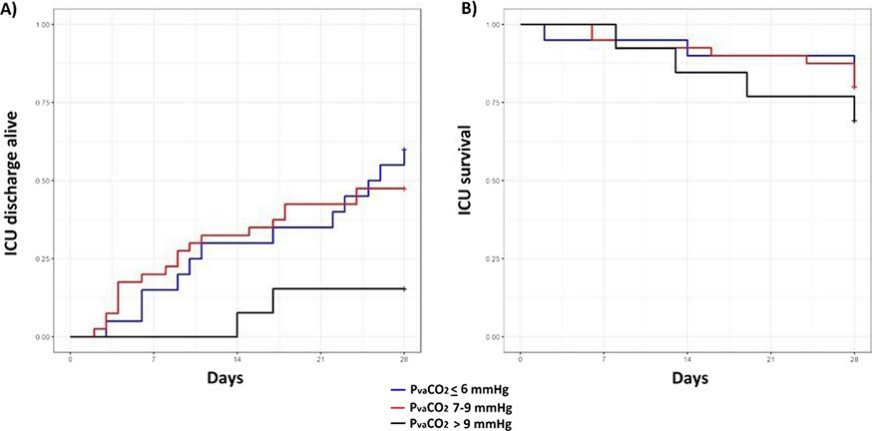
PvaCO_2_ and outcome probability of ICU discharge (**A**) and survival (**B**) at 28 days

## P041

### Dynamics and viability of venous blood samples in critical care patients

#### KD Damgaard^1^, SF Fagerberg^2^, MK Pedersen^1^, ML Lumholdt^2^

##### ^1^Regionshospital Nordjylland, Anesthesia, Hjoerring, Hjoerring, Denmark, ^2^University Hospital, Anesthesia and Intensive Care, Aalborg, Denmark

*Critical Care* 2022, **26(Suppl 1):** P041

**Introduction:** During the COVID-19 pandemic laboratory recourses has been exhausted, leading to longer waiting times before blood samples are delivered for sample analysis. Not much is known about how blood sample parameters behave when stored in room temperature or at ice for prolonged time. Here we investigate the viability and clinical usability of blood samples drawn from critical care patients admitted ICU, and examine blood sample stability and dynamics over time.

**Methods:** Abnormal venous gas blood samples drawn from 30 patients admitted to the ICU at Northern Regional Hospital, were included in the study. 20 samples were drawn per patient at time zero. 9 samples were stored on ice and 9 samples at room temperature. Two samples were controls. Samples were turned every 5 min, and parameters (pH, v-PO_2_, v-PCO_2_, v-lactate, pH, and base excess) were measured over the course of 180 min. Values were compared using 2-way ANOVA, and sample means compared as non-inferiority tests, with allowable lower limits for sample mean-mean differences.

**Results:** We find that values for pH, PCO_2_ and PO_2_ were within an acceptable margin for clinical decision-making until 162 min after time-zero when stored on ice, and for 72 min after time-zero when stored in room temperature. Additionally pH, PCO_2_ and PO_2_ follow a linear regression model during the initial 180 min for both ice stored and room stored samples, allowing for the conversion to values at time-zero.

**Conclusions:** Blood samples from critical care patients can be used as basis for clinical decision-making until 72 min after they are drawn when stored in room temperature, and until 162 min when stored on ice. These data suggest that there is a window of at least 60 min where blood samples can remain in room temperature before analysis and still be used for clinical decision-making.

## P042

### Concordance of oxygen extraction values, arteriovenous oxygen difference and shunt calculated from venous gases and SpO_2_ compared with arteriovenous gases

#### LA León-Guerrero^1^, A Mondragon-Cardona^1^, JD Charry^2^, A Muñoz-Tovar^1^

##### ^1^Universidad SurColombiana, Critical Medicine and Intensive Care, Neiva, Colombia, ^2^Universidad SurColombiana, Neiva, Colombia

*Critical Care* 2022, **26(Suppl 1):** P042

**Introduction:** Calculation of perfusion and oxygenation indicators often includes the simultaneous measurement of arterial and venous gases in critically ill patients. This entails a large amount of supplies and inconvenience caused to patients who require frequent arterial blood sampling, a situation that is aggravated if there is no arterial line, as is the case in developing countries. The objective of this study was to determine the degree of correlation and concordance of the gasimetric values of arteriovenous oxygen difference, oxygen extraction and intrapulmonary shunt calculated with venous gases and pulse oximetry compared with the calculations performed in arteriovenous gases.

**Methods:** One hundred hospitalized patients from intensive care units in Colombia were evaluated. Adult patients with medical or surgical pathologies were included. All patients underwent arterial and venous gasimetry upon admission and 6 h later, and arterial and venous gas measurements were performed in all of them, together with the recording of pulse oximetry at the time of sample collection.

**Results:** Average age of 63.3 years, 65% was men, an average APACHE of 10.75 (95% CI: 9.3–12.1) and a SOFA of 5.2 ( 95% CI: 4.5–6.06). An excellent correlation was found between the values of arteriovenous oxygen difference and oxygen extraction measured with venous gases and pulse oximetry compared with arteriovenous gases (*p* < 0.00001). An excellent agreement was also found for the values in the case of the arteriovenous oxygen difference; and for oxygen extraction. In the case of the intrapulmonary shunt, a good correlation was also found between the two measurements with a good agreement (*p* < 0.00001).

**Conclusions:** The calculation of the values of arteriovenous oxygen difference, oxygen extraction and intrapulmonary shunt can be performed from the venous blood gas only and the pulse oximetry taken at the same time without the need to take an arterial sample. This can reduce costs and inconvenience for critically ill patients.

## P043

### Correlation and concordance of the excess base with the hydrogen ion delta in critically patients from a reference center in Colombia

#### LA León-Guerrero^1^, A Mondragon-Cardona^1^, JD Charry^2^, A Muñoz-Tovar^1^

##### ^1^Universidad SurColombiana, Critical Medicine and Intensive Care, Neiva, Colombia, ^2^Universidad SurColombiana, Neiva, Colombia

*Critical Care* 2022, **26(Suppl 1):** P043

**Introduction:** The hydrogen ion delta was described for quantifying the degree of acidosis or alkalosis originated in determinants other than CO_2_, that is, the metabolic component of the acid–base state. The indicator has not been compared with the excess base, for which the present study was developed. The objective of this study is determine the degree of correlation between the base excess and the delta of hydrogen ions and the concordance for the diagnosis of acid–base disorders of metabolic origin.

**Methods:** One hundred hospitalized patients from intensive care units of Colombian, adult patients with medical or surgical pathologies were included. All patients underwent arterial and venous blood gas analysis upon admission and 6 h later, and measurements of base excess and delta of hydrogen ions were performed at the same time. The group of patients was evaluated during their stay in the intensive care unit. Correlation of the base excess and the delta of hydrogen ions using Pearson's coefficient and Kappa coefficient.

**Results:** Average age of 63.3 years, 65% was men average APACHE of 10.75 (95% CI: 9.3–12.1) and a SOFA of 5.2 (95% CI: 4.5–6.06). A significant correlation was found between the base excess and the delta of hydrogen ions. For the diagnosis of metabolic acidosis, the excess base and the hydrogen ion delta, an excellent agreement was found with a Kappa of 0.91 (*p* < 0.0001). The change in the delta of hydrogens and the change in the excess base between the first and second samples showed a significant area under the ROC curve for the prediction of mortality. (for the delta of hydrogens AUC: 0.779; for the base excess AUC: 0.70).

**Conclusions:** There is a good correlation between the values of base excess and delta of hydrogen ions, and the concordance for the diagnosis of acid–base imbalances is excellent. The change in delta H and excess base in the first 6 h is a predictor of mortality. The hydrogen ion delta is a useful tool in describing the acid–base status of critically ill patients.

## P044

### Lactate and lactate-to-pyruvate ratio in critically ill COVID-19 patients: a pilot study

#### AG Vassiliou^1^, S Tsipilis^2^, C Keskinidou^2^, C Vrettou^2^, E Jahaj^2^, C Routsi^2^, SE Orfanos^2^, A Kotanidou^2^, I Dimopoulou^2^

##### ^1^National and Kapodistrian University of Athens, 1st Department of Critical Care Medicine & Pulmonary Services, GP Livanos and M Simou Laboratories, Athens, Greece, ^2^National and Kapodistrian University of Athens, Athens, Greece

*Critical Care* 2022, **26(Suppl 1):** P044

**Introduction:** A limited number of coronavirus disease-19 (COVID-19) cases may require treatment in an intensive care unit (ICU). Arterial blood lactate levels are routinely measured in the ICU to estimate disease severity, predict poor outcomes, and monitor therapeutic handlings. A number of studies suggest that simultaneously with lactate, pyruvate should also be measured, since this might augment prognostic ability, and provide a better understanding of the underlying metabolic alterations in ICU patients. Hence, the aim of the present study was to elucidate the relationship between lactate levels, the lactate-to-puruvate (LP) ratio, and clinical outcome in mechanically ventilated COVID-19 patients.

**Methods:** Lactate and pyruvate were serially measured during the first 24 h of ICU stay. A group of ICU non-COVID-19 patients was additionally studied. The subgroup of COVID-19 patients presenting with elevated lactate was also analysed in an attempt to assess the contribution of hypoxic and non-hypoxic causes of hyperlactatemia.

**Results:** The majority of COVID-19 patients (82.5%) had normal lactate and LP ratio on ICU admission. A small, yet significant percentage of patients, who had either elevated lactate levels or a high LP ratio, had a significantly higher risk of ICU mortality (72.7% vs. 34.6%, *p* = 0.04). Finally, high lactate seemed to be related to hypoxic or non-hypoxic causes in COVID-19 patients.

**Conclusions:** In our critically ill COVID-19 patients, hyperlactatemia was not only due to tissue hypoperfusion. Furthermore, elevated lactate levels or high LP ratios on admission to the ICU could be associated with poor clinical outcome.

## P045

### Body mass index and lactate levels on admission predict ICU mortality in elderly critically ill with severe COVID-19 pneumonia

#### H Božič, M Kurnik, A Vindišar, P Kolar, M Podbregar, CE COVID-19 Study Group

##### General Hospital Celje, Department of Internal Intensive Medicine, Celje, Slovenia

*Critical Care* 2022, **26(Suppl 1):** P045

**Introduction:** Obesity paradox is an established phenomenon regarding overweight patients treated in ICUs. Mortality of especially elderly patients with COVID-19 pneumonia is high and there is still scarcity of definitive predictors. Aim of our study was to assess the prediction value of Body Mass Index (BMI) on mortality of elderly critically ill with severe COVID-19 pneumonia.

**Methods:** Data of patients, older than 70 years, with severe COVID-19 pneumonia, admitted to 22-bed mixed ICU, level 3, was analysed retrospectively. We especially focused on BMI and lactate levels at admission.

**Results:** A total of 102 patients (average age 77 ± 5 years) were included. Average length of ICU stay was 11.4 ± 9.2 days. Average BMI was 29.3 ± 5.2 kg/m^2^ and lactate levels 2.9 ± 3.2 mmol/l (Table 1). High-flow oxygenation, non-invasive ventilation and invasive ventilation were at some point used to support 34/102, 36/102 and 69/102 patients respectively. ICU mortality was 51/102 (50.0%). ICU stay was shorter in survivors (9.1 ± 8.5 vs 13.6 ± 9.4 days, *p* = 0.01). BMI was higher in survivors (30.5 ± 5.6 vs. 28.1 ± 4.5 kg/m^2^, *p* = 0.02); 26/51 (51%) survivors and only 15/51 (29%) non-survivors had BMI ≥ 30 kg/m^2^ (chi-square *p* = 0.043). At admission BMI (OR 0.9153, 95%CI 0.8610–0.9730, *p* = 0.0045) and lactate levels (OR 1.3492, 95%CI 1.0856–1.676, *p* = 0.0069) were independent predictors of ICU mortality.

**Conclusions:** BMI and lactate levels at admission are independent predictors of ICU mortality of elderly patients with severe COVID-19 pneumonia. The study confirmed the obesity paradox in elderly critically ill with severe COVID-19 pneumonia.

**Table 1 (abstract P045):** Results

Variable	All (n = 102)	ICU survivors (n = 51)	ICU non-survivors (n = 51)	*p* Value
BMI, kg/m^2^	29.3±5.2	30.5±5.6	28.1±4.5	0.020
Lactate at admission, mmol/l	2.9±3.2	2.1±1.6	3.7±4.2	0.012
**Logistic regression models**	**OR**	**95%CI**	***p* Value**	
BMI - univariate	0.9151	0.8623-0.9711	0.01	
Lactate - univariate	1.3033	1.0666-1.5926	0.0096	
BMI - multivariate	0.9153	0.8610-0.9730	0.0045	
Lactate - multivariate	1.3492	1.0856-1.6768	0.0069	

BMI: Body mass index, CI: Confidence interval, ICU: Intensive care unit, OR: Odds ratio

## P046

### The effect of premorbid metformin use on lactate kinetics, kidney injury and mortality in patients with sepsis and septic shock: an observational study

#### N Van Moorter^1^, T Tackaert^2^, N De Neve^3^, B Van Vlem^4^, K De Decker^3^

##### ^1^Ghent University Hospital, Internal Medicine, Ghent, Belgium, ^2^Ghent University Hospital, Emergency Medicine, Ghent, Belgium, ^3^OLVZ Aalst, Anaesthesiology and Critical Care Medicine, Aalst, Belgium, ^4^OLVZ Aalst, Nephrology, Aalst, Belgium

*Critical Care* 2022, **26(Suppl 1):** P046

**Introduction:** Sepsis and septic shock cause significant mortality worldwide, with no targeted molecular therapies available. Metformin has pleomorphic effects that may be beneficial in sepsis, but at present, the impact of metformin exposure in sepsis remains controversial. Metformin might alter lactate metabolism, but little is known about its impact on lactate kinetics. We therefore investigated the impact of preadmission metformin use on lactate kinetics, acute kidney injury (AKI) and mortality in sepsis and septic shock.

**Methods:** We conducted a retrospective cohort study in patients with sepsis and septic shock admitted to the ICU between January 2013 and September 2020, including 77 users and 390 non-users (subdivided in diabetics, n = 48 and non-diabetics, n = 342). AKI staging was based on the AKIN criteria including urinary output data. Chronic haemodialysis patients were excluded.

**Results:** Groups and subgroups did not differ in severity of illness nor sepsis aetiology. Admission lactate levels were not different, but evolution in lactate over the first 24 h showed a larger decrease in users vs nonusers (median − 53% vs − 36%, *p* = 0.010). No difference in AKI or need for renal replacement therapy was found. Mortality data showed lower mortality in users vs nonusers in case of septic shock (21.9% (n = 7) vs 42.7% (n = 61) for 90-d mortality, *p* = 0.029, OR 0.38 [95% CI: 0.15–0.93]) (see Table 1), but revealed no significant differences in the general sepsis population.

**Conclusions:** In our data, preadmission metformin use is associated with a significantly larger decrease in lactate after admission in sepsis or septic shock and with reduced mortality in septic shock. This underscores the need for further studies investigating the interplay between metformin, lactate and sepsis, thereby exploring the potential of metformin use in the treatment of sepsis.

**Table 1 (abstract P046):** Survival in patients with septic shock according to metformin use.

	Users	Nonusers		*p* Value (users vs all non-users)
		Diabetics	Nondiabetics	
ICU mortality, n (%)	3 (9.4%)	2 (13.3%)	40 (31.3%)	0.019
30-day mortality, n (%)	6 (18.8%)	5 (33.3%)	44 (34.4%)	0.087
90-day mortality, n (%)	7 (21.9%)	5 (33.3%)	56 (43.8%)	0.029
1-year mortality, n (%)	10 (31.3%)	7 (46.7%)	66 (51.6%)	0.043

## P047

### Body temperature and blood lactate production in coronary artery bypass grafting patients

#### D Lončar-Stojiljković^1^, MP Stojiljkovic^2^

##### ^1^Institute for Cardiovascular Diseases Dedinje, Anesthesia and Intensive Care, Belgrade, Serbia, ^2^Faculty of Medicine, University of Banja Luka, Department of Pharmacology, Toxicology and Clinical Pharmacology, Banja Luka, Bosnia and Herzegovina

*Critical Care* 2022, **26(Suppl 1):** P047

**Introduction:** Production of blood lactate during and after cardiopulmonary bypass (CABG) are associated with tissue hypoperfusion during hypothermia. The lungs were found to be a significant source of lactate, and this pulmonary lactate flux was accentuated by CPB and hypotermia.

Objective of this study was to determine an association between blood and pulmonary lactate levels after CPB and temperature in first four postoperative hours in isolated coronary artery bypass grafting (CABG).

**Methods:** This was prospective observational study in 20 patients, ASA III classification, scheduled for elective CABG operation (one to four bypasses). Lactate concentration was measured using a lactate analyser in simultaneously drawn arterial (A) and mixed venous (V) blood samples. At the 60-min interval four measurements per patient were taken and the temperature and the haemodynamic parameters were measured (heart rate, mean arterial pressure -MAP, pCO_2_ difference) at the same time. Concomitantly, measurements of cardiac output were also performed. Pulmonary lactate release was calculated as the product of transpulmonary A-V lactate and cardiac index. Statistical analysis was performed by one-way ANOVA with *post* *hoc* analysis employing two-tailed t-test with Bonferroni correction.

**Results:** The mean cardiopulmonary bypass duration was 89.43 ± (6.69) min, and the aortic cross-clamping time was 63.33 ± 20.73 min. The ejection fraction (EF) was 42.12 ± 11.72. There were no significant differences among the haemodynamic parameters. Temperature values were rising across the four hour study period. The lactate values from venous and arterial blood, as well as their differences decrease significantly over the time (Table 1).

**Conclusions:** The pulmonary lactate production during and after CABG inversely correlated with the increase in body temperature.

**Table 1 (abstract P047):** Postoperative lactate level

	1 h	2h	3h	4h
Temp.	35.18 ± 0.70	35.67 ±0.56*	36.02 ± 0.4*	36.46 ± 0.59*
Venous	2.65 ± 1.13	2.17 ± 1.08	1.62 ± 0.78*	1.48 ± 0.61*
Arterial	2.31 ± 1.02	1.76 ± 0.99	1.5 ± 0.81	1.40 ± 0.59*
Lact(diff)	0.34 ± 0.11	0.41 ± 0.09	0.12 ± 0.04*	0.08 ± 0.02*

*p < 0.05 vs 1h, ¶*p* < 0.05 vs 2h

## P048

### The role of anion gap normalization time in the management of pediatric diabetic ketoacidosis. is it time for a new perspective?

#### I Lazar^1^, D Orlov^2^, G Hazan^1^, A Orbach^3^, A Hayim^4^, Y Cavari^2^, Y Feinstein^2^, E Neeman^2^, E Hershkovitz^4^, Y Faingelernt^1^

##### ^1^Soroka Medical Center and the Faculty of Health Sciences, Ben Gurion University of the Negev, Pediatrics, Beer Sheva, Israel, ^2^Soroka Medical Center and the Faculty of Health Sciences, Ben Gurion University of the Negev, Pediatric Intensive Care Unit, Beer Sheva, Israel, ^3^Soroka Medical Center, Pediatric Intensive Care Unit, Beer Sheva, Israel, ^4^Soroka Medical Center and the Faculty of Health Sciences, Ben Gurion University of the Negev, Pediatric Endocrinology, Beer Sheva, Israel

*Critical Care* 2022, **26(Suppl 1):** P048

**Introduction:** Diabetic ketoacidosis (DKA) is a life-threatening metabolic crisis. Pediatric DKA criteria include hyperglycemia (serum glucose > 200 mg/dl), acidosis (pH < 7.2) and\or serum bicarbonate < 15 mEq/l. DKA resolution criteria (normalization of PH, bicarbonate and anion gap) are somewhat confusing because anion gap and clinical conditions improve and normalize hours before PH and bicarbonate do. This may prolong PICU admission and carry unnecessary treatments, laboratory tests and medical expenses. We hypothesized that anion gap normalization time (AGNT) correlates with the severity of DKA in children admitted to the PICU and could be a good marker for DKA resolution.

**Methods:** We extracted data from medical charts of all PICU DKA admissions over ten years.

**Results:** Ninety-five patients met inclusion and exclusion criteria. Admission age was 10 ± 4.64 years. In 87% it was their first DKA episode. Mean admission serum glucose and pH were 508 mg/dl and 7.12 respectively. Median AGNT was 8 h. Late AGNT correlated with pH < 7.1 (OR 2.94 95% CI 1.2–7.4 *p* = 0.020) and serum glucose > 500 (OR = 3.31 95%CI 1.42–7.74, *p* = 0.005). Multivariate analysis showed glucose > 500 mg/dl increases the risk for delayed AGNT, by 3.41 folds and admission glucose elevation by each 25 mg/dl was associated with 10% risk increment for delayed AGNT. Median AGNT preceded median PICU discharge by 15 h (8 h vs. 23 h respectively).

**Conclusions:** AGNT could be used for DKA recovery assessment and late AGNT correlated with markers of its severity. AGNT represents return to normal physiology—stopping ketone body (KB) production, and KB and other organic acids clearance from the serum by restoration of intravascular volume and improvement of liver, kidneys and skeletal muscle perfusion. Acidosis beyond AGNT is caused by hyperchloremia and not by DKA. Adding AGNT to the DKA resolution criteria could have shorten PICU admission time. We suggest DKA resolution criteria in children be re-evaluated and AGNT role should be further studies.

## P049

### The management of profound hyponatraemia

#### A Baker, S Lobaz, P Duncan

##### Barnsley Hospital NHS Foundation Trust, Barnsley, UK

*Critical Care* 2022, **26(Suppl 1):** P049

**Introduction:** Barnsley Hospital uses the European Society of Intensive Care Medicine (ESICM) guidelines to manage profound hyponatraemia [1], but adverse outcomes have still been reported. A service evaluation was performed to determine current practice.

**Methods:** A retrospective study was undertaken. 30 inpatients with a serum sodium concentration of < 110 mmol/l were identified between 01/01/2018 and 07/06/2020. Data from ICE™ pathology reporting system and patient notes were collected then analysed for ESICM guideline compliance.

**Results:** Patients (aged 28–85 yrs) demonstrated a variety of signs and symptoms, with 27% asymptomatic at initial presentation. 83% of cases were identified in acute care specialities. Endocrinology and Critical Care were involved in 10% and 40% of cases respectively. 7 patients (23%) were admitted to Critical Care. Mean initial serum sodium concentration was 106.5 mmol/l. Frequency of blood sampling ranged between 1 and 11 tests in the first 48 h. Correction in the first 24 h ranged from 1 to 21 mmol/l. Correction at 24–48 h ranged from 1 to 12 mmol/l. 2.7% hypertonic saline was given in 27% of cases, all authorised by a senior clinician. With or without hypertonic saline, the rate of serum sodium correction exceeded ESICM limits in 47% cases. Overcorrection concerns were documented in one case. 11 out of 14 cases (79%) received no management for overcorrection. One patient developed osmotic demyelination syndrome. 6 of the 30 patients died during the index admission.

**Conclusions:** High over-correction rates highlight the importance of close monitoring, prompt intervention and a low threshold for Critical Care involvement. Trust guidelines require urgent reform with tighter daily sodium correction rates and management algorithms publicised. Staff awareness and education is key. Re-evaluation is planned for 6 months after guideline modification.

**Reference**

1. Spasovski G et al. Eur J Endocrinol 170:G1-G47, 2014.

## P050

### Hypercalcemia after admission to intensive care unit is associated with mortality

#### Y Hayashi, N Iguchi, A Tanaka, A Uchiyama, Y Fujino

##### Osaka University Graduate School of Medicine, Department of Anesthesiology and Intensive Care Medicine, Suita-City, Japan

*Critical Care* 2022, **26(Suppl 1):** P050

**Introduction:** The purpose of this study is to evaluate the association between the development of hypercalcemia and the patient outcomes.

**Methods:** This single-center retrospective cohort study included patients aged ≥ 15 years admitted to the intensive care unit (ICU) of Osaka University Hospital between January 1, 2016, and December 31, 2018 (approval number: 20258). The hypercalcemia group comprised patients with ≥ 11.0 mg/dl serum calcium and/or albumin corrected calcium levels occurring even once in the ICU, while others were defined as the normal calcium group. All information were retrieved from the institutional electronic medical records. The primary outcome was ICU mortality. Data of the outcome variables and baseline characteristics of each group were compared using the Mann–Whitney U-test or Fisher’s exact test. We used a propensity score (PS) matching by fitting a logistic regression model that was adjusted for age, sex, and the acute physiology and chronic health evaluation II score. We also compared hypercalcemia patients who died and survived in the ICU. All *p* values were 2-sided, and the level of significance was set at 0.05.

**Results:** A total number of 2410 patients were investigated; of whom, 147 (6.1%) patients had hypercalcemia. The hypercalcemia group had a higher rate of ICU mortality than that of the normal calcium group (25.2% vs. 1.2%, *p* < 0.001) even after PS matching (25.2% vs. 4.8%, *p* < 0.001). The hypercalcemia group had higher rates of ICU morbidity and hospital mortality (*p* < 0.001 for all) (Table). The patients of the hypercalcemia group who died in the ICU were found to have higher maximum calcium levels (12.4 [12.2–12.6] vs 11.4 [11.2–12.0] mg/dl, *p* < 0.001), and the highest calcium level was observed on a day later than that for patients who survived (37.0 [23.5–66.0] vs 9.0 [2.0–18.0] days, *p* < 0.001) (Table 1).

**Conclusions:** Hypercalcemia occurred in 6.1% of the ICU patients, and it was significantly associated with ICU mortality.

**Table 1 (abstract P050):** Patient outcomes

	Hypercalcemia group N = 147	Normal calcium group N = 2263	*p*
ICU mortality	37 (25.2%)	28 (1.2%)	< 0.001
Hospital mortality	58 (39.5%)	74 (3.3%)	< 0.001
Tracheostomy	62 (42.2%)	98 (4.3%)	< 0.001
Mechanical circulation support	36 (24.5%)	143 (6.3%)	< 0.001
Renal replacement therapy	79 (53.7%)	140 (6.2%)	< 0.001
Propensity score matching ICU mortality	37/147 (25.2%)	7/147 (4.8%)	< 0.001
Propensity score matching hospital mortality	58/147 (39.5%)	14/147 (9.5%)	< 0.001

## P051

### A hospital-wide quality improvement project to enhance patient handover between the emergency department and hospital wards

#### M Vreven^1^, H Vanden Eede^1^, J Bergs^2^

##### ^1^AZ Rivierenland, Department of Anaesthesiology and Intensive Care, Rumst, Belgium, ^2^Hasselt University, Faculty of Medicine and Life Sciences, Hasselt, Belgium

*Critical Care* 2022, **26(Suppl 1):** P051

**Introduction:** Consecutive incident reports pertaining to patient handovers between our emergency department (ED) and inpatient wards prompted a quality improvement project using the ADaPT® method. This led to a series of interventions aimed at improving handovers: a structured form within the electronic record; continued advocacy of the ISBAR structure; bedside handover; education; points of contact; and creating a positive environment to speak-up. Bedside handover was considered the most prominent change from current practice. Our study aimed to evaluate the effect of this project.

**Methods:** To evaluate the quality of patient handovers scoring tools were utilized, the *Hasselt* *Instrument* *for* *Assessing* *Handover* *Quality* (HIAHQ) and the *Handoff* *Clinical* *Evaluation* *Exercise* (handoff-CEX). The HIAHQ scores overall quality of patient handovers, whereas the handoff-CEX scores individual handovers. Both scoring tools were utilized before and after the implementation of abovementioned interventions. The HIAHQ was issued to all nurses concerned with patient handovers from the ED to inpatient wards. The handoff-CEX was made electronically available to both the ED and the ward nurse after a handover took place during two fortnight study periods.

**Results:** A total of 191 HIAHQ assessments were collected, respectively 109 pre-intervention and 82 post-intervention. A total of 194 individual handover interactions were scored with the handoff-CEX. Data from the HIAHQ showed a significant improvement in the overall perceived quality of patient handovers both for the ED as well as the ward nurses. The assessment of individual handovers showed a significant improvement as well on all domains of the handoff-CEX tool. This effect was most outspoken for the ward nurses. Figure 1 shows a graphical representation of the abovementioned results.

**Conclusions:** The ADaPT® method, providing a tailor-made improvement program enabled a project team to significantly improve the quality of nursing handovers between the ED and inpatient wards.

**Fig. 1 (abstract P051):**
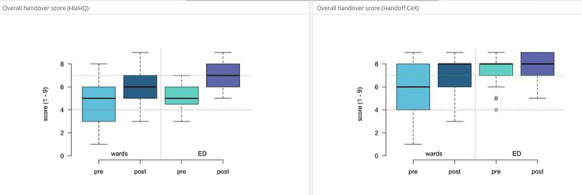
Pre- and post-intervention scores of HIAHQ and handoff CEX

## P052

### Elderly patients in the emergency department—a challenging job for emergency providers

#### A Mellouli, R Ghzel, Z Albatta, B Sakouhi, M Merchaoui

##### Ibn Jazzar University Hospital, Units of the Aghlabids, Emergency Department, Kairouan, Tunisia

*Critical Care* 2022, **26(Suppl 1):** P052

**Introduction:** Taking care of the elderly might be real challenge especially in the emergency department (ED). The noticed increase in lifespan during the last decades made it the job of health care providers to ensure a good quality of care provided for these subjects. However, in areas of low resources where geriatric departments are still lacking, managing this fragile category of the population might be an even harder job to do. The goal of our study was to establish the epidemiological characteristics, as well as to draw the pathological profile of these patients.

**Methods:** This a prospective single-center study conducted in the ED of the Aghlabids Hospital, over a two-month period, from March 2020 till April 2020. We used SPSS22.0 for analytical analysis.

**Results:** We enrolled 276 patients. There was a slight male predominance (56.88%, n = 157). Mean age was at 72 years ranging from 65 years till 97 years. One third of the patients presented at least one comorbidity. A number of 186 patients presented a Daily Activity living (ADL) scale > 3 which means 67.4% of the patients were not self-dependent. In our study, 76.81% (n = 212) of the patients were performed additional medical investigations. Urinary tract pathologies were most common (39%, n = 107) followed by locomotor system pathologies (20%, n = 55), and surgical pathologies (14.5%, n = 40). Only 3.5% (n = 10) of the patients were admitted to the resus room and only 1.25% (n = 3) of the patients needed urgent neurosurgical intervention. In our study, 56% of the patients were discharged to go home, while 44% of the patients were admitted to departments other than the ED.

**Conclusions:** This study drew the particular and distinguished aspect of elderly patients who visit the emergency department. Trying to provide health care for such patients is challenging and requires a preestablished protocols. A geriatric department could also be a good idea since such departments are still lucking in areas of low resources such as Kairouan.

## P053

### Reduction of emergency CT diagnostic in suspected pulmonary embolism—an age- and risk-adapted protocol

#### L Averhoff^1^, D Dürschmied^2^, K Müller-Peltzer^3^, CN Lang^4^, HJ Busch^1^, B Schmid^1^

##### ^1^Medical Center – University of Freiburg, Department of Emergency Medicine, Freiburg im Br., Germany, ^2^Medical Center Mannheim, University of Heidelberg, Department of Medicine I, Mannheim, Germany, ^3^Medical Center – University of Freiburg, Department of Radiology, Freiburg im Br., Germany, ^4^Medical Center – University of Freiburg, Department of Medicine III (Interdisciplinary Medical Intensive Care), Freiburg im Br., Germany

*Critical Care* 2022, **26(Suppl 1):** P053

**Introduction:** Patients with symptoms of pulmonary embolism (PE) are common in the emergency department (ED). Positive D-dimers (DD) often trigger emergency computer tomography (CT). With this study we aimed to address the dilemma between overuse of CT scans and fear of missed PE. To rule out PE, grant a high grade of security for the patients, reduce radiation, costs, and occupied beds, we evaluated a combination of age- and risk-adapted protocol.

**Methods:** We performed a monocentric, retrospective study on patients tested for DD in order to diagnose PE between 01/2018 and 08/2018. We analyzed patients with DD between 0.5–1.0 mg/l, using age-adjustment (recommended by the guidelines) and risk-adaption with the Well’s Score. In patients with DD ≤ 1 mg/l and the Well’s Score of ≤ 4 diagnosis of PE deemed to be unlikely. The CT scans of these low-risk patients were re-read in detail.

**Results:** Data on DD were raised in 1,270 patients in the examined period. We excluded 1,022 patients as DD tests were carried out for other reasons than PE, or DD values were below 0.5 mg/l or over 1.0 mg/l. 148 patients were included. PE was unlikely in 60 patients using age-adjustment for DD. PE was likely in 88 patients, in 19 patients a CT scan was not performed and had to be excluded. Only 7 out of 69 patients had a Well’s score over 4 in the risk-adaption analysis. Of these 7 patients, 1 patient had a paracentral PE. Out of 62 low-risk patients, 2 patients had a subsegmental PE, 1 patient a known thrombus. This results in a reduction of 89.8% in scans in the analyzed cohort, missing 2 new minor PEs (3.2%). In the group who would have had a scan 1 out of 7 patients (14.3%) was diagnosed with a paracentral PE.

**Conclusions:** The combination of age- and risk-adapted assessment in patients with suspected PE resulted in a convincing reduction of CT scans with a tolerable risk for missing PE.

## P054

### Routine D-dimer assessment for the prediction of pulmonary embolism in patients with COVID-19 pneumonia in the ICU

#### J Louwsma^1^, B Langeveld^2^, J Luyendijk^3^, H van den Oever^1^

##### ^1^Deventer Hospital, Intensive Care Unit, Deventer, Netherlands, ^2^Deventer Hospital, Department of Pulmonology, Deventer, Netherlands, ^3^Deventer Hospital, Department of Radiology, Deventer, Netherlands

*Critical Care* 2022, **26(Suppl 1):** P054

**Introduction:** Pulmonary embolism (PE) is a common complication of the coronavirus disease 2019 (COVID-19) and is associated with an increased mortality risk in hospitalized patients [1]. We conducted this study to assess the specificity of an elevated absolute D-dimer for PE in COVID-19 patients admitted to the ICU, as well as the specificity of a D-dimer increment in COVID-19 patients who developed PE during ICU-stay.

**Methods:** We conducted a case–control study in patients with COVID-19 pneumonia admitted to our ICU. D-dimer values were paired with results of CT pulmonary angiograms (CTPA), and compared in patients with and without PE on admission. In patients without PE on initial imaging and available follow-up CTPA during ICU-stay, the D-dimer increment between initial and follow-up imaging was calculated. Patients that developed PE during ICU-stay were compared with those with persistently no PE.

**Results:** Of 124 patients, CTPA on admission was performed in 100; PE was diagnosed in 22. D-dimer values were elevated in both groups, but more in those with proven PE (median 6060, IQR 1105–16,600 vs 850, IQR 492–1570 μg/l; *p* < 0.0001). Using the standard cut-off value of 500 μg/l, specificity was 27.3%, which increased to 100.0% when an adjusted cut-off value of 9000 μg/l was used. In 38 patients without PE on initial CTPA, follow-up imaging was obtained during ICU-stay; PE was diagnosed in 21, increasing the overall incidence of PE in COVID-19 patients in our ICU to 34.7%. Patients that developed PE during ICU-stay showed a higher D-dimer increase than those who remained PE negative (median 8890 IQR 5640–19,000 vs 4970 IQR 1970–7803 μg/l; *p* < 0.01). Using a cut-off value of > 8000 μg/l for delta D-dimer, specificity was 100% in our study population.

**Conclusions:** When adjusted cut-off values are used, routine measurement of D-dimer might aid clinicians in diagnosing pulmonary embolism in patients with COVID-19 pneumonia admitted to the ICU.

**Reference**

1. Van den Berg et al. J Crit Care 64:18–21, 2021

## P055

### Epidemiology and clinical characteristics of COVID-19 patients admitted to the emergency department

#### A Sghaier^1^, I Dlala^2^, N Chebbi^2^, A Belkacem^2^, S Bettout^2^, N Jerbi^2^, S Marghli^2^

##### ^1^Taher Sfar Hospital, Mahida, Tunisia, ^2^Taher Sfar Hospital, Emergency Department, Mahida, Tunisia

*Critical Care* 2022, **26(Suppl 1):** P055

**Introduction:** The COVID-19 epidemic has been developing in Tunisia officially since March 2, 2020. Since the spread of the epidemic, the governorate of Mahdia has experienced 23,271 cases of contamination including 673 deaths and 20,739 cases of recovery. The aim of this study is to describe the epidemiological and clinical characteristics of patients with COVID-19 who have been hospitalized in the emergency department of Mahdia.

**Methods:** This is a retrospective study, carried out in the Emergency Department of Mahdia over a period of 12 months extending from September 2020 until August 2021. We included patients over 18 years-old hospitalized with COVID-19 lung disease confirmed either by RT-PCR or rapid antigen testing. For all patients included, demographic, clinical, biological and therapeutic data were collected as well as the patient's outcome.

**Results:** A total of 976 patients were included, the mean age was 67. Patients over 70 years of age accounted for 40%. The sex ratio 1.06. 74.2% of patients had at least one comorbidity, including hypertension (52%) and diabetes (46%). The most frequent clinical sign was dyspnea (77%). Initial management included low flow oxygen therapy in 319 patients (33%), high flow oxygen therapy in 426 patients (44%). And 102 patients (10%) received non-invasive ventilation and 13 (1%) received invasive ventilation. Severe forms are frequent (43% of cases). Patients admitted to the emergency department only (360 or 37%), compared to those transferred to intensive care (209, 21%) and to the COVID department (373, 38%), were older [age average respectively: 73 years (SD ± 13); 66 (SD ± 13); 61 (SD ± 13); *p* = 0.001] and likely to have at least one comorbidity (79%, 76%, 73% respectively). The case fatality rate was 31.5% in all patients.

**Conclusions:** In this study, our patients were mainly elderly, male, having at least one comorbidity with a high mortality rate, hence the need for a prospective study specifying the predictive factors of mortality.

## P056

### Descriptive epidemiology of COVID-19 death cases reported at the emergency department Taher Sfar Mahdia

#### A Sghaier, N Jerbi, M Ben Amira, A Marsit, N Farhat, S Bettout, I Dlala, S Marghli

##### Taher Sfar Hospital, Emergency Department, Mahida, Tunisia

*Critical Care* 2022, **26(Suppl 1):** P056

**Introduction:** The Coronavirus disease 2019 (COVID-19) is a pandemic responsible for 3 million deaths worldwide according to the World Health Organization (WHO) hence considered as a " global health emergency”. The aim of the present study was to describe the demographic characteristics, clinical presentation, of COVID-19 death cases in the emergency department Taher Sfar Mahdia.

**Methods:** We did a retrospective study over a period of 1 year, from August 2020 to August 2021, carried out at Taher Sfar Mahdia hospital, in the Emergency Department, on patients over 18 years old with COVID 19 confirmed by either RT-PCR and or SARS-COV-2 rapid antigen test and dead in emergency room.

**Results:** A total of 976 cases were detected over the study period including 308 (29,9%) COVID-19 deaths among them dead in emergency department. The median age of those patients was 76 years [IQR 69–83]. The sex ratio (M / W) was 1.3. 57 (56%) patients had at least one comorbidity mainly arterial hypertension (57%) followed by diabetes (38%). The median time from symptoms onset to patient’s admission was 5 days [IQR 4–10], they had a median SpO_2_ of 81% [IQR 75%-88. While their hospitalization, these patients required high flows of O_2_, mainly HCM: 148 (48%); NIV 49 (15%) and MV 101 (33%). The median length of stay was 4 days [IQR 2–7].

**Conclusions:** This descriptive retrospective study shows also a high mortality rate in the emergency department. These deaths could be avoided if the intensive care department accepted them, but the advanced age, several comorbidities, a severe clinical form were the reasons why the medical resuscitation department refused to take them in charge preferring to save the few places they have for patients with better prognosis.

## P057

### Psychological impact of COVID-19 pandemic assessed among emergency department consultants

#### A Jlidi, S Borji, W Farhat, Y Walha, EM Ben Othmane, A Abri, D Hamdi, N Nouira

##### Mongi Slim Academic Hospital, Emergency Department, La Mara, Tunisia

*Critical Care* 2022, **26(Suppl 1):** P057

**Introduction:** The COVID-19 pandemic, declared in March 2020, had a significant impact on the well-being of the general population around the world. The quarantine, isolation, restriction of activities and the economic crisis were at the origin of these mental health disorders. The aim of our study was to assess the COVID-19 pandemic psychological impact.

**Methods:** This was a cross-sectional prospective that lasted one month starting from October 1, 2021. we interviewed the emergency department consultants and their companions. The Form included 23 items assessing the anxious and depressive symptoms by a score validated by the High Authority for Health.

**Results:** We included 190 adults, the average age was 30 ± 10 years with a sex-ratio of 1.5, the majority (78%) were of university education, 17% had a secondary level of education. In our sample, 14.7% had a pathological history, 12.1% were on symptomatic treatment for psychiatric disorders, 10% were already followed by a psychiatrist. Half of the population had a COVID-19 infection, 77.4% had a parent or family member who was infected with COVID-19. Concerning the psychological impact 48.9% had signs of definite depression and 50.5% had signs of anxiety. A significantly higher level of anxiety in people who have had a member of the family infected with COVID-19 ( OR 2.99, IC 95% [1.44; 6.19]; *p* < 0.01).

**Conclusions:** In this context of COVID-19 pandemic the psychological impact is almost inevitable and the fear of infecting and losing a loved one remains the most dreadful event.

## P058

### Neutrophil-to-lymphocyte ratio on admission to predict mortality of COVID-19 patients admitted to the emergency department

#### A Sghaier, I Dlala, N Chebbi, F Ben Salem, N Farhat, S Bettout, N Jerbi, S Marghli

##### Taher Sfar Hospital, Emergency Department, Mahida, Tunisia

*Critical Care* 2022, **26(Suppl 1):** P058

**Introduction:** COVID-19 is a pandemic declared in 2020, the gravity of which lies in the risk of developing a cascade of complications in 20% of cases, which can lead to death. These complications result from an inadequate immuno-inflammatory reaction. Several factors are predictive of severe forms and death. Taking into account these predictive markers will allow the classification of patients with COVID-19 and their optimal management. The aim of our study was to investigate the value of neutrophil–lymphocyte ratio (NLR) in predicting mortality of COVID-19 patients hospitalized in the emergency department of Mahdia.

**Methods:** A retrospective cohort study including patients over 18 years old hospitalized in the emergency department with COVID 19 confirmed by either RT-PCR and or SARS-COV-2 Rapid Antigen Test, over a period of 9 months from September 2020 to May 2021. The parameters collected were: demographic, clinical, biological characteristics as well as patient outcome and hospital mortality. The NLR was calculated for all patients.

**Results:** A total of 454 patients were included, the mean age of the patients was 67 years (SD ± 14), the sex ratio (M/W) was 1.11. In-hospital mortality was 34% (155 patients). On-admission NLR levels were significantly higher in the non-survivor group 14.4 (SD ± 15.0) compared to the survivor group 9.7 (SD ± 9.6) (*p* = 0.001). Although NLR differed significantly between the survivor and non-survivor groups, this ratio was not identified as a predictor of mortality in COVID-19 lung disease as the informational index study showed low sensitivity and specificity.

**Conclusions:** Our study showed that on-admission neutrophil–lymphocyte ratio (NLR) was statistically higher in non-survivors patients, however we did not find a cut-off value that could discriminate between surviving and non-surviving patients with acceptable statistical significance.

## P059

### Comparison of a cellular host response test to common sepsis indicators in a suspected infection population presenting to the emergency department (ED)

#### H O’Neal^1^, R Sheybani^2^, T Caffery^3^, H Tse^2^, A Shah^2^, C Thomas^1^

##### ^1^LSU Health Sciences Center / Our Lady of the Lake Regional Medical Center, Pulmonary & Critical Care Medicine, Baton Rouge, LA, USA, ^2^Cytovale, LLC, Cytovale, San Francisco, CA, USA, ^3^LSU Health Sciences Center / Our Lady of the Lake Regional Medical Center, Emergency Medicine, Baton Rouge, LA, USA

*Critical Care* 2022, **26(Suppl 1):** P059

**Introduction:** Sepsis, a common and fast-moving condition, often arises in the community, and presents to the ED where providers must perform the initial diagnosis for patients with signs of infection. Currently, a rapid diagnostic with clinically actionable performance is needed to assist ED clinicians in this task. The objective of this study was to assess the potential of the IntelliSep test when compared to commonly-assessed indicators of sepsis.

**Methods:** The IntelliSep test is an investigational in-vitro diagnostic that quantifies the state of immune activation by measuring the biophysical properties of leukocytes from a routine blood sample in under 10 min. The test provides a single score, the IntelliSep Index (ISI), between 0.1–10.0 (inclusive), stratified into three discrete interpretation bands of risk for disease severity: Green, Yellow, and Red. Adult patients presenting to the ED with signs or suspicion of infection were prospectively enrolled at multiple sites in the USA (Feb. 2016–Sept. 2019). EDTA-anticoagulated blood was assayed within 3 h of draw, and patients were followed by retrospective chart review. Treating clinicians did not have access to assay results. All other measurements were taken per standard of care. Retrospective physician adjudication determined sepsis status (Sepsis-3).

**Results:** For the 549 subjects (sepsis prevalence 20%) included in the final analysis, the ISI achieved an AUC of 0.88 (0.84–0.91, 95% CI) with a sensitivity of 87.4 (78.7– 92.2, 95% CI) and specificity of 91.3 (84.1–95.6, 95% CI) in the Green and Red interpretation bands, respectively. Table 1 includes a comparison of the ISI with a selection of other commonly-assessed indicators of sepsis. Lactate values were available for 414 subjects.

**Conclusions:** The ISI, a quantitative measure of immune activation, compared favorably to common indicators as an aid in the rapid assessment of sepsis risk for patients presenting to the ED with signs of infection.

**Table 1 (abstract P059):** Comparison of the ISI to common biomarkers and scoring systems for sepsis

	AUC (95% CI)	Negative predictive value (95% CI)	Positive predictive value (95% CI)	Diagnostic odds ratio (95% CI)
2+ SIRS	0.56 (0.53–0.58)	91.6 (84.1–95.6)	22.3 (14.4–30.4)	3.12 (0.6–7.7)
qSOFA (≥ 2)	0.63 (0.57–0.68)	82.8 (73.6–88.6)	41.8 (32.2–51.2)	3.45 (0.6–7.7)
WBC ( < 4 or > 12 ×103 cells/µl)	0.61 (0.57–0.65)	89.7 (81.9–94.3)	25.6 (17.5–34.4)	3.0 (0.6–7.7)
Lactate (low < 2; high ≥ 4)	0.72 (0.67–0.77)	84.5 (72.1–91.4)	52.8 (38.9–64.0)	6.1 (1.7–14.8)
APACHE II (≥ 15)	0.64 (0.59–0.69	83.6 (74.6–89.4)	28.2 (19.8–37.2)	2.0 (0.2–6.4)
SOFA (≥ 2 up to 24 hours following presentation)	0.74 (0.69–0.78)	93.3 (86.3–96.8)	28.5 (19.8–37.2)	5.56 (2.0–11.4)
IntelliSep Index (low Green band; high Red band)	0.88 (0.84–0.91)	95.7 (88.1–98.7)	64.5 (52.6–74.1)	40.2 (29.2–51.1)

## P060

### Prediction of safe discharge of emergency department patients with suspected acute infection using a 29-mRNA host response test

#### E Diehl-Wiesenecker^1^, N Galtung^1^, F Uhle^2^, O Liesenfeld^2^, W Bauer^1^

##### ^1^Charité-University Medicine, Emergency Department, Berlin, Germany, ^2^Inflammatix, Burlingame, USA

*Critical Care* 2022, **26(Suppl 1):** P060

**Introduction:** Unnecessary hospital admission causes increased resource utilization and may harm patients. Therefore, in patients presenting to the emergency department (ED) with suspected infections, the early and reliable identification of those without need for hospitalization is crucial and remains an unmet medical need. We evaluated the 29-host-response-mRNA severity classifier IMX-SEV-2, already validated for identifying critically ill patients, for safe discharge dispositions.

**Methods:** A total of 312 adult patients presenting to a tertiary care ED with suspected acute infection or sepsis were prospectively enrolled [1]. Expression of 29-host mRNAs was measured and interpreted with IMX-SEV-2 from whole blood to determine low, moderate or high risk categories. Results were compared with the clinically adjudicated requirement for hospital-level care based on chart review.

**Results:** Among 312 patients, 22 patients (7.1%) died in hospital and 56 (17.9%) experienced multi organ failure (MOF). 22 (7.1%) patients were adjudicated as not requiring hospital care. For predicting safe discharge, IMX-SEV-2 had the highest area under the receiver operating characteristic (AUROC) of 0.81 (95% CI 0.76–0.93) (Fig. 1). Of patients with Confusion, Respiratory Rate, Blood Pressure, Age > 65 [2] (CRB-65) scores < 1 in combination with IMX-SEV-2 low-risk, only one patient (0.3%) had MOF and one (0.3%) died compared to 9 (2.9%) patients with MOF and 3 (1%) deaths when only applying CRB-65.

**Conclusions:** IMX-SEV-2 outperforms established risk prediction systems and biomarkers in identifying low-risk patients with suspected infections or sepsis in the ED. Combined with a clinical score like CRB-65, IMX-SEV-2 enhances severity prediction and could support in patient management.

**References**

1. Bauer W et al. Crit Care Med 49:1664–1673,2021
2. Bauer TT et al. J Intern Med 260:93–101, 2006

**Fig. 1 (abstract P060):**
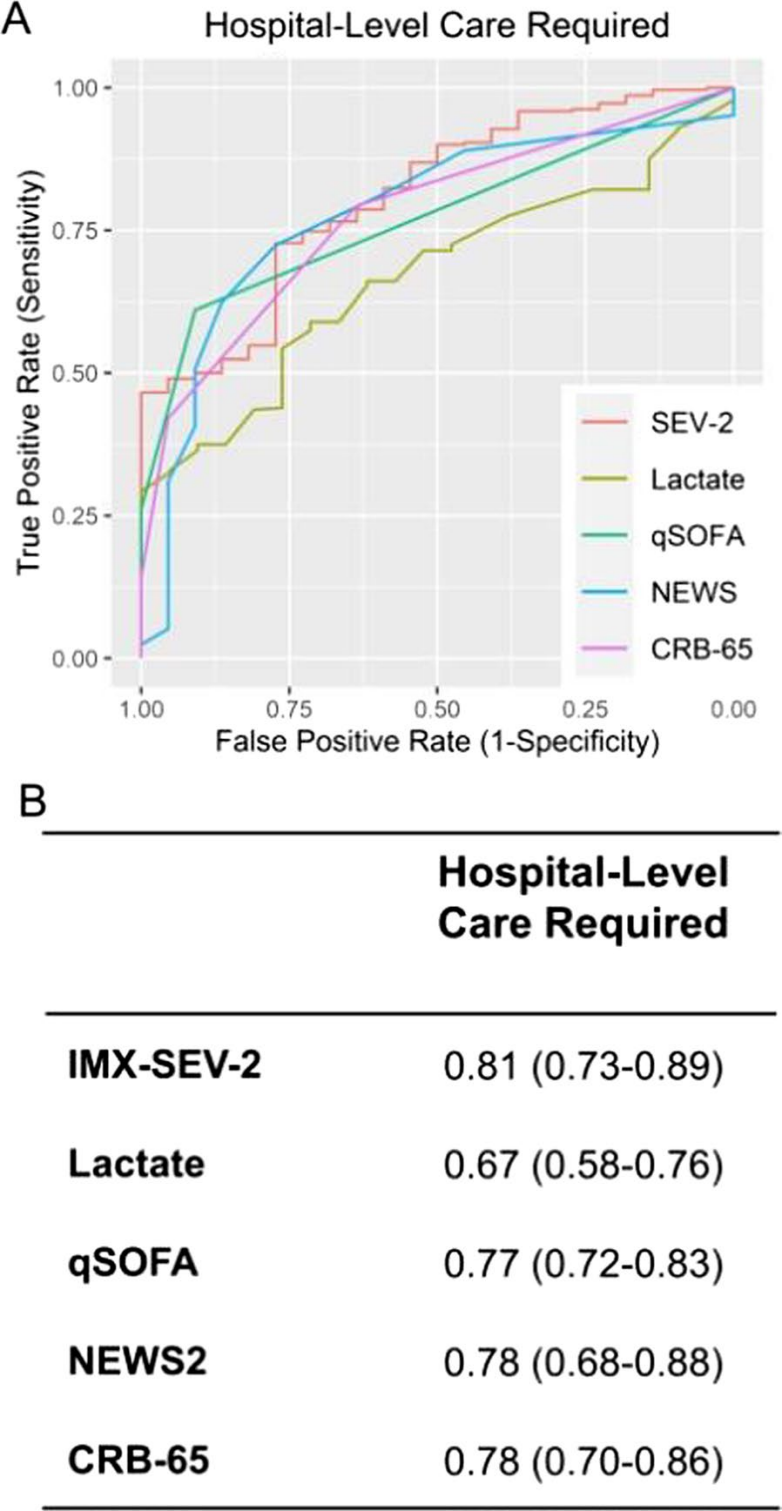
AUROCs of IMX-SEV-2 and comparators for predicting the requirement of hospital-level care (AUROC = area under the receiver operating characteristic, IMX-SEV-2 = Inflammatix Severity Classifier Version 2, qSOFA = quick Sequential Organ Failure Assessment, NEWS2 = National Early Warning Score 2, CRB-65 = Confusion, Respiratory Rate, Blood Pressure, Age > 65-Score)

## P061

### A novel transcriptomic host response classifier accurately predicts bacterial infections and 30-day mortality among critically ill surgical patients

#### L Moldawer^1^, UI Chen^2^, R Bacher^3^, L Zhong^1^, T Loftus^1^, P Starostik^4^, O Liesenfeld^5^, U Midic^2^, T Sweeney^2^, S Brakenridge^1^

##### ^1^University of Florida College of Medicine, Sepsis and Critical Illness Research Center, Department of Surgery, Gainesville, FL, USA, ^2^Inflammatix Inc, Burlingame, USA, ^3^University of Florida College of Medicine, Gainesville, FL, USA, ^4^University of Florida College of Medicine, Department of Pathology, Gainesville, FL, USA, ^5^Inflammatix Inc, Clinical Affairs, Burlingame, USA

*Critical Care* 2022, **26(Suppl 1):** P061

**Introduction:** Accurate identification of sepsis and prediction of its severity remains a clinical challenge. A 29-mRNA-host response classifier, IMX-BVN-3/SEV-3 (BVN/SEV-3) demonstrated excellent performance in identifying bacterial and viral infections and 30-day mortality in emergency department patients. Here we investigated the predictive performance of BVN/SEV-3 in surgical ICU patients.

**Methods:** Patients were prospectively enrolled at ICU admission with either suspected sepsis (cohort A; n = 52) or considered at-risk for sepsis (cohort B; n = 137); 11 patients initially enrolled in Cohort B subsequently suspected of sepsis and were moved to an additional crossover cohort. BVN/SEV-3 was measured from whole blood sample (via NanoString nCounter) at cohort enrollment. Likelihoods of bacterial and viral infection and illness severity were determined by BVN/SEV-3 and its accuracy was compared with procalcitonin, IL-6, and sequential organ failure assessment (SOFA) scores.

**Results:** Among 200 patients (median age 62.5 years) 30-day mortality was 3.6%, 15.4% and 18.2% in cohort A, B and crossover, respectively. Overall, BVN/SEV-3 had an area under the receiver operator curve (AUROC) of 0.84 [0.78–0.90] for diagnosing bacterial infection at time of enrollment, similar to procalcitonin (0.85 [0.80–0.91]) but significantly better than IL-6 (0.67 [0.59–0.76], *p* < 0.001). BVN/SEV-3 predicted 30-day mortality with an AUROC of 0.81 [0.66–0.95], significantly better than IL-6 (0.57 [0.37–0.77], *p* = 0.006), marginally better than procalcitonin (0.65 [0.50–0.79], *p* = 0.056) and similar to SOFA (0.76 [0.62–0.91]). In patients with SOFA ≥ 6, BVN-3/SEV-3 high, moderate and low severity scores stratified mortality incidences to 36%, 6%, and 0%.

**Conclusions:** BVN-3/SEV-3 accurately predicts infections and 30-day mortality in surgical ICU patients. With implementation as a rapid point of care test, BVN-3/SEV-3 could guide clinical care and improve resource utilization in critically ill surgical patients.

## P062

### Extracellular plasma DNA levels limit the predictive value of genetic TLR9 variant rs352162 in multimorbidity sepsis patients

#### V Pisarev^1^, A Chumachenko^2^, E Grigoriev^3^, E Ershova^4^, S Kostyuk^4^, D Shlykova^2^

##### ^1^Federal Research and Clinical Center of Intensive Care Medicine and Rehabilitology, V.A.Negovsky Institute of General Reanimatology, Moscow, Russian Federation, ^2^Federal Research and Clinical Center of Intensive Care Medicine and Rehabilitology, Moscow, Russian Federation, ^3^O.M. Filatov’ City Clinical Hospital, Moscow, Russian Federation, ^4^Research Centre for Medical Genetics, Moscow, Russian Federation

*Critical Care* 2022, **26(Suppl 1):** P062

**Introduction:** Extracellular DNA (exDNA) fragments in plasma mediate intercellular signaling in critical illness contributing to systemic inflammation reactions. Most studied mechanism includes binding of exDNA to its receptor TLR9 to induce NFkB gene expression. Carriers of genetic variant CC of TLR9 rs352162 are predisposed to multiple organ failure and/or lethal outcome in post-trauma[1], post-stroke [2] and sepsis[3]. In a novel study, we assess whether the concentration of exDNA in plasma may limit or reveal the predictive value of rs352162 TLR9 CC genotype in multimorbidity sepsis patients.

**Methods:** The cohort included 110 post-surgery and diabetes patients with sepsis and multimorbidities (70% with cardiovascular diseases, hypertension, coronary artery diseases), Charlson index value, 7 (4–9), CIRS scale value, 23 (17–27). Circulating exDNA was isolated from plasma and quantified by CYBR Green dye. Genotyping of allelic variants CC, CT and TT of the TLR9 rs352162 polymorphism was performed using a PCR and allele-specific tetra primer set followed by electrophoretic separation of the PCR products. Statistical analysis was performed using SigmaPlot 12.5 software. The Shapiro–Wilk test was used to assess the normality of variable distribution in the groups. Significance between groups was evaluated by Chi-square with Yates correction, Fisher’s test, Mann–Whitney test and One-was ANOVA.

**Results:** Carriers of TLR9 CC rs352162 genotype exhibit significantly increased CIRS index for lower GI and hepatic/pancreatic comorbidities. Only subcohort of patients with exDNA > 100 ng/ml, however, exhibit significant association of TLR9 CC rs352162 genotype and increased risk for unfavorable outcome (odds ratio 6.4, 95%CI: 1.338–30.6) (Fig. 1), whereas no linking of TLR9 genotype and outcome are revealed in patients with < 100 ng/ml exDNA in plasma.

**Conclusions:** Determining the polymorphism of TLR gene and exDNA concentration in plasma might aid in stratification of multimorbid sepsis patients in ICU for early personalized intensive care.

**Fig. 1 (abstract P062):**
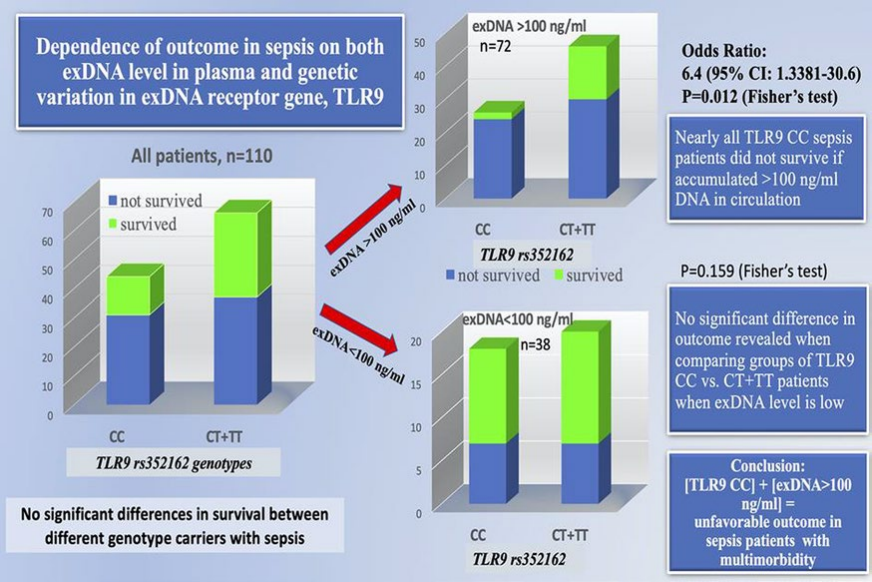
Increased concentrations of plasma exDNA is required to reveal linking TLR9 genetic polymorphism and outcome in sepsis

## P063

### Usefulness and cost-effectiveness of procalcitonin in critical care patients admitted to the East Sussex Healthcare NHS Trust (ESHT)

#### C Cabaret^1^, J Evans^1^, J Perrins^2^

##### ^1^East Sussex NHS Healthcare Trust, Anaesthetics and Critical Care Medicine, Eastbourne, UK, ^2^East Sussex NHS Healthcare Trust, Pharmacy, Eastbourne, UK

*Critical Care* 2022, **26(Suppl 1):** P063

**Introduction:** This study evaluated the usefulness and cost-effectiveness of procalcitonin (PCT) following its introduction to aid antibiotics discontinuation in critical care patients admitted to ESHT during the COVID-19 pandemic.

**Methods:** Non-surgical critical care patients with a diagnosis of sepsis or lower respiratory tract infection during their admission between 01st January and 30th June 2020. Retrospective analysis of data using ICCA (IntelliSpace Critical Care and Anaesthesia) to compare the number of antibiotic doses administered per patient before and after introduction of PCT. After PCT introduction, we recorded the number of PCT levels requested, their frequency as well as the level of PCT and when discontinuation occurred.

**Results:** A total of 81 patients were included—13 admitted before PCT introduction and 68 after (this important increase in the number of patients is explained by the increased proportion of patients with COVID-19 pneumonitis). The average dose of antibiotics administered per patient was reduced by 28.8% (70.24 vs 49.98) following introduction of PCT. Despite an incurred cost of £12 per PCT assay, the overall average cost per patient was reduced by £59.60 (£257.94 vs £146.78). A lack of consistency in the frequency of PCT level request was observed.

**Conclusions:** Introduction of PCT to aid discontinuation of antibiotics resulted in a 28.8% reduction in average antibiotics prescription and an overall cost reduction of £59.60 per patient. The reduction in antibacterial exposure also brings non-financial benefits such as increased patient safety through experience of less side-effects, reduction in antibiotics resistance among others. The lack of consistency in the requests of PCT resulted in the design of a protocol for its use within ESHT.

## P064

### Early prediction of sepsis in intensive care patients using a machine learning algorithm, a randomized clinical validation trial

#### F Sjövall^1^, I Persson^2^

##### ^1^Carl Bertil Laurells Gata 9, Intensive Care Unit, Malmö, Sweden, ^2^Department of Statistics, Department of Statistics, Uppsala, Sweden

*Critical Care* 2022, **26(Suppl 1):** P064

**Introduction:** The primary objective of this trial was to, in an ICU setting, externally validate the prognostic accuracy of a machine learning sepsis prediction algorithm that uses routinely collected vital parameters, blood gas values and lab values as input. An improved detection of sepsis will enable earlier treatment that may influence outcome. The tested algorithm has previously retrospectively demonstrated the ability to detect ICU patients at high risk of developing sepsis within the coming hours [1].

**Methods:** Adult patients admitted to the ICU at Skåne University Hospital Malmö from December 2020 to September 2021 were eligible for recruitment to the trial. A total of 305 subjects were randomized into one of two groups: Active algorithm, with sepsis alerts, or Standard of care. The algorithm made predictions in the whole cohort but was not displayed in the Standard of care group, in order to evaluate its performance without disturbing the outcome. The study was blinded, i.e. study personnel did not know for which subjects the algorithm was being used. The ICU followed standard practices in assessing possible development of sepsis and intervening accordingly. The subjects were followed in the study until ICU discharge.

**Results:** The algorithm could identify patients with high risk of developing sepsis, with high performance (accuracy 0.79; sensitivity 0.80; specificity 0.78) for predictions 3 h before sepsis onset.

**Conclusions:** The accuracy, sensitivity, and specificity were all high, validating the prognostic accuracy of the algorithm in an ICU setting. This is, as far as the authors are aware, the largest clinical trial ever conducted with a machine learning sepsis prediction algorithm, and the first one that clinically validates a sepsis prediction algorithm against the Sepsis-3 criteria.

**Reference**

1. Persson et al. JMIR Form Res 5:e28000, 2021.

## P065

### NEWS2 predicts severity of underlying inflammatory response and outcome in COVID-19 patients

#### MT Howard^1^, O Watson^2^, JC Zaldua^2^, S Pillai^2^, J Whitley^2^, M Lawrence^3^, K Hawkins^3^, O Guy^3^, PA Evans^2^

##### ^1^Welsh Centre for Emergency Medicine Research, Emergency Department, Swansea, UK, ^2^Welsh Centre for Emergency Medicine Research, Swansea, United Kingdom, ^3^Swansea University, Swansea, UK

*Critical Care* 2022, **26(Suppl 1):** P065

**Introduction:** COVID-19 is a severe respiratory disease associated with a marked inflammatory response. Clinical methods of assessing severity of disease, including National Early Warning Score 2 (NEWS2), have been shown to predict severity in COVID-19 [1]. However, little research has been undertaken comparing NEWS2 to underlying inflammatory processes. In this study, we assessed whether inflammatory markers taken at presentation to the Emergency Department could predict and mortality in COVID-19 patients.

**Methods:** Whole blood samples were taken at admission to the emergency department for procalcitonin, fibrinogen, CRP, von Willebrand Factor (vWF), IL-6 and TNFα. NEWS2 was also recorded on admission. Levels of inflammatory markers were retrospectively compared to NEWS2 scores and mortality outcomes.

**Results:** A total of 95 patients positive for COVID-19 were included. NEWS2 values > 5 were associated with higher CRP (131.5 ± 87.9 vs 86.4 ± 106.5, *p* = 0.03), IL-6 (71.9 ± 111 vs 43.4 ± 99, *p* = 0.007), and vWF (334.1 ± 83.3 vs 296.3 ± 93.4, *p* = 0.04). The trend of increasing inflammatory markers was also shown in patients who died, significantly so for IL-6 (44.4 ± 54.97 vs 18.8 ± 48.36, *p* = 0.035). NEWS2 was also shown to be significantly higher in patients who died (7.8 ± 2.2 vs 4.3 ± 2.8, *p* =  < 0.01).

**Conclusions:** NEWS2 predicted the severity of underlying inflammatory response. All inflammatory markers showed a marked increase with severity and mortality, most significant with IL-6. This suggests NEWS2 and inflammatory markers may predict severity and mortality in COVID-19 patients. Further research is required to evaluate these mechanistic changes in inflammatory response.

**Reference**

1. Kostakis I et al. Resuscitation. 159:150–157, 2021.

## P066

### Using the cardio-vascular index (CVRI) to predict mortality in septic shock

#### O Raphaeli^1^, I Bendavid^1^, C Hajaj^2^, L Statlander^1^, A Goldstein^2^, E Chen^2^, P Singer, U Gabbay

##### ^1^Beilinson Hospital, Intensive Care Unit, Petah Tikva, Israel, ^2^Ariel University, Industrial Engineering and Management, Ariel, Israel, ^3^Beilinson Hospital, Quality and Safety Department, Petah Tikva, Israel

*Critical Care* 2022, **26(Suppl 1):** P066

**Introduction:** The Cardio-Vascular Index (CVRI) is a multi-vital sign index suggesting promising association with diverse conditions and morbidities along the entire hemodynamic spectrum [1–4]. Our study aimed to evaluate CVRI predictability of death in septic shock.

**Methods:** Dataset included adult patients stayed at Beilinson hospital ICU for more than 48 h (2012–2018). This study uses a cohort of 2,122 patients. CVRI patterns along 48 h from ICU admission were computed. Five machine learning (ML) classification algorithms were trained and tested for the development of prediction model of ICU mortality (Python software). We compared models using admission conditions only with models adding CVRI. Prediction performance was assessed by the receiver operating characteristics area under the curve (AUC) of ten-fold cross-validation and validation sets. The study was authorized by the local IRB.

**Results:** In the cohort, the median (IQR) age was 63 (49–72) years, BMI 26 (23–31). Main admission conditions: surgical (40%), trauma (28%) and medical (21%). Septic shock was detected in 509 patients (24%). The best performing ML algorithm was Random Forest classifier. The model adding CVRI was preferable (AUC = 0.85) over model including admission conditions only (AUC = 0.77) (Fig. 1).

**Conclusions:** CVRI values of the first 48 h added to admission conditions were good predictors of death in septic shock. CVRI had an added value in predicting death over the existing admission conditions. This index may improve overall identification of high-risk patients.

**References**

1. Gabbay U et al. Med Hypotheses 82: 694–699, 2014
2. Gabbay U, et al. Clin Trials Regul Sci Cardiol 12:1–5, 2015
3. Gabbay U et-al. J Trauma Treat 8: 450, 2019
4. Schiffmann N et al. Clin J Sport Med 31.3: 232–236, 2021

**Fig. 1 (abstract P066):**
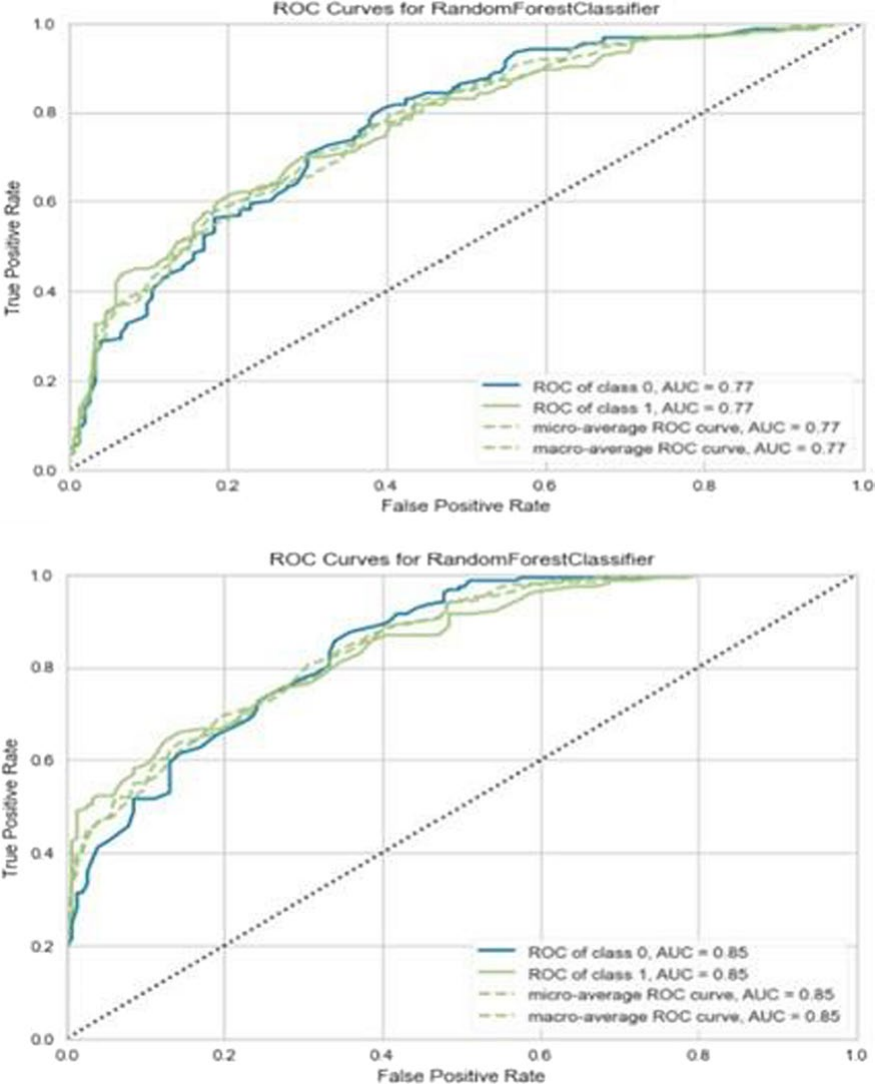
ROC Curves: admission conditions model (top) admission conditions & CVRI model (bottom)

## P067

### Relationship between the procalcitonin levels and clot microstructure in acute exacerbation of chronic obstructive pulmonary disease (AECOPD)

#### S Pillai^1^, M Lawrence^1^, JC Zaldua^1^, J Whitley^1^, O Watson^1^, M Howard^1^, K Harrison^2^, K Hawkins^3^, K Morris^4^, PA Evans^1^

##### ^1^Welsh Centre for Emergency Medicine Research, Emergency Department, Morriston Hospital, Swansea, UK, ^2^Morriston Hospital, Swansea, UK, ^3^Swansea University, Swansea, UK, ^4^Cardiff Metropolitan University, Cardiff, UK

*Critical Care* 2022, **26(Suppl 1):** P067

**Introduction:** Procalcitonin (PCT) is a widely used biomarker that helps to differentiate systemic inflammatory response syndrome (SIRS) from sepsis. PCT is increasingly used in emergency departments to differentiate between infective with non-infective exacerbation of COPD to guide antibiotic therapy [1]. D_*f*_ is a functional biomarker that quantifies the incipient blood clots fibrin network across sepsis spectrum [2]. The aim of the study was to compare the changes in clot microstructure at different levels of PCT fractal dimension-d_*f*_. Patients were divided into five groups based on their PCT levels.

**Methods:** A total of 85 AECOPD patients were recruited from the emergency department of a tertiary teaching hospital. Those patients who did not have PCT levels at admission were excluded. Blood samples were taken to perform fractal dimension (d_*f*_) and PCT. The five groups based on PCT levels and what these levels indicates were < 0.05 ng/ml (no SIRS), ≥ 0.05 to < 0.5 ng/ml (SIRS), ≥ 0.5 to < 2.0 ng/ml (sepsis), ≥ 2.0 to < 10 ng/ml (severe sepsis) and ≥ 10 ng/ml (septic shock).

**Results:** The d_*f*_ was highest at PCT level of ≥ 0.5 to < 2.0 ng/ml (Fig. 1). There was a significant correlation between d_*f*_ at PCT at this level (*p* = 0.04). The d_*f*_ was not significantly different between the five groups.

**Conclusions:** Our results indicate that COPD patients develop denser and tighter clot microstructure as indicated by high d_*f*_ when PCT levels are at a level consistent with sepsis. These changes in d_*f*_ were previously demonstrated across sepsis spectrum. This study demonstrates the utility of d_*f*_ to quantify clot microstructure across the sepsis spectrum in COPD patients particularly during exacerbations.

**References**

1. Pantzaris et al. J Clin Med Res 10:545–551, 2018
2. Davies et al. Intensive Care Med 42:1990–1998, 2016

**Fig. 1 (abstract P067):**
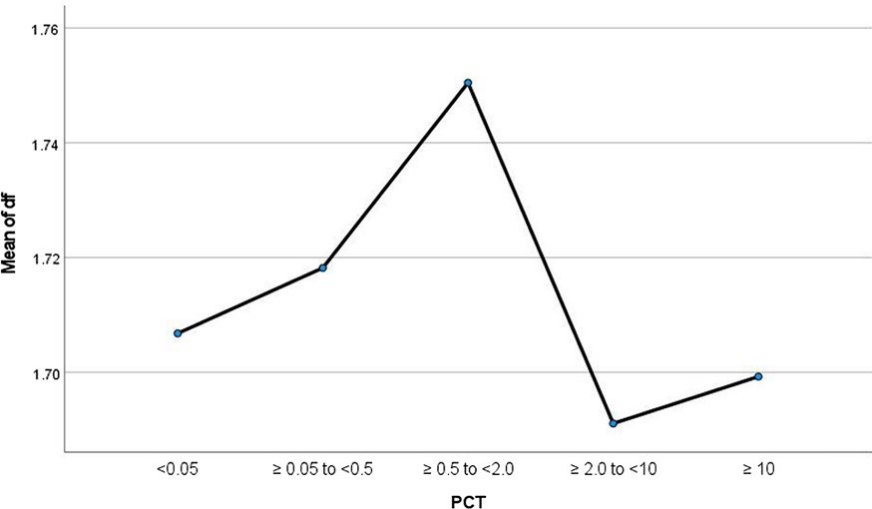
D_*f*_ at different levels of PCT in AECOPD patients

## P068

### Performance of ED and discharge diagnosis of sepsis against objective assessments underscores the need for a reliable diagnostic

#### H O’Neal^1^, R Sheybani^2^, T Caffery^3^, D Hamer^4^, D Burgin^4^, S Alwood^3^, T Jagneaux^1^, C O’Neal^5^, A Shah^2^, C Thomas^1^

##### ^1^LSU Health Sciences Center / Our Lady of the Lake Regional Medical Center, Pulmonary & Critical Care Medicine, Baton Rouge, LA, USA, ^2^Cytovale, LLC, Cytovale, San Francisco, CA, USA, ^3^LSU Health Sciences Center / Our Lady of the Lake Regional Medical Center, Emergency Medicine, Baton Rouge, LA, USA, ^4^LSU Health Sciences Center / Our Lady of the Lake Regional Medical Center, Internal Medicine, Baton Rouge, LA, USA, ^5^LSU Health Sciences Center / Our Lady of the Lake Regional Medical Center, Infectious Diseases, Baton Rouge, LA, USA

*Critical Care* 2022, **26(Suppl 1):** P068

**Introduction:** Despite increasing awareness of and consensus guidelines for the treatment of sepsis, there is no reference standard for its diagnosis. Clinical diagnosis at presentation is difficult, resulting in heterogeneity in outcome reporting and trial performance when these diagnoses are used as classifiers.

**Methods:** Adult patients presenting to the ED with signs or suspicion of infection were prospectively enrolled at multiple sites in the USA (Feb. 2016–Sept. 2019) in four discrete but similar cohorts. We compared different standards for sepsis diagnosis (ED Diagnosis, Discharge Diagnosis, and an Objective Assessment) with a reference standard of retrospective physician adjudication. ED diagnosis and Discharge Diagnosis were recorded from the medical record. Objective Assessment for sepsis was based on evidence of an infection defined by modified CDC surveillance definitions *and* an increase in SOFA of 2 or more points over baseline during any of the first three days of hospitalization.

**Results:** A total of 678 subjects were included in the final analysis with a sepsis prevalence of 17.8% by the adjudication standard. Table 1 includes a comparison of ED diagnosis, discharge diagnosis, and objective assessment of sepsis in predicting adjudicated sepsis. While ED and Discharge Diagnoses performed similarly in comparison to physician adjudication, Objective Assessment had a significantly higher diagnostic odds ratio than either. In general, treating clinicians are more likely to over-diagnose sepsis.

**Conclusions:** Treating clinicians are biased toward the over-diagnosis of sepsis, making ED and Discharge Diagnosis less reliable than an Objective Assessment, thus impacting outcome data and trial performance and underscoring the need for a reliable diagnostic biomarker for sepsis at ED presentation.

**Table 1 (abstract P068):** Comparison of included standards for sepsis diagnosis

	ED DIAGNOSIS	Discharge diagnosis	Objective assessment (3 days post presentation)
AUC (95% CI)	0.79 (0.74–0.83)	0.78 (0.74–0.82)	0.87 (0.83–0.90)
Positive percent agreement/sensitivity (95% CI)	65.3 (56.1–73.7)	63.6 (54.4–72.2)	86.0 (78.5–91.6)
Negative percent agreement/specificity (95% CI)	91.7 (85.3–96.0)	92.5 (85.3–96.0)	87.4 (79.4–92.2)
Diagnostic odds ratio (95% CI)	20.9 (13.8–29.0)	21.5 (13.8–29.0)	42.6 (33.2–51.5)

## P069

### Prospective evaluation of the PAWSS ability to detect alcohol withdrawal in ICU patients.

#### M Geslain, E Lucchese, X Chapalain, O Huet

##### CHRU Brest, Brest, France

*Critical Care* 2022, **26(Suppl 1):** P069

**Introduction:** Up to 20% of ICU patients present AUDs (Alcohol Use Disorders) in their medical history. An unplanned and brutal stop of alcohol consumption, as it can occur during ICU stay, may lead to an alcohol withdrawal syndrome (AWS). Prediction of Alcohol Withdrawal Severity Scale (PAWSS) has been designed to identify medical ward patients at risk of complicated AWS, with a threshold of 4 (Sensitivity 93%; Specificity 99%) [1]. In this study, we aim to test the accuracy of PAWSS to predict complicated AWS in ICU patients.

**Methods:** Prospective cohort of surgical ICU patients of a university-affiliated, community hospital in France. Patients eligible for a PAWSS assessment in the first 24H of their ICU stay were included. Patients were excluded if: hospital length of stay was greater than 5 days prior to ICU admission, patient under sedation, impaired neurological state, patients with AWS at admission in ICU. During their ICU stay, alcohol withdrawal signs were daily assessed by clinicians using the Clinical Institute Withdrawal Assessment-Alcohol, Revised (CIWA-Ar). Primary outcome was the occurrence of a mild or sever AWS within 5 days.

**Results:** A total of 618 patients were enrolled in the study. 202 patients didn’t have any alcohol consumption within the last 30 days. Among the 416 patients with alcohol consumption within the past 30 days, 61 patients had a PAWSS ≥ 4 (14.6%). For a cut-off of 4, PAWSS sensitivity for detecting a severe AWS (CIWA > 15) is 91% and specificity is 90%, and for detecting a mild AWS (CIWA > 8), sensitivity is 80% and specificity 91%.

**Conclusions:** PAWSS is an excellent tool to predict severe AWS in ICU patients (Fig. 1). A cut-off of 4 is able to detect most of patients who will suffer from a severe AWS.

**Reference**

1. Maldonado JR et al. Alcohol Alcohol 50:509–18, 2015

**Fig. 1 (abstract P069):**
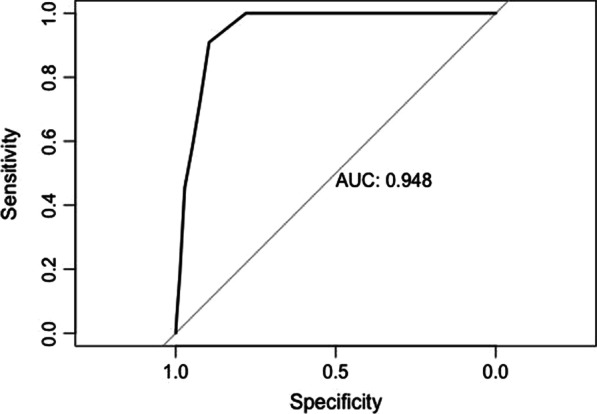
Receiver operating characteristic analysis for PAWSS and severe AWS

## P070

### Perioperative red blood cell transfusion in cytoreductive surgery with hyperthermic intraperitoneal chemotherapy

#### GA Madrid^1^, E Celis^2^, OA Ballesteros^2^, S Pabón^1^, EE Rodríguez^2^

##### ^1^Fundación Santa Fe de Bogotá, Anesthesiology Department, Bogotá, Colombia, ^2^Fundación Santa Fe de Bogotá, Intensive Care Department, Bogotá, Colombia

*Critical Care* 2022, **26(Suppl 1):** P070

**Introduction:** Cytoreductive surgery with hyperthermic intraperitoneal chemotherapy (CRS/HIPEC) is a complex procedure that may require transfusion of a large volume of red blood cells (RBC). Previously, large transfusions have been associated with worse outcomes [1]. We aimed to describe the need for RBC transfusion in patients undergoing CRS/HIPEC at a university hospital in Bogotá, Colombia, during the perioperative period and to explore possible associations with clinical outcomes.

**Methods:** A retrospective cohort study was conducted, including patients who underwent CRS/HIPEC at a university hospital from 2007 to 2018. We excluded patients with blood dyscrasia, active infection, and surgical injury (vessel or solid organ). We recorded the number of RBC units transfused in the perioperative period and clinical outcomes as ICU and hospital stay, days on mechanical ventilation, ICU readmission, days to oral intake and ambulation. We used descriptive statistics according to variable distribution. Exploratory analyses were performed using ANOVA, chi-square, or Fisher exact test as appropriate.

**Results:** A sample of 130 patients was analyzed. No transfusion was needed in 12% of patients, 50% required less than 4 units, and 38% received at least 4 units. Patients who required transfusion had a lower mean hemoglobin than those who were not transfused (single-factor ANOVA *p* < 0.01). BMI < 18 and bleeding > 1500 ml were associated with transfusion of ≥ 4 units of RBC (*p* 0.016 and *p* < 0.01, respectively). BMI in the overweight range was associated with no transfusions (*p* 0.016). Exploratory analysis showed that the ICU stay was longer in patients transfused (*p* < 0.01) and more days of ventilation were needed when ≥ 4 units were transfused (*p* 0.036).

**Conclusions:** Most patients programed for CRS/HIPEC will need transfusion. Risk factors in our study are underweight, heavy bleeding, and inferior preoperative hemoglobin.

**Reference**

1. Fisher OM et al. Eur J Surg Oncol 45:2412–2423, 2019.

## P071

### Viscoelastic hemostatic assays and coagulopathy in patients with sepsis

#### TH Yeoh, PY Ng

##### Queen Mary Hospital, Adult Intensive Care Unit, Hong Kong, Hong Kong, SAR China

*Critical Care* 2022, **26(Suppl 1):** P071

**Introduction:** Conventional laboratory coagulation tests based on plasma samples are routinely used to assess for coagulopathy. However, point of care viscoelastic hemostatic assays (VHAs) have been used increasingly in recent years. This study aimed to examine the utilization of VHAs in characterizing the hemostatic pattern of patients with sepsis admitted to the ICU.

**Methods:** This was a single-centre, prospective observational study including patients with sepsis admitted to the Adult Intensive Care Unit between December 2020 and July 2021. Simultaneous blood samples were taken for thromboelastography (TEG®), rotational thromboelastometry (ROTEM®) together with conventional laboratory tests, including platelet count, prothrombin time, activated partial thromboplastin time, and fibrinogen. Data from VHAs were compared with conventional laboratory tests with Spearman’s correlation coefficient.

**Results:** A total of 75 patients were recruited. The median (IQR) age was 67 (57–78) years, and 42 (56.0%) were male. Upon enrolment, they had a mean APACHE IV score of 96 ± 33. Up to 25 (33.3%) patients had documented bacteremia. The median time to results availability was 77.4 min (60.3–107.3) for platelet, 106.0 min (84.2–167.8) for clotting time, and 120.9 min (88.8–179.6) for fibrinogen. It took a median time of 5 min (5–10) for ROTEM® and 10 min (10–10) for TEG® to achieve interpretable results. Assessment for hyperfibrinolysis took a mean time of 60.0 ± 0.0 min for ROTEM® and 50.6 ± 5.8 min for TEG®. There were significant positive correlations between platelet and CRT A10 (correlation coefficient, r = 0.86), and between fibrinogen and CFF A10 (r = 0.77) of TEG® (Fig. 1). ROTEM®showed similar findings, with EXTEM A5 correlating well with platelet (r = 0.90), and FIBTEM A5 with fibrinogen (r = 0.81). The correlation between VHAs and clotting time were less optimal.

**Conclusions:** There was good correlation between VHAs and platelet and fibrinogen levels in patients with sepsis, with a much shorter turnaround time.

**Fig. 1 (abstract P071):**
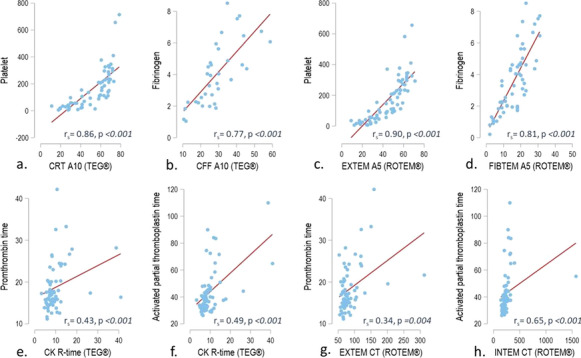
Scatter plots of platelet and fibrinogen with TEG® and ROTEM® parameters showing good correlation (graphs a-d); prothrombin time (PT) and activated partial thromboplastin time (APTT) with TEG® and ROTEM® parameters showing less optimal correlation (graphs e-h).

## P072

### Clot microstructure (d_*f*_) as a biomarker and measurement of thrombogenicity in acute exacerbation of chronic obstructive pulmonary disease (AECOPD)

#### S Pillai^1^, JC Zaldua^1^, M Lawrence^1^, J Whitley^1^, M Howard^1^, O Watson^1^, K Harrison^2^, K Hawkins^3^, K Morris^4^, PA Evans^1^

##### ^1^Welsh Centre for Emergency Medicine Research, Emergency Department, Morriston Hospital, Swansea, UK, ^2^Morriston Hospital, Swansea, UK, ^3^Swansea University, Swansea, UK, ^4^Cardiff Metropolitan University, Cardiff, UK

*Critical Care* 2022, **26(Suppl 1):** P072

**Introduction:** COPD is a chronic inflammatory condition that leads to lung parenchymal changes and microvascular damage. There is an increased incidence of venous thromboembolisms and in particular pulmonary embolism during exacerbations (15–30%). The aim of the study was to investigate whether there is a difference in clot microstructure between stable (SCOPD) and acute exacerbations of COPD (AECOPD) using the functional biomarker fractal dimension (d_*f*_) [1].

**Methods:** A total of 30 ambulatory SCOPD patients were recruited from the chest clinic and 85 AECOPD were recruited from the emergency department of a tertiary teaching hospital. Blood samples were taken to perform fractal dimension (d_*f*_), full blood count (FBC), platelet aggregometry, PT, aPTT, fibrinogen, D-dimer, procalcitonin (PCT), CRP and Factor XIII.

**Results:** The mean d_*f*_ in stable COPD patients was 1.69 ± 0.05 when compared to 1.71 ± 0.06 (*p* = 0.02). The time to gel point (T_GP_) that indicates the time for the initiation of blood clot was significantly lower in AECOPD group (327 ± 88 vs 275 ± 73, *p* = 0.004). Inflammatory markers such as CRP, PCT, WBC and Neutrophils was significantly higher in AECOPD group. D-dimer was significantly higher in AECOPD group however, FXIII was significantly lower in AECOPD group. FBC and platelet aggregometry was not statistically significant between the two groups (Fig. 1).

**Conclusions:** COPD patients during exacerbations develop a profound inflammatory response which is associated with tighter and denser clot microstructure as indicated by significantly increased d_*f*_. We have previously reported similar findings in sepsis. Therefore, d_*f*_ could be used to assess the underlying inflammatory effects of COPD on patient’s clotting profile and future anticoagulant therapy.

**Reference**

1. Davies et al. Intensive Care Med 42:1990–1998, 2016

**Fig. 1 (abstract P072):**
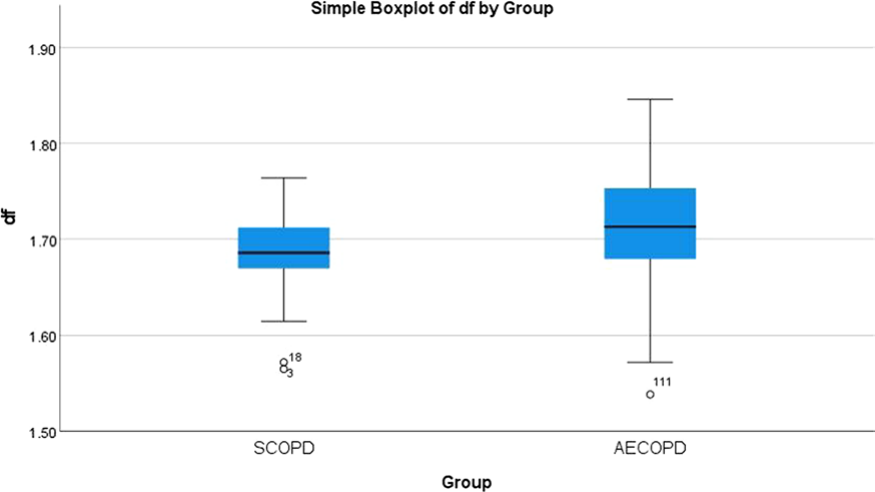
D_*f*_ is significantly increased in AECOPD group compared to SCOPD group

## P073

### The FiiRST-2 prospective, randomized study of clotting factor concentrates versus standard massive hemorrhage protocol in severely bleeding trauma patients

#### L Da luz^1^, J Callum^2^, A Beckett^3^, H Peng^4^, P Engels^5^, N Parry^6^, H Tien^7^, A Nathens^7^, B Schwartz^8^, K Karkouti^9^

##### ^1^Sunnybrook Health Sciences Centre, Department of Surgery, Toronto, Canada, ^2^Kingston Health Sciences Centre, Kingston, Canada, ^3^Saint Michael’s Hospital, Toronto, Canada, ^4^Defence Research and Development Canada, Toronto Research Center, Toronto, Canada, ^5^Hamilton General Hospital, Hamilton, Canada, ^6^London Health Sciences Centre, London, Canada, ^7^Sunnybrook Health Sciences Centre, Toronto, Canada, ^8^Octapharma, Paramus, USA, ^9^University Health Network, Sinai Health System, and Women’s College Hospital, Department of Anesthesia and Pain Management, Toronto, Canada

*Critical Care* 2022, **26(Suppl 1):** P073

**Introduction:** The FiiRST-2 study will investigate whether fibrinogen concentrate (FC) and prothrombin complex concentrate (PCC) given ≤ 1 h after hospital arrival is superior to the standard of care in bleeding trauma patients. Bleeding coupled with acute trauma coagulopathy (ATC) is a leading cause of in-hospital mortality in trauma. Acquired fibrinogen deficiency and impaired thrombin generation are major drivers of ATC. Prompt and targeted coagulation factor replacement with FC and PCC may be superior to the current standard of care, a ratio-based plasma resuscitation via a massive hemorrhage protocol (MHP).

**Methods:** FiiRST-2 is a randomized, parallel-control, superiority trial with an adaptive two-stage design, performed in 11 Canadian Level One Trauma Centers. Bleeding trauma patients > 16 years old (N = 350) will receive FC + PCC or a minimum 2:1 red blood cells (RBCs):plasma transfusion plus platelets, until the second MHP pack has been given, MHP is terminated, or 24 h has elapsed from admission (Fig. 1). Exclusion criteria include receipt of > 2 units RBCs before randomization, > 3 h elapsed from injury, catastrophic brain injury, or known bleeding disorders. The primary endpoint is superiority in the number of composite allogeneic blood product units transfused ≤ 24 h after admission. Secondary endpoints include RBC units transfused ≤ 24 h after admission, ventilator-free days, and 28-day mortality. Adverse and serious adverse events, including thromboembolic complications, will be assessed through 28 days.

**Results:** FiiRST-2 has enrolled 60 patients at 4 sites to date. An interim analysis will be performed after 120 patients have completed the study. Completion is expected in Q1 2023.

**Conclusions:** The FiiRST-2 study will determine if early use of factor concentrates (FC + PCC) is superior to the standard of care in bleeding trauma patients. Results could have a major impact on clinical practice, improving management and outcomes for this high-risk patient population.

**Fig. 1 (abstract P073):**
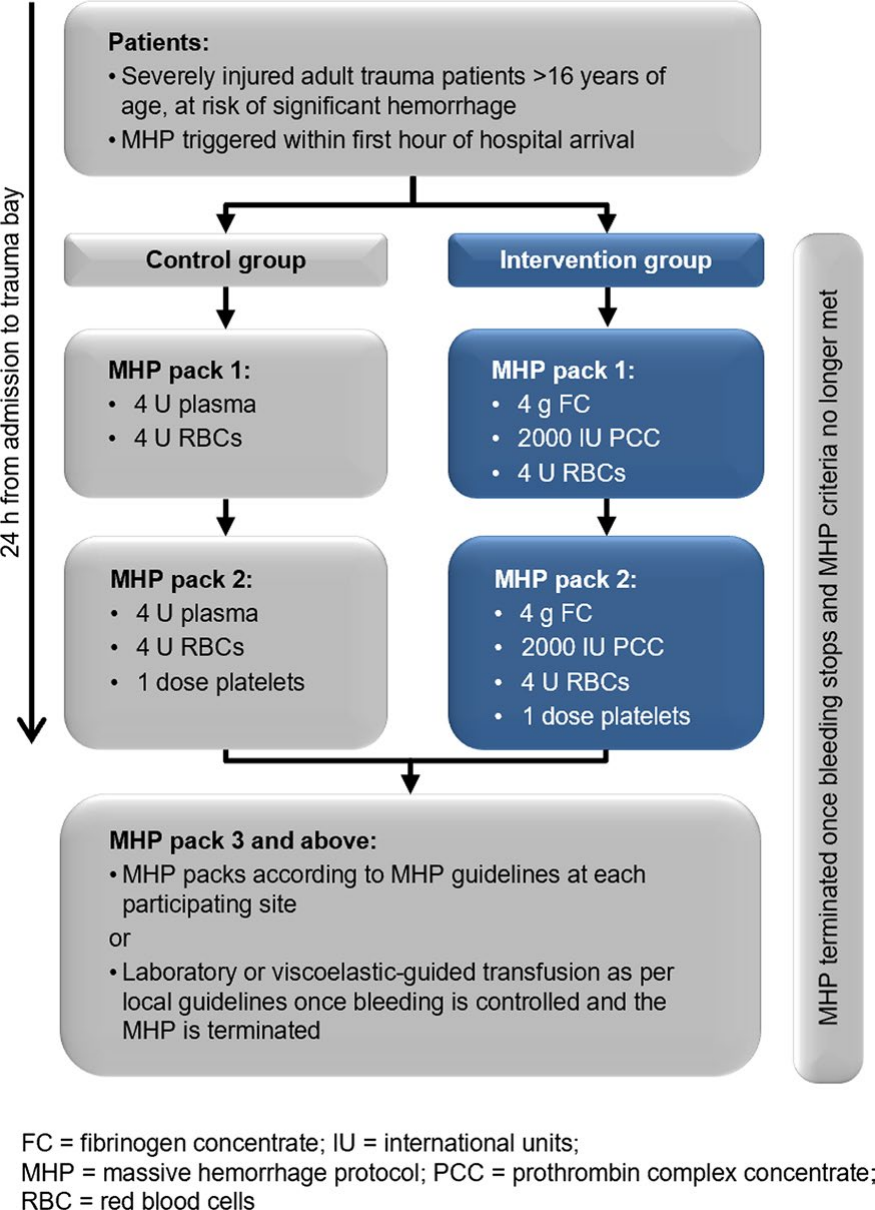
Study treatment plan

## P074

### Weight-adjusted dosing of fibrinogen concentrate and cryoprecipitate in the treatment of hypofibrinogenemic bleeding adult cardiac surgical patients: a post hoc analysis of the FIBRES randomized controlled trial

#### J Bartoszko^1^, C Devine^2^, J Callum^3^, K Karkouti^1^

##### ^1^University Health Network, Sinai Health System, Women’s College Hospital, University of Toronto, Department of Anesthesia and Pain Management, Toronto, Canada, ^2^University of Ottawa, Department of Anesthesiology and Pain Medicine, Ottawa, Canada, ^3^Kingston Health Sciences Centre, Kingston, Canada

*Critical Care* 2022, **26(Suppl 1):** P074

**Introduction:** While empiric dosing of fibrinogen replacement is widely utilized and well-tolerated, it may be suboptimal in some cardiac surgery patients, leading to inadequate bleeding control due to acquired hypofibrinogenemia. This may lead to additional transfusion requirements, impacting clinical outcomes. Our aim was to compare the efficacy and safety of weight-adjusted *vs.* empiric dosing of fibrinogen replacement.

**Methods:** This *post-hoc* analysis of the FIBRES randomized controlled trial included adults experiencing clinically significant bleeding and hypofibrinogenemia after cardiac surgery at 11 Canadian centers and utilized empiric dosing of fibrinogen concentrate (FC; Fibryga®, Octapharma; 4 g) or cryoprecipitate (10 IU) [1]. Patients were grouped into quartiles based on increasing weight-adjusted dosing of either product. The primary outcome was the number of red blood cell (RBC) units transfused within 24 h of cardiopulmonary bypass (CPB).

**Results:** The median weight-adjusted FC dose was 52 mg/kg (IQR 45–61; n = 372) and 1.30 U/10 kg (IQR 1.11–1.54; n = 363) for cryoprecipitate. In patients receiving a single dose of either product where plasma fibrinogen level was measured pre and post dosing, the increase in plasma fibrinogen was higher with FC (0.96 g/l, IQR 0.74–1.28; n = 252) vs*.* cryoprecipitate (0.78 g/l, IQR 0.52–1.00; n = 225; *p* < 0.0001). There was no difference between patients in the quartile receiving the lowest weight-adjusted doses compared to higher quartiles in the number of RBCs (Table 1) or allogenic transfusions received within 24 h of CPB, return to the operating room, or thromboembolic/ischemic complications within 28 days.

**Conclusions:** Transfusion and safety outcomes for low and high weight-adjusted doses of fibrinogen replacement were comparable. Weight-adjusted dosing does not appear to offer advantages over fixed dosing in hypofibrinogenemic bleeding adult cardiac surgical patients.

**Reference**

1. Callum J et al. JAMA 322:1966–76, 2019.

**Table 1 (abstract P074):** Adjusted hierarchical generalized estimating equation models for number of red blood cells transfused within 24 hours of CPB. Poisson models accounting for clustering by study site, adjusted for surgical complexity, urgency, critical pre-operative status, sex, and age

Quartile	Fibrinogen concentrate		Cryoprecipitate	
	Mean (SD) dosing (mg/kg)	Relative risk (95% CI); *p* value	Mean (SD) dosing (IU/10 kg)	Relative risk (95% CI); *p* value
1	40 (5)	Reference	1.01 (0.09)	Reference
2	49 (2)	1.04 (0.77, 1.40); *p* = 0.81	1.21 (0.06)	0.82 (0.52, 1.27); *p* = 0.37
3	57 (2)	0.89 (0.70, 1.15); *p* = 0.38	1.39 (0.06)	1.17 (0.85, 1.60); *p* = 0.33
4	71 (10)	0.90 (0.71, 1.13); *p* = 0.36	1.78 (0.22)	1.04 (0.76, 1.43); *p* = 0.80

CI, confidence interval; CPB, cardiopulmonary bypass; IU, international units; SD, standard deviation

## P075

### Phenogrouping of hemorrhagic trauma patients using latent variable machine learning

#### C Nagpal^1^, A Dubrawski^2^

##### ^1^Carnegie Mellon University, Pittsburgh, USA, ^2^Carnegie Mellon University, School of Computer Science, Pittsburgh, USA

*Critical Care* 2022, **26(Suppl 1):** P075

**Introduction:** The CRASH2 trial [1] was previously conducted to estimate the effect of early administration (< 8 h from injury) of tranexamic acid (TXA) on outcomes such as mortality in a cohort of n = 20,21 bleeding trauma patients. 10,096 were treated with TXA (1 g over 10 min then infusion of 1 g over 8 h).

**Methods:** We applied Deep Cox Mixtures latent variable model [2] to recover phenogroups that demonstrate different survival rates in the CRASH2 population conditioned on baseline covariates (Age, Sex, Trauma type (blunt/penetrating), Systolic BP, Respiratory Rate, Heart Rate, Glasgow Coma Score). The recovered phenogroups were compared using Kaplan–Meier estimators and SHAP [3] values were used to explain the phenogroups in terms of the baseline patient variables. Multivariate Cox regression was performed to identify phenogroup-specific predictors of survival.

**Results:** Deep Cox Mixtures recovered 3 phenogroups from the CRASH2 study demonstrating differential mortality rates: High (Group A, n = 3,074, 13.68–15.00 days), Low (Group B, n = 12,277, 27.70–28.06 days) and Medium Risk (Group C, n = 4757, 24.30–25.00 days) (Times are 95% CIs of 30-day restricted mean survival). Hours since injury and age of patient were predictive of survival in high risk patients (Fig. 1). Nature of trauma was not found to be predictive for the Medium Risk patients.

**Conclusions:** Latent variable machine learning models can recover diverse patient phenogroups and have potential for supporting precise decisions, contextualized to specific subjects treated for trauma care with hemorrhage.

**References**

1. Crash-2 Collaborators. Lancet 377:1096–101, 2011.
2. Nagpal C et al. Proc Mach Learn Res 149:674–708, 2021
3. Lundberg SM et al. NeurIPS, 2017.

**Fig. 1 (abstract P075):**
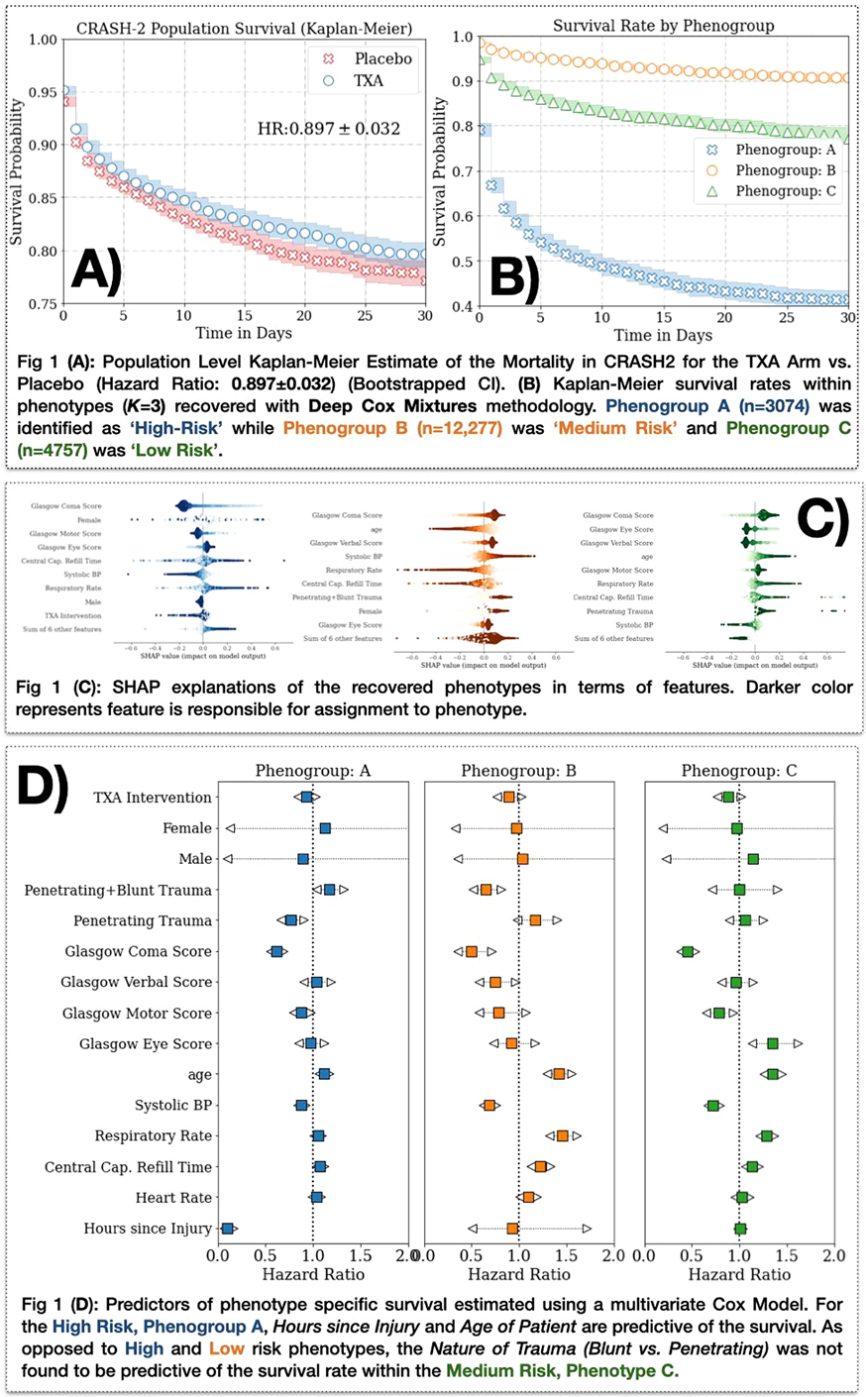
Survival rates and hazard ratios stratified by discovered phenogroups

## P076

### A phase 3, randomized, double-blinded study of four-factor prothrombin complex concentrate in patients with acute major bleeding on direct oral anticoagulant therapy with factor Xa inhibitors: the LEX-210 study

#### R Sarode^1^, S Maack^2^, C Solomon^2^, S Knaub^2^, S Schulman^3^

##### ^1^UT Sothwestern, Dallas, USA, ^2^Octapharma, Octapharma AG, Lachen, Switzerland, ^3^McMaster University, Thrombosis and Atherosclerosis Research Institute and Dept of Medicine, Hamilton, Canada

*Critical Care* 2022, **26(Suppl 1):** P076

**Introduction:** LEX-210 aims to demonstrate hemostatic efficacy/safety of four-factor prothrombin complex concentrate (4F-PCC; Octaplex®, Octapharma) in adults with acute major bleeding on direct oral anticoagulant (DOAC) therapy with Factor Xa Inhibitors (FXaI). Patients on FXaI can experience major bleeding associated with substantial morbidity, mortality, and hospitalization. Therefore, reversal/hemostatic agents are used to control FXaI-related bleeding. The efficacy/safety of PCCs as hemostatic agents for FXaI-related bleeding requires further investigation.

**Methods:** LEX-210 (NCT04867837) is a Phase 3, multicenter, prospective, randomized, double-blinded, group-sequential, parallel-group, adaptive design study. Key inclusion criteria include major bleeding and DOAC level ≥ 100 ng/ml equivalent; exclusion criteria include life-threatening bleeding and acute trauma for which hemostatic agent alone would not control bleeding. LEX-210 will enroll ~ 200 patients, randomized 1:1 to receive 50 IU/kg or 15 IU/kg 4F-PCC to demonstrate superior hemostatic efficacy of high dose 4F-PCC for emergent FXaI-related major bleeding. The primary endpoint is the proportion of patients with effective (excellent/good rating) or non-effective (poor/none rating) hemostasis in bleeding management within 24 h of 4F-PCC, as assessed by an independent adjudication committee according to predefined criteria (Fig. 1) [1]. Secondary endpoints include changes in endogenous thrombin potential; 30-day rate of thromboembolic events, all-cause mortality and adverse events; vital signs; and laboratory parameters.

**Results:** LEX-210 commenced in Q4 2021 and will be performed at ~ 60 sites in North America and Europe. Completion is expected Q1 2024.

**Conclusions:** If results confirm 4F-PCC hemostatic efficacy/safety in the management of FXaI-related major bleeding, it would offer an alternative for the management of major bleeding in these patients.

**Reference**

1. Sarode R et al. Circulation 128:1234–43, 2013

**Fig. 1 (abstract P076):**
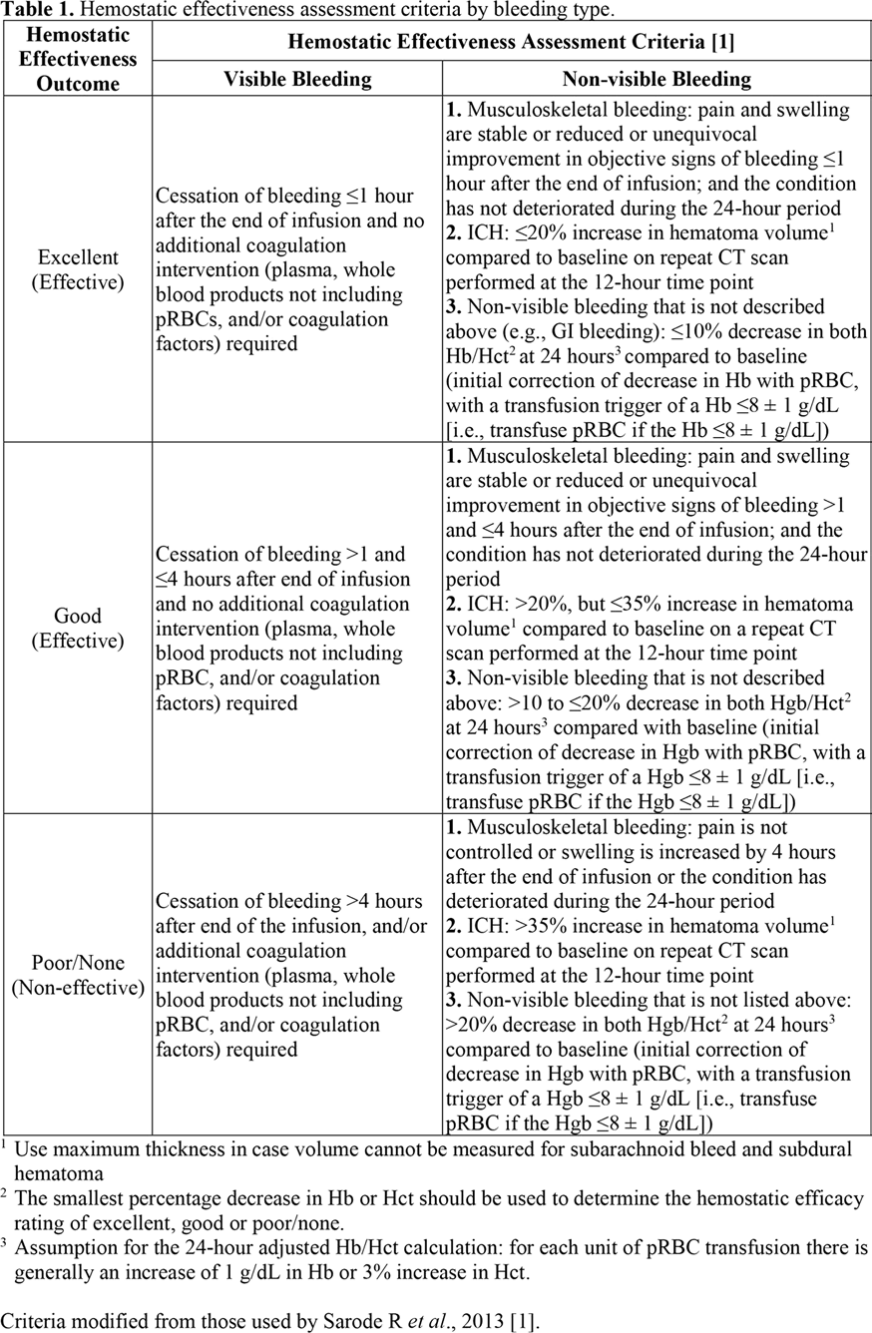
Hemostatic effectiveness criteria

## P077

### Impaired fibrinolysis is implicated in mortality in COVID-19 infection

#### O Watson^1^, M Howard^2^, J Cezar-Zaldua^2^, J Whitley^2^, S Pillai^2^, M Lawrence^2^, B Burgess^2^, K Hawkins^3^, K Morris^4^, PA Evans^2^

##### ^1^Welsh Centre for Emergency Medicine Research, Emergency Department, Swansea, UK, ^2^Welsh Centre for Emergency Medicine Research, Swansea, UK, ^3^Swansea University, Swansea, UK, ^4^Cardiff Metropolitan University, Cardiff, UK

*Critical Care* 2022, **26(Suppl 1):** P077

**Introduction:** A significant degree of mortality and morbidity in COVID-19 is due to thromboembolic disease. Changes in coagulation markers have been well described in critically unwell patients on ICU. There is less clear evidence regarding these changes at the time of presentation to the Emergency Department and the progression of disease over time. We sought to investigate how coagulation markers change over the course of COVID-19 infection and whether they might predict disease severity.

**Methods:** Patients were recruited from a single University Teaching Hospital ED at the time of presentation. Those with a positive PCR test were followed up throughout their stay. Rotational thromboelastometry (ROTEM) was performed on arrival, after 24 h, 3–5 days and 7 days, alongside routine haematological and biochemical testing. ROTEM values at each of these time points were analysed, and compared. Length of stay and patient outcome were also recorded for subgroup analysis. The ROTEM parameters selected for analysis were both EXTEM and INTEM Clotting Time (CT), Clot Formation Time (CFT), Maximal Clot Firmness (MCF), Alpha Angle (Alpha) and Maximum Lysis Percentage (ML). This reflects clot formation kinetics, mechanical strength and clot breakdown via both extrinsic and intrinsic pathways.

**Results:** EXTEM (7.64 ± 5.53 vs 11.83 ± 6.30) and INTEM ML (4.69 ± 3.55 vs 9.95 ± 5.22) were significantly reduced in those who died vs patients with a prolonged hospital stay. Over time there were no patterns of change to ROTEM values in any outcome group.

**Conclusions:** Comparisons between groups demonstrated that one distinguishing feature between those who require ICU admission or die of COVID-19 compared with those who survive a prolonged hospital stay to discharge was the extent to which fibrinolysis could occur. Failure to break clots down could be a significant mechanism in the mortality and morbidity of COVID-19.

## P078

### Viscoelastic testing in COVID-19 infection demonstrates a resistance to low molecular weight heparin

#### O Watson^1^, M Howard^2^, J Cezar-Zaldua^2^, J Whitley^2^, S Pillai^2^, M Lawrence^2^, K Guy^2^, K Hawkins^3^, K Morris^4^, PA Evans^2^

##### ^1^Welsh Centre for Emergency Medicine Research, Emergency Department, Swansea, UK, ^2^Welsh Centre for Emergency Medicine Research, Swansea, UK, ^3^Swansea University, Swansea, United Kingdom, ^4^Cardiff Metropolitan University, Cardiff, UK

*Critical Care* 2022, **26(Suppl 1):** P078

**Introduction:** A significant degree of mortality and morbidity in COVID-19 is due to thromboembolic disease. We use viscoelastic testing to investigate changes to coagulation profile over the progression of COVID-19 infection.

**Methods:** Patients presenting to a single large University Teaching Hospital ED were recruited at presentation. Those with positive COVID-19 PCR test were included for analysis. Whole blood samples were taken for viscoelastic tests. Fractal Dimension (D_*f*_) and Time to Gel Point (T_GP_) are biomarkers of thrombogenicity which measure the biomechanical properties of the incipient clot [1]. Patients were followed up throughout their hospital stay, with sampling taken at arrival, after 24 h, 3–5 days and 7 days. Length of stay and patient outcome were recorded and used for subgroup analysis. Once admitted to the hospital all patients received low molecular weight heparin (LMWH) as per standard treatment pathways, if commenced before the first sample was taken, this was recorded and controlled for.

**Results:** D_*f*_ and T_GP_ showed no changes over time in COVID-19 infection. Subgroup analysis also showed no differences in D_*f*_ or T_GP_ in different outcome groups. Patients who received LMWH from the clinical team before recruitment to the study demonstrated no significant difference in Df (1.715 ± 0.061 no LMWH vs 1.699 ± 0.068 with LMWH), but T_GP_ was prolonged in those receiving LMWH(445.0 ± 195.2 vs 307.6 ± 91.6). Additionally there was no correlation between Anti-Xa level and D_*f*_.

**Conclusions:** The therapeutic efficacy of LMWH appears to be blunted in COVID-19 infection. This may be due to the inflammatory state creating a resistance to the activity of LMWH, and may in part explain why LMWH appears to have less effect in reducing thromboembolic disease in COVID-19 than it does in other disease states.

**Reference**

1. Evans et al. Blood 116:3341–6, 2010

## P079

### Caplacizumab rapidly inhibits vWF–platelet interaction: pharmacodynamic data from healthy volunteers and patients with aTTP

#### F Callewaert^1^, J Minkue Mi Edou^2^, R De Passos Sousa^3^

##### ^1^Sanofi, Diegem, Belgium, ^2^Sanofi, Ghent, Belgium, ^3^Sanofi, Lisbon, Portugal

*Critical Care* 2022, **26(Suppl 1):** P079

**Introduction:** Caplacizumab targets the A1 domain of von Willebrand factor (vWF) and inhibits vWF–platelet interaction. In clinical trials in patients with acquired thrombotic thrombocytopenic purpura (aTTP), the 10 mg dosing regimen of caplacizumab completely blocked vWF-mediated platelet adhesion within 24 h. The aim of this study was to further characterize the speed of action of caplacizumab.

**Methods:** vWF activity data (ristocetin cofactor [RICO] assay) from a Phase 1 study with caplacizumab in healthy White and Japanese volunteers (single intravenous [IV] or subcutaneous [SC] 10 mg dose; n = 16 per group), and from the Phase 2 TITAN study in a subset of patients (n = 12) with RICO sampling at 5–10 min, 3–6 h, and 8–24 h after the IV loading dose, were included in this analysis. RICO inhibition to < 20% reflects full neutralization of vWF–platelet binding by caplacizumab. Informed consent was obtained from all study participants.

**Results:** Complete inhibition of RICO activity was achieved in 15/16 healthy subjects (94%) at 1 h after caplacizumab IV dosing, and in all participants at 3 h after dosing (Table 1). With the 10 mg SC dose, RICO activity < 20% was achieved in half of subjects (8/16) after 1 h and in all subjects after 3 h. RICO remained suppressed for 24 h in 30/32 volunteers after a single IV or SC dose and started to recover thereafter. In TITAN, Day 1 RICO activity values were available for 11/12 patients; 8/11 (72.7%) achieved RICO < 20% within 5–10 min after the first IV loading dose, and the remaining 3 patients (27.3%) after 3–6 h. In 8/12 patients with available data, RICO remained < 20% at 8–24 h after the IV loading dose.

**Conclusions:** Caplacizumab, through its IV loading dose, induces rapid and sustained inhibition of vWF–platelet interaction, starting within minutes in most patients, which is essential in a life-threatening disease like aTTP.

This study and editorial support funded by Ablynx, a Sanofi company. Previously presented at 29th ISTH Congress.

**Table 1 (abstract P079):** Effect of caplacizumab on RICO activity in the healthy volunteer study

	10 mg IV	10 mg IV	10 mg SC	10 mg SC
Analysis time point at Day 1, n (%)	White (n = 8)	Japanese (n = 8)	White (n = 8)	Japanese (n = 8)
1 hour post-dose: RICO < 20% / RICO ≥20%	7 (87.5) / 1 (12.5)	8 (100.0) / 0	4 (50.0) / 4 (50.0)	4 (50.0) / 4 (50.0)
3 hours post-dose: RICO < 20% / RICO ≥20%	8 (100.0) / 0	8 (100.0) / 0	8 (100.0) / 0	8 (100.0) / 0
24 hours post-dose: RICO < 20% / RICO ≥20%	6 (75.0) / 2 (25.0)	8 (100.0) / 0	8 (100.0) / 0	8 (100.0) / 0
48 hours post-dose: RICO < 20% / RICO ≥20%	1 (12.5) / 7 (87.5)	0 / 8 (100.0)	5 (62.5) / 3 (37.5)	6 (75.0) / 2 (25.0)
72 hours post-dose: RICO < 20% / RICO ≥20%	0 / 8 (100.0)	0 / 8 (100.0)	0 / 8 (100.0)	0 / 8 (100.0)

IV, intravenous; RICO, ristocetin cofactor; SC, subcutaneous. RICO activity was ≥20% in all healthy volunteers at baseline

## P080

### Caplacizumab induces fast and durable platelet count responses with improved time to complete remission and recurrence-free survival in patients with acquired thrombotic thrombocytopenic purpura

#### P Coppo^1^, M Scully^2^, J De la Rubia^3^, F Peyvandi^4^, S Cataland^5^, JA Kremer Hovinga^6^, P Knoebl^7^, K Pavenski^8^, J Minkue Mi Edou^9^, R De Passos Sousa^10^

##### ^1^Saint-Antoine University Hospital, AP-HP, Paris, France, ^2^University College London Hospital, London, UK, ^3^Catholic University of Valencia; Hospital Doctor Peset, Valencia, Spain, ^4^Fondazione IRCCS Ca' Granda Ospedale Maggiore Policlinico; Università degli Studi di Milano, Milan, Italy, ^5^The Ohio State University, Columbus, OH, USA, ^6^Inselspital, Bern University Hospital, University of Bern, Bern, Germany, ^7^Medical University of Vienna, Wien, Austria, ^8^St. Michael’s Hospital, University of Toronto, Toronto, Canada, ^9^Ablynx, a Sanofi company, Zwijnaarde, Belgium, ^10^Sanofi, Lisbon, Portugal

*Critical Care* 2022, **26(Suppl 1):** P080

**Introduction:** Key therapeutic goals in patients with acquired thrombotic thrombocytopenic purpura (aTTP) are to rapidly control platelet consumption and maintain durable remission. In the Phase 3 HERCULES trial (NCT02553317), caplacizumab (CPLZ) treatment resulted in significantly faster time to platelet count normalization versus placebo (PBO). Aim: to characterize the durability of platelet count responses in the HERCULES trial.

**Methods:** This was a post hoc analysis of the HERCULES intent-to-treat population (CPLZ, n = 72; PBO, n = 73). We identified patients with fast platelet count response and described the exacerbation rate. Time to durable platelet count response, time to complete remission (CR), and recurrence-free survival (RFS) were calculated.

**Results:** Most patients achieved initial platelet count normalization ≤ 3 days (CPLZ, 56/72 [78%]; PBO, 43/73 [59%]). In patients with fast platelet count response (≤ 3 days), exacerbation rate was 4% (2/56) with CPLZ and 44% (19/43) with PBO. In patients with time to platelet count response > 3 days, exacerbation rate was 7% (1/15) with CPLZ and 30% (9/30) with PBO. Of patients who had exacerbations, 90% (CPLZ, 2/3; PBO, 26/28) switched to open-label CPLZ, which may have favored outcomes of PBO patients. Median (95% confidence interval) time to durable response was 4.5 (4.4–4.6) days with CPLZ and 10.5 (6.5–14.5) days with PBO (Fig. 1A); median time to CR was 40.0 (37.7–41.1) days with CPLZ and 44.2 (42.0–48.2) days with PBO (Fig. 1B). Overall RFS during the study period demonstrated early and sustained benefit for CPLZ over PBO (Fig. 1C).

**Conclusions:** CPLZ was associated with a faster and sustained platelet count response versus PBO, where many fast responders subsequently had an exacerbation. Fast platelet count responses with CPLZ were maintained and translated into clinically relevant improvements in time to CR and overall RFS.

This study and editorial support funded by Ablynx, a Sanofi company. Previously presented at 25th EHA Congress and 62nd ASH Meeting.

**Fig. 1 (abstract P080):**
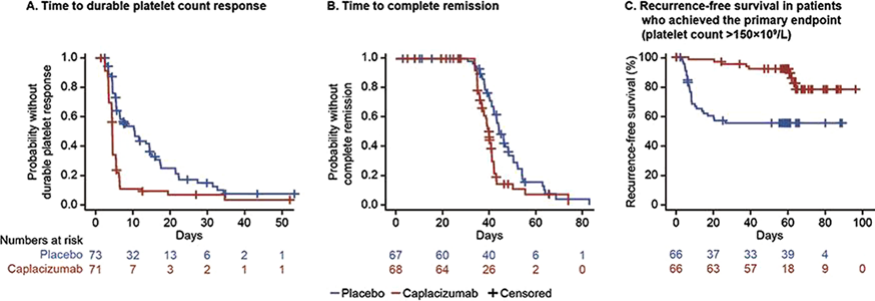
Time to durable platelet count response was defined as time to last daily TPE during the overall treatment period. Time to complete remission was defined as platelet count > 150×10^9^/l and lactate dehydrogenase < 1.5× the upper limit of normal for > 30 days after cessation of daily TPE. Recurrence-free survival was defined as absence of exacerbation or relapse during the overall study period. TPE, therapeutic plasma exchange.

## P081

### Evaluation of a pharmacist-led protocol for antixa-based enoxaparin dosing in trauma patients

#### JE Murphy, EN Morales, TS Lam, JT Jancik

##### Hennepin Healthcare, Department of Clinical Pharmacy, Minneapolis, USA

*Critical Care* 2022, **26(Suppl 1):** P081

**Introduction:** Patients with trauma are at high risk for venous thromboembolism (VTE) [1]. AntiXa (aXa) monitoring of prophylactic low molecular weight heparin (LMWH) is recommended by the 2002 EAST guidelines following initiation of therapy or after dose changes in some patients [2]. This study evaluates the efficacy of a pharmacist-led protocol for enoxaparin dose adjustment based on aXa levels in trauma patients.

**Methods:** This single-center retrospective chart review included adult trauma patients admitted from 3/1/2018 to 6/20/2020. Patients received LMWH per protocol based on body mass index (BMI). Patients with BMI < 40 kg/m^2^ received enoxaparin 30 mg twice daily, and patients with BMI ≥ 40 kg/m^2^ received enoxaparin 40 mg twice daily. The goal therapeutic range based on peak level was 0.2–0.4 IU/ml. The primary objective was time to goal peak aXa level after enoxaparin initiation. Secondary objectives include enoxaparin dosing regimen in milligrams and milligrams per kilogram, number of adjustments to reach goal level, rates of VTE, and rates of bleeding attributed to enoxaparin.

**Results:** Ultimately, 635 patients met inclusion criteria. Median time to goal antiXa was 2 days (IQR 2–4) [Table 1]. Peak aXa levels were within the goal range for 43.2% of patients on the first check. Of the 66 patients with BMI ≥ 40 kg/m^2^, 14/66 (21.2%) were initially dosed below protocol and 20/66 (30.3%) of these patients met goal aXa at first check. Of the 569 patients with BMI < 40 kg/m^2^, 535/569 (94.0%) were initially dosed according to protocol and 254/569 (44.6%) of these patients met goal antiXa at first check. Rates of VTE were similar for all patients, and the overall rate of bleeding was low.

**Conclusions:** This study demonstrates the safety of a pharmacist-led enoxaparin dosing protocol based on low rates of bleeding events. The rate of VTE events was low despite less than half of patients achieving goal aXa level on first check.

**References**

1. Walker CK et al. Ann Pharmacother 51:323–331, 2017.
2. Rogers FB et al. J Trauma 53:142–164, 2002.

**Table 1 (abstract P081):** Outcome measures

	All patients, N = 635	BMI < 40 kg/m^2^, N = 569	BMI > 40 kg/m^2^, N = 66
Median time to goal aXa (days)[IQR]	2 [2–4]	2 [2–4]	3 [2–4]
AXa within goal at first check [%]	274/635 [43.2%]	254/569 [44.6%]	20/66 [30.3%]
Rate of VTE [%]	11/635 (1.73%)	10/569 [1.76%]	1/66 [1.51%]
Rate of bleeding [%]	4/635 (0.63%)	4/569 [0.70%]	0/66 [0.00%]

## P082

### Perioperative behavior of inflammatory markers in cytoreductive surgery with hyperthermic intraperitoneal chemotherapy

#### GA Madrid^1^, CA Triana^1^, EE Rodríguez^2^, ES Calle^3^

##### ^1^Fundación Santa Fe de Bogotá, Anesthesiology Department, Bogotá, Colombia, ^2^Fundación Santa Fe de Bogotá, Intensive Care Department, Bogotá, Colombia, ^3^Universidad de los Andes, School of Medicine, Bogotá, Colombia

*Critical Care* 2022, **26(Suppl 1):** P082

**Introduction:** Cytoreductive surgery with hyperthermic intraperitoneal chemotherapy (CRS/HIPEC) is the treatment of various abdominal and gynecological malignancies. Complexity of the procedure and effects of hyperthermia demand patients are recovered in the ICU. Biomarkers that identify patients at risk of postoperative complications could guide management in this setting [1]. Our purpose was to explore associations between neutrophil-to-lymphocyte ratio (NLR) and platelet-to-lymphocyte ratio (PLR) to clinical outcomes. We hypothesized inflammation is associated with worse outcomes.

**Methods:** A retrospective cohort study including patients taken to CRS/HIPEC in 2017 at a university hospital was conducted. Patients were excluded if they presented an active infection, surgical trauma, and lacked preoperatory laboratories. NLR and PLR were calculated from complete blood counts obtained preoperatively, 24-, 48-, 72-h and 7 days after surgery. Inflammation was defined as NLR > 2.5 and PLR > 90. Primary outcomes were ICU stay, hospital stay, days of mechanical ventilation, and days to start ambulation and oral intake. Mann–Whitney and chi-square test were carried as appropriate.

**Results:** We included 127 patients in our study. Inflammation according to NLR was present in 30.7%, 96.9%, 99.2%, 98.4%, and 91.3% of patients before surgery, 24-, 48-, 72-h and 7 days after surgery, respectively. Inflammation according to PLR was present in 92.1%, 80.3%, 81.1%, 83.5%, and 92.9% at the same time points. A PLR > 90 48-h postoperative was associated with more days of mechanical ventilation (*p* 0.049). Inflammation determined by NLR and PLR was not associated with other outcomes at the timepoints evaluated.

**Conclusions:** CRS/HIPEC is a complex procedure that requires ICU care. NLR and PLR do not predict outcomes. More studies are needed to identify markers that can impact the prognosis of CRS/HIPEC patients.

**Reference**

1. Rangarajan K et al. Int J Hyperthermia 34:559–563, 2018.

## P083

### Chimeric antigen receptor (CART) cell therapy: experience from a specialist cancer intensive care unit

#### A Patel^1^, K Naik^1^, E Nicholson^2^, P Gruber^1^

##### ^1^The Royal Marsden Hospital, Intensive Care Unit, London, UK, ^2^The Royal Marsden Hospital, Haematology, London, UK

*Critical Care* 2022, **26(Suppl 1):** P083

**Introduction:** CART cell therapy has revolutionized treatment for cancer patients. Primarily indicated for the treatment of relapsed large B cell lymphomas (DLBCL) and acute myeloid leukaemias (AML), but now the use of CART therapy been extended for use for other solid organ cancers. The use of CART is frequently associated with toxicities, such as cytokine release syndrome (CRS) and immune effector cell associated neurotoxicity syndrome (ICANS) necessitating critical care admission. We describe 2 years data of outcomes of patients receiving CART therapy from a cancer specialist centre in the United Kingdom.

**Methods:** A retrospective analysis undertaken of all patients undergoing CART cell therapy at The Royal Marsden Hospital between February 2019 and May 2021.

**Results:** We studied a total of 20 patients with a median age of 62 (IQR 46–72) years, 60% male, 95% with relapsed DLBCL and 5% with AML. 50% were admitted to the intensive care unit (ICU) with a median length of ICU stay of 5.5 (IQR 4–15.2) days. ICU admission rates reduced from 85.7% in 2020 to 25% in 2021 until May. 20% needed cardiovascular support, 15% needed intubation and 5% required high flow nasal oxygen. The median length of stay in hospital was 25.5 (IQR 18.5–32.7) days. Table 1 illustrates the demographics of CART patients with neurotoxicity during hospital admission. Using outcome analysis, by day 28, 60% of patients were in complete remission and 15% of patients were refractory to treatment. In terms of patient survival, 95% were alive at day 28 and 66.6% at 12 months.

**Conclusions:** CART cell therapy patients have high ICU requirements with relatively high rates of toxicities, but this is changing with novel therapies, better patient selection and greater confidence in management of these patients in the ward setting. Excellent communication between oncologists, critical care outreach team and the ICU team and education of ward staff is key in ensuring prompt identification and escalation of care in CART patients at risk of deterioration.

**Table 1 (abstract P083):** The demographics of our cohort of CART patients who developed neurotoxicity. CRS and ICANS are graded between 1 and 4, where 4 is most severe.

	Grade	Number of patients	% of cohort	Median day of onset (IQR)
CRS	≥1	19	95	1 (1–3)
	3 or 4	5	25	
ICANS	≥1	9	45	7 (4–8)
	3 or 4	5	25	

## P084

### Incidence of persistent ventilation following emergency laparotomy—admission to an intensive care unit over 2 years

#### N Boyer^1^, K Mensah^1^, V Bennett^2^, S Green^1^, P Alexopolou^2^, S Huddart^2^, L Forni^1^, B Creagh-Brown^1^

##### ^1^Royal Surrey County Hospital, Intensive Care Department, Surrey, UK, ^2^Royal Surrey County Hospital, Anaesthetics Department, Surrey, UK

*Critical Care* 2022, **26(Suppl 1):** P084

**Introduction:** After surgery it is usual to awaken the patient and remove their artificial airway. There are some circumstances where persistent ventilation (PV) is continued. Data is limited to guide decision-making. Clinicians will choose not to extubate on the assumption that benefits outweigh the risks. Our hypothesis is that this strategy remains uncertain, with risks being potentially significant [1].

**Methods:** Our site, like most hospitals in the UK, submits data to National Emergency Laparotomy Audit. These were cross referenced with WardWatcher and ICCA, collated in Excel and analysed with GraphPad. Analyses were retrospective and did not include paper notes.

**Results:** In a two year period, 274 patients had surgery with data in the NELA database, 195 were admitted to ICU. Of these admissions 54 received PV (28%). Duration of ventilation was short, median 2 days. Hospital length of stay was significantly longer in PV (median 21.5 days vs 11.0 (*p* < 0.0001)). The PV group were sicker, with lower pH, higher lactate, higher dosing of vasopressor infusions and higher APACHE II score. 31% met criteria for moderate or severe ARDS. Receipt of blood transfusion (13%) and vasopressors (93%) were common (Table 1).

**Conclusions:** There no guidelines to inform decisions about PV following emergency laparotomy. Perhaps the most robust indication for PV is significantly impaired oxygenation or inadequate ventilation. Patients arriving out of hours were more likely to remain ventilated, the risks of extubation may not be greater [2]. Adverse outcomes in the PV group are likely to be influenced by the reasons for PV, however it’s conceivable that *in* *itself* PV could have a significant influence on outcome, and deserves further study.

**References**

1. Nabozny et al. Crit Care Med 44:1091–1097, 2016.
2. Krebs et al. J Thorac Cardiovasc Surg 157:1533–1542, 2019.

**Table 1 (abstract P084):** Table demonstrating patient characteristics

	All patients (n = 195)	Persistent ventilation (n = 54)	Self-ventilating (n = 141)
Age, mean (SD)	66.7 (15.21)	68.8 (13.8)	65.9 (15.83)
APACHE II score, median, (IQR)	12.0 (6)	13.0 (10.5–16)	12 (9–14)
pH, median, (IQR)	7.36 (0.09)	7.32 (7.25–7.36)	7.37 (7.33–7.44)
Lactate, median (IQR)	1.1 (1.0)	1.5 (1.0–3.025)	1 (0.1–1.6)
Noradrenaline dose (mcg/kg/min), median, (IQR)	0.11 (0.24)	0.18 (0.08–0.48)	0 (0.00–0.03)
Hospital LOS, median (IQR)	12.0 (14.0)	21.5 (12–33.25)	11.0 (7–16)
Moderate/ Severe ARDS, number (%)		17 (31%)	

## P085

### INTREPID project: intelligent toolkit for reconnaissance and assessment in perilous incidents

#### AM Cintora^1^, S Gomez de la Oliva^2^, P Blanco Hermo^2^, FJ Hernandez Prieto^2^, MD Semprun^2^, C Mendez^2^, C Navarro Sanguino^2^

##### ^1^SUMMA 112, Research Department of Emergency Service Madrid Community SUMMA 112, Madrid, Spain, ^2^SUMMA 112, Madrid, Spain

*Critical Care* 2022, **26(Suppl 1):** P085

**Introduction:** The objective is to develop technologies that help to improve disaster response, facilitating safe and efficient performance of first responders in emergencies, taking into account the different risks, whether natural or man-made [1]. The objectives that have been set are:

- To develop tools to facilitate the exploration and assessment of potentially hazardous inhabited spaces.
- To improve the safety and efficiency of first responders [2].

**Methods:** The INTREPID project is based on the collaborative work of research, technology and communications organizations focus on technology development within drones and robots unmanned. This tolls are testing by European emergency services.

- Mobile platform for scanning and assessment of a disaster area, for multidisciplinary teams
- Drones and robots that will act as cybernetic assistants.
- Positioning, mapping and environmental assessment module.
- Tactical communications system for disasters.

The first testing exercise was developed in Stockholm [2]:

- Metro flooding on 2 November 2021

**Results:** Key Performance Indicators [2].

Unmanned aerial vehicles have been tested in emergencies indoor with these new capabilities.

- Positioning accuracy of indoor UAV 10 cm 50

Unmanned ground vehicles with target value of:

- Ability to climb stairs and an arm reach of 1.3 m
- Min range of network full unit 1 km
- Max deployment time of network full unit 10
- Number of concurrent users of network full unit 20 Subject

**Conclusions:** Innovation with information technology (ICT), building sensors, mixed reality and autonomous vehicles, increases the efficiency in the attention to major catastrophes. Effective inter-group communication, obtaining information in real time and coordination through technology adapted to our needs, helps to reduce the initial chaos of the catastrophe, improve our assistance to patients and increase our safety in the intervention [2].

**References**

1. https://cordis.europa.eu/project/id/883345/es Last access 01/12/2021
2. https://intrepid-project.eu/ Last access 01/12/2021

## P086

### Burden of decision making and cognitive function amongst high consequence decision-makers in intensive care

#### N Khalil, P Shah, J Chui, P Kotecha, M O’Connor, V Sathianathan

##### Northwick Park Hospital, Intensive Care Unit, London, UK

*Critical Care* 2022, **26(Suppl 1):** P086

**Introduction:** Decision fatigue is a crucial concept in the intensive care unit (ICU) setting; it is the idea that after making many decisions, a person's ability to make additional decisions becomes worse. ICU consultants make numerous high consequence decisions on patients with complex pathology in time-critical scenarios. The aim of the study was to identify the density of decision making and its consequences on the quality of the decisions made by ICU consultants in our unit.

**Methods:** This study took place across four twenty-four-hour on-call periods. Each consultant was monitored by a dedicated ICU trainee who recorded the daytime data. Demographic variables included the age, seniority of the ICU consultants, acuity of patients on the unit and staffing gaps. Baseline measurements included the number of days the consultant had been on the unit, their decision fatigue scale (DFS) score [1] and their reaction times. Real-time measurements of the number of decisions and interruptions during the on-call period were documented.

**Results:** Our study demonstrated increased reaction times and DFS scores for all consultants post-on-call compared with their baseline measurements (Table 1).

**Conclusions:** This study highlighted the number of decisions and interruptions consultants experience throughout their day. Signs of decision fatigue and reduced confidence in decision making were evident. Strategies to help reduce this could include moving non-urgent decisions to an alternative time of the day and a 'silent cockpit' rule to reduce non-urgent interruptions during ward rounds.

**Reference**

1. Hickman RL Jr et al. West J Nurs Res 40:191–208, 2018

**Table 1 (abstract P086):** Overall findings from Decision Fatigue Study

Consultant number	1	2	3	4
Change in DFS score	+23	+32	+24	N/A
Change in reaction time	+133%	+100%	+24%	+14.6%
Morning decisions	218	250	321	158
Morning interruptions	48	60	33	20
Afternoon decisions	123	92	63	85
Afternoon interruptions	14	13	5	11

## P087

### Comparison of the psychological impact between critical care and non-critical care nurses during the first year of the COVID-19 pandemic

#### M Vreven, H Vanden Eede

##### AZ Rivierenland, Department of Anaesthesiology and Intensive Care, Rumst, Belgium

*Critical Care* 2022, **26(Suppl 1):** P087

**Introduction:** We compared the psychosocial impact of the COVID-19 pandemic on nurses in our intensive care unit (ICU), emergency department (ED), and non-critical care departments.

**Methods:** Self-report questionnaires were supplied to all nurses in employ during four periods in the first year of this pandemic. General questions were supplemented with standardized tests: anxiety (GAD7); insomnia (ISI); traumatic impact (IESr); and depression (PHQ9).

**Results:** During four periods, 255 questionnaires were completed, including 52 by ICU and 38 by ED nurses. See Table 1 for a summary of the results and relevant p values. IES-r median scores were highest among ICU nurses (16.5). ICU nurses reported more sadness about working with COVID-19 patients as compared to non-critical care nurses (relative risk, RR 3.8). At the same time ICU nurses reported more often feeling sufficiently safe as compared to ED (RR 1.4) and non-critical nurses (RR 1.3). Leisure time for ICU nurses consisted more often of passive indoor activities (RR 2.3) and hobbies (RR 2.2) as opposed to more active leisure activities by non-critical care nurses. Most results for ICU nurses remained consistent regardless of registration period. However their fear decreased over time (RR 0.3, *p* = 0.012) and they felt more sufficiently trained (RR 1.5, *p* = 0.046). ED nurses felt less committed towards patient care when compared to non-critical care nurses (RR 0.9). ED nurses felt the hospital was less prepared to deal with continuing outbreaks when compared to non-critical care nurses (RR 0.5). On other subjects ED nurses did not differ significantly.

**Conclusions:** The psychological impact of the COVID-19 pandemic seems to be worse on our ICU nurses when compared to ED nurses and non-critical care nurses. The impact of the pandemic seems to be more traumatic causing more sadness among our ICU nurses with less active leisure activities. Our ED nurses on the other hand feel the hospital is less prepared for ongoing outbreaks and feel less motivated to continue care.

**Table 1 (abstract P087):** Overview of significant differences between nursing groups

	ED nurse	ICU nurse	Non-critical care nurse	*p* value
IES-r score (traumatic impact); median	12.0	16.5	10.0	0.025 (Dunn’s test)
Sadness towards possible COVID-19 exposure	8.1%	11.8%	3.1%	0.026 (Fisher’s Exact)
Sufficiently safe	67.6%	92.0%	72.6%	0.008; .003 (Fisher’s Exact)
Passive leisure time inside	24.2%	41.7%	18.1%	< 0.001 (Chi^2^)
Hobbies as leisure time	27.3%	29.2%	13.2%	0.011 (Chi^2^)
Full commitment towards patient care	83.3%	89.6%	95.7%	0.006 (Chi^2^)
Sufficiently prepared for new outbreak	40.0%	77.3%	72.9%	0.016 (Chi^2^)

## P088

### Impact of extracorporeal membrane oxygenation on burnout development in intensive care units

#### A Omar^1^, A Labib^2^, S Hanoura^2^, A Rahhal^2^, R Kaddoura^2^, T Chughtai^2^, E Karic^2^, M Shaikh^2^, W Hamad^2^, M Khatib^2^

##### ^1^Hamad Medical Corporation, Cardiothoracic Surgery, Doha, Qatar, ^2^Hamad Medical Corporation, Doha, Qatar

*Critical Care* 2022, **26(Suppl 1):** P088

**Introduction:** Burnout syndrome (BOS) has been recognized for over 50 years. Over time, it has been reported that certain health care specialties are more vulnerable to BOS, such as those working in an intensive care unit (ICU). The introduction of extracorporeal membrane oxygenation (ECMO) and its growing demand, adds to the overall workload in ICU, and exposes practitioners to complex ethical and administrative situations, which may impact their psychological well-being. We aim to investigate the effects of an ECMO service, on BOS development in the ICU.

**Methods:** We conducted a cross-sectional descriptive study, using an online questionnaire; The Maslach Burnout Inventory Human Services Survey for Medical Personnel. In addition, demographic variables, workload, salary satisfaction, and caring for coronavirus disease 2019 (COVID-19) patients were assessed. Participants were divided based on working in ICU with ECMO service into ICU with (ECMO-ICU) and without (non-ECMO-ICU) ECMO service, and burnout status (burnout and no burnout).

**Results:** The response rate for completing the questionnaire was 36.4% (445/1222). Males represented 53.7% of the participants. The overall prevalence of burnout was 64.5%. The overall burnout prevalence did not differ between ECMO- and non-ECMO-ICU groups (64.5% and 63.7, respectively). However, personal accomplishment (PA) score was significantly lower among ECMO-ICU personnel compared to those in a non-ECMO ICU (42.7% versus 52.6, *p* = 0.043). Significant predictors of burnout included profession (nurse or physician), acquiring COVID-19 infection, knowing other practitioners who were infected with COVID-19, salary dissatisfaction, and extremes of workload.

**Conclusions:** Burnout was equally prevalent among participants from ECMO- and non-ECMO ICU, but PA was lower among participants in ICU with an ECMO service. The reported high prevalence of burnout, and its predictors, requires special attention to try and reduce its occurrence.

## P089

### The impact of COVID-19 pandemic on intensive care workload at Mater Dei Hospital in Malta.

#### C Mizzi, MB Buttigieg, S Sciberras, C Tua, S Santucci, K Torpiano, N Grech, M Drake, B Spiteri

##### Mater Dei Hospital, Intensive Care, Msida, Malta

*Critical Care* 2022, **26(Suppl 1):** P089

**Introduction:** The aim of the study was to determine the impact of COVID-19 pandemic on intensive care workload [1,2] at our only acute main general hospital on the island. During the pandemic surge in March 2021, our intensive care was running at 200% capacity. Mater Dei Hospital has a 20-bedded adult intensive care catering for a population of 500,000.

**Methods:** This is a prospective cohort study conducted in the COVID-19 Intensive Care Unit at Mater Dei Hospital, Malta. Data analysed is from March 2020 to May 2021. Data collected daily from admission until death or discharge from ICU.

**Results:** A total of 261 patients with severe acute respiratory distress syndrome coronavirus 2 (SARS-Cov-2) required admission to our intensive care. ICU facilities required expansion into a total of 5 Intensive Care Units, therefore reaching a capacity of 44 intensive care beds during the peak month of March 2021. A maximum of 21 patients were admitted per week culminating to a total of 33 COVID-19 Intensive Care beds during the month of March 2021. A total of 179 patients (68.6%) required mechanical ventilation for a median duration of 11 days per patient. Proning was required in 124 mechanically ventilated patients (70.5%). 50 patients (20%) required CRRT with a maximum number of 7 patients per day requiring CRRT.

**Conclusions:** COVID-19 pandemic transformed the way how we provide critical care with improved bed capacity, ICU triage and ICU devices. This study highlighted the need for more clinical guidelines and their availability for online use. This will positively impact the care of non-COVID patients. It also highlighted the need for more training of non-ICU staff to allow for surges in ICU capacity. The COVID-19 pandemic has seen Mater Dei hospital already investing in ICU personnel and equipment as this cannot be reactive to large scale events but must be a proactive planned strategy to enhance resilience of our ITU.

**References**

1. Wynne R et al. J Clin Nurs, https://doi.org/10.1111/jocn.15916, 2021
2. Goh KJ et al. Crit Care 24:215, 2020

## P090

### Analysis of mitochondrial function in COVID-19 patients using *in vivo* and *ex vivo* techniques

#### LWJM Streng^1^, EG Mik^1^, CJ De Wijs^1^, NJH Raat^1^, PAC Specht^1^, M Van der Kaaij^1^, MH Wijnen^1^, D Sneiders^1^, H Endeman^2^, FA Harms^1^

##### ^1^Erasmus MC, Anesthesiology, Rotterdam, Netherlands, ^2^Erasmus MC, Intensive Care Unit, Rotterdam, Netherlands

*Critical Care* 2022, **26(Suppl 1):** P090

**Introduction:** Mitochondrial dysfunction has been linked to the persistent hypoxia and altered aerobic glycolytic metabolism seen in COVID-19 patients. This observational pilot study assessed mitochondrial function in COVID-19 patients and healthy controls (HC) utilizing *in* *vivo* and *ex* *vivo* techniques.

**Methods:** This single center observational study examined COVID-19 patients on two time points, the first within 72 h after intensive care admission (T1), and the second seven days after T1 (T2). HC were age and sex matched to the included COVID-19 patients. *In* *vivo* epidermal mitochondrial oxygen utilization was analyzed using the COMET (Cellular Oxygen METabolism) monitor, which employs the protoporphyrin-IX triplet state technique. *Ex* *vivo* measurements consisted of *in* *vitro* mitochondrial respiration analyzed by the Oroboros O2k respirometer and free mitochondrial DNA (fMtDNA) which was isolated from plasma and quantified by qPCR.

**Results:** 16 COVID-19 sepsis patients and 16 HC were included. The median MitoVO_2_ of COVID-19 patients on T1 was 4.6 mmHg s-1 [IQR; 3.6–6.0], 4.6 mmHg s-1 [IQR; 3.9–5.8] on T2 and 5.3 mmHg s^−1^ [IQR; 4.5–6.3] in the HC. Basal platelet respiration did not differ substantially between the three groups, whilst PBMC basal respiration was increased by approximately 80% in the T1 group when contrasted to T2 and the HC. fMtDNA was 14 times higher in the T1 group and 5 times higher in the T2 group when compared to the HC.

**Conclusions:** fMtDNA levels were increased in COVID-19 patients, but were not associated with decreased mitochondrial O_2_ consumption *in* *vivo* in the skin, and *ex* *vivo* in platelets or PBMC. This suggests the presence of mitochondrial stress, with concurrent preservation of mitochondrial respiration and function. It must be noted that due to the timing of T1, the optimal measurement window could have been missed. Therefore, the role of mitochondrial dysfunction in COVID-19 should be further evaluated at different time points.

## P091

### Naive T and B cells in COVID-19 patients

#### M Khadzhieva^1^, A Gracheva^2^ L Salnikova^2^, A Kuzovlev^3^

##### ^1^Federal Research and Clinical Center of Intensive Care Medicine and Rehabilitology; Dmitry Rogachev National Research Center of Pediatric Hematology, Oncology and Immunology; Vavilov Institute of General Genetics, Russian Academy of Sciences, Moscow, Russian Federation, ^2^Federal Research and Clinical Center of Intensive Care Medicine and Rehabilitology; Vavilov Institute of General Genetics, Russian Academy of Sciences, Moscow, Russian Federation, ^3^Federal Research and Clinical Center of Intensive Care Medicine and Rehabilitology, Moscow, Russian Federation

*Critical Care* 2022, **26(Suppl 1):** P091

**Introduction:** COVID-19 patients with acute respiratory distress syndrome (ARDS) have an immune imbalance when systemic inflammation and dysfunction of circulating T and B cells lead to a more severe disease. TREC (T cell receptor excision circle) formed during maturation of naive T cells in the thymus and KREC (kappa-deletion recombination excision circle) developed during maturation of naive B cells in bone marrow. Using TREC/KREC analysis, we studied the level of naive T and B cells in peripheral blood of COVID-19 patients.

**Methods:** TREC/KREC analysis was performed by multiplex real-time quantitative PCR on a DNA samples of peripheral blood. The total sample size was 36 patients from 18 to 45 years old; 10 (27.78%) patients had ARDS, and 4 (11.11%) of them did not survive.

**Results:** Patients with ARDS differed from the non-ARDS group ones in reduced lymphocyte count (*p* = 0.014), increased neutrophil count (*p* = 0.049), and neutrophil-to-lymphocyte ratio (NLR) (*p* = 0.002). During days 6 to 20 of hospitalization, a higher NLR was detected in ARDS patients compared with non-ARDS patients (Fig. 1A). Analysis of TREC/KREC levels both per 100,000 cells revealed significant differences: TREC/KREC values were lower in the group of ARDS patients; these differences persisted after adjustment for multiple comparisons (Fig. 1B). The TREC/KREC levels were also lower in non-survivors than in survivors. TREC/KREC negatively correlated with NLR; the highest correlation was recorded for TREC per 100,000 cells (Spearman’s rho =  − 0.726, *p* = 1.0 × 10E-06, coefficient of determination R^2^ = 0.527).

**Conclusions:** Thus, TREC/KREC analysis is a potential prognostic marker for assessing the severity and outcome in COVID-19.

**Fig. 1 (abstract P091):**
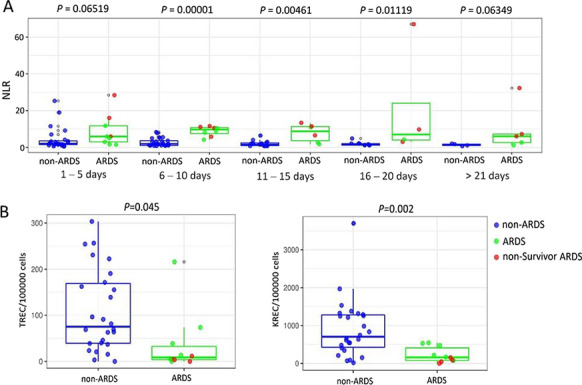
**A**—Comparison of NLR between ARDS and non-ARDS COVID-19 patients. **B**—Comparison of TREC/KREC levels per 100 000 cells between ARDS and non-ARDS COVID-19 patients.

## P092

### First own experience of intensive care multisystem inflammatory syndrome in children (MIS-C) associated with COVID-19.

#### I Yovenko^1^, D Gavrychenko^2^, N Belyakov^2^

##### ^1^Medical Home Odrex, Department of Anesthesiology and Intensive Care, Odessa, Ukraine, ^2^Medical Home Odrex, Odessa, Ukraine

*Critical Care* 2022, **26(Suppl 1):** P092

**Introduction:** Timeliness of diagnosis and treatment of MIS-C has increased amid the COVID-19 pandemic.

**Methods:** A child was admitted to our clinic (male, 14 years old). He was in contact with a COVID-19 patient 17 days before. Upon admission, the patient complained of a rise in body temperature to 40° C, abdominal pain, vomiting, and diarrhea. Hemorrhagic rash on the skin of the upper and lower extremities, hyperemia of the mucous membrane of the lips and tongue, arterial hypotension were found. Hospitalized at ICU. In laboratory tests: WBC 3.42 × 10^9^/l, RBC 4 × 10^12^/l, HB 111 g/l, HTC 31, PLT 31 × 10^9^/l, CRP 283 mg/l, PCT 6.66, D-dimer 9.2, LDG 194 U/l, ferritin 989 mcg/l, ALT 54 U/l, GGT 79 IU/l, albumin 32 g/l; proteinuria 0.75 g\l, hematuria. Diagnosis: MIS-C associated with COVID-19.

**Results:** Prescribed: Meropenem 20 mg/kg/d, methylprednisolone 2 mg/kg/d. After 8 h—septic shock. 0.3 μg/kg/min norepinephrine was started. ECG—a violation of repolarization with ST elevation up to 0.3 mm. Echocardiography—a decrease in the left ventricular ejection fraction to 47%, pericardial effusion. Ultrasound examination of the abdominal cavity: hepatosplenomegaly. Dobutamine 3 μg/kg/min was added to the therapy. An increase in PCT up to 19.8 was found. IV IgG 2 g/kg was added to the therapy. On the 3rd day of therapy, regression of all symptoms was obtained. On the 8th day, the child was transferred from the ICU to the pediatric department. On the 12th day he was discharged home.

**Conclusions:** Thus, the timely diagnosis of MIS-C associated with COVID-19 and the appointment of intensive therapy with the inclusion of methylprednisolone and IV IgG allows achieving a positive result in the shortest possible time.

**Consent to Publish**: Written informed consent was obtained from the next of kin.

## P093

### Impact of COVID-19 on critical care nosocomial infection rates

#### S Helyar, D Hadfield, K O’Reilly, J Smith, P Hopkins

##### King’s College Hospital, ACET Research Team, London, UK

*Critical Care* 2022, **26(Suppl 1):** P093

**Introduction:** Critical care nosocomial infection (CCNI) increases risk of patient mortality and morbidity [1,2]. The impact of the Coronavirus 19 (2019-nCoV) pandemic on CCNI in terms of increased strain and infection control measures, is uncertain. Departmental strain has the potential to confound impact of infection control measures aimed to reduce CCNI incidence. This study will describe the impact of 2019-nCoV on non-COVID CCNI incidence and mortality.

**Methods:** A retrospective cohort study of adult patients admitted to critical care in one Central London teaching hospital. CCNI incidence, (diagnosed ≥ 48 h post critical care admission), was compared between pre (Jan 2019–Feb 2020) and peak COVID (Mar 2020-Jun 2020).

**Results:** Of 2,266 patients, 1788 were admitted pre and 478 peak COVID. Mean age was 57.2 years and 56.1 years pre and peak COVID respectively, with 35.5% and 37.4% of patients, female. There was a significant increase in rate of total CCNI incidence (1.6% to 3.6%) in the pre and peak period respectively. There was a significant increase in rate of incidence of gram negative bacterium and *C.* *diff*, but not in gram positive bacterium, MRSA, VRE and fungus. The increase in rate of peak (23.5%) compared to pre COVID (13.5%) CCNI non-COVID mortality, was not significant (Table 1).

**Conclusions:** Increased infection control measures did not protect against non-COVID CCNI and mortality across all infection types. Increased strain is likely to confound additional infection control measures and resulted in excess patient non-COVID CCNI and mortality, secondary to the pandemic. Greater emphasis is needed to protect other patients from expected CCNIs.

**References**

1. https://www.ecdc.europa.eu/sites/default/files/documents/surveillance-report-HAI-Net-ICU-mortality-2008-2012.pdf Accessed 27/1/22
2. Dasgupta S et al. Indian J Crit Care Med 19:14–20, 2015

**Table 1 (abstract P093):** Rate of CCNI incidence and mortality

CCNI	Pre COVID	Peak COVID	Non-COVID CCNI mortality	
All gram -ve	4.1%	4.1%	Pre COVID	Post COVID
All gram +ve	4.1%	8.6%	13.7%	23.5%
MRSA	1.4%	1.3%	*Significant at 0.05 level	
VRE	2.0%	3.1%		
*C.* Diff*	0.4%	3.6%		
Fungus	0.2%	0.7%		
All CCNI*	1.6%	3.6%		

## P094

### Culture positivity is a strong prognostic indicator of in-hospital mortality for COVID-19 patients with sepsis

#### MJP Patton^1^, AG Gagger^2^, NE Erdmann^3^, CH Orihuela^4^, KH Harrod^5^, MM Might^1^

##### ^1^University of Alabama at Birmingham, Hugh Kaul Precision Medicine Institute, Birmingham, USA, ^2^University of Alabama at Birmingham, Department of Medicine, Division of Pulmonary, Allergy and Critical Care and Program in Protease and Matrix Biology, Birmingham, USA, ^3^University of Alabama at Birmingham, Department of Medicine, Division of Infectious Diseases, Birmingham, USA, ^4^University of Alabama at Birmingham, Microbiology, Birmingham, USA, ^5^University of Alabama at Birmingham, Department of Anesthesiology and Perioperative Medicine, Birmingham, USA

*Critical Care* 2022, **26(Suppl 1):** P094

**Introduction:** Despite numerous clinical scoring systems, outcome modeling for COVID-19 patients with sepsis remains poor. To address this deficit, we assessed the impact of culture positivity on in-hospital mortality for COVID-19 patients with sepsis. We report that culture positive sepsis derived from blood, bronchoalveolar lavage (BAL), or cerebrospinal fluid (CSF) is a stronger prognostic indicator of in-hospital mortality for COVID-19 patients than the Sequential Organ Failure Score (SOFA). These results support inclusion of culture status in future clinical scoring systems.

**Methods:** The cohort was defined by inpatients from 03/20 to 09/21 with a COVID-19 + test (PCR, rapid-antigen, antibody) and septic event (n = 792) as defined by Sepsis-3 guidelines [1]. Each patient's worst SOFA score was computed during their suspected infection window (defined as 24 h prior to and 48 h after the first antibiotic administration or body-fluid culture taken). Study groups included culture positive (n = 478) and culture negative (n = 314) sepsis patients. Charlson comorbidity scores for each patient were calculated prior admission. Positive predictors of in-hospital mortality were assessed with multivariate logistic regression and evaluated for statistical significance using the CAR-ANOVA Type-III test with Bonferroni method.

**Results:** Multivariate logistic regression analysis showed that culture positivity had the greatest adjusted odds ratio (OR: 3.19, 95% CI: 2.09–4.98, *p* < 0.001, corr. *p* < 0.001), compared to worst SOFA score (OR: 1.91, 95% CI: 1.61–2.27, *p* < 0.001, corr. *p* < 0.001), patient age (OR: 1.46, 95% CI: 1.20–1.80, *p* < 0.001, corr. *p* < 0.001), male sex (OR: 1.67, 95% CI: 1.15–2.42, *p* < 0.006, corr. *p* = NS) and comorbidity score (OR: 1.02, 95% CI: 0.84–1.22, *p* = NS, corr. *p* = NS) (Fig. 1).

**Conclusions:** Culture positivity is a strong prognostic indicator of in-hospital mortality for COVID-19 sepsis patients and warrants investigation as a candidate variable for future clinical outcome algorithms.

**Reference**

1. Singer et al. JAMA 315:801–10, 2016

**Fig. 1 (abstract P094):**
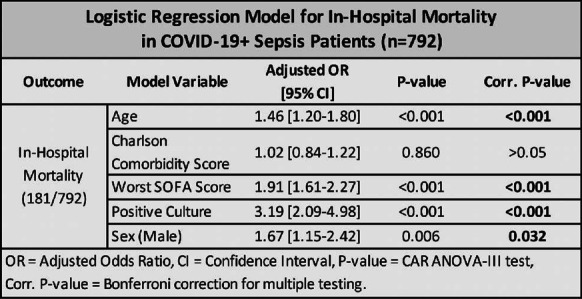
Multivariate logistic regression reveals culture positivity as strongest indicator of in-hospital mortality for COVID-19 patients with sepsis

## P095

### Organising pneumonia due to COVID-19 and its impact on prognosis

#### M Maneira Sousa^1^, M Adao-Serrano^1^, B Rodrigues^2^, S M Fernandes^1^, JM Ribeiro^1^

##### ^1^Centro Hospitalar Universitário Lisboa Norte, Serviço Medicina Intensiva, Lisbon, Portugal, ^2^Faculdade de Medicina de Lisboa, Universidade de Lisboa, Lisbon, Portugal

*Critical Care* 2022, **26(Suppl 1):** P095

**Introduction:** Organising pneumonia (OP) diagnosis is histological, however may be inferred by CT pattern [1, 2]. OP due to COVID-19 has been reported but its role remains unknown.

**Methods:** A single-centre, ethical commission approved, retrospective study was conducted in a tertiary university hospital. Data was collected from patients admitted to ICU with severe COVID-19 between March 2020 and February 2021. OP was defined according to CT chest findings. OP patients were treated with 1 mg/kg/day methylprednisolone as per our protocol. Data was analysed using STATA 15.1.

**Results:** We included 338 patients admitted due to COVID-19 pneumonia, mainly male (68%) with mean age 65.0 ± 13.1 years, 71% underwent invasive mechanical ventilation (IMV) for a median time of 13 days and 84% received corticosteroid treatment, 107 dexamethasone only, the remainder methylprednisolone. 126 patients (37%) featured CT compatible with OP. There were no differences between OP and non-OP regarding age, gender, SAPSII or comorbidities. Although patients with OP more frequently underwent IMV (*p* < 0.01), time from symptoms until IMV was longer (10.1 ± 6.1 in C vs 11.9 ± 6.1 days, *p* = 0.02). Interestingly, duration of IMV and length of stay (LOS) were increased in the OP group (24.5 ± 20.7 vs 14.2 ± 13.9 days, *p* < 0.001; LOS: 28.2 ± 27.6 vs 14.4 ± 15.6 *p* < 0.001), although no difference in ICU (30% vs 29% in OP) or hospital mortality ( 42% vs 53% in OP, *p* = 0.126) was observed. Not surprisingly, delirium (22 vs 36%, *p* = 0.01), ICU acquired weakness (20 vs 43%, *p* < 0.01) and nosocomial infections (41vs 69%, *p* < 0.01) were more frequent in OP patients. Of note, 87% versus 45% of C patients were still on corticosteroids at the time of ICU discharge.

**Conclusions:** High prevalence of OP was demonstrated in this severe COVID cohort associated with longer IMV time but not a significant increase in mortality. More data is required to determine adequate treatment and impact on prognosis.

**References**

1. Kligerman SJ et al. Radiographics 33:1951–75, 2013
2. Drakopangiotakis F et al. Am J Med Sci 335:34–9, 2018

## P096

### Characteristics and outcomes of critical COVID patients in the intensive care unit

#### J Rua^1^, AS Alves^1^, D Viana^1^, D Roriz^2^, H Santos^3^, AL Dias^4^, I Militão^3^, JM Maia^3^, N Barros^3^, F Esteves^3^

##### ^1^Centro Hospitalar Trás-os-Montes e Alto Douro, Internal Medicine, Vila Real, Portugal, ^2^Centro Hospitalar Trás-os-Montes e Alto Douro, Anesthesiology, Vila Real, Portugal, ^3^Centro Hospitalar Trás-os-Montes e Alto Douro, Intensive Care, Vila Real, Portugal, ^4^Centro Hospitalar de São João, Anesthesiology, Porto, Portugal

*Critical Care* 2022, **26(Suppl 1):** P096

**Introduction:** This study aimed to determine the mortality and morbidity of COVID-19 patients in an intensive care unit (ICU) until hospital discharge, and explore the factors that influence in‐ICU and in-hospital mortality rates.

**Methods:** Single center retrospective cohort regarding COVID-19 critical patients in a tertiary hospital ICU, from September/20 to June/21. Demographic data, clinical characteristics, admission SOFA score, frailty score (FS) and clinical management were analyzed.

**Results:** We included 159 consecutive COVID-19 critical patients. The median (IQR) age was 66(57–72); 101(63.5%) were male. A total of 126 (79.2%) patients received hospital discharge, ICU-mortality rate was 18.9%(30 deaths). The median (IQR) ICU length of stay was 12 days (6–20) and in-hospital stay was 21(13–35), and no significant differences were found in ICU and in-hospital length of stay between survivors and non-survivors. At admission to the ICU total SOFA score was 4(3–7). In univariate analysis, increased age, higher admission SOFA score, acute kidney injury and acute neurologic disfunction at admission were significantly associated with increased hazard of mortality. The need for mechanical ventilation were associated with higher risk of ICU and in-hospital mortality. Previous comorbidities (hypertension, diabetes, obesity, heart failure, COPD, renal, hepatic, oncologic or immunosuppression) or the FS were not significantly associated with in-hospital mortality. None of the COVID-19 pharmacologic treatments (remdesivir, steroids and tocilizumab) were significantly associated with in-hospital mortality. In a multivariable analysis with in-hospital death as the dependent variable, a 10 year increase in age was associated with a mortality OR of 2.9 (95 CI:1.5–5.5)( *p* = 0.002) and the development of shock during ICU stay was associated with a mortality OR of 8.8 (95 CI:1.5 to 53.3).

**Conclusions:** In this cohort, only age and the development of shock during ICU stay were independently associated with higher risk of in-hospital death.

## P097

### The usage of the immunosuppressant agents and secondary infections in patients with COVID-19 in intensive care unit: a retrospective study

#### ZT Sarikaya^1^, B Gucyetmez^2^, F Tuzuner^3^, O Dincer^4^, C Sahan^5^, L Telci^6^, IO Akinci^7^, C COVID-19 Study Group^8^

##### ^1^Acıbadem Mehmet Ali Aydınlar University School of Medicine, Department of Anesthesiology and Reanimation, Istanbul, Turkey, ^2^Acıbadem Mehmet Ali Aydınlar University School of Medicine, Istanbul, Turkey, ^3^General Intensive Care Unit, Acıbadem Taksim Hospital, Istanbul, Turkey, Istanbul, Turkey, ^4^General Intensive Care Unit, Acıbadem Atakent Hospital, Istanbul, Turkey, ^5^General Intensive Care Unit, Acıbadem Maslak Hospital, Istanbul, Turkey, ^6^General Intensive Care Unit, Acıbadem International Hospital, Istanbul, Turkey, ^7^General Intensive Care Unit, Acıbadem Altunizade Hospital, Istanbul, Turkey, ^8^Acibadem Health Group, Acibadem Health Group, Istanbul, Turkey

*Critical Care* 2022, **26(Suppl 1):** P097

**Introduction:** It’s known that immunosuppressant agents such as pulse methylprednisolone (PMP), dexamethasone (DXM) and interleukin-blockers (IL-B) are used in COVID-19 [1–3]. The aim of this study is to investigate the effect of these immunosuppressant agents on secondary infections in patients with COVID-19 in intensive care units (ICU).

**Methods:** This study was retrospectively designed and all data between March 2020 and October 2021 of six tertiary ICU was evaluated. All patients were divided by three groups as Group I (GI, no immunosuppressant or MP ≤ 1.0 mg/kg), Group II (GII, PMP and/or DXM) and Group III (GIII, only IL-B and PMP and/or DXM). Demographic data, PaO_2_/FiO_2_ (P/F) ratio, C-reactive protein (CRP) and procalcitonin, hemogram parameters, ferritin and d-dimer, culture results and outcomes were recorded. For comparison between GI-GII and GI-GIII, propensity score matching (PSM) was used by matching 14 parameters [age, gender, BMI, CCI, APACHE II, P/F ratio, CRP, procalcitonin, hemogram parameters, ferritin, d-dimer and invasive mechanical ventilation (IMV) requirements].

**Results:** 412 ICU patients were included in the study (GI = 118, GII = 184, GIII = 110). Mortality rates were 27.1%, 39.7% and 55.5% respectively. After PSM, in GII and GIII, the number of ( +) tracheal cultures, ( +) bloodstream cultures, detected different microorganisms during ICU period, neuropathy, tracheotomized patients, duration of IMV and length of ICU stay were significantly higher than GI. Mortality rate and ( +) CMV-DNA-PCR were similar in GI and GII whereas they were significantly higher in GIII than GI.

**Conclusions:** The usage of immunosuppressant agents in COVID-19 causes increased secondary infections. Moreover, increased secondary infections appear as a reason for prolonged ICU stay and duration of IMV, and also, increased mortality.

**References**

1. Edalatifvard M et al. Eur Respir J 56:2,002,808, 2020
2. Horby P et al. N Engl J Med 8:693–704, 2021
3. Biran N et al. Lancet Rheumatol 2:e603-12, 2020

## P098

### Prediction model for outcomes following tocilizumab treatment for severe COVID-19 pneumonia.

#### K Singla^1^, G Puri^1^, S Niyogi^1^, V Mahajan^1^, K Kajal^1^, A Bhalla^2^

##### ^1^Post Graduate Institute of Medical Education and Research, Department of Anaesthesia and Intensive Care, Chandigarh, India, ^2^Post Graduate Institute of Medical Education and Research, Chandigarh, India

*Critical Care* 2022, **26(Suppl 1):** P098

**Introduction:** The cause of respiratory distress by the novel corona virus is an acute hyper inflammatory “cytokine storm”. Besides glucocorticoids, tocilizumab, a recombinant monoclonal antibody, directed against the IL-6 receptor, has been used as a treatment modality with variable results [1]. Factors affecting poor response to tocilizumab remain unrecognized. We report a model to predict worse outcomes among patients with severe COVID-19 pneumonia treated with tocilizumab.

**Methods:** In this retrospective study, patients with severe COVID 19 pneumonia admitted to the intensive care unit of our hospital who received Inj. tocilizumab besides the standard treatment between July 2020 to July 2021, were included. Electronic records of such patients were accessed and demographic, biochemical and outcome measures were recorded. Patients were divided into survivor cohort and mortality cohort. To predict mortality as an outcome, a multivariate logistic regression model was constructed.

**Results:** Total of 101 patients were included, 71 in survival cohort and 30 in mortality cohort. Lactate dehydrogenase (LDH), neutrophil to lymphocyte ratio (NL ratio), creatine kinase myocardial band (CKMB) and partial pressure of oxygen to fraction of inspired oxygen ratio (PFR) on day of drug administration differed significantly among the two cohorts after correction for multiple comparison. However, on multivariable logistic regression analysis, a model incorporating LDH, NL ratio, pro-brain natriuretic peptide levels (ProBNP) and PFR best predicted mortality (Fig. 1). A nomogram was also created to estimate probability of mortality using the model parameters.

**Conclusions:** LDH, ProBNP, NL ratio and PFR at Tocilizumab administration are independently associated with mortality. A model incorporating the combination of these parameters at admission can predict mortality among patients with severe COVID-19 pneumonia with good accuracy.

**Reference**

1. Morrison AR et al. J Autoimmun 114:102,512, 2020.

**Fig. 1 (abstract P098):**
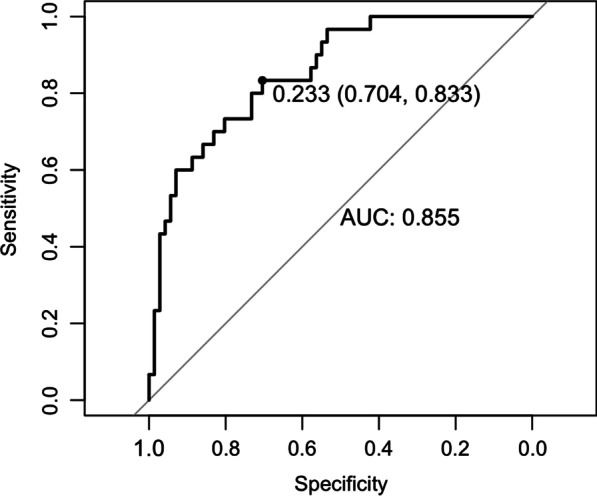
Area under receiver operator characteristics curves for the model

## P099

### Early dexamethasone treatment does not alter the development of pulmonary fibroproliferation in critically ill COVID-19 patients

#### EJ Kooistra^1^, AE Van Herwaarden^2^, J Gerretsen^3^, RL Smeets^2^, S Van der Velde^3^, MJW Van den Berg^3^, JG Van der Hoeven^3^, M Kox^3^, P Pickkers^3^

##### ^1^Radboudumc, intensive Care, Nijmegen, Netherlands, ^2^Radboudumc, Department of Laboratory Medicine, Nijmegen, Netherlands, ^3^Radboudumc, Department of Intensive Care Medicine, Nijmegen, Netherlands

*Critical Care* 2022, **26(Suppl 1):** P099

**Introduction:** A subgroup of critically ill COVID-19 patients develops pulmonary fibroproliferation (PF), which is associated with worse outcomes. We explored the kinetics of fibrosis markers and ventilatory parameters prior to and following use of steroids to treat suspected PF. Furthermore, we investigated the effects of early dexamethasone (DEXA) treatment, the current standard-of-care for COVID-19, on the incidence and time to development of PF and clinical outcomes.

**Methods:** We included 191 critically ill COVID-19 patients spanning two treatment cohorts: no DEXA treatment (pre-DEXA cohort, n = 67) and dexamethasone treatment as standard-of-care (DEXA cohort, n = 124). Kinetics of circulating fibrosis markers and ventilatory parameters were analyzed in suspected PF patients prior to and following initiation of steroid therapy as well as in patients in whom PF was not suspected. Furthermore, associations between PF and clinical outcomes were explored.

**Results:** Patients with suspected PF exhibited higher circulating fibrosis markers, lower lung compliance and PaO_2_/FiO_2_ ratios, and increased dead space ventilation. Incidence of suspected PF was 28% in the pre-DEXA cohort and 25% in the DEXA cohort (*p* = 0.61), and time to development of suspected PF was also similar between cohorts (16 [12–21] vs. 19 [14–23] days from ICU admission, *p* = 0.11). Time on ventilator, LOS in ICU and mortality were significantly higher in suspected PF patients than in no suspected PF patients, with no differences between the cohorts (Fig. 1).

**Conclusions:** Increased circulating fibrosis markers reflect development of PF in critically ill COVID-19 patients, which is associated with prolonged ICU length of stay and high mortality rates. Introduction of dexamethasone as standard-of-care is not associated with altered incidence of PF or improved clinical outcomes in patients with PF.

**Fig. 1 (abstract P099):**
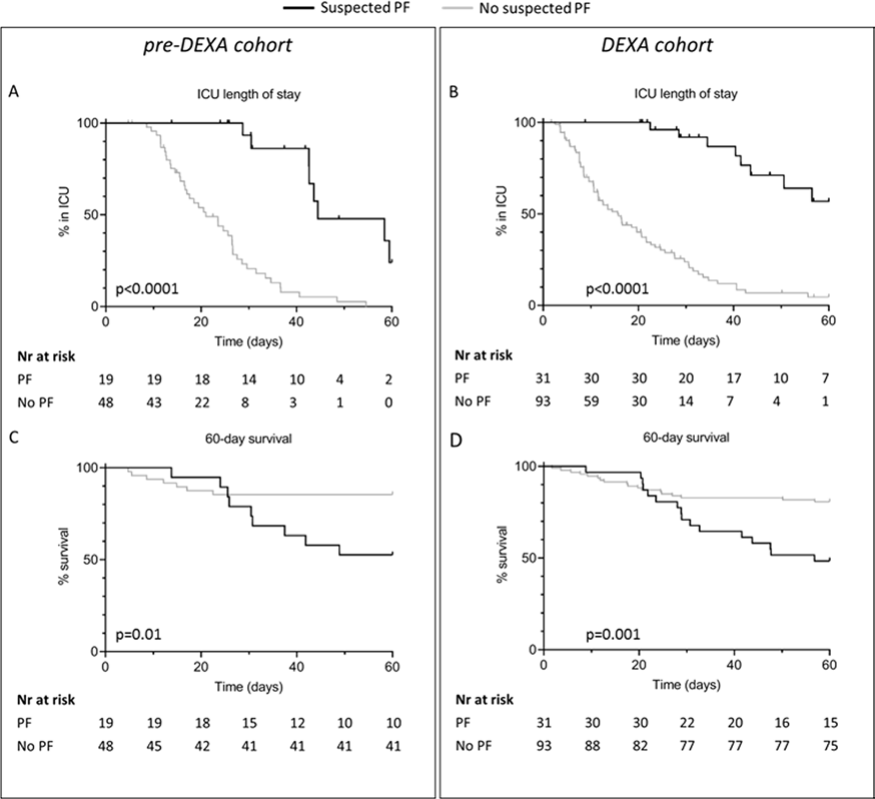
Clinical outcomes. Kaplan-Meier curves of length of stay (LOS) in the intensive care unit (ICU) in (**a**) the pre-DEXA cohort and (**b**) the DEXA cohort, and 60-day hospital mortality in (**c**) the pre-DEXA cohort and (**d**) the DEXA cohort. P values were calculated using log-rank tests. For analysis of 60-day hospital mortality, patients who were discharged alive from the hospital or were still in the ICU or hospital on day 60 were censored at day 60. Numbers at risk are shown below graphs.

## P100

### Management of steroid induced hyperglycaemia in patients critically unwell with COVID-19

#### B Gay, R Mohamed, M Ghannam, K Brown, O Creswell, B Scrace

##### Royal Cornwall Hospital, Intensive Care Unit, Truro, UK

*Critical Care* 2022, **26(Suppl 1):** P100

**Introduction:** The aim of this study was to improve treatment of corticosteroid induced hyperglycaemia in patients critically unwell with COVID-19. Management with high dose steroids reduces mortality and has become standard practice. However, high dose glucocorticoid therapy impairs glucose metabolism in patients already at risk of insulin resistance and impaired insulin production, resulting in increased incidence of hyperglycaemia [1].

**Methods:** A retrospective audit was undertaken, collecting data on steroid use, glycaemic control, and insulin treatment in 100 patients admitted to the Royal Cornwall Hospital Intensive Care Unit with COVID-19. A standard operating procedure (SOP) for the treatment of steroid induced hyperglycaemia was created, based on guidelines from the National Inpatient Diabetes COVID-19 Response Group [1].

**Results:** Of 100 patients, 91 received high dose steroids. The majority (64.8%) experienced glycaemic control issues, defined as one episode of blood sugar > 12 mmol/l. Of the patients treated with 6 mg dexamethasone 52% experienced hyperglycaemia, compared to 71% of those treated with higher steroid doses. There was no significant difference in the highest blood sugar level of either cohort (t54 =  − 0.450, *p* = 0.654). The average time between first episode of hyperglycaemia and commensal of insulin was 76 h. There was a lack of consensus in management of steroid-induced hyperglycaemia—no treatment was administered in 37% of patients. In those who were treated, 19 different combinations of insulin were given. Sliding scale insulin was administered in most patients who experienced no further hyperglycaemia.

**Conclusions:** These results highlight a necessity for consensus management of steroid induced hyperglycaemia. In line with these findings, the devised SOP recommends initial therapy with rapid acting insulin and administration of a sliding scale if hyperglycaemia persists.

**Reference**

1. Rayman G et al. Diabet Med 38:1 e14378, 2020

## P101

### SARS-CoV-2 associated aspergillus and HSV opportunistic infections

#### P Bral^1^, S Thiesen^2^, W Boer^2^, T Fivez^2^, M Vanderlaenen^2^, K Engelen^2^, D Mesotten^2^, X Willaert^2^

##### ^1^ZOL Genk, Anesthesiology, Genk, Belgium, ^2^ZOL Genk, Intensive Care, Genk, Belgium

*Critical Care* 2022, **26(Suppl 1):** P101

**Introduction:** SARS-CoV-2 associated hyperinflammatory syndrome (HIS) is a major cause of ARDS and death. The use of corticosteroids (CS) has shown good effect on both attenuation of HIS and outcome. However, CS use has also been associated with increasing incidence of opportunistic infections in SARS-CoV-2 infected patients. We investigated whether opportunistic infections with Aspergillus species (Asp) or Herpes Simplex Virus (HSV) were more common after implementation of the RECOVERY trial results.

**Methods:** We retrospectively analyzed all patients that were admitted to our ICU for severe COVID-19 induced respiratory failure from 03/20 until 01/21. We identified a total of 186 patients that were dichotomized according to dexamethasone administration upon admission (DEX vs NO-DEX). Patient baseline characteristics, mode of ventilation and survival rates are shown in Fig. 1.

**Results:** Patient baseline characteristics did not differ between both groups (Wilcoxon *p* > 0.05). In the NO-DEX group, 35 patients were treated with high dose CS for perceived HIS, while rescue CS were used in 21 DEX-group patients. A total of 42 patients received one or more broncho-alveolar lavages (BAL): 18 DEX patients and 24 NO-DEX patients. In the DEX group, Asp antigen was detected in 8 BAL (44%), while HSV DNA was found in 11 BAL (61%). In the NO-DEX group, 10 BAL (42%) were positive for Asp antigen and HSV DNA was detected in 19 BAL (79%). Approximately 70% of positive BAL results in the NO-DEX group originated from patients that had received CS.

**Conclusions:** We identified a total of 18 (43%) Aspergillus positive and 30 (71%) HSV DNA positive BAL results. Despite the retrospective analysis inherent bias, our study suggests a strong correlation between the use of CS and Asp and HSV opportunistic infection in SARS-CoV-2 critical ill patients.

**Fig. 1 (abstract P101):**
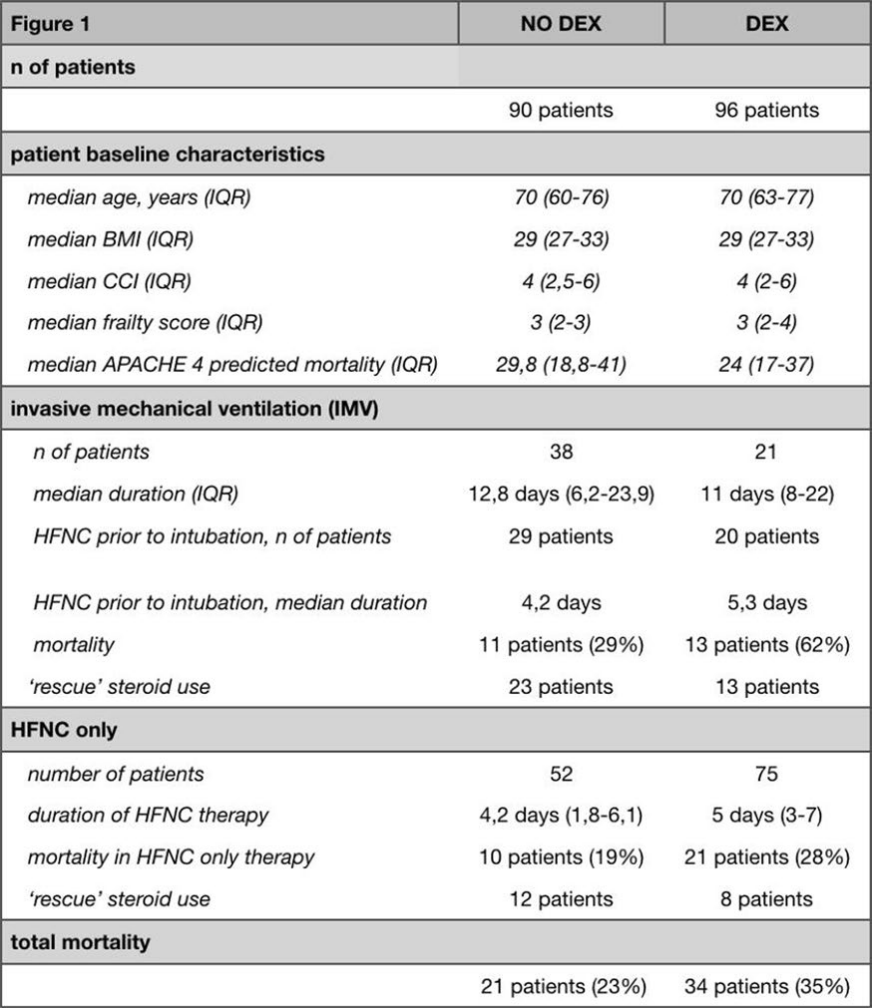
Patient baseline characteristics, mode of ventilation and survival rates

## P102

### Impact of steroid therapy on bacterial and fungal superinfections in patients with severe COVID-19 admitted to ICU.

#### P Giaccaglia^1^, E Casarotta^2^, M Antonini^1^, F Barsotti^1^, G Galli^1^, G Indri^1^, A Martini^1^, E Compagnucci^1^, F Uehelie^1^, A Donati^1^

##### ^1^Università Politecnica delle Marche, Biomedical Sciences and Public Health, Ancona, Italy, ^2^Università Politecnica delle Marche, Biomedical Sciences, Ancona, Italy

*Critical Care* 2022, **26(Suppl 1):** P102

**Introduction:** Patients with severe COVID-19 admitted to ICU have an increased risk of bacterial and fungal superinfections [1,2]. Steroid therapy with dexamethasone is one of the recommended treatments for patients on oxygen therapy. The aim of this study is to evaluate the incidence of superinfections in patients treated with steroids.

**Methods:** We performed an observational retrospective study, including patients with severe COVID-19 admitted to our ICU between March 2020 and February 2021. Data on bacterial and fungal superinfections and steroid therapy were collected.

**Results:** Among the 152 patients enrolled, 82 (53.9%) received steroid therapy before admission to ICU, 50 (32.9%) did not receive steroids, for 20 (13.2%) the steroid treatment was not known. The clinical characteristics of the two groups of patients at admission are presented in Table 1. Comparing patients receiving steroids and those not receiving steroids, the incidence of superinfections due to fungi was respectively 29.6% vs 12.2% (RR 2.41, CI 95%: 1.06–5.50). The incidence of Gram- and Gram + superinfections was respectively 56% vs 55% (RR 1.03, CI 95%: 0.75–1.41) and 54% vs 38% (RR 1.40, CI 95% 0.93–2.09). Among Gram- superinfections, we observed a significant association between steroid therapy and *Acinetobacter* *spp.* superinfection (19.7% in patients on steroids and 6.1% in patients who did not receive steroids, *p* = 0.03). The duration of steroid therapy was directly correlated with the number of superinfections for each patient (Spearman’s rho = 0.34, CI 95% 0.18–0.48, *p* < 0.001).

**Conclusions:** In patients with severe COVID-19 admitted to ICU, steroid therapy seems to be a risk factor for fungal superinfections and associated with *Acinetobacter* *spp.* superinfections. The duration of the steroid therapy is directly correlated to the number of superinfections for each patient.

**References**

1. Langford BJ et al. Clin Microbiol Infect 26:1622–1629, 2020
2. Falcone M et al. J Antimicrob Chemoth 76:1078–1084, 2021

**Table 1 (abstract P102):** Clinical characteristics of the two groups of patients at admission

Clinical features	Patients receiving steroids (n = 82)	Patients not receiving steroids (n = 50)	*p* Value (unpaired t-test or Mann-Whitney U test)
Age, years	67 [59-74]	60.5 [50-68]	< 0.01*
SOFA score	7.14 ± 1.98	7.94 ± 1.69	0.02*
PaO_2_/FiO_2_	107 [75-164]	118 [84.5-193.5]	0.34
Leukocytes, × 10^3^/mm^3^	10.81 [7.93-15.25]	10.00 [6.94-13.08]	0.11
Lymphocytes, × 10^3^/mm^3^	0.50 [0.35-0.76]	0.54 [0.36-0.87]	0.31
Procalcitonin, ng/ml	0.27 [0.11-0.7]	0.55 [0.19-1.59]	0.02 *

## P103

### Acquired infections in intensive care unit (ICU) COVID-19 patients

#### J Rua^1^, AS Alves^1^, M Contente^2^, J Rodrigues^3^, R Amaral^2^, AL Dias^4^, AV Cristino^2^, JM Maia^2^, N Barros^2^, F Esteves^2^

##### ^1^Centro Hospitalar Trás-os-Montes e Alto Douro, Internal Medicine, Vila Real, Portugal, ^2^Centro Hospitalar Trás-os-Montes e Alto Douro, Intensive Care, Vila Real, Portugal, ^3^Hospital Sousa Martins—Unidade Local de Saúde de Guarda, Internal Medicine, Guarda, Portugal, ^4^Centro Hospitalar de São João, Anesthesiology, Porto, Portugal

*Critical Care* 2022, **26(Suppl 1):** P103

**Introduction:** Patients with COVID-19 admitted to the ICU are at high risk of developing infectious complications during their ICU stay. Data on acquired(AI) in Portuguese critical COVID-19 patients are scarce. The aim of this study was to investigate the characteristics and risk factors for AI in critical patients with COVID-19 pneumonia admitted to the ICU.

**Methods:** Retrospective cohort of patients with COVID-19 pneumonia admitted to an ICU in a tertiary hospital, between September 2020 and June 2021. AI considered were ventilator-associated pneumonia (VAP) or tracheobronchitis (VAT), bacteremia, CVC associated infections, urinary tract infections and soft skin tissue infections. Baseline characteristics, 3-months previous antibiotic (ATB) exposure, ATB treatment at ICU-admission and clinical management of COVID-19 pneumonia were analyzed.

**Results:** Of the 159 patients included, with a median (IQR) age of 66 (57–72) and 63.5% males, 14 (8.8%) had no known comorbidities. A total of 63 patients(39.6%) developed AI: 45(71.4%) VAP, 20(33.3%) VAT, 28 (45.2%) UTI, 6 (9.5%) CVC associated infections and 3(4.8%) soft skin tissue infections. In univariate analysis, both SOFA score at admission (*p* < 0.001), acute cardiovascular (*p* = 0.003) and neurologic (*p* = 0.006) disfunction at ICU admission were associated with the development of AI. AI were also correlated to need of tracheostomy(*p* < 0.001), development of delirium (*p* < 0.001) or shock (*p* < 0.001); and with longer ICU and in-hospital stay (*p* < 0.001) and ICU and hospital mortality (*p* = 0.011 and *p* = 0.011, respectively). None of the COVID-19 pharmacologic treatments considered (remdesivir, steroids and tocilizumab), neither different regimens of ATB therapy at ICU admission were significantly associated with AI.

**Conclusions:** In this cohort, almost 40% of the patients developed AI, that was associated with 4 times higher hazard of needing mechanical ventilation and higher rate of adverse events such as delirium, shock during in-ICU stay and longer length of ICU and in-hospital stay.

## P104

### The rate of secondary infections and diagnostic challenges in critically ill patients with COVID-19

#### G McCreath^1^, MRS Ralston^2^, AJ Roe^1^, MJ Watson^3^, MAB Sim^3^

##### ^1^Institute of Infection, Immunity & Inflammation, University of Glasgow, Glasgow, UK, ^2^Institute of Cardiovascular & Medical Science, University of Glasgow, Glasgow, UK, ^3^School of Medicine, Dentistry & Nursing, College of Medical, Veterinary & Life Sciences, University of Glasgow, Glasgow, UK

*Critical Care* 2022, **26(Suppl 1):** P104

**Introduction:** Patients admitted to intensive care with severe SARS-CoV-2 infection frequently present with sepsis. These patients are at a higher risk of developing a secondary infection, but this can be difficult to distinguish from the primary viral infection [1]. Liberal use of broad-spectrum antibiotics can lead to proliferation of antimicrobial resistant organisms. We aimed to identify the rates of bacterial secondary infections and antimicrobial usage in critically ill COVID-19 patients.

**Methods:** We performed a retrospective review of case records for patients admitted to critical care from three hospitals in the Greater Glasgow and Clyde Health Board, Scotland. Patients with confirmed SARS-CoV-2 infection admitted to high dependency or intensive care were eligible. We collected data on background patient demographics, comorbidities, admission SOFA scores, antimicrobial usage, and positive microbiological cultures from within a 10-day period. Cultured organisms that were unlikely to be clinically significant were excluded.

**Results:** Records for 105 patients admitted between December 2020 and September 2021 were reviewed. At admission to critical care, 100% met the criteria for sepsis in accordance with the Sepsis-3 International Consensus definition, and 33% went on to develop septic shock [2]. The mortality rate was 34%. All patients received corticosteroids, and 74% were treated with an IL-6 receptor antagonist. Half of the patients had at least one clinically significant positive microbial culture, however a much higher proportion (73%) were treated with antibiotics.

**Conclusions:** Secondary infections can be difficult to diagnose in the presence of severe COVID-19 disease, with a disproportionally high use of antibiotics relative to positive cultures. Additional diagnostic tools would be useful in this patient population to aid in antimicrobial stewardship.

**References**

1. Russell CD et al. Lancet Microbe 2:e354-e365, 2021.
2. Singer M et al. JAMA 315:801–810, 2016.

## P105

### Evaluation of the utility of bioelectrical impedance analysis as a bedside tool to monitor the volume of distribution of hydrophilic antibiotics in critically ill patients

#### B Mertens^1^, E Simons^2^, Y Debaveye^3^, J Wauters^4^, I Spriet^1^, M Gijsen^1^

##### ^1^KU Leuven, Department of Pharmaceutical and Pharmacological Sciences, Leuven, Belgium, ^2^University Hospitals Leuven, Pharmacy Department, Leuven, Belgium, ^3^KU Leuven, Department of Cellular and Molecular Medicine, Leuven, Belgium, ^4^KU Leuven, Department of Microbiology, Immunology and Transplantation, Leuven, Belgium

*Critical Care* 2022, **26(Suppl 1):** P105

**Introduction:** Increased distribution volume (V_d_) of hydrophilic antibiotics may lead to inadequate antimicrobial exposure and potential therapeutic failure in critically ill patients. Covariates accurately describing (variability in) V_d_ are lacking. Bioelectrical impedance analysis (BIA) has been proposed as a promising tool to monitor V_d_ in critical illness. Therefore, we aimed to evaluate correlations between BIA-derived fluid estimates and V_d_ of piperacillin-tazobactam and vancomycin in critically ill patients.

**Methods:** A prospective observational study was conducted in adult patients treated with piperacillin-tazobactam or vancomycin at the intensive care units of UZ Leuven. BIA was performed on consecutive antibiotic treatment days, in conjunction with blood sampling to calculate V_d_ (one-compartment analysis). Absolute and differential (i.e. changes) values of consecutive BIA-derived fluid estimates and V_d_ were determined. Correlations between fluid estimates and V_d_ were expressed as Pearson correlation coefficients.

**Results:** We included 21 patients treated with piperacillin-tazobactam and 7 patients treated with vancomycin. Absolute (n = 80) and differential (n = 52) values of fluid estimates and V_d_ are summarized in Table 1. Overall, correlations were weak and non-significant. A significant correlation with V_d_ was only observed for absolute intracellular water volumes in patients treated with vancomycin (r = 0.44; *p* = 0.03). No significant correlations were found for changes in fluid estimates and V_d_ of the studied antibiotics.

**Conclusions:** This study failed to demonstrate clinically relevant correlations between BIA-derived fluid estimates and V_d_ of piperacillin-tazobactam and vancomycin, expressed as absolute and differential values. Consequently, we were not able to corroborate the previous suggestion that V_d_ of hydrophilic antibiotics can be estimated with BIA. Further research on the utility of covariates to reliably monitor V_d_ in critically ill patients should be encouraged.

**Table 1 (abstract P105):** ∆ differential value, i.e. change between two consecutive inclusion days

	Piperacillin-tazobactam: median [IQR] (n = 55)	Piperacillin-tazobactam: median [IQR] ∆ (n = 34)	Vancomycin: median [IQR] (n = 25)	Vancomycin: median [IQR] ∆ (n = 18)
TBW (l)	42.3 [36.9;46.7]	−0.4 [−2.1;0.7]	44.3 [37.6;48.7]	−1.2 [−3.5;0.8]
ECW (l)	20.4 [18.4;23.5]	−0.1 [−1.1;0.3]	20.2 [19.4;25.7]	−0.3 [−0.9;0.6]
ICW (l)	21.8 [17.9;23.8]	−0.3 [−1.1;0.6]	23.6* [17.7;27.1]	−0.3 [−1.7;0.1]
FFMH (%)	78.2 [74.0;80.5]	−0.4 [−1.5;0.6]	77.2 [76.3;82.6]	−0.4 [−2.7;1.8]
Excess fluid (l)	2.8 [0.2;6.2]	−0.2 [−1.1;0.2]	3.3 [1.9;6.7]	−0.3 [−1.3;1.4]
V_d_ (l)	25.5 [16.6;40.1]	−1.1 [−10.7;17.0]	82.5 [46.2;114.5]	−7.0 [−26.3;16.4]
V_d_ (l/kg)	0.35 [0.25;0.59]	−0.02 [−0.19;0.23]	1.10 [0.70;1.55]	−0.09 [−0.36;0.22]

*statistically significant (*p* < 0.05) correlation with V_d_; BIA: bioelectrical impedance analysis; ECW: extracellular water; FFMH: fat-free mass hydration; ICW: intracellular water; IQR: interquartile range; TBW: total body water; V_d_: volume of distribution

## P106

### The burden of ventilator-associated pneumonia and its impact in hospital resources in ICU patients: a national, multicenter, retrospective study (EVAP-PT study)

#### J Duarte^1^, P Mergulhao^2^, J Gonçalves Pereira^3^, L Pássaro^1^, F Froes^4^

##### ^1^MSD Portugal, Medical Affairs, Paço de Arcos, Portugal, ^2^Hospital Lusíadas, Intensive Medicine, Hospital Lusíadas, Porto, Porto, Portugal, ^3^Hospital Vila Franca de Xira, Intensive Medicine, Hospital Vila Franca de Xira, Vila Franca de Xira, Portugal, ^4^Entro Hospitalar Universitário Lisboa Norte, Hospital Pulido Valente, Lisboa, Portugal

*Critical Care* 2022, **26(Suppl 1):** P106

**Introduction:** This study aims to evaluate the impact of ventilator-acquired pneumonias (VAP) on the patient and healthcare system in Portuguese ICUs. VAP is associated to increased patients’ length of stay (LOS), as well as prolonged periods of mechanical ventilation(MV), responsible for a fraction of antibiotic prescriptions. This study compared demographic characteristics of MV patients who developed VAP versus patients who did not.

**Methods:** This study included adult subjects, admitted to ICU for any reason between July 1, 2016 and December 31, 2017. Only subjects who started MV for at least 48 h during their ICU admission were included. VAP patients and MV patients who did not develop VAP (MVnon-VAP) were identified retrospectively and randomly included in a 1:1 ratio. Demographic information, VAP-related characteristics, and baseline severity scores were captured from medical records.

**Results:** 197 VAP patients and 197 MVnon-VAP patients were included. A larger number of patients (46.7%) in the MV non-VAP group were aged 70 years or older, *p* = 0.021. The main reasons for ICU admission were different between groups. Differences were also observed in severity scores between VAP and non-VAP patients (SAPSII, *p* = 0.048) (Fig. 1). On healthcare resource utilization, statistical differences (*p* < 0.001) were observed between VAP vs MVNon-VAP patients in hospital (61 vs 35.9 days, respectively) and ICU LOS (27.5 vs 11 days, respectively). Also, MV duration was always higher for VAP patients (20.7 vs 8 days, respectively, *p* < 0.001).

**Conclusions:** Among MV patients, VAP group experienced more hospitalizations and longer LOS, supporting a large healthcare resource utilization required by VAP which might lead to higher admission costs. Several limitations must be considered in this retrospective study including the different criteria associated to VAP diagnosis in each center. Nevertheless, these data reinforce the importance of characterizing adequately hospital infections, for an improved management and optimize health resources.

**Fig. 1 (abstract P106):**
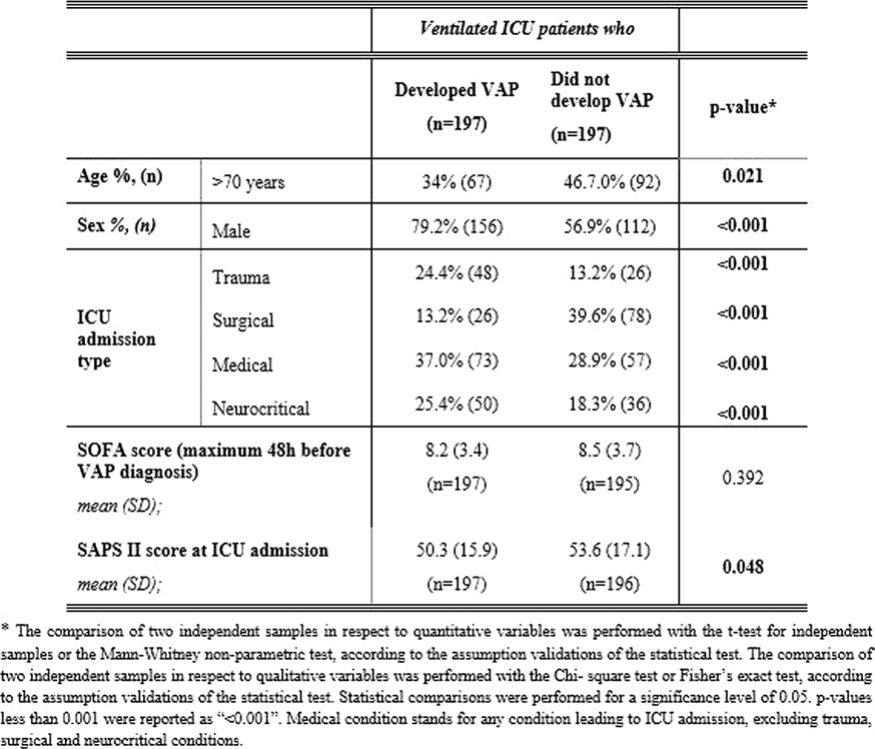
Results

## P107

### Feasibility, usefulness and clinical results in the implementation of a CAPA active surveillance protocol.

#### MC Soriano, M López-Olivencia, J Higuera Lucas, G Narváez Chávez, L López Vergara, I Pozuelo Echegaray, R Marín Ráez, S García Plaza, S Sáez Noguero, R De Pablo

##### Hospital Universitario Ramón y Cajal, Intensive Care Department, Madrid, Spain

*Critical Care* 2022, **26(Suppl 1):** P107

**Introduction:** Coronavirus disease associated pulmonary aspergillosis (CAPA), can be a devastating complication in patients on mechanical ventilation (MV) and ARDS. Recently, we had implemented an active screening and surveillance protocol, focused on the early detection of CAPA. Here we present our institutional results.

**Methods:** We included all consecutive patients admitted to a polyvalent ICU, with SARS-CoV-2 pneumonia, from Mar 2020 to Jul 2021. The protocol was implemented in Aug 2020. Our surveillance protocol consists of lower respiratory track samples, obtaining by bronchial aspiration, processed with calcofluor staining, once a week. In case of positive results, respiratory or clinical worsening, we collect lower respiratory track samples by bronchoalveolar lavage (BAL). Probable CAPA definition is in accordance with ECMM/ISHAM consensus criteria [1].

**Results:** During the study period, 345 patients were admitted in our ICU with SARS-CoV-2 pneumonia related ARDS. 90% required invasive MV. The mean age was 60 years, 69% were male, with severity scores mean values of SOFA 6.4, APACHE II 16.5, and SAPS II 39.5. 8.7% (n = 30) of the patients met the diagnostic criteria of probable CAPA. 90% with GM index > 1 in BAL. 70% with culture samples and GM assay from BAL were positive. This represents a global incidence of 8.7%. The ICU mortality in patients with probable CAPA was 23.3%. The ICU mortality of patients on MV without CAPA was 15.7% (*p* = 0.3).

**Conclusions:** CAPA is a severe complication and entails significant increase in morbi-mortality. We implemented an active surveillance system in our ICU. Early and timely detection, together with optimal organ support management could have influenced clinical outcomes, lowering the ICU mortality rate. In our sample, this mortality is not statistically significant despite being higher than in the non-CAPA group. It is possible that this difference was not greater, thanks to the proposed active surveillance protocol.

**Reference**

1. Koehler P et al. Lancet Infect Dis 21:e149-e162, 2021.

## P108

### Healthcare-associated infections (HAI) in trauma patients admitted to ICU

#### CD Dominedò^1^, GS Saltelli^1^, BM Mariani^2^, AM Martinotti^1^, EG Cingolani^1^

##### ^1^San Camillo Forlanini Hospital, Department of Shock and Trauma, Rome, Italy, ^2^San Camillo Forlanini Hospital, Microbiology and Virology, Rome, Italy

*Critical Care* 2022, **26(Suppl 1):** P108

**Introduction:** Little is known about HAI in severe trauma patients admitted to ICU [1,2]. We aimed to describe the prevalence of infections in this specific subgroup of patients, the pathogens most frequently isolated, the time from ICU admission to HAI development.

**Methods:** A retrospective cohort study was performed at the II Level Trauma Center of the San Camillo Hospital in Rome. We included adult patients admitted to ICU between June 2019 and June 2020 with severe trauma (Injury Severity Score > 20), with no infection and no antibiotic therapy at admission. Cultures performed during ICU stay were analyzed.

**Results:** A total of 142 patients met the inclusion criteria. 95 patients (66.90%) presented HAI during ICU stay. Demographic characteristics of the infected and no infected group are presented in Table 1. 82 patients (86.3%) presented microbiological isolation in endotracheal aspirates (*Klebsiella* spp 35.36%, *Staphylococcus* spp 34.14%, *Pseudomonas* spp 31.70%), 65 patients (68.4%) in blood cultures (*Staphylococcus* coagulase negative 67.69%, Klebsiella spp 24.61%, *Acinetobacter* spp 10.76%), and 16 patients (16.8%) in physiologically sterile liquid (*Staphylococcus* coagulase negative 43%, *Staphylococcus* *aureus* 25%, fungi 25%). Rectal swab was positive in 20 patients (21%). Mean time from ICU admission to HAI development was 4.75 ± 3 days (5.2 ± 3.3 for respiratory infections, 8.3 ± 5 for blood infections, 4.2 ± 2.7 for sterile liquid infections).

**Conclusions:** Due to its complex pathophysiology and management, trauma is a major risk of HAI. The incidence of HAI, mainly caused by Gram negative pathogens, in severe trauma patients can be high. Knowing ICU ecology and adopting specific preventive measure may help reducing HAI development and improving trauma patients outcome.

**References**

1. Major JS et al. J Intensive Care Soc. 16:193–198, 2015
2. Eguia E et al. Am J Surg. 218:851–857, 2019

**Table 1 (abstract P108):** Demographic characteristics and outcomes in infected and no infected patients

	Total population (n = 142)	Infected group (n = 95)	No infected Group (n = 47)
Male sex n (%)	109 (76.8%)	77 (77.9%)	35 (74.5%)
Age (mean ± DS)	49.4 ± 17.4	50 ± 17.1	48.2 ± 18.2
ISS (mean ± DS)	39.7 ± 14.7	41.8 ± 13.3	35.3 ± 14.3
ICU length of stay (mean ± DS)	16.7 ± 14.4	20.7 ± 15.5	8.6 ± 6.4
ICU mortality n (%)	10 (7%)	8 (8.4%)	2 (4.2%)

## P109

### Comparison of admission versus critical care acquired MRSA, VRE and *C. difficile* on patient mortality, critical care length of stay and costs

#### S Helyar, D Hadfield, K O’Reilly, J Smith, P Hopkins

##### King’s College Hospital, ACET Research Team, London, UK

*Critical Care* 2022, **26(Suppl 1):** P109

**Introduction:** To compare the impact of admission versus critical care acquired MRSA, VRE and *C.* *difficile* on critical care patient mortality, LOS and costs over a 9 year period. Critical care patients are most at risk of nosocomial infection (NI), morbidity and mortality due to underlying co-morbidities, acute disease processes and increased use of interventions [1,2]. However, many patients are at risk of NI pre-admission to the critical care unit which also impact on patient outcomes. Limited data exist on the impact of admission versus critical care acquired NI on patient mortality, length of stay (LOS) and costs.

**Methods:** Retrospective analysis of adult (aged ≥ 18 years critical care patients (stay ≥ 48 h) admitted to one of three critical care units at one Central London hospital between Apr 2011–Jun 2020.

**Results:** 18,864 patients were admitted to critical care during the study period. 40.2% of patients were female, mean patient age was 57.9 years with an overall patient mortality of 15.8%. Testing for MRSA, VRE and *C.* *difficile* occurred in 18,596 (98.5%), 1943 (10.4%) and 4814 (25.5%) of patients respectively. Both pre admission and critical care acquired NI increased patient mortality in VRE and C. Diff as well as length of stay and costs across all NI groups (Fig. 1).

**Conclusions:** This study underlines the variable impact of pre-admission versus critical care acquired pathogens on patient outcomes and impact on critical care patient care and costs. This study reinforces the importance of optimal pre-admission and critical care infection control procedures to reduce excess critical care mortality, LOS and cost.

**References**

1. https://www.ecdc.europa.eu/sites/default/files/documents/surveillance-report-HAI-Net-ICU-mortality-2008-2012.pdf Accessed 27/1/22.
2. Dasgupta S et al. Indian J Crit Care Med 19:14–20, 2015.

**Fig. 1 (abstract P109):**
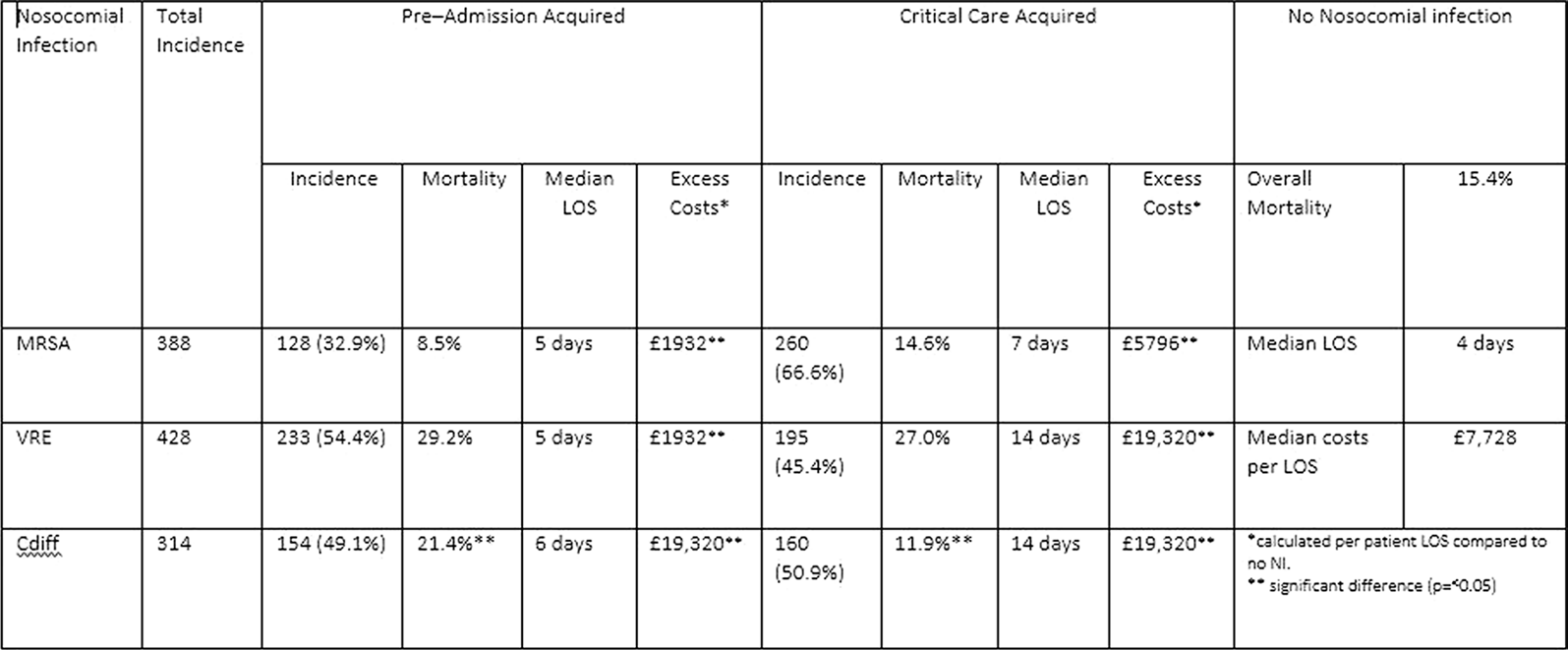
Infection incidence, mortality, length of stay and costs

## P110

### Evaluation of clinical efficacy of fosfomycin in an urban academic medical center

#### H Drone, K Robinson

##### Hennepin Healthcare, Clinical Pharmacy, Minneapolis, USA

*Critical Care* 2022, **26(Suppl 1):** P110

**Introduction:** This study aimed to evaluate the efficacy of fosfomycin for uncomplicated urinary tract infections (UTI) in a single center urban academic medical center. We proposed that the efficacy and sensitivity of fosfomycin has decreased similarly to other first line antibiotics since the publication of the Infectious Diseases Society of America and European Society for Microbiology and Infectious Diseases guidelines in 2011 [1]. Because fosfomycin sensitivities are not routinely performed, results of this clinical outcome based study may guide direction of microbiologic testing in an aim to reduce treatment failure with empiric fosfomycin prescribing.

**Methods:** This retrospective chart review included adult patients prescribed fosfomycin in the inpatient or outpatient setting at Hennepin Healthcare from January 1, 2019 to October 31, 2021. The primary outcomes were return to clinic, hospital, or emergency department within 30 days for ongoing infection and additional antibiotic courses for UTI within 14 days.

**Results:** Eighty four patients with 107 encounters met inclusion criteria. Fosfomycin was prescribed for diagnosis of uncomplicated UTI in 94/107 (87%) of cases and 73/107 (68%) was in relation to a known culture result versus 34/107 (34%) was empirically prescribed. Of cultures collected (n = 103), 47% were ESBL producing organisms and 53% were non-ESBL producing organisms. The 30 day return rate for ongoing symptom management was 23% (25/107) and 21% (23/107) received further antibiotics within 14 days.

**Conclusions:** Fosfomycin may have worsening clinical cure rates when compared to historical data. Fosfomycin sensitivity testing may be warranted in some patient populations and use must be weighed in relation to other first line agents.

**Reference**

1. Gupta K et al. Clin Infect Dis 52: e103-e120, 2011.

## P111

### Multiresistant ESKAPE bacteria in critical care unit: colonization, infection and appropriate empirical antibiotic rate

#### M Muñoz Garach^1^, O Moreno Romero^2^, P Fernandez Morales^2^, MT Cruces Moreno^2^

##### ^1^Hospital Universitario Clínico San Cecilio, Critical Care, Granada, Spain, ^2^Hospital Universitario Clínico San Cecilio, Intensive Care Unit, Granada, Spain

*Critical Care* 2022, **26(Suppl 1):** P111

**Introduction:** Multiresistant bacteria (MRB) increases the patient’s morbimortality rates, particularly in the critically ill. The choice of a correct empirical antibiotic may reduce the impact of these infections and the disseminations in our units.

**Methods:** Analysis of a prospectively database (ENVIN HELICS registry)12 months (year 2018) in our ICU. We registered all + cultures with ESKAPE germs (Enterobacteriae, SARM, Klebsiella, Acinetobacter, Pseudomona, Enterococcus) and divided in 2 categories: "colonization culture" and "suspected infection". From all “suspected infection” registered the empirical antibiotic therapy used. When the microorganism was identified, analysed if the antibiotic was either maintained, changed or added a 2º one. With the antibiogram defined whether the empirical treatment was appropriate or not.

**Results:** A total of 1590 patients admitted to ICU in 2018. 61% male, 49% female. Mean age 57 ± 11. 498 + cultures, 111 (22.2%) with MRB-ESKAPE in 75 different patients. From these, 78 (70.3%) were “colonization cultures” and 33 (29.7%)“suspected infection”. Localization infections: 25 (60%) respiratory, 8 (19%) urine tract, 5 (12%) bacteremia and 4 (9%) other infections. In these suspected infections (33) an empirical antibiotic was initiated. After the identification: 22 (66.66%) no modifications in treatment, 7 (21.21%) the antibiotic was changed and 4 (12.12%) added a 2º one. After antibiogram: 26 (78.78%) first empirical treatment was appropriate, 3 (9%) right at the second election, and 4 (12.12%) none of the antibiotic were correct. ESKAPE % were: 29% Enterobacteria, 25% SARM, 19% Klebsiella, 16% Pseudomona, 11% Acinetobacter/enterococcus.

**Conclusions:** Most frequent ESKAPE bacteria in our unit is the Enterobacteriae. ESKAPE MRB infection rate is 7.8% and the appropriate empirical antibiotic rate were 79%. In half of the 21% of inappropriate empirical antibiotic treatment was due to an unusual early infection with Klebsiella MR in patients with no risk factors for infection or colonization with MRB.

## P112

### Impact of remdesivir in COVID-19 patients under non-invasive ventilation in an intermediate care unit

#### J Rua^1^, AR Nogueira^1^, JE Mateus^1^, AF Costa^2^, A Ribeiro^3^, C Silva^4^, J Trêpa^5^

##### ^1^Centro Hospitalar Universitário de Coimbra, Serviço de Medicina Intensiva, Coimbra, Portugal, ^2^Centro Hospitalar Universitário de Coimbra, Serviço de Pneumologia, Coimbra, Portugal, ^3^Centro Hospitalar Universitário de Coimbra, Serviço de Hematologia Clínica, Coimbra, Portugal, ^4^Centro Hospitalar Universitário de Coimbra, Serviço de Medicina Interna, Coimbra, Portugal, ^5^Centro Hospitalar Universitário de Coimbra, Serviço de Doenças Infecciosas, Coimbra, Portugal

*Critical Care* 2022, **26(Suppl 1):** P112

**Introduction:** COVID-19 has generated enormous difficulties globally due to the high number of critically ill patients and uncertainty of the best therapeutic approach, even after 18 months of pandemic and multiple clinical trials. The antiviral remdesivir (RDV) has shown to reduce time to clinical recovery and, in a subgroup with low flow O2 at time of drug initiation, to reduce mortality by 70% (ACTT-1). Subsequent open-label RCT, Solidarity and Discovery, didn’t confirm these findings. In our unit, a strict protocol was used, including a 5-day cycle of 20 mg dexamethasone and start of HFNC/CPAP when increased work of breathing became noticeable, along with prolonged periods of awake prone position. The use of RDV was a point of significant variability, allowing us to compare outcomes. We describe our unit's experience and RDV impact on patients under non-invasive ventilation (NIV).

**Methods:** Descriptive retrospective study. Data were collected from 202 COVID-19 patients under NIV at our intermediate care unit between September/2020 and March/2021, through medical records in the electronic clinical file. Categorical data are presented as frequency (percentage) and were compared using χ2 -test. Continuous variables were compared using Mann–Whitney U test. Statistical significance was set at *p* < 0.05.

**Results:** Each group consisted of 101 patients, with the group not submitted to RDV being slightly older (mean age 70.5 vs 63 years), more frail (mean CFS 3.5 vs 2.8) and with higher mean SOFA at admission (4.0 vs 3.2). The RDV group had a lower mortality rate (20.8 vs 52.5%; *p* < 0.001), less NIV failure (20.8 vs 50.5%; *p* < 0.001), shorter duration of ventilation in survivors (7.0 vs 8.5 days; *p* = 0.036) and less need for intensive care admission (14.9 vs 23.8%), with favorable impact on mortality (26.6 vs 50%) in this subgroup.

**Conclusions:** In our cohort of patients under NIV, RDV use was associated with lower mortality, less need for IMV and shorter duration of ventilation.

## P113

### A monocentric observational analysis of the use of prophylactic antimicrobial therapy and perioperative cultures during pancreaticoduodenectomy

#### A De Wilde^1^, J Fierens^2^, J De Waele^2^, P Depuydt^2^, F Berrevoet^3^, F Gryspeerdt^3^, L De Bus^2^

##### ^1^University Hospital Ghent, Intensive Care, Ghent, Belgium, ^2^University Hospital Ghent, Intensive Care Unit, Ghent, Belgium, ^3^University Hospital Ghent, General and HPB surgery, Gent, Belgium

*Critical Care* 2022, **26(Suppl 1):** P113

**Introduction:** Postoperative infections (PI) are common after major surgery. To reduce PI after pancreaticoduodenectomy (PD), perioperative antimicrobial therapy (AMT) is often prophylactically administered. At our hospital, amoxicillin-clavulanic acid (AMC) or piperacillin-tazobactam (PTZ) is given in the absence or presence of biliary stenting respectively. The objective of this study is to analyze antimicrobial susceptibility of perioperative biliary cultures in relation to prophylactic AMT and the incidence of PI.

**Methods:** All adult patients undergoing PD in our hospital from 2015 until 2020 were evaluated for inclusion. Analysis included demographics (gender, age, BMI, APACHE and SAPS on admission, median SOFA score), prophylactic AMT details, perioperative biliary cultures and characteristics of PI.

**Results:** Out of 261 patients who underwent PD, 78 (29.9%) had a biliary stent, 183 did not. Perioperative cultures were positive in 71 (91.2%) stent patients and in 36 (19.7%) non-stent patients (*p* < 0.05). In stent patients, 29 (40.9%) cultures were resistant to PTZ. In non-stent patients, 17 (47.2%) cultures were resistant to AMC. *Enterobacterales* and *Enterococci* were most commonly found in both groups. Most frequent PI were intra-abdominal infections and superficial wound infections. More PI were diagnosed in non-stent patients (46 patients, 25.1%) than in stent patients (9 patients, 11.4%). Pathogens identified in PI often differed from perioperative biliary cultures and were resistant to earlier prophylactic treatment (23 non-stent patients (50%), 7 stent patients (77.8%)).

**Conclusions:** This study shows *Enterobacterales* and *Enterococci* as our most common biliary pathogens, independently of the presence of a biliary stent. Therefore if prophylactic AMT in PD would be undertaken, it should preferably be tailored to biliary tract ecology. PI were present in 1 out of 5 patients, more prevalent in non-stent patients and often associated with need for widening AMT, highlighting rigorous culture procurement in PI.

## P114

### Prognostic markers of acute liver failure: a retrospective observational study.

#### M Benlabed^1^, S Benlabed^2^, S Nedjari^3^, R Gaudy^4^, A Ladjouze^3^, S Aissaoui^3^

##### ^1^Lille University, Anesthesiology, Lille, France, ^2^Free University of Brussels, Internal Medicine, Brussels, Belgium, ^3^Algiers University, Anesthesiology, Algiers, Algeria, ^4^Lille University, Internal Medicine, Lille, France

*Critical Care* 2022, **26(Suppl 1):** P114

**Introduction:** Prognostic criteria in patients with acute liver failure (ALF) are an important tool to evaluate the severity and to predict the mortality of this disease. The objective of this study was to analyze the impact of different prognostic markers on the outcome of patients with ALF.

**Methods:** We conducted a retrospective observational study enrolling 40 patients who presented acute liver failure (ALF) at the emergency department of an university hospital from May 2016 to May 2020. The main etiologies of ALF were paracetamol poisoning and Fulminant viral hepatitis. We collected, analyzed and compared the data of 2 groups of 20 patients with ALF: a first group of survivors and a second group of non-survivors. The patients were 35±15 years old without anterior comorbidity. Patients’ baseline characteristics were well matched between groups. In the two groups, we measured and recorded blood lactate level at T0, T12 h and T24 h, arterial ammonia concentration at T0, T24 h and T 48 h, blood fibrinogen level, INR, biological parameters particularly blood sodium and serum creatinine. We registered the requirement of invasive mechanical ventilation (IMV), of renal replacement therapy (RRT), the incidence of grade 3–4 hepatic encephalopathy (HE) and cerebral edema. We calculated SOFA Score at day 1, and day 3.

**Results:** Statistic analysis used Mann–Whitney test and results expressed as mean values with standard deviation (Table 1). We observed also that cerebral edema and 3–4 HE were more frequent in non-survivors compared to survivors respectively 45% vs 20%. Fibrinogen was significantly reduced in non-survivors compared to survivors, respectively 0.89 g/l±0.11 vs 2.1±0.59 *p* < 0.0001.

**Conclusions:** We observed that these parameters, particularly the combination of lactate [1], serum creatinine, ammonia is of great importance to identify early, patients who will not survive with medical therapy and so to select potential candidates for liver transplantation.

**Reference**

1. Figueira et al. BMC Gastroenterol 21:252, 2021

**Table 1 (abstract P114):** Variables related to outcome in patient survivors and nonsurvivors

	Survivors	Non-survivors	*p*
Lactates T0 mmol/l	3.17±0.96	6.20±1.99	< 0.0001
Lactates T 12h	3.42±0.7	7.34±2.21	< 0.0001
Ammonia µmol/l Day 2	104.9±20.8	162.55±35.3	< 0.0001
Sodium mmol/l	134.5±5.10	125.7±5.21	< 0.0001
SOFA score Day 3	9.66±1.66	13.95±2.99	< 0.0007

## P115

### Outcome of critically ill patients with liver cirrhosis and prolonged intensive care unit stay

#### K Roedl^1^, A Drolz^2^, T Horvatits^2^, J Kluwe^2^, S Kluge^1^, V Fuhrmann^1^

##### ^1^University Medical Centre Hamburg-Eppendorf, Department of Intensive Care Medicine, Hamburg, Germany, ^2^University Medical Centre Hamburg-Eppendorf, Department of Medicine I, Hamburg, Germany

*Critical Care* 2022, **26(Suppl 1):** P115

**Introduction:** Critically ill patients with liver cirrhosis and acute-on-chronic liver failure (ACLF) suffer from exceeding high mortality. Different studies proposed to discontinue therapy when > 3 organs fail or a CLIF-C ACLF score of > 64 points at day 3–7 after admission is present. However, the outcome of critically ill cirrhotic patients with prolonged ICU stay (≥ 7 days) remains largely unclear.

**Methods:** Retrospective analysis of prospectively collected data of all patients with liver cirrhosis admitted to the Department of Intensive Care Medicine of the University Medical Center Hamburg-Eppendorf (Hamburg, Germany) between 01/2009 and 01/2017. Demographics, clinical parameters, length of ICU stay and mortality as well as common ICU and liver specific scores were assessed.

**Results:** We could identify 1041 patients with liver cirrhosis, of those 32% (n = 335) had a prolonged ICU stay (≥ 7 days). In the multivariate regression analysis, a prolonged ICU stay was independently associated with SAPS II [OR 0.981, 95% CI (0.971 to 0.991); *p* < 0.001], vasopressor therapy [OR 4.355, 95% CI (2.715 to 6.521); *p* < 0.001], mechanical ventilation [OR 4.208, 95% CI (2.715 to 6.521); *p* < 0.001], RRT [OR 2.531, 95% CI (1.809 to 3.541); *p* < 0.001] and antibiotic therapy [OR 6.475, 95% CI (2.508 to 16.718); *p* < 0.001]. After 28-/90 days 48% vs. 35% and 62% vs. 42% patients with prolonged or non-prolonged ICU stay had died or received liver transplantation, respectively (both *p* < 0.001). Best predictors for 28-day mortality of patients with prolonged ICU stay were CLIF-C ACLF_Lactate_ (AUC 0.781) and CLIF-SOFA (AUC 0.780) score.

**Conclusions:** One third of critically ill patients with liver cirrhosis presents with a prolonged ICU stay. Although mortality was higher compared to patients with a short ICU stay outcomes were not futile.

## P116

### Cytokine adsorption in patients with acute-on-chronic liver failure (CYTOHEP): a clinical trial protocol of a single center, open-label, randomized, controlled intervention trial

#### A Sekandarzad^1^, D Bettinger^2^, E Weber^3^, E Graf^4^, EP Prager^5^, T Wengenmayer^1^, A Supady^1^

##### ^1^University of Freiburg, Department of Medicine III (Interdisciplinary Medical Intensive Care), Medical Center, Faculty of Medicine & Department of Cardiology and Angiology I, Heart Center, Freiburg, Germany, ^2^Medical Center University of Freiburg, Faculty of Medicine, University of Freiburg, Department of Medicine, Freiburg, Germany, ^3^Medical Center University of Freiburg, Faculty of Medicine, University of Freiburg, Institute of Medical Biometry and Statistics, Freiburg, Germany, ^4^University of Freiburg, Medical Center University of Freiburg, Faculty of Medicine, University of Freiburg, Institute of Medical Biometry and Statistics, Freiburg, Germany, Freiburg, Germany, ^5^Medical Center University of Freiburg, Faculty of Medicine, University of Freiburg, Department of Nephrology and Primary Care, Freiburg, Germany

*Critical Care* 2022, **26(Suppl 1):** P116

**Introduction:** During recent years the evolution of systemic inflammation in patients with progressing hepatic decompensation and acute-on-chronic liver failure (ACLF) has been recognized and identified as an important trigger of extrahepatic organ failures [1–3]. In theory one assumes that reduction of pro-inflammatory cytokines helps to gain control over the systemic inflammation process, hereby preventing further progress of ACLF and improve survival.

**Methods:** The CYTOHEP study is designed to assess the benefit of extracorporeal hemoadsorption using the CytoSorb (CytoSorbents Corporation, Monmouth Junction, NJ, USA) device in patients with ACLF. The CytoSorb device will be used in addition to continuous renal replacement therapy (CRRT) for 72 h and will be compared to a control group treated with CRRT alone. For safety assessment, a third group will be assessed without CRRT and extracorporeal hemoadsorption. All patients will be randomized in a 1:1:1 fashion in one of the study groups.

**Results:** Our primary endpoint of the study is that extracorporeal hemoadsorption using the CytoSorb adsorber in combination with CRRT in patients with ACLF is a safe and efficient method to reduce bilirubin. In order to gain more insight into the pathogenesis of ACLF and to better understand the mode of action of the CytoSorb device a broad array of inflammatory parameters are examined.

**Conclusions:** The extracorporeal hemoadsorption with CytoSorb is clinically widely applied but lacks reliable evidence. Its application in ACLF is not justifiable since randomized controlled studies are non-existent. The CYTOHEP study attempts to elucidate the role of extracorporeal hemoadsorption in the systemic inflammation process and progress of ACLF and represents an innovative and novel approach in the therapy of ACLF.

**References**

1. Arroyo V et al. J Hepatol 74:670–685, 2021.
2. Claria J et al. Hepatology 69:1686–1701, 2019.
3. Moreau R et al. J Hepatol 72:688–701, 2021.

## P117

### The effects of the first feeding time on the prognosis of the patients with trauma who were followed up in pediatric intensive care unit

#### D Yildizdas

##### Çukurova University, PICU, Adana, Turkey

*Critical Care* 2022, **26(Suppl 1):** P117

**Introduction:** Providing support for early enteral nutrition reduces the severity and complications of the disease, shortening the length of stay in the intensive care unit and affecting the patients positively. Although there is not enough evidence about when to start feeding in intensive care patients, it is recommended to start feeding within 24–48 h with hemodynamically stable condition. Conditions such as surgery, sepsis and trauma increase the energy needs of patients. The aim of this study was to determine the first enteral nutrition time of patients with trauma followed up in the pediatric intensive care unit.

**Methods:** The cases followed up between July 2018 and June 2019 in our pediatric intensive care unit due to trauma were examined. The demographic, clinic findings and first enteral nutrition hours of the patient were recorded retrospectively.

**Results:** A total of 49 patients with trauma were included in the study. The ages of the patients were minimum 6 months and maximum 17 years. Thirty eight (77.6%) of the patients were male. There were 31 patients (63.2%) fed within the first 24 h of admission to intensive care unit and 14 (28.6%) patients fed within 24–48 h, and the number of patients with an initial enteral nutrition time over 48 h was 4 (8.2%). The reason for not being fed within the first 48 h was the requirement of an abdominal surgery after trauma.

**Conclusions:** It is of importance to provide nutritional support in critically ill children with trauma in the intensive care unit, as in other patient groups. If there is no contraindication in terms of nutrition, patients should be fed as early as possible, the enteral route should be preferred, and the sufficiency of the calories given should be frequently evaluated.

**Reference**

1. Sachdev G et al. J Parenter Enteral Nutr 44:874–9, 2020.

## P118

### Effects of a carbohydrate-modified, diabetes-specific enteral tube feed high in monounsaturated fatty acids on glycemic variability in neurocritical care patients: a randomized, double-blind, multicenter study

#### R Beer^1^, M Kofler^2^, E Höfner^3^, J Weber^3^, F Gruber^4^, V Rass^2^, AJ Schiefecker^2^, B Pfausler^2^, R Helbok^2^

##### ^1^Medical University of Innsbruck, Department of Neurology—Neurointensive Care, Innsbruck, Austria, ^2^Medical University of Innsbruck, Innsbruck, Austria, ^3^Klinikum Klagenfurt am Wörthersee, Klagenfurt, Austria, ^4^Kepler Universitätsklinikum, Linz, Austria

*Critical Care* 2022, **26(Suppl 1):** P118

**Introduction:** High glycemic variability (GV) may exacerbate secondary injury in neurocritical care patients and is associated with adverse outcomes. We investigated whether a high-caloric (1.5 kcal/ml), diabetes-specific formula (DSF) improves blood glucose (BG) control in patients with poor-grade hemorrhagic stroke receiving intensive insulin therapy and enteral nutrition.

**Methods:** Mechanically ventilated patients with intracerebral (ICH) or subarachnoid hemorrhage (SAH) were randomized at day 3–4 after ICU admission to receive either DSF (Diben 1.5 kcal HP tube feed; n = 13) or an isocaloric, isonitrogenous standard formula (SF, Fresubin HP energy fibre tube feed; n = 15) for at least 5 days. Primary endpoint: GV (standard deviation of BG values) on intervention day (D) 3; other endpoints: insulin requirement, dysglycemic events, nutritional adequacy (ratio actual/planned calories), gastrointestinal (GI) tolerance.

**Results:** On D3, there was a trend towards lower GV with DSF vs. SF (within patient standard deviation [SD] 18.6 ± 8.7 vs. 21.2 ± 7.7 mg/dl, pooled SD 20.4 vs. 22.9 mg/dl; *p* = 0.349). The number of patients with hypoglycemic events (8/13 vs. 15/15) and events per patient (2.6 ± 4.5 vs. 5.3 ± 6.7) was significantly lower in the DSF than in the SF group (*p* = 0.013 and *p* = 0.026). More SAH patients receiving DSF required insulin over time. The insulin dose tended to be higher in the DSF than in the SF group. Nutritional adequacy was app. 90% overall and higher with DSF vs. SF from D2-D5 (*p* = 0.036 at D3). GI tolerance was comparable between groups. Fewer patients receiving DSF experienced GI adverse events potentially related to the feeding formula (6/13 vs. 9/15, *p* = 0.70).

**Conclusions:** In poor-grade hemorrhagic stroke patients, a DSF tempered GV and reduced the number of hypoglycemic events compared to a SF. The DSF was safe and well-tolerated, allowing an increased nutrition delivery while contributing to improved BG control. The DSF may be considered as a meaningful feeding formula in this vulnerable patient population.

## P119

### Alternative substrates in the critically ill subject (ASICS): a feasibility study

#### A McNelly^1^, A Langan^2^, D Bear^3^, T Martin^4^, K Rooney^5^, S Eaton^6^, S Heales^6^, J Prowle^2^, H Montgomery^7^, Z Puthucheary^1^

##### ^1^Queen Mary University of London, London, UK, ^2^Royal London Hospital, London, UK, ^3^St Thomas’s Hospital, London, UK, ^4^Royal London, London, UK, ^5^Bristol Royal Infirmary, London, UK, ^6^UCL Great Ormond Street Institute of Child Health, London, UK, ^7^UCL, London, UK

*Critical Care* 2022, **26(Suppl 1):** P119

**Introduction:** Ketogenic feeding (KF) might reduce muscle loss in critical illness (CI). We performed a randomised controlled feasibility study to see if intensive care unit (ICU) patients can be recruited into a study of enteral KF; if feed can be prepared, administered, tolerated, and raises plasma and urine ketone body levels.

**Methods:** Patients recruited ≤ 48 h after ICU admission in two UK ICUs; randomised to 10d KF or standard feed (SF) enterally. Inclusion: (i) > 18 yrs old (ii) prescribed enteral feed(iii)likely mechanical ventilation > 48 h, on ICU ≥ 5d survival ≥ 10d (iv) multi-organ failure (SOFA score > 2 in > 2 areas). ClinicalTrials.gov NCT04101071. Dietitian-prescribed modular KF: reconstituted on ICU [80% fat (40–80% medium chain triglycerides (Betaquik®, Vitaflo)); 20% protein (Renapro®, Stanningley Pharma); 5% carbohydrate (Maxijul®, Nutricia)]. Data were collected for feasibility of recruitment, retention (receipt of 10d randomised feed and those on ICU ≥ 5d if < 10d feed); Adverse events (AEs); blood glucose levels; urine/plasma ketone body levels.

**Results:** Of 286 patients screened, 29 recruited, 24 retained (12/14: SF; 12/15 KF). It was possible but laborious to calculate and prepare feed constituents to meet patients’ daily nutritional needs, and for bedside nurses to give it. No related or unexpected serious AEs were found. AE rates for episodes of daily vomiting and high gastric residual volume (GRV; > 350mls) were similar in SF and KF arms; episodes of diarrhoea (3d in a row) were more prevalent with KF than SF (76.92% vs. 52.33%, respectively). Daily blood glucose concentration remained ≥ 3.9 mmol/l in all KF patients but was ≥ 10 mmol/l in a greater proportion of SF than KF patients (57.48% vs. 26.85%, respectively). KF was associated with mild plasma ketosis (up to 2.7 mmol/l) and a greater urinary ketosis (up to 8 mmol/l).

**Conclusions:** Enteral KF in ICU patients is safe, tolerated and induces ketone body production. Ready-made feed would improve feasibility.

**Acknowledgement:** NIHR RfPB Grant PB-PG-0317–20,006.

## P120

### Medical nutrition therapy practices in cardiac surgical patients on ICU: current findings of the International Nutrition Survey

#### E Dresen^1^, C Stoppe^1^, X Jiang^2^, DK Heyland^3^

##### ^1^University Hospital Wuerzburg, Department of Anesthesiology and Intensive Care Medicine, Wuerzburg, Germany, ^2^Kingston General Hospital, Clinical Evaluation Research Unit, Kingston, Canada, ^3^Queen’s University, Department of Critical Care Medicine, Kingston, Canada

*Critical Care* 2022, **26(Suppl 1):** P120

**Introduction:** Among critically ill patients, cardiac surgery patients are at increased risk for inadequate medical nutrition therapy (MNT) leading to iatrogenic undernutrition during their stay in an intensive care unit (ICU). This study aimed to evaluate current MNT practices in critically ill patients after cardiac surgery.

**Methods:** An international prospective observational study was performed in 13 ICUs enrolling mechanically ventilated cardiac surgery patients with an ICU stay of at least 72 h. Nutrition data of routine clinical practice (e.g., estimated target energy and protein supply, initiation timepoint and type of nutrition used, actual amounts of energy and protein delivered) were collected daily from ICU admission to a maximum of 12 days. Data on MNT practices are shown as n (%) and mean (range), respectively.

**Results:** Across all participating sites, a total of 237 patients were enrolled. In the study population, enteral nutrition (EN) was initiated within 45 (0–227) hours after ICU admission (site average: 53 [10–79] hours). EN was predominantly used in 187 (79%) and supplemental parenteral nutrition (SPN) in 33 (14%) of patients. Overall, patients received 44.2% (0.0–117.2%) and 39.7% (0.0–122.8%) of prescribed energy and protein, respectively; the average adequacy per site was 47.5% (30.5–78.6%) for calories and 43.6% (21.7–76.6%) for protein, respectively, received from all nutritional sources.

**Conclusions:** In patients with prolonged ICU stay after cardiac surgery, initiation of EN was delayed and SPN was rarely used, both leading to inadequate delivery of energy and protein. The findings obtained in the present study emphasize that improvements in clinical practice are urgently needed to avoid iatrogenic undernutrition.

## P121

### Does the amino acid pattern in medical nutrition therapy affect muscle mass loss in adult ICU patients? A secondary analysis of a randomized controlled trial

#### E Dresen^1^, L Siepmann^2^, C Weißbrich^3^, L Weinhold^4^, C Putensen^3^, P Stehle^2^

##### ^1^University Hospital Wuerzburg, Department of Anesthesiology and Intensive Care Medicine, Wuerzburg, Germany, ^2^University of Bonn, Department of Nutrition and Food Sciences, Nutritional Physiology, Bonn, Germany, ^3^University Hospital of Bonn, Department of Anesthesiology and Intensive Care Medicine, Bonn, Germany, ^4^University Hospital of Bonn, Institute of Medical Biometry, Informatics and Epidemiology, Bonn, Germany

*Critical Care* 2022, **26(Suppl 1):** P121

**Introduction:** In long-term immobilized intensive care unit (ICU) patients, muscle mass loss is a crucial determinant of patient outcome. In this context, both the quantity and quality (amino acid pattern) of protein nutrition may play a key role in supporting muscle mass preservation. Our analysis aimed to evaluate potential associations of enteral/parenteral amino acid intake with muscle mass loss during the ICU stay.

**Methods:** Data were collected from a recent randomized controlled trial conducted in ICU patients investigating the effect of protein quantity (intervention [IG]: 1.8 g/kg body weight [BW]; standard [SG]: 1.2 g/kg BW/d) on the muscle mass loss over 28 days. Intake of individual amino acids (AA) and sum scores of indispensable, conditionally indispensable, and dispensable AA were calculated (group specific [n = 21 each] and in total [n = 42]). Inter-group differences were analyzed by t-tests; linear regression models tested the effects of individual AA and sum scores on the extent of muscle loss (significance level adjusted according to the Bonferroni procedure [α = 0.002]).

**Results:** Intake of indispensable AA covered actual recommendations (IG: 51.1 g/d, SG: 38.5 g/d; 41% of total AA supply). Conditionally indispensable AA (glutamine, tyrosine, cysteine, histidine, and arginine) accounted for 17% (IG: 21.4 g/d) and 18% (SG: 16.6 g/d) of total AA; glutamine supply (only given enterally) reached 0.06–0.07 g/kg BW/d. The intake of dispensable AA showed broad individual variations. During the study period, muscle mass decreased in both groups (IG: 30.4%; SG: 51.8%), but there was no statistically significant association observed with quantitative intake of single AA and sum scores (all *p* > 0.05).

**Conclusions:** Our working hypothesis could not be supported by the data presented. Possible explanations are the limited variations in AA intake due to the routine use of similarly composed nutrition products in the study center and the low number of patients included.

## P122

### Accuracy of ICU nurses versus physicians and impact of ICU experience on early prediction of mortality of critically ill patients: a further in-depth analysis of the PREMIUMS trial.

#### S Bogaert^1^, L Depauw^2^, T De Corte^2^, J Vermassen^2^, K Colpaert^2^, J Decruyenaere^2^

##### ^1^UZ Gent, Intensive Care, Ghent, Belgium, ^2^UZ Gent, Ghent, Belgium

*Critical Care* 2022, **26(Suppl 1):** P122

**Introduction:** We wanted to investigate if the effectiveness of predicting ICU mortality and in-hospital mortality (IHM) by caregivers was influenced by function and years of experience on ICU. This abstract is a deeper analysis of the PREMIUMS trial, the results of which are described in another abstract.

**Methods:** The study was performed at the 1056-bed Ghent University Hospital, and the questionnaire was made available electronically via the PDMS system. Included were all admitted surgical critically ill patients with an anticipated stay of more than 48 h. A total of 162 patients where at least one nurse and one physician were questioned about their level of experience and the patient’s prospect on probability of ICU and hospital survival were included in this analysis.

**Results:** We found an effective ICU mortality of 12.5% and effective IHM of 20.9%. Nurses vs. physicians showed no statistical difference in their ability of predicting mortality, here calculated by an AUC of 0.848 [0.729;0.967] vs. 0.899 [0.816;0.983] for ICU mortality and 0.834 [0.735;0.933] vs. 0.913 [0.858;0.968] for IHM. Both nurses and physicians overestimated mortality risk, with a mean predicted ICU mortality of 19.4% vs. 29.9%, and a predicted IHM of 26.8% vs. 29.1%. When we analysed if more experienced healthcare workers (> 20y) performed better than younger colleagues (< 5y experience) we did not find a significant difference. Their ability of predicting showed an AUC of 0.899 [0.817;0.890] vs. 0.914 [0.827;1.000] for ICU mortality and an AUC of 0.845 [0.748;0.942] vs. 0.894 [0.819;0.968] for IHM.

**Conclusions:** We found no difference between nurses and physicians working on ICU in their ability of predicting ICU mortality and in-hospital mortality. Very experienced ICU staff was not better in predicting mortality than colleagues with less experience.

## P123

### PREMIUMS: predicting mortality in ICU patients by healthcare workers, scoring systems and artificial intelligence.

#### L Depauw^1^, S Bogaert^2^, T De Corte^2^, J Vermassen^2^, K Colpaert^2^, J Decruyenaere^2^

##### ^1^UZ Gent, intensive Care, Ghent, Belgium, ^2^UZ Gent, Ghent, Belgium

*Critical Care* 2022, **26(Suppl 1):** P123

**Introduction:** Healthcare workers (HW) often have a gut feeling concerning the probability of a patient surviving an admission in the ICU. However, limited data is available on the accuracy of this estimation. Our goal was to compare mortality predictions in ICU patients made by healthcare workers, validated scoring systems and a machine learning model.

**Methods:** ICU staff (physicians and nurses) of a Belgian university hospital were asked to give a prediction of mortality (both ICU and in hospital (IHM)) for patients in their care within the first 24 h of admission. All adult patients were eligible for the study, elective surgery patients with anticipated length of stay less than 48 h were excluded. Comparison was made with validated scoring systems (APACHE II, SAPS II, SOFA) and a machine learning model (Random Forest (RF)) trained on a dataset of 15.000 patients from the same hospital.

**Results:** A total of 602 patients were admitted during this 12 week intervention. Mean ICU mortality was 8.5% (IHM 14.1%) which HW overestimated in most cases (ICU mortality 15.1% + / − 22.7 and IHM 19.9% + / − 25.1). Area under the curve (AUC) for prediction of ICU mortality was 0.922 (95% CI 0.877–0.968) for HW, compared to 0.739 (95% CI 0.663–0.815); 0.864 (95% CI 0.813–0.915); 0.84 (95% CI 0.785–0.896) and 0.812 (95% CI 0.74–0.885) for SOFA, APACHE II, SAPS II and RF respectively. For prediction of IHM, AUC was 0.871 (95% CI 0.828–0.915) for HW, compared to 0.633 (95% CI 0.56–0.705); 0.768 (95% CI 0.709–0.828); 0.75 (95% CI 0.69–0.811) and 0.768 (95% CI 0.728–0.843) for SOFA, APACHE II, SAPS II and RF respectively.

**Conclusions:** Healthcare workers are at least as accurate in predicting mortality as validated scoring systems and a trained Random Forest model.

## P124

### Post-intensive care COVID survivorship clinic: a single centre experience

#### M Gilmartin^1^, J Collins^1^, S Mason^1^, G McDermott^1^, M Baily-Scanlan^2^, D Hevey^3^, M Donnelly^1^, V O’Doherty^4^, YP Kelly^1^

##### ^1^Tallaght University Hospital, Intensive Care, Dublin 24, Ireland, ^2^Tallaght University Hospital, Physiotherapy, Dublin 24, Ireland, ^3^Trinity College Dublin, School of Psychology, Dublin 2, Ireland, ^4^Tallaght University Hospital, Psychology, Dublin 24, Ireland

*Critical Care* 2022, **26(Suppl 1):** P124

**Introduction:** Patients discharged from the intensive care unit (ICU) post coronavirus-19 (COVID-19) pneumonitis may experience long-term morbidity related to their critical illness, the treatment for this and the ICU environment.

**Methods:** We performed a prospective cohort study in a post-ICU (PICS) follow-up clinic at Tallaght University Hospital in October 2020 for patients who had been admitted to the ICU in our institution with COVID-19 pneumonitis six months earlier. Our clinic was staffed by critical care physicians, a psychologist, a physiotherapist and a research nurse. Our aim was to characterise the cognitive, psychological and physical consequences of COVID-19 in patients admitted to the ICU and discharged alive.

**Results:** A total of 22 patients attended the 6-month PICS follow-up clinic following admission to ICU with COVID-19 pneumonitis. The majority of these patients were male and obese. The most common comorbidities were hypertension, diabetes mellitus and ischaemic heart disease. The median ICU length of stay was 21 days (IQR 2–75 days) with a median hospital length of stay of 37 days (IQR 8–130 days). The mean ICU Mobility Scale (IMS) score at the PICS clinic was low at 9.8 (SD 0.4). Only 59% of patients were independent with regard to their activities of daily living (ADLs). 8/14 (57%) of patients had returned to work by 6 months post ICU discharge. Their mean Intensive Care Psychological Assessment Tool (IPAT) score was high at 6.7 (SD 4.6) with a high mean Post-Traumatic Stress Disorder (PTSD) score of 21.1 (SD 17.5).

**Conclusions:** In this single centre prospective cohort study, we found that patients have a high burden of physical and psychological impairment at 6 months following ICU discharge post COVID-19 pneumonitis; in many cases requiring specialist referrals for long-term input. We advocate for increased resources for this much needed follow-up multidisciplinary intervention for an ever-growing population of patients.

## P125

### Nutritional status of COVID-19 patients one year post-ICU stay

#### P Lakenman^1^, J Van Bommel^2^, J Olieman^3^, K Joosten^4^

##### ^1^Erasmus Medical Centre, Division of Dietetics, Department of Internal Medicine,, Rotterdam, Netherlands, ^2^Erasmus Medical Centre, Intensive Care Medicine, Rotterdam, Netherlands, ^3^Erasmus Medical Centre, Division of Dietetics, Department of Internal Medicine, Rotterdam, Netherlands, ^4^Erasmus Medical Centre—Sophia Children’s Hospital, Intensive Care Unit, Department of Paediatrics and Paediatric Surgery, Rotterdam, Netherlands

*Critical Care* 2022, **26(Suppl 1):** P125

**Introduction:** Our aim was to describe nutritional status (NS) of critically ill COVID-19 patients 1 year post-ICU stay. Malnutrition and weight loss were observed during hospitalization. Post-ICU discharge patients often continue to suffer from physical complaints and poor nutritional intake, which can negatively affect NS.

**Methods:** Observational study including adult COVID-19 patients 1 year post-ICU stay. NS assessment (nutrient balance, body composition and physical status) was performed. Nutritional intake (energy and protein), nutrition related complaints and losses were examined. Indirect calorimetry (Q-NRG +) was performed to determine nutritional requirements. Body composition (e.g. fat mass, fat free mass) was measured with bio-electrical impedance analysis (InbodyS10). Fat-free mass index (FFMI) and fat mass index (FMI) were calculated. Physical status was determined with handgrip strength (HGS). Overall values ≤ 10th percentile were considered too low and ≥ 90^th^ too high. Descriptive statistics were used for analysis.

**Results:** 48 patients were included (72% male; median age 60 years [IQR 52;65]). Median ICU stay was 19 days [IQR 10;30]. Median weight loss during ICU stay was 13% [IQR − 10; − 16] and 12% of this loss [IQR 7;16] was regained after 1 year. BMI was 26 kg/m^2^ [IQR 23;30] 1 year post-ICU, of which 24% was obese (BMI > 30 kg/m^2^). Normometabolism was predominantly observed (62%), followed by hypermetabolism (30%). Mean nutritional intake was 78% of calculated requirements. Nutrition related complaints occurred in < 5% of the patients. Most patients had a high FMI (55%) and minority had low FFMI (35%). Combination of a high FFMI and FMI was present in 17%. Three patients (6%) had low HGS.

**Conclusions:** Weight loss was almost fully regained 1 year post-ICU, often in combination with a high fat mass. A minority had low physical function. Whereas reported calculated requirements were not met, lifestyle coaching remains indicated to optimize NS.

## P126

### Neuromuscular electrical stimulation prevents the skeletal muscle weakness in patients with severe COVID-19 associated with sepsis and septic shock: a case series

#### WN Costa, ST Grams, LT Saraiva, IC Salles, RF Righetti, WP Yamaguti

##### Hospital Sírio-Libanês, Serviço de Reabilitação, São Paulo, Brazil

*Critical Care* 2022, **26(Suppl 1):** P126

**Introduction:** Neuromuscular electrical stimulation (NMES) results on muscle strength and functionality in patients with severe coronavirus disease 2019 (COVID-19) associated with sepsis and septic shock are unknown.

**Methods:** Patients with severe COVID-19 associated with sepsis or septic shock were selected. The NMES intervention was performed on 7 consecutive days in a daily session of 40 min (frequency of 100 Hz and a pulse of 350 μs). Electrodes were positioned in the vastus medialis and vastus lateralis muscles, and inguinal region. The outcome measures were the femoris cross-sectional area (RF-CSA), thickness of the anterior compartment of the quadriceps muscle, rectus femoris echogenicity, International Classification of Functioning, Disability, and Health (ICF)-muscle strength, Physical Function ICU Test-scored (PFIT-s), Morton Mobility Index (DEMMI), and the Surgical Intensive Care Unit Optimal Mobilization Score (SOMS). The patients were evaluated on days 1, 5, and 8.

**Results:** The RF-CSA area decreased significantly (− 16.9%; *p* < 0.05) from days 1 to 8, but showed maintenance of the thickness of the anterior compartment of the quadriceps muscle (− 3.20%; *p* = 0.3) from days 1 to 8. These patients showed a reduction of 2.1% per day in the rectus femoris cross-sectional area and 0.3% per day in the thickness of the anterior compartment of the quadriceps muscle during 8 days. Patients showed maintenance of the echogenicity (1.3%; *p* = 0.8) from days 1 to 8 with an increase of 0.16% per day. All patients showed an increase in the MRC score and reduction of the ICF-muscle strength, meaning improved muscle strength from days 1 to 8 (*p* < 0.05). The PFIT-s increased from days 1 to 5 and improved until day 8 compared to day 5 (*p* < 0.05). DEMMI and SOMS scores increased on day 8 compared to days 1 and 5 (*p* < 0.05).

**Conclusions:** NMES showed a protective effect on muscle strength and improve the functionality of patients with several COVID-19 associated with sepsis and septic shock.

## P127

### Long term follow-up of patients admitted to ICU for acute respiratory failure from SARS-CoV-2 infection

#### E Casarotta, E Damiani, G Mariotti, C D’Angelo, G Stanzione, D Di Falco, C Gorbi, F Picchio, C Sampaolo, A Donati

##### Università Politecnica delle Marche, Biomedical Sciences, Ancona, Italy

*Critical Care* 2022, **26(Suppl 1):** P127

**Introduction:** The clinical features and acute complications of patients with severe COVID-19 have long been described. However, little information is available about the quality of life and long-term persistent symptoms after discharge from ICU [1]. The purpose of this study is to describe the symptoms, the neurological, functional and psychological status 6 months after discharge from ICU.

**Methods:** We performed an observational prospective monocentric study. We considered patients admitted to ICU for acute respiratory failure from SARS-CoV-2 infection from March 2020 to March 2021 and discharged alive. Patients underwent a telephone interview 6 months after discharge. We asked for residual symptoms. Neurological, psychological and functional status was assessed using validated questionnaires.

**Results:** Of the 111 eligible patients, 6 (5.4%) died after discharge and 35 (31,5%) were lost to follow-up. Demographic characteristics of the population are presented in Table 1. At 6 months after discharge, 9 (12.8%) patients reported no symptoms. Dyspnoea was present in 45 (64.3%) patients, asthenia in 39 (55.7%). 32 patients (45.7%) reported memory deficit, 28 (40%) peripheral neuropathy, 17 (24.3%) artrhalgias and 6 (8.6%) dysphagia. Palpitations were present in 16 (22.8%) patients and 10 (14.3%) patients experienced insomnia or agitation. The PC-PTSD-5 was positive in 21 (30%) patients. Based on PCFS Scale*,* 13 (18.6%) patients reported no functional limitations, 14 (20%) negligible functional limitations, 24 (34.3%) slight functional limitations, 5 (7.1%) moderate functional limitations and 14 (20%) severe functional limitations. The GOS-E score was 6 [5–8] and it was inversely correlated with the number of hypoxia episodes (Spearman rho =  − 0.25, CI 95% − 0.47 to − 0.01, *p* = 0.03).

**Conclusions:** At 6 months after ICU discharge, only a few patients reported no symptoms or functional limitations. Dyspnoea, asthenia and neurological symptoms were the most frequently described.

**Reference**

1. Shoucri SM et al. BMJ Open 11:e049488, 2021

**Table 1 (abstract P127):** Demographic characteristics of the population

	Patients (n = 70)
Male gender, n (%)	56 (80)
Age, years	58.8 ± 10.9
Weight, kg	90 [80–110]
Height, cm	175.3 ± 9.5
BMI, kg/m^2^	29.4 [27.1–35.1]
Charlson Comorbidity Index	2.1 ± 1.5

## P128

### Evaluating post-intensive care syndrome in critically unwell COVID-19 patients

#### R Mani

##### St Georges Hospital, General Intensive Care, London, UK

*Critical Care* 2022, **26(Suppl 1):** P128

**Introduction:** The aim of this study is to investigate post-intensive care syndrome (PICS) in COVID-19 patients treated in intensive care, using a validated screening tool. The COVID-19 pandemic increased pressure on critical care, but also increased the number of patients vulnerable to long-term effects of a critical care admission. PICS is a deterioration of physical, cognitive or mental functioning as a result of a critical illness, after hospital discharge [1]. PICS-Q is a validated 18-point screening tool to identify PICS. Each question has a value from 0 (never) to 3 (always) [2].

**Methods:** Using PICS-Q, this was a prospective single-centre study of adult COVID-19 patients treated in level 2 or 3 care for greater than 4 days, and then suitable for follow-up at the same centre.

**Results:** From study period there were 32 eligible patients. Postal questionnaires were sent and replies were anonymised. There were 13 replies. Individual scores were combined to give a total score for each question, allowing analysis of which symptoms were most reported by patient group, seen below. Patients suffered from the physical symptoms., such as joint stiffness and fatigue and weakness the most (cumulative scores of 21, 16 and 15 from the 13 patients). Mental symptoms such as worry and ease to anger were also highly reported (16 and 13). Cognitive symptoms were reported the least apart from struggling to concentrate (Fig. 1).

**Conclusions:** These patients represent a snapshot of those affected by COVID-19 outbreak treated in critical care. As the number of patients treated in ICU goes up, the number of patients vulnerable to suffering from PICS will mirror that, representing a further challenge to stretched health services. Further work is required to identify and treat symptoms related to PICS.

**Acknowledgments:**

Thanks to Jeong Y, Kang J, for allowing use of PICS-Q here.

**References**

1. Needham DM et al. Crit Care Med 40:502–9, 2012
2. Jin Jeong Y et al. Intensive Crit Care Nurse 55:102,756, 2019

**Fig. 1 (abstract P128):**
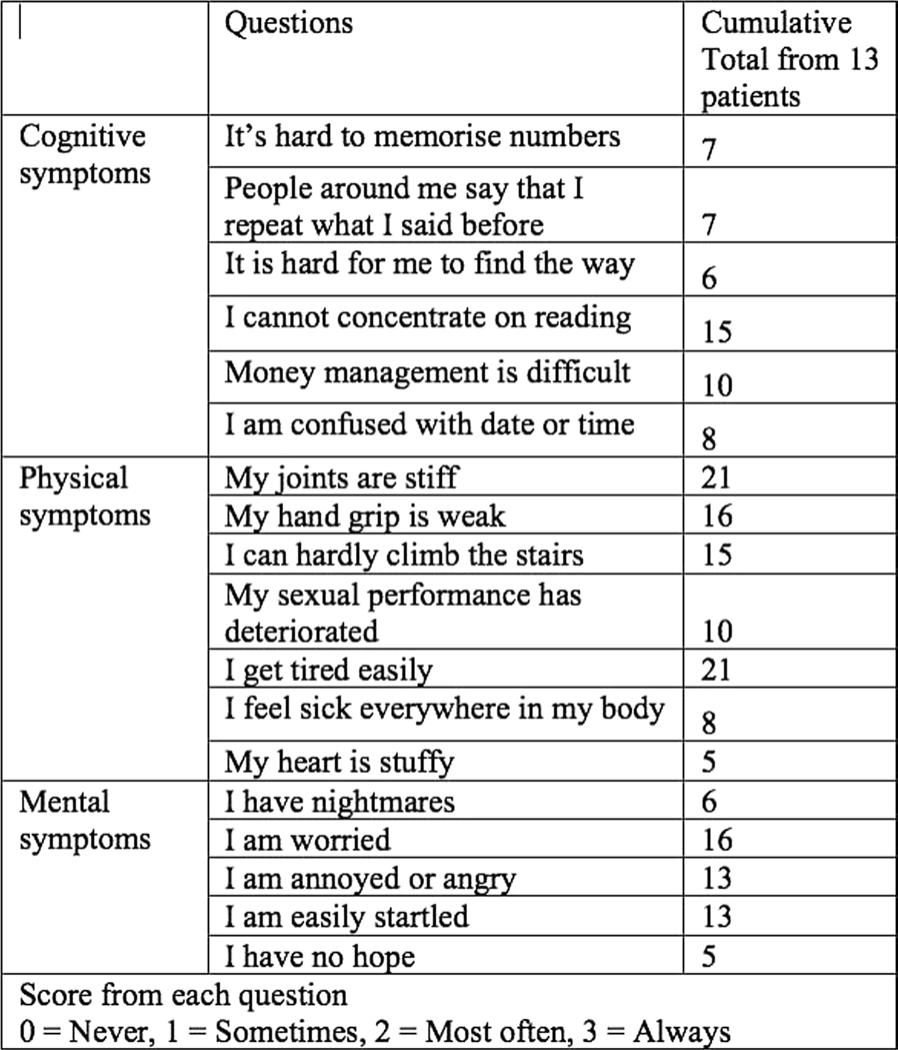
The validated 18-point PICS-Q questionnaire, with the cumulative scores from the patient group [2]. Participants can score each question from 0 to 3 depending on how frequently they report the symptom. This gives a total score of 39 from the patient group (as there were 13 patients).

## P129

### Novel sub-phenotypes highlight heterogeneity of characteristics and guide individualized treatment in chronic critical illness

#### PZ Liu^1^, SC Li^1^, T Zheng^2^, J Wu^3^, WB Gong^2^, ZB Zhang^4^, XW Wu^2^, Y Zhao^5^, JA Ren^1^

##### ^1^Jinling Hospital, Medical School of Nanjing University, Research Institute of General Surgery, Nanjing, China, ^2^Jinling Hospital, Research Institute of General Surgery, Nanjing, China, ^3^BenQ Medical Center, The Affiliated BenQ Hospital of Nanjing Medical University, Research Institute of General Surgery, Nanjing, China, ^4^Chinese PLA General Hospital, Beijing, China, ^5^BenQ Medical Center, The Affiliated BenQ Hospital of Nanjing Medical University, Nanjing, China

*Critical Care* 2022, **26(Suppl 1):** P129

**Introduction:** Patients who survived from the initial attack in ICU yet represent the long-term stay status with persistent organ dysfunction are recognized as chronic critical illness (CCI) and cause heavy burden on their family, healthcare system and the society. CCI patients showed great heterogeneity. Therefore, it is important to identify the subtypes in order to guide clinical practice and trials. In this study, we used multiple machine learning methods to identified CCI phenotypes and their characteristics in disease pattern and heterogenous treatment effect.

**Methods:** Data from three large critical care electronic healthcare record databases and one single-center tertiary referral hospital in China were extracted and formed four observational cohorts. Multiple unsupervised clustering algorithms were employed independently for phenotypes derivation and cross validation. A Bayesian-based framework was applied for treatment effect investigation.

**Results:** In total 6862 patients were enrolled in this study. Clustering process identified four CCI phenotypes as A (mild), B (short course), C (dehydrated and catabolic multiple organ dysfunction), and D (multiple organ failure). Each phenotype showed distinct clinical features and disease behaviors (Fig. 1). Survival analysis indicated that Type D has the worst prognosis (ICU mortality: 29.9%, *p* < 0.001) and Type A has the best prognosis (ICU mortality: 13.1%, *p* < 0.001). Bayesian causal inference analysis demonstrated that there were great differences in the efficacy of various therapies in different subtypes and we proposed an optimized treatment framework in order to individually improve critical care.

**Conclusions:** We identified four novel phenotypes that revealed the different patterns within CCI patients. Our findings could assist clinical practice and enlighten future researches.

**Fig. 1 (abstract P129):**
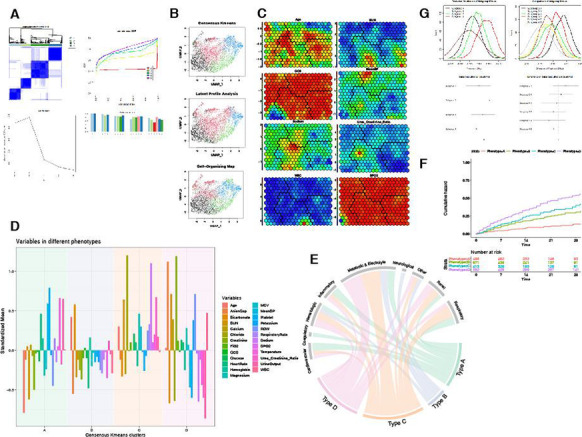
**A** Consensus K-means results showed best cluster number as 4; **B**) UMAP plots for comparison of three independent unsupervised clustering processes (Consensus K-means, Latent profile analysis, and Self-organizing map) showed similar phenotypes distribution; **C** Selected representative variable property maps of SOM showed heterogeneous assignment of different subgroups; **D** Standardized mean values of variables barplot in four CCI phenotypes; **E** Chord plot showed four phenotypes with different pattern features on organ system level; **F** Kaplan-Meier curve showed distinct cumulative hazard in subclasses; **G** Bayesian hierarchical modeling revealed heterogeneity of phenotypes responses to sedation drug dose rate.

## P130

### Development of an algorithm for predicting mortality in pre-hospital patients encountered by a physician-staffed helicopter emergency medical service system

#### E Reitala^1^, M Lääperi^2^, MB Skrifvars^1^, T Silfvast^3^, H Vihonen^4^, P Karhivuori^5^, M Tommila^6^, L Raatiniemi^7^, J Nurmi^1^

##### ^1^University of Helsinki and Helsinki University Hospital, Department of Emergency Care and Services, Helsinki, Finland, ^2^FinnHEMS Ltd, HEMS Operations, Vantaa, Finland, ^3^University of Helsinki and Helsinki University Hospital, Department of Anaesthesia, Intensive Care, and Pain Medicine, Helsinki, Finland, ^4^Tampere University Hospital, Emergency Medical Services, Centre for Prehospital Emergency Care, Department of Emergency, Anaesthesia and Pain Medicine,, Tampere, Finland, ^5^Kuopio University Hospital, Centre for Prehospital Emergency Care, Kuopio, Finland, ^6^Turku University Hospital and University of Turku, Department of Perioperative Services, Intensive Care Medicine and Pain Management, Turku, Finland, ^7^Oulu University Hospital, Medical Research Centre, University of Oulu, Research Group of Surgery, Anaesthesiology and Intensive Care, Division of Anaesthesiology, Oulu Centre for Prehospital Emergency Care, Oulu, Finland

*Critical Care* 2022, **26(Suppl 1):** P130

**Introduction:** Severity of illness scoring systems are in use in the intensive care unit setting and enable estimation for quality assurance purposes [1, 2], but similar tools are lacking in the prehospital emergency medicine setting. Using a national helicopter emergency medical services (HEMS) database, we developed an algorithm for predicting mortality in patients treated by physician-staffed HEMS units, suitable for real-life scenarios.

**Methods:** We performed a retrospective observational register-based cohort study of patients treated by all five Finnish HEMS units between 2012 and 2019. We analyzed the relationship between 30-day mortality and physiological, patient-related, and circumstantial variables and performed derivation of the multivariable models for each combination of missing variables. Data were imputed using multiple imputation by chained equations. We used Least Absolute Shrinkage and Selection Operator to select the variables for each model and pooled the results using Rubin’s rule. The models were combined into an algorithm to allow risk estimation tool that accounts for missing variables.

**Results:** 28,373 of the 36,633 patients encountered were included of whom 8,611 died before 30 days. 11 variables (systolic blood pressure, heart rate, oxygen saturation, GCS, sex, age, vehicle type, mission located in medical facility, cardiac rhythm, delay from emergency call to HEMS arrival, and dispatch code) were included. The algorithm had an area under the receiver operating characteristic (AUROC) curve of 0.932 (95% CI, 0.929 to 0.935), Brier score of 0.091, calibration intercept of − 0.002 (95% CI, − 0.041 to 0.038), and slope of 0.996 (95% CI, 0.974 to 1.018).

**Conclusions:** We present a novel severity of illness algorithm to be used in patients encountered by the HEMS that may aid in future quality improvement of HEMS and facilitate comparison between units.

**References**

1. Vincent JL et al. Crit Care 14:207, 2010.
2. Bouch DC et al. Continuing Education in Anaesthesia Critical Care & Pain 8:181–185, 2018.

## P131

### Incidence and leading causes of preventable deaths in a French regional trauma system

#### M Geslain, S Cozien, X Chapalain, N Chahir, J Thereaux, M Nonent, R Seizeur, O Huet

##### CHRU Brest, Brest, France

*Critical Care* 2022, **26(Suppl 1):** P131

**Introduction:** Trauma remains a leading cause of death world-wide, especially among young people. Optimizing care in trauma system remains a challenge. Trauma-related preventable death (TRPD) analysis is one of the means to improve the quality of care. The aim of this study was to assess the TRPD rate in our regional trauma system.

**Methods:** Retrospective cohort study. Trauma patients with fatal outcome hospitalized in Brest university-affiliated hospital in France between 2016 et 2020 were enrolled. Preventable death was defined using the WHO definition. All cases were reviewed by a multidisciplinary panel composed by anesthesiologist, intensivist, emergency doctor, surgeons and radiologists. Primary outcome was the TRPD incidence. Secondary outcomes were the type of errors leading to death according to the Joint Commission on the Accreditation of Healthcare Organizations (JCAHO) taxonomy.

**Results:** 104 deaths due to trauma were reviewed. Death causes were mainly due to traumatic brain injury (70%), and hemorrhagic shock (20%). The TRPD incidence was 15.4% (7 preventable deaths, 9 potentially preventable deaths and 88 non-preventable deaths). Mean ISS in preventable deaths was 28, and 36 in non-preventable deaths (*p* = 0.062). 244 errors were identified, and 82 in preventable deaths (34%). Most errors (53%) were due to excessive delay (diagnostic or treatment) (Table 1).

**Conclusions:** Trauma-related preventable deaths occurred in 15.4% of our patients. Triage error, delay and non-trauma-team initial resuscitation were the leading causes of errors in the studied population.

**Table 1 (abstract P131):** Main factors significantly associated with preventable deaths

	Preventable deaths (n = 16)	Non-preventable deaths (n = 88)	*p*
Triage error	7	4	< 0.001
Secondary transfer to level I trauma-center	7	6	0.001
Non-trauma-team initial resuscitation	14	38	0.003

## P132

### Risk factors associated with augmented renal clearance in a mixed ICU population: a retrospective study

#### M Perreault^1^, K Archambault^2^, E Bing^2^, YT Fang^2^, C Gras^3^, C Jabamikos^2^, KD Nguyen^2^, A Sananikone^2^, MA Duceppe^2^, A Marsot^4^

##### ^1^The Montreal General Hospital, Pharmacy Suite C1-200, Montreal, Canada, ^2^The McGill University Health Center, Dept of Pharmacy, Montreal, Canada, ^3^Université de Montpellier, Dept of Pharmacy, Montpellier, France, ^4^Université de Montreal, Faculté de Pharmacie, Montreal, Canada

*Critical Care* 2022, **26(Suppl 1):** P132

**Introduction:** Augmented renal clearance (ARC) is increasingly recognized in critically ill patients. This condition may lead to underdosing of renally excreted medications with negative clinical outcomes. The goal of this study was to identify demographic and clinical factors associated with ARC in a mixed ICU population.

**Methods:** This retrospective single center observational cohort study collected data of patients admitted in a mixed (medical, surgical and trauma) adult ICU. ARC, defined as a creatinine clearance of ≥ 130 ml/min/1.73 m^2^, was assessed through weekly 24-h urine collection. Univariate analysis was used to identify variables associated with ARC which were then entered as covariates in a logistic regression using a backward stepwise selection. Goodness-of-fit of the model was assessed and a receiver operator characteristic curve was generated.

**Results:** ARC was observed in 25.3% (n = 82) of the study cohort (n = 324). Age < 50 years old (AOR 7.32; 95% CI 4.03–13.29, *p* < 0.001), lower serum creatinine at ICU admission (AOR 0.97; 95% CI 0.96–0.99, *p* < 0.001) and admission for trauma (AOR 2.26; 95% CI 1.12–4.54, *p* = 0.022) were identified as independent risk factors. Our model showed acceptable discrimination in predicting ARC (area under the receiver operator curve 0.810; 95% CI 0.756–0.864, *p* < 0.001).

**Conclusions:** Age < 50 years old, lower serum creatinine upon ICU admission and trauma were identified as independent risk factors for ARC. Our findings are consistent with the literature and suggest that patients with a low serum creatinine upon ICU admission could have a higher risk of developing ARC.

## P133

### Utility of 8 h urine creatinine clearance to guide dosing in critically ill patients: a single centre retrospective analysis

#### P Rajeevkumar^1^, M Ostermann^2^, F Hanks^3^

##### ^1^Guys’ and St Thomas’ NHS Foundation Trust, Pharmacy, London, UK, ^2^Guys’ and St Thomas’ NHS Foundation Trust, Department of Critical Care, London, UK, ^3^Guys’ and St Thomas’ NHS Foundation Trust, Critical Care Pharmacy, London, UK

*Critical Care* 2022, **26(Suppl 1):** P133

**Introduction:** Serum creatinine (SCr) is used to calculate Cockcroft-Gault creatinine clearance (C&G CrCl) to estimate glomerular filtration rate (GFR) and guide drug dosing [1]. However, SCr is reported to have low sensitivity for detecting renal dysfunction in critical illness. Population based equations such as C&G CrCl have not been validated in acute kidney injury (AKI), secondary to induced pathophysiological changes. Direct measurement of CrCl is advocated such as 4–8 h urine creatinine clearance (UrCrCl) [1]. In this retrospective analysis we describe the variation in estimated GFR (eGFR) from results of C&G CrCl, CKD-EPI eGFR and measured UrCrCl.

**Methods:** Retrospective data collection and case note review from electronic health records (Phillips ICCA® and iSOFT Clinical Manager®) was completed from Dec 2018 to Nov 2021, for adult critical care patients admitted to Guy’s and St Thomas’ Foundation Trust who had a measured UrCrCl. The aims were to compare results and to assess the impact on drug dosing.

**Results:** In 28/31 (90.3%) patients, C&G CrCl was an over-estimation of renal function, with a median difference between C&G CrCl and measured UrCrCl of 239%. UrCrCl results triggered a change in drug dosing in 16/31 (51.6%) of patients. Drug dosing could have been optimised in a further 7/31 (22.6%) patients if the UrCrCl results had been noticed and acted upon within 24 h (Table 1).

**Conclusions:** UrCrCl provides a more accurate measure of GFR compared to C&G CrCl or eGFR and triggered a change in drug therapy in 52% of patients. UrCrCl is useful in patients in whom serum creatinine results may be confounded by extremes of age, frailty, low muscle mass and fluctuations in fluid balance. Thus, UrCrCl results serve to optimise drug dosing and limit risks of harm from both, toxicity and underdosing.

**Reference**

1. Hoste EAJ et al. Nephrol Dial Transplant 20:747–753, 2005

**Table 1 (abstract P133):** Results

Patient characteristics (n = 31)	Results
Median age	48 yrs
Median SCr [micromole/l]	56 (IQR 27–104.5)
Median C&G CrCl [ml/min]	109 (IQR 41–244)
Median eGFR [ml/min]	104 (IQR 52–236)
Median UrCrCl [ml/min]	39 (IQR 15–75)
Median % difference between C&G CrCl and UrCrCl	239% (IQR 134–415%)
UrCrCl result prompted change of drug therapy	51.6% (16/31)

IQR, interquartile range

## P134

### Validation of the augmented renal clearance in trauma intensive care (ARCTIC) scoring system for augmented renal clearance prediction in a trauma subgroup of a mixed ICU population

#### M Perreault^1^, K Archambault^2^, E Bing^2^, YT Fang^2^, C Jabamikos^2^, KD Nguyen^2^, A Sananikone^2^, MA Duceppe^2^, A Marsot^3^, M Chagnon^4^

##### ^1^The Montreal General Hospital, Pharmacy Suite C1-200, Montreal, Canada, ^2^The McGill University Health Center, Dept of Pharmacy, Montreal, Canada, ^3^Université de Montreal, Faculté de Pharmacie, Montreal, Canada, ^4^Université de Montreal, Service de Consultation Statistique, Montreal, Canada

*Critical Care* 2022, **26(Suppl 1):** P134

**Introduction:** Augmented renal clearance (ARC) is prevalent in trauma patients and leads to subtherapeutic levels of renally eliminated medications with potentially unfavorable clinical outcomes. The Augmented Renal Clearance of Trauma in Intensive Care (ARCTIC) score has been developed to predict ARC in critically ill trauma patients. Our primary objective was to validate this score among the trauma subgroup of a mixed intensive care patient cohort.

**Methods:** This single-center, retrospective, observational cohort study assessed ARC using a timed 24-h urine collection performed weekly. ARC was defined as a measured creatinine clearance of ≥ 130 ml/min/1.73m^2^. ARCTIC score performance was evaluated through an analysis of sensitivities and specificities and examination of receiver operator characteristic curves for the trauma subgroup, the medical/surgical subgroup and the pooled cohort.

**Results:** ARC was observed in 33.9% (n = 58) of trauma patients (n = 171) and 15.7% (n = 24) of medical/surgical patients (n = 153). Examination of different cutoffs for the ARCTIC score in our trauma population confirmed that the optimal cutoff score was ≥ 6. Comparison between ROC curves for ARCTIC score and for regression model based upon our data in trauma patients indicated validation of the score in this subgroup. Comparison of sensitivities and specificities for ARCTIC score between trauma (93.1% and 41.6%, respectively) and medical/surgical subjects (87.5% and 49.6%, respectively) showed no clinical nor statistical difference, suggesting validation for the medical/surgical subgroup as well.

**Conclusions:** In our mixed ICU population, the ARCTIC score was validated in the trauma subgroup. In addition, the ARCTIC score performed well in the surgical/medical population. Future studies should assess the performance of the ARCTIC score prospectively.

## P135

### Biomarkers of kidney stress during early critical illness identify patients with impaired kidney function at ICU discharge when assessed using cystatin C but not creatinine

#### AG Gillespie, A Bhardwaj Shah, JP Prowle, RH Haines

##### Critical Care and Perioperative Medicine Research Group, William Harvey Research Group, Queen Mary University of London, London, UK

*Critical Care* 2022, **26(Suppl 1):** P135

**Introduction:** The Nephrocheck™ assay is a combination two urinary biomarkers (TIMP-2∙IGFBP7) which predicts AKI risk. We investigated the association between kidney stress during early critical illness and kidney function at ICU discharge.

**Methods:** Participants were all inpatients at a critical care unit, UK. Those who died in ICU were excluded. TIMP-2∙IGFBP7 was measured on day 1, 3, 5, and 7, peak measurement in first 7 days was categorised as: Low risk (< 0.3), Low-Medium Risk (0.3–0.99), High-Medium Risk (1–2) or High Risk (2 +). eGFR at ICU discharge was assessed using creatinine and cystatin C. Differences in eGFR between groups were assessed using the Jonckheere-Terpstra test.

**Results:** We included 35 patients, median age 54 yrs (range 21–76). Median ICU stay was 16 days (range 5–54). Median baseline eGFR-Cr was 96 ml/min/1.73 m^2^ (range 16–121). 13 patients developed creatinine defined AKI in the first 10 days with 7 receiving kidney replacement therapy (KRT). Distribution of peak TIMP-2∙IGFBP7 within the first 7 days was: Low risk (N 3), Low-Medium Risk (N 11), High-Medium Risk (N 11), High Risk (N 10). Cystatin C eGFR at ICU discharge was lower than creatinine eGFR (70 vs 108 ml/min/1.73m^2^, *p* < 0.001). No patient remained on KRT at ICU discharge. Peak TIMP-2∙IGFBP7 category correlated with ICU discharge cystatin C eGFR (*p* = 0.0128) but not creatinine eGFR (*p* = 0.166) (Fig. 1).

**Conclusions:** In critical illness assessment of kidney function is impeded by falls in creatinine generation. Here kidney stress was detected in 86% of cases whereas creatinine defined AKI was observed in 40%. When kidney function was assessed using cystatin C, severity of early kidney stress identified worse kidney function at ICU discharge. TIMP-2∙IGFBP7 may help risk-stratify long term renal function following critical illness. Evaluation of kidney biomarker tests should consider using cystatin C rather than creatinine to assess kidney outcomes.

**Fig. 1 (abstract P135):**
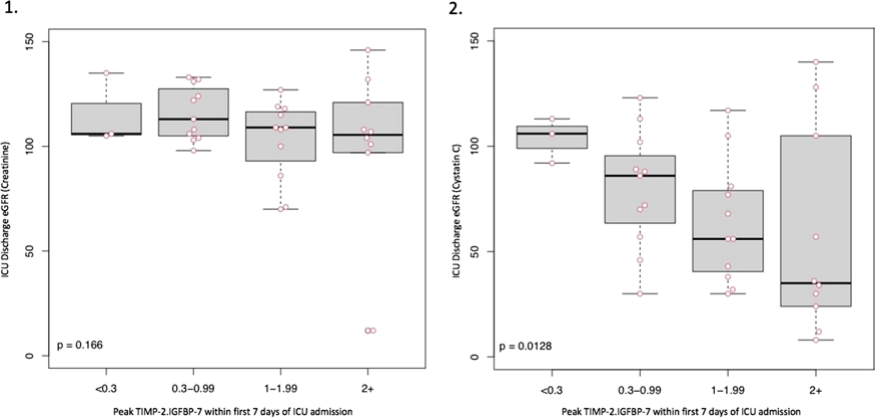
Whisker and Boxplot charts of findings. Charts show peak TIMP-2∙IGFBP7 (Nephrocheck) within first 7 days against ICU discharge eGFR (creatinine—Chart 1) (cystatin C—Chart 2). P values shown are based on Jonckheere-Terpstra test for trend.

## P136

### Epidemiology of acute kidney injury (AKI) within patients hospitalized in the emergency department of Mahdia

#### E Sghaier, N Jerbi, S Kebaier, N Chebbi, F Ben Salem, K Mefteh, S Bettout, I Dlala, S Marghli

##### Taher Sfar Hospital, Emergency Department, Mahida, Tunisia

*Critical Care* 2022, **26(Suppl 1):** P136

**Introduction:** Acute renal failure (AKI) is a real global public health which is a diagnostic and therapeutic issue in emergency departments. Better knowledge of the epidemiological characteristics will help improve the prevention and management of this disease.

**Methods:** Prospective descriptive study carried out in the emergency department of the Taher Sfar CHU in Mahdia between March 1 and August 31, 2021. Patients older than 18 years with acute renal impairment were included. Chronic renal insufficiency with hemodialysis stage was excluded as well as renal insufficiency having kept stable creatinine figures. The main objective of this work was to study the epidemiological profile of AKI within patients hospitalized in the emergency department.

**Results:** Our study englobed 103 patients: 53 men and 50 women, with a sex ratio of 1.06. The average age was 66 years. 94% of the patients included had a medical history of: diabetes 58.7% and HTA 54.4%, chronic renal failure was reported in 33%. Thus, most of the cases were under disease-modifying treatment: 64% under diuretics. The COVID attack affected 42% of hospitalized patients. Sepsis was the most common cause of AKI in the emergency room 38.8% of patients, followed by functional AKI(33,9%), 4 patients presented with obstructive AKI requiring placement of a JJ catheter. Patients were classified according to the KDIGO classification: stage I (n = 58) stage II (n = 8) stage (n = 37). Urgent hemodialysis was indicated in the presence of uremic syndrome (14.6%) in 23% of cases, otherwise vascular filling was indicated in 60 patients (58.3%). 64 patients were hospitalized in our emergency room (65%), 15 patients died (14.6%). Full recovery was observed in 18.4% of patients, 29.1% of patients partially recovered while 13.6% were dependent on long-term dialysis.

**Conclusions:** Acute kidney injury is one of the most common serious complications for all hospital admissions. The early diagnosis as well as the management of AKI creates a challenge for the emergency physician.

## P137

### Serial measurement of urinary C–C-motif chemokine ligand 14 (CCL14) and the persistence of severe acute kidney injury during critical illness

#### J Prowle^1^, LS Chawla^2^, P Kampf^3^, T Kwan^3^, P McPherson^3^, JA Kellum^4^

##### ^1^Queen Mary University of London, William Harvey Research Institute, London, UK, ^2^Veterans Affairs Medical Center, Veterans Affairs Medical Center, San Diego, CA, USA, ^3^Astute Medical, Inc., Astute Medical, Inc. (a bioMérieux company), San Diego, CA, USA, ^4^Department of Critical Care Medicine, University of Pittsburgh, Center for Critical Care Nephrology, Pittsburgh, PA, USA

*Critical Care* 2022, **26(Suppl 1):** P137

**Introduction:** In critically ill patients with stage 2–3 acute kidney injury (AKI) elevated urinary C–C-motif chemokine ligand 14 (CCL14) predicts persistence of severe AKI however the relationship of its trajectory to kidney outcomes has not been reported.

**Methods:** Using existing data from two multicenter studies (Ruby and Sapphire), we analyzed urinary CCL14 at 12 h intervals after onset of moderate to severe AKI. CCL14 was measured with the NEPHROCLEAR™ CCL14 Test (Astute Medical). Primary endpoint was persistent severe AKI (PS-AKI), defined as 72 h of stage 3 AKI, or death or receipt of dialysis prior to 72 h. We stratified the CCL14 concentrations into three levels: Low (≤ 1.3 ng/ml), Medium (> 1.3 to ≤ 13 ng/ml), and High (> 13 ng/ml) based on previously determined clinical risk cut-offs and grouped patients by CCL14 levels across 3 samples.

**Results:** We included 417 patients (median 65 yr) with 3 consecutive CCL14 measurements, 75 developed PS-AKI. Initial CCL4 levels were low in 196 (47%), medium in 180 (43%) and high in 41 (9.8%). In 66% of cases CCL14 category was unchanged from first to last timepoint. 158/196 patients with initially low levels of CCL14 remained in this category and had the lowest risk of developing PS-AKI, while patients with High CCL14 at any time had the highest risk. Patients with CCL14 levels in the mid-range (> 1.3 to ≤ 13 ng/ml) had intermediate risk and the risk increased with number of medium CCL14 results (Fig. 1).

**Conclusions:** In two-thirds of patients CCL14 levels were stable over 24 h. High CCL14 levels at any time conferred highest risk of PS-AKI while those with persistently low levels had lowest risk. Clinicians can have confidence in the prognostic interpretation of a single CCL14 result, but serial measurement may help refine prognosis over time.

**Fig. 1 (abstract P137):**
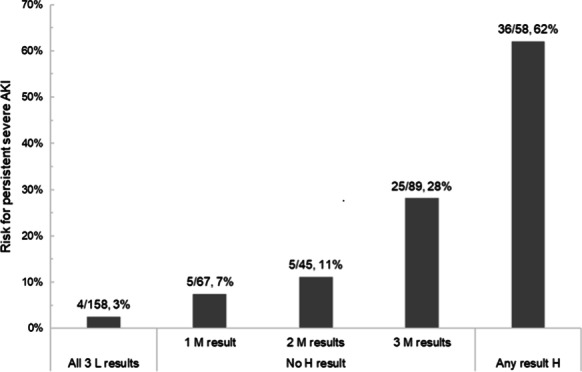
CCL14 trajectory over 24 h and kidney outcomes in patients with moderate to severe AKI. Urinary CCL14 levels were classified as low (L), medium (M) or high (H)

## P138

### Individual contributions of the KDIGO AKI care bundle’s components for the prevention of postoperative acute kidney injury (AKI)

#### TC Von Groote^1^, M Ostermann^2^, LG Forni^3^, M Meersch^1^, A Zarbock^1^, R Weiss^1^

##### ^1^University Hospital Münster, Department of Anaesthesiology, Intensive Care and Pain Medicine, Münster, Germany, ^2^King’s College London, Guy’s & St Thomas’ NHS Foundation Hospital, Department of Intensive Care, London, UK, ^3^University of Surrey, Department of Clinical and Experimental Medicine, Guildford, UK

*Critical Care* 2022, **26(Suppl 1):** P138

**Introduction:** The incidence and severity of postoperative acute kidney injury (AKI) can be reduced by implementing an AKI care bundle as developed by the Kidney Disease Improving Global Outcomes (KDIGO) guidelines. However, the impact of each individual component of this bundle was unclear and was therefore investigated in this analysis.

**Methods:** Data of the two PrevAKI-trials were combined, together including 554 cardiac surgery patients at high risk for AKI, as identified by elevated urinary biomarkers TIMP-2∙IGFBP7 [1, 2]. Patients were randomized to standard of care versus implementation of the care bundle. Univariate logistic regression of the bundle’s components was performed as a risk factor analysis of the whole cohort. Following this, individual treatment effects were analyzed, using the same method for the intervention group only.

**Results:** Hypotension, low cardiac index, and use of radiocontrast agents significantly increased the risk for AKI. Similarly, optimizing the hemodynamic situation (i.e. avoiding hypotension or a low cardiac output state) was the most important individual component for the prevention of AKI. However, this was not the case for avoidance of radiocontrast. Avoidance of nephrotoxic drugs was identified as an important component in the individual treatment effect analysis, however this finding showed wide confidence intervals (Table 1).

**Conclusions:** Our findings demonstrate the importance of maintaining adequate systemic blood pressure and cardiac output. If hypotension or low cardiac index occurs, timely hemodynamic optimization should be performed to prevent AKI. Whilst our analyses suggested a possible role for radiocontrast agents and nephrotoxic drugs, these factors had wide confidence intervals, indicating low certainty of these findings. Besides hemodynamic optimization, other bundle components had little or no impact on the bundle’s overall effectiveness.

**References**

1. Meersch M et al. Intensive Care Med 43:1551–1561, 2017
2. Zarbock A et al. Anesth Analg 133:292–302, 2021

**Table 1 (abstract P138):** Univariate logistic regression analysis of the KDIGO AKI care bundle’s individual components

Analysis	Risk factor (intervention + control; n = 554)	Individual treatment effect (intervention; n = 274)
	Odd’s ratio (95%CI), *p* value	Odd’s ratio (95%CI), *p* value
Hypotension MAP < 65 mmHg twice or < 60 mmHg once	2.30 (1.61–3.27), *p* < 0.05	2.37 (1.41–3.98), *p* < 0.05
Cardiac index < 3.0 l/min/m^2^	1.93 (1.10–3.38), *p* < 0.05	1.97 (1.11–3.52), *p* < 0.05
Hyperglycemia	1.44 (0.99–2.10), *p* = 0.056	1.07 (0.64–1.77), *p* = 0.80
ACEi/ARBs	1.19 (0.75–1.90), *p* = 0.46	0.85 (0.41–1.76), *p* = 0.86
Radiocontrast agents	3.57 (1.55–8.24), *p* < 0.05	2.57 (0.81–8.18), *p* = 0.11
Nephrotoxic drugs	1.58 (0.91–2.73), *p* = 0.11	8.19 (1.86–36.02), *p* < 0.05

## P139

### Optimizing postoperative hemodynamics in patients at high risk for acute kidney injury (AKI)—the higher, the better?

#### TC von Groote^1^, M Ostermann^2^, LG Forni^3^, M Meersch^1^, A Zarbock^1^, R Weiss^1^

##### ^1^University Hospital Münster, Department of Anaesthesiology, Intensive Care and Pain Medicine, Münster, Germany, ^2^King’s College London, Guy’s & St Thomas’ NHS Foundation Hospital, Department of Intensive Care, London, UK, ^3^University of Surrey, Department of Clinical and Experimental Medicine, Guildford, UK

*Critical Care* 2022, **26(Suppl 1):** P139

**Introduction:** We hypothesized that postoperative hemodynamics are strongly associated with acute kidney injury (AKI) and investigated optimal target thresholds of systemic blood pressure and cardiac output in the cardiothoracic ICU.

**Methods:** Data of the two PrevAKI-trials were combined, together including 554 patients after cardiac surgery [1, 2]. Four hours after surgery, patients at high risk for AKI were identified by the urinary biomarkers TIMP-2∙IGFBP7. Hemodynamics were then analyzed for the consecutive 12 h. In the combined dataset, the incidence and severity of AKI were compared between patients with postoperative hypotension and/or low cardiac index versus patients with hemodynamic stability. Thereafter, the incidence of AKI was evaluated for different hemodynamic thresholds of mean arterial pressure (MAP) and cardiac index (CI).

**Results:** AKI occurred significantly more frequently in patients with postoperative hypotension and/or low cardiac index compared to patients with hemodynamic stability (57.1% vs. 37,2%, *p* < 0.05). This effect was especially marked for more severe stages of AKI (29.8% vs. 9,3%, *p* < 0.01). Our analyses demonstrated an almost constant increase in the rate of postoperative AKI with lower MAP and CI thresholds. A prominent threshold for increase in AKI rates was found at a mean MAP of between 70 mmHg or 75 mmHg and a cardiac index lower than 3.0 l/min/m^2^ (Table 1).

**Conclusions:** Postoperative hypotension and low cardiac output are key risk factors for AKI in the ICU. For cardiac surgery patients at high risk for AKI, a higher target mean arterial pressure than the generally recommended 65 mmHg may be beneficial to prevent AKI, although this needs to be balanced against the potential side effects of required treatment intensity. Finally, our analyses strongly underline the importance of low cardiac output in the immediate postoperative period as a risk factor for kidney injury.

**References**

1. Meersch M et al. Intensive Care Med 43:1551–1561, 2017
2. Zarbock A et al. Anesth Analg 133:292–302, 2021

**Table 1 (abstract P139):** Hemodynamic thresholds of arterial blood pressure and cardiac output, over a 12-hour post-operative period

Mean MAP (mmHg)	Incidence of any AKI (KDIGO criteria)	Mean cardiac index (l/min/m^2^)	Incidence of any AKI (KDIGO criteria)
55–60	83.3% (5/6)	1.5–2.0	61.9% (13/21)
60–65	75.6% (34/45)	2.0–2.5	64.0% (48/75)
65–70	65.6% (82/125)	2.5–3.0	56.4% (62/110)
70–75	56.8% (92/162)	3.0–3.5	42.1% (24/57)
75–80	51.4% (56/109)	3.5–4.0	42.3% (11/26)
80–85	52.2% (35/67)	4.0–4.5	n/a
85–90	35.7% (10/28)	4.5–5.0	n/a

## P140

### Renin kinetics in cardiac surgery patients with postoperative administration of angiotensin-II

#### R Weiss^1^, M Meersch^1^, C Massoth^1^, M Küllmar^1^, L Chawla^2^, G Landoni^3^, R Bellomo^4^, J Gerss^5^, A Zarbock^1^, TC von Groote^1^

##### ^1^University Hospital Münster, Department for Anesthesiology, Intensive Care and Pain Medicine, Münster, Germany, ^2^Veterans Affairs Medical Center, San Diego, USA, ^3^IRCCS San Raffaele Scientific Institute, Department of Intensive Care and Anesthesia, Milan, Italy, ^4^Royal Melbourne Hospital, Department of Intensive Care, Melbourne, Australia, ^5^University of Münster, Institute of Biostatistics and Clinical Research, Münster, Germany

*Critical Care* 2022, **26(Suppl 1):** P140

**Introduction:** Hyperreninemia after cardiac surgery may contribute to the development of acute kidney injury (AKI) [1]. Through biofeedback, angiotensin-II (AT-II) may potentially attenuate hyperreninemia, whilst maintaining target mean arterial blood pressure (MAP > 65 mmHg). This trial assesses the association between administration of AT-II and plasma renin levels in cardiac surgery patients with postoperative vasoplegia and hyperreninemia.

**Methods:** A cohort of 40 patients after cardiopulmonary bypass (CPB) and high D-renin levels (baseline vs. 4 h after CPB, cut-off > 3.7 µU/ml, [1]) received vasopressor therapy with either norepinephrine (NE) and AT-II or standalone NE. The primary outcome was the renin plasma level at 12 h after surgery, adjusted by baseline renin plasma level at 4 h after surgery.

**Results:** Overall, the median renin plasma concentration increased from a median baseline (quartiles) of 44.3 µU/ml (14.6, 155.5) to 188.6 µU/ml (29.8, 379.0) 4 h after CPB. Patients with high D-renin were then treated with either NE alone [median (quartiles) dose of 3.25 mg (1.00, 4.75)] or with NE with additional AT-II [NE dose: 1.33 mg (0.78, 2.04)]; [AT-II dose: 0.34 mg (0.29, 0.78)]. At 12 h after surgery, renin levels were lower in patients who received AT-II, compared to patients that did not receive AT-II: 71.7 µU/ml (21.9, 211.4) vs. 130.6µU/ml (62.9, 317.0; *p* = 0.034 adjusted for baseline renin plasma level at 4 h after surgery). AT-II effects on renin levels were particularly pronounced in patients without ACEi or ARB premedication. Finally, AT-II significantly decreased the dose of NE required to maintain the target MAP (Table 1).

**Conclusions:** In cardiac surgery patients with post-OP vasoplegia and high D-renin levels, AT-II reduced renin plasma levels at 12 h compared to NE alone, which was associated with increased renin levels. Furthermore, AT-II significantly decreased the NE dose required to maintain target MAP.

**Reference**

1. Küllmar M et al. Am J Respir Crit Care Med 203:1119–26, 2021.

**Table 1 (abstract P140):** Renin characteristics

Renin	Total (n = 40)	AT-II (n = 20)	No AT-II (n = 20)	*p* value
Pre-OP levels, median (Q1, Q3), µU/ml	44 (15, 156)	45 (9, 156)	44 (17, 231)	0.745
Levels 4h post-OP, median (Q1, Q3), µU/ml	189 (30, 379)	214 (28, 461)	126 (33, 353)	0.457
Levels 12h post-OP, median (Q1, Q3), µU/ml	107 (32, 291)	72 (22, 221)	131 (63, 317)	0.213
Change, median (Q1, Q3), % (12h/4h post-OP)		−48 (−73, 40)	+14 (−42, 131)	0.023
Predicted 12h levels adjusted by 4h levels post-OP		55.7	118.0	0.034
Change without prior ACEi/ARBs premedication, %		−52 (−74, −10)	+45 (−38, 147)	0.028
Change with prior ACEi/ARBs premedication, %		−53 (−78, 84)	−8 (−46, 61)	0.336

AT-II, angiotensin-II; ACEi = angiotensin converting enzyme inhibitors; ARB = angiotensin receptor blockers

## P141

### Effects of red blood cell transfusions on renal blood flow in critically ill patients with moderate anemia

#### M Mari, A Fogagnolo, E Morelli, G Benetto, CA Volta, S Spadaro

##### Arcispedale Sant’Anna, University of Ferrara, Department of Anesthesia and Intensive Care Unit, Ferrara, Italy

*Critical Care* 2022, **26(Suppl 1):** P141

**Introduction:** The effects of red blood cell (RBC) transfusion on renal circulation are not elucidated yet. The aim of the study is to show the effects of RBC transfusion on renal blood flow, as detected by renal resistivity index (RRI) and renal venous stasis index (RVSI). Moreover, we hypothesized that RBC transfusion could be more significant in patients with higher oxygen extraction rate (O_2_ER).

**Methods:** Critically ill patients who experienced Hb levels between 7 and 9 g/dl were included in the study. RBC transfusion was decided by physician; in non-transfused patients an equivalent volume of crystalloids (i.e., 250 ml) were given. The peak systolic velocity (Vmax) and the minimal diastolic velocity (Vmin) were determined from an interlobar or arcuate artery using a posterolateral approach for kidney ultrasound. The RRI was calculated as (Vmax–Vmin)/Vmax and RVSI was calculated as (cardiac cycle time/venous flow time)/cardiac cycle time. RRI and RVSI were calculated before intervention (T_1_) and 2 h after intervention (T_2_) from both kidney and the worst values were used for analysis.

**Results:** Twenty-two patients were enrolled in the study. Hb was 8.6 [8–8.7] g/dl and SOFA was 8.5 [5–10]: twelve (55%) were transfused and ten (45%) were not. Median RRI was 0.70 [0.65–0.75] at T_1_ and 0.68 [0.66–0.70] at T_2_ (*p* = 0.39), while median RSVI was 100 [85–100] at T_1_ and 100 [94–100] at T_2_. From T_1_ to T_2_ only transfused patients lowered their RRI, from 0.71 [0.66–0.76] to 0.68 [0.66–0.70], *p* = 0.38; in the mixed model analysis RBC transfusion was significantly associated with RRI reduction (*p* = 0.47). RSVI did not changed after intervention in both groups. The size of the RBCT effect on RRI was magnified in patients with higher O_2_ER (*p* = 0.01 for interaction) (Fig. 1). AKI occurred in 3/12 transfused patients and 3/10 in non-transfused.

**Conclusions:** In patients with moderate anemia RBCT is associated with increased renal blood flow, as detected by RRI. This is particularly evident in patients with higher O_2_ER.

**Fig. 1 (abstract P141):**
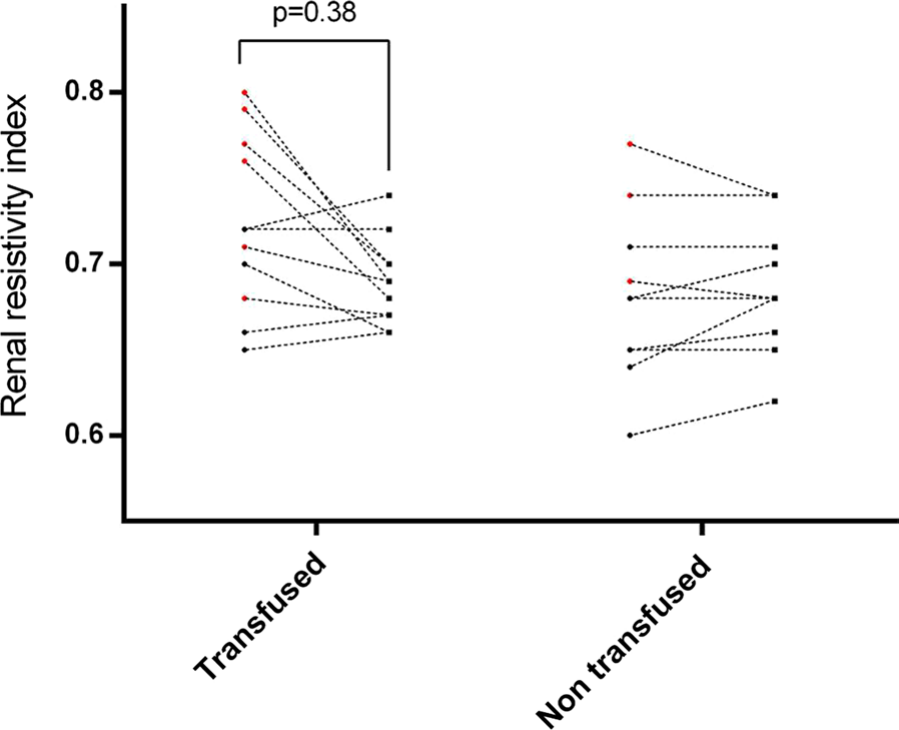
Renal resistivity Index before and after study intervention. Red points indicate patients with oxygen extraction rate > 30%

## P142

### Personalization of renal replacement therapy initiation: a secondary analysis of the AKIKI and IDEAL-ICU trials

#### F Grolleau^1^, R Porcher^1^, S Barbar^2^, D Hajage^3^, A Bourredjem^4^, JP Quenot^5^, D Dreyfuss^6^, S Gaudry^7^

##### ^1^INSERM U1153 Hôpital Hôtel-Dieu, Centre of Research in Epidemiology and Statistics, Paris, France, ^2^Nîmes University Hospital, University of Montpellier, Intensive Care Department,, Nîmes, France, ^3^Hôpital Pitié-Salpêtrière, Département de Santé Publique, Centre de Pharmacoépidémiologie, Paris, France, ^4^Dijon Bourgogne University Hospital, Clinical Investigation Center, Clinical Epidemiology/Clinical trials unit, Dijon, France, ^5^Dijon Bourgogne University Hospital, Department of Intensive Care, Dijon, France, ^6^Hôpital Louis Mourier, AP-HP, Service de Médecine Intensive-Réanimation, Colombes, France, ^7^Hôpital Avicenne, AP-HP, Service de Réanimation Médico-Chirurgicale, Bobigny, France

*Critical Care* 2022, **26(Suppl 1):** P142

**Introduction:** Trials comparing early and delayed strategies of renal replacement therapy (RRT) in patients with severe acute kidney injury may have missed differences in survival as a result of mixing together patients at heterogenous levels of risks.

**Methods:** We relied on recent guidelines [1] to evaluate the heterogeneity of treatment effect on 60-day mortality from an early vs a delayed strategy across levels of risk for RRT initiation under a delayed strategy. We used data from the AKIKI [2] and IDEAL-ICU [3] randomised controlled trials to develop a multivariable logistic regression model for RRT initiation within 48 h after allocation to a delayed strategy. We then used an interaction with spline terms in a Cox model to estimate treatment effects across the predicted risks of RRT initiation.

**Results:** We analysed data from 1,107 patients (619 and 488 in the AKIKI and IDEAL-ICU trial respectively). In the pooled sample, we found evidence for heterogenous treatment effects (*p* = 0·023). Patients at an intermediate-high risk of RRT initiation within 48 h may have benefited from an early strategy (absolute risk difference, − 14%; 95% CI, − 27% to − 1%). For other patients, we found no evidence of benefit from an early strategy of RRT initiation but a trend for harm (absolute risk difference, 8%; 95% CI, − 5% to 21% in patients at intermediate-low risk) (Fig. 1).

**Conclusions:** We have identified a clinically sound heterogeneity of treatment effect of an early vs a delayed strategy of RRT initiation that may reflect varying degrees of kidney demand-capacity mismatch.

**References**

1. Kent DR et al. Ann Intern Med 172:35–45, 2020.
2. Gaudry S, et al. N Engl J Med 375:122–33, 2016.
3. Barbar SD et al. N Engl J Med 379:1431–1442, 2018.

**Fig. 1 (abstract P142):**
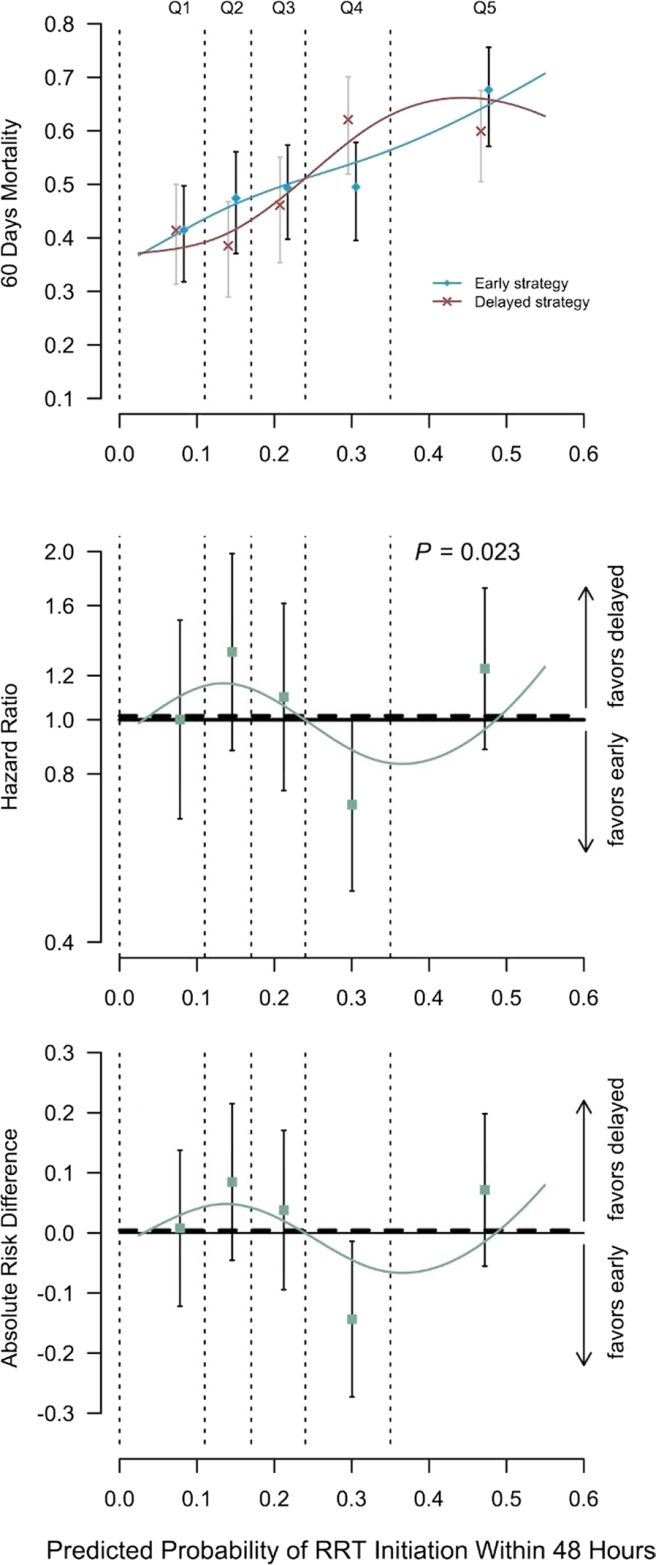
Heterogeneity of treatment effect (early vs delayed strategy) across different levels of risk of RRT initiation within 48 hours after allocation to a delayed strategy.

## P143

### Advanced organ support (ADVOS) in patients with acidosis and multiple organ failure: subgroup analysis of the registry on extracorporeal multiple organ support

#### V Fuhrmann^1^, AP Perez Ruiz de Garibay^2^, A Faltlhauser^3^, B Tyczynski^4^, D Jarczak^1^, J Weinmann-Menke^5^, M Sander^6^, A Kribben^4^, S Kluge^1^

##### ^1^Department of Intensive Care Medicine, University Medical Center Hamburg-Eppendorf, Hamburg, Germany, ^2^ADVITOS GmbH, Clinical Affairs, Munich, Germany, ^3^First Department of Internal Medicine, Kliniken Nordoberpfalz AG, First Department of Internal Medicine, Kliniken Nordoberpfalz AG, Weiden, Germany, ^4^Klinik für Nephrologie, Universität Duisburg-Essen and Universitätsklinikum Essen, Essen, Germany, ^5^Division of Nephrology, I. Department of Medicine, University Medical Center Mainz, Mainz, Germany, ^6^Department of Anesthesiology, Operative Intensive Care Medicine and Pain Therapy, University Hospital of Giessen and Marburg, Giessen, Germany

*Critical Care* 2022, **26(Suppl 1):** P143

**Introduction:** The objective of this registry (DRKS00017068) is to collect data on real-life treatment conditions for patients for whom multiple organ support with the ADVOS albumin hemodialysis is indicated. This subgroup analysis summarizes the effect of the ADVOS therapy on acid–base parameters.

**Methods:** The design of this patient’s registry has been described previously [1]. For this analysis, all the patients enrolled until August 31, 2020, were assessed for eligibility. It comprised patients for whom a complete data set for blood gas parameters including pH, serum bicarbonate, pCO2 and base excess was documented at hospital admission, immediately before (baseline) and immediately after the first ADVOS session, and after the last ADVOS session. Patients with baseline blood pH ≥ 7.35 were classified as having “no acidemia”, while patients with pH < 7.35 were subclassified into different types of acidosis.

**Results:** 240 out of 282 patients from 5 study sites in Germany were eligible. 123 had no acidemia, while 119 were subdivided into having metabolic acidosis (34), respiratory acidosis (17), mixed acidosis (62) or compensated acidemia (6). A total of 971 ADVOS treatment sessions were documented with a median of 3 (IQR: 2, 5) sessions per patient. The median duration of the ADVOS sessions was 19 h (IQR: 12, 23). Median blood flow rate was 100 ml/min (IQR: 100,150) and median dialysate pH was 7.8 (IQR: 7.4–8.4). Significant differences were observed in the latter between patients with or without acidemia at baseline (7.4 vs. 8.0). The figure shows the course of blood gas analysis from hospital admission to baseline and after the 1st and the last ADVOS treatments (Fig. 1).

**Conclusions:** Overall, an improvement in acid–base parameters could be achieved in patients with acidemia treated with ADVOS, especially in blood pH, serum bicarbonate and base excess. Moreover, in patients with respiratory acidosis, a significant PCO_2_ reduction could be observed.

**Reference**

1. Fuhrmann V et al. Medicine (Baltimore) 100:e24653, 2021.

**Fig. 1 (abstract P143):**
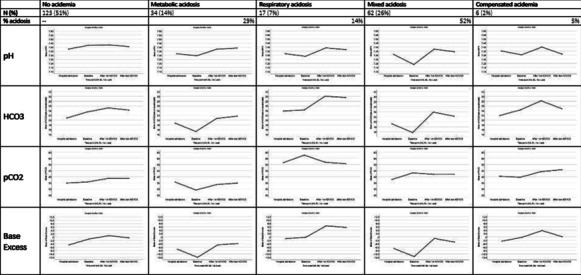
Course of blood gas analysis from hospital admission to baseline and after the 1st and the last ADVOS treatments. Subgroup analysis in different types of acidosis.

## P144

### Optimising filter lifespan in critically ill patients with coronavirus disease (COVID-19) receiving renal replacement therapy (RRT): an observational study in a UK district general hospital

#### C Evans^1^, R Sunduram^2^, J Hunter^2^, L Gemmell^2^

##### ^1^Royal Alexandra Hospital, Intensive Care Unit, Paisley, UK, ^2^Royal Alexandra Hospital, Paisley, UK

*Critical Care* 2022, **26(Suppl 1):** P144

**Introduction:** Evidence shows COVID-19 patients requiring RRT have an increased incidence of filter cartridge failure [1], this study aimed to reduce filter changes using an increased citrate dose protocol. Frequent filter changes can lead to reduced therapy delivery, increased cost and anaemia, however, the benefits of preserving filter lifespan must be balanced against the risks of anticoagulation associated bleeding.

**Methods:** A retrospective observational study was performed of our COVID-19 patients requiring RRT using an increased citrate presciption of 4 mmol/l and continuous venovenous haemodiafiltration (CVVHDF).

**Results:** The unit treated 106 patients with COVID 19 of whom 15 required RRT. 8 patients were managed exclusively on the adjusted protocol. The median duration spent on RRT was 188 h. The average lifespan of a filter in COVID-19 patient was improved from 37 to 45 h using the increased citrate protocol. There were no adverse bleeding outcomes and no documented evidence of citrate toxicity.

**Conclusions:** This small study showed an improved filter life for patients on an increased citrate dose protocol of CVVHDF without any adverse outcomes. This results in cost savings and more appropriate resource usage during a pandemic without increased bleeding risk. Another suggested measure to reduce filter malfunctions was that centres returned to using heparin anticoagulation but this is known to have increased bleeding risk [2]. Further research in a larger cohort is required but this is a promising improvement in a complex patient group.

**References**

1. Argenziano MG et al. Br Med J 369:m1996, 2020
2. Bai M et al. Intensive Care Med 41:2098–110, 2015

## P145

### Scavenging nucleus-derived molecules by polymyxin B membrane inhibits the stimulation of Toll-like receptors

#### N Takeyama, D Ohishi, T Irahara, M Tsuda, S Tanabe, M Hattori, Y Kuge, H Kano

##### Aichi Medical University, Department of Emergency and Critical Care Medicine, Aichi, Japan

*Critical Care* 2022, **26(Suppl 1):** P145

**Introduction:** The nucleus-derived molecules elicit various immunostimulatory effects via toll-like receptors (TLRs). Direct hemoperfusion with a Polymyxin B (PMX)-immobilized fiber column (Toraymyxin; Toray Industries, Tokyo, Japan) is an extracorporeal therapy developed for the treatment of endotoxemia. We hypothesize that PMX membrane can absorb nucleus-derived molecules and can block the ability of these nucleus-derived molecules to activate TLRs.

**Methods:** We first evaluated whether PMX membrane can capture and remove nucleus-derived molecules and then determined whether treatment with PMX membrane can limit the ability of nucleus-derived molecules to activate innate immune response via TLRs using reporter cell lines HEK-Blue hTLR9 and 7. The capacity of PMX-membrane absorption to DNA, RNA, cell-free neutrophil extracellular trap (NET) and nucleosome was estimated by incubation of these components with PMX-membrane. HEK-Blue hTLR7 and 9 cells were incubated with PMX-treated or -untreated nucleus-derived chromatin components. TLR7 and 9 activation of NF-κB were also measured.

**Results:** One cm^2^ PMX membrane can absorb 3 ± 0.01 mg authentic DNA within 5 min and removes DNA from incubation solution in a surface size-dependent manner. One cm^2^ PMX membrane significantly absorb 5 ± 0.02 mg authentic RNA, 6 ± 0.02 mg nucleosome and 1.3 ± 0.006 mg cell-free NET as well as DNA when compared with incubation without PMX membrane. HEK-Blue hTLRs cells showed that PMX membrane treatment significantly inhibited the ability of nucleus-derived molecules to stimulate nucleic acid-recognizing TLRs.

**Conclusions:** We demonstrated that PMX membrane can effectively capture various nucleus-derived molecules. Furthermore, scavenging nucleus-derived molecules by treatment with PMX membrane inhibited the stimulation of TLRs 9 and 7. Removing systemic nucleus-derived molecules from circulation by PMX may ameliorate irrelevant inflammatory complications in patients with sepsis and trauma.

## P146

### Clinical course and outcomes of critically ill COVID-19 patients after hemoperfusion in combination with standard therapy

#### D Somboonviboon^1^, P Aramareerak^1^, A Lertamornpong^1^, K Piyavechviratana^1^, B Satirapoj^2^

##### ^1^Division of Pulmonary and Critical Care, Phramongkutklao Hospital, Department of Medicine, Bangkok, Thailand, ^2^Division of Nephrology, Phramongkutklao Hospital, Department of Medicine, Bangkok, Thailand

*Critical Care* 2022, **26(Suppl 1):** P146

**Introduction:** Cytokine release syndrome is associated with multiple organ dysfunction in COVID-19 infection. Implementing extracorporeal blood purification could be benefit in omitting inflammatory mediators and supporting organ systems. We aims to investigate the effectiveness of hemoperfusion in combination with standard therapy in critically ill COVID-19 patients and examine factors associated with in-hospital mortality.

**Methods:** The observational study included critically ill COVID-19 patients on HA-330 hemoperfusion (Jafron Biomedical Co, Ltd). Clinical and laboratory findings were monitored after hemoperfusion. Factors associated with death after hemoperfusion were also examined.

**Results:** Fifty-five patients with COVID-19 pneumonia on hemoperfusion were analyzed. A total of 43 patients (78.2%) received mechanical ventilation and in-hospital mortality was 58.2%. Overall, mean Sequential Organ Function Assessment (SOFA) score was 8.56 ± 3.62. The hemoperfusion resulted in a significant increase in the PaO_2_/FiO_2_, white blood cell count and a significant decrease in the hsCRP and platelet counts of patients. Multi-factor Cox analysis showed increasing odds of in-hospital death associated with older age (HR 1.08, 95%CI 1.02–1.14), high body mass index (HR 1.16, 95%CI 1.07–1.26), high serum LDH level (HR 1.01, 95%CI 1.01–1.02), and high SOFA score (HR 1.26, 95%CI 1.02–1.55). Additionally, changes in patient profiles after hemoperfusion including increase in white blood cell count of > 60%, serum creatinine of > 20%, serum ferritin of > 50%, SOFA score of > 40%, norepinephrine dosage of > 25% and PaO_2_/FiO_2_ of < 50% was associated with increased risk of death.

**Conclusions:** In this study of patients with severe COVID-19, hemoperfusion therapy improve respiratory distress and cell response, and decreased inflammatory mediators. Aging, obesity, worsening in inflammatory response, renal function and no critical improving oxygenation were associated with in-hospital mortality.

## P147

### Influence of fluid therapy on kidney function in the early postoperative period after lung transplantation

#### P Nadziakiewicz^1^, M Wajda-Pokrontka^2^, A Krauchuk^3^, M Ochman^4^, P Knapik^2^, P Przybyłowski^5^

##### ^1^Silesian Centre for Heart Diseases, Cardiac Anesthesia and Intensive Therapy, Zabrze, Poland, ^2^Silesian Centre for Heart Diseases, Department of Cardiac Anesthesia and Intensive Therapy, Zabrze, Poland, ^3^Medical University of Silesia, Doctoral School, Katowice, Poland, ^4^Silesian Centre for Heart Diseases, Department of Lung Transplantation, Zabrze, Poland, ^5^Silesian Centre for Heart Diseases, Department of Cardiac Surgery, Heart Transplantation and Mechanical Circulatory Support, Zabrze, Poland

*Critical Care* 2022, **26(Suppl 1):** P147

**Introduction:** Perioperative fluid therapy among patients undergoing lung transplantation (LT) has a significant clinical importance, including development of acute kidney injury (AKI). The development of AKI in the first days after LT is associated with increased mortality in the lung transplant recipients. The aim of the study was to analyze the relationship between the volume of infused fluids, the balances of crystalloids and colloids during LT surgery and in the first 24 h and the eGFR values in the following days of the postoperative period.

**Methods:** Retrospective data analysis of 73 patients undergoing lung transplantation in 2015–2018 in our institution. Exclusion criterium was absence of 7 days observation post LTx. Deterioration of renal function was defined as the change in eGFR that occurred between baseline eGFR and the first and seventh day of observation post-LT. The CKD-EPI formula was used to calculate the eGFR value.

**Results:** The greatest decline of eGFR in the early postoperative period was demonstrated on day 7 (ΔeGFR = 75.76 ± 40.08). The decrease of eGFR on day 7 shows a weak, negative correlation both with the volume of infused colloids (r =  − 0.195, *p* = 0.309) and the volume of transfused blood products (r =  − 0.189, *p* = 0.324) during the procedure and the first day after operation. Negative crystalloid balance during the LT procedure and the first postoperative day is associated with a strong, negative correlation with decrease in eGFR on the 7th day post-LT (r =  − 0.997, *p* < 0.05).

**Conclusions:** In the analyzed population, colloids and blood products transfusions did not affect the renal function in the early perioperative period. Negative crystalloid balances in early postoperative period post-LT has a potentially protective effect on kidney function.

## P148

### The effects of fluid bolus technique with a limited volume of crystalloids on oxygen delivery: a non-inferiority study

#### H Baelongandi^1^, C Pierrakos^1^, D Velissaris^2^, R Attou^1^, K Kaefer^1^, PM Honore^1^, D De Bels^3^

##### ^1^CHU Brugmann, Intensive Care Unit, Bruxelles, Belgium, ^2^University Hospital of Patras, Intensive Care Unit, Patras, Greece, ^3^CHU Brugmann, Bruxelles, Belgium

*Critical Care* 2022, **26(Suppl 1):** P148

**Introduction:** Hemodilution after fluid bolus (FB) limits the potential increase in oxygen delivery (DO_2_). Minimizing FB volume can decrease the hemodilution effects but also the cardiac index (CI) elevations. The study aimed to assess the DO_2_ changes after FB of different technics. We speculated that FB effects on DO_2_ are not inferior when a limited crystalloid fluid volume is given within a short period than larger volumes of crystalloids or colloids given at longer periods.

**Methods:** This prospective observational study was conducted in critically ill adult patients without active bleeding and venous-to-arterial carbon dioxide tension (PvaCO_2_ > 6 mmHg) treated with FB. Arterial blood gas samples were taken, and CI was measured through transthoracic echocardiogram before and after FB. The primary endpoint was the DO_2_ changes after FB, and the secondary endpoint was Hb changes.

**Results:** Sixty patients were enrolled with P_va_CO_2_ 8.5 mmHg (IQR:7.5 to 10.2). Twenty-five patients received FB with colloids (7 ml/kg (IQR: 6.25 to 7.6)) within 30 min (IQR:28 to 39), 14 patients received FB with crystalloids (16 ml/kg (IQR: 11 to 20)) within 40 min (IQR: 28 to 40) and 21 patients crystalloids (4 ml/kg (IQR: 3.6 to 4.6)) within 20 min (infusion pump) (*p* < 0.01). FB with crystalloids 4 ml/kg increased DO_2_ 9% (IQR: − 1 to 19) compared to -2% (IQR: − 9 to 16) and 5% (IQR: − 8 to 14) in patients who received FB with higher volumes of crystalloids and colloids, respectively (*p* = 0.15) (Fig. 1). FB with crystalloids 4 ml/kg resulted to a decrease in Hb − 3% (IQR: − 6 to − 1) compared to − 7% (IQR: − 11 to − 4) and -8% (IQR: − 11 to − 5) in patients who received FB with higher volumes of crystalloids and colloids, respectively (*p* < 0.01).

**Conclusions:** Fluid bolus with a limited volume of crystalloids given within 20 min has no inferior effects on oxygen delivery and has significantly lower hemodilution effects.

**Fig. 1 (abstract P148):**
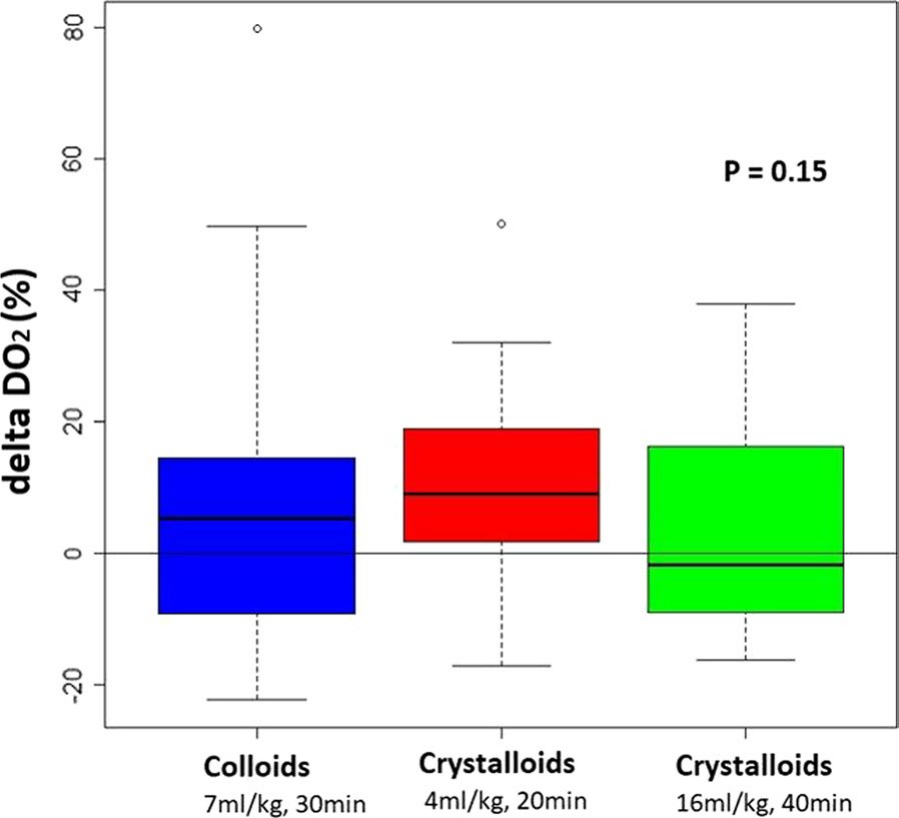
Results

## P149

### Efficacy and safety of TrachFlush artificial cough maneuver for fluid removal in simulated breathing

#### DS Karbing^1^, A Zanella^2^, SE Rees^1^

##### ^1^Aalborg University, Department of Health Science and Technology, Respiratory and Critical Care Group, Aalborg, Denmark, ^2^Universitá degli Studi di Milano, Dipartimento di Fisiopatologia Medico-Chirurgica e dei Trapianti, Milan, Italy

*Critical Care* 2022, **26(Suppl 1):** P149

**Introduction:** Suctioning through the endotracheal tube (ETT) to remove secretion has several side effects. TrachFlush (AW Technologies) applies a method for secretion removal by Zanella et al. [1] by artificial coughs (ACs) implemented by rapid ETT cuff deflation/inflation during inspiration. Peak airway flow (Q_peak_) ≥ 60 l/min is necessary to displace secretion [2]. Zanella et al. showed at peak inspiratory pressure (PIP) of 30–40 cmH_2_O AC Q_peak_ ≥ 60 l/min and safe fluid removal with no aspiration. This study evaluated TrachFlush performance at PIP of 20–40 cmH_2_O.

**Methods:** Nine scenarios were simulated with 3 PIP (20, 30 and 40 cmH_2_O) and 3 lung conditions (healthy, low compliance (C_RS_) and high resistance (R_AW_)). An artificial lung (QuickLung Breather, IngMar Medical) was ventilated in pressure control (PC) (RR = 7 min^−1^, I:E = 1:2, PEEP = 5 cmH_2_O) via a Ø = 7.5 mm ETT in an artificial trachea (25 cm, Ø = 19 mm PVC tube) with resistance 3.7 cmH_2_O/l/s at 60L/min. Q_peak_ was measured with the trachea horizontal during 5 ACs. We analysed the middle 3 ACs. Fluid removal and aspiration at PIP 20 and 30 cmH_2_O were visually assessed over 3 ACs, with trachea horizontal and 2 mL dyed saline injected between ETT cuff and lung or trachea at 45° and 2 mL saline injected above cuff, respectively.

**Results:** The Figure shows effect of PIP and lung condition (C_RS_ and R_AW_ in cmH_2_O and l/cmH_2_O/s) on Q_peak_ and AC time with Q ≥ 60 l/min. Q_peak_ ≥ 60 l/min for > 0.5 s in all scenarios. PIP and lung condition (*p* < 0.01) were important linear regression predictors for Q_peak_ (multivariate model adjusted R^2^ = 0.873, *p* < 0.001). The saline was fully removed in all scenarios, and no fluid aspiration was observed in any of the scenarios (Fig. 1).

**Conclusions:** The TrachFlush AC produced sustained and sufficient Q_peak_ for secretion removal and removed saline and avoided aspiration in all simulated scenarios.

**References**

1. Zanella A et al. Respir Care 64:372–83, 2019.
2. Volpe MS et al. Respir Care 53:1287–94, 2008.

**Fig. 1 (abstract P149):**
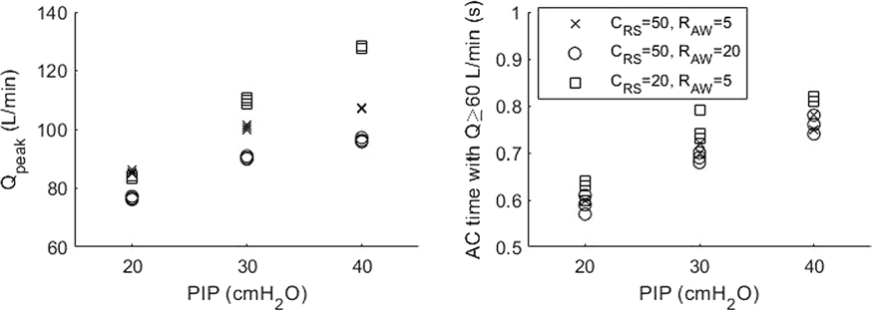
AC Qpeak and time with Q≥60 l/min vs PIP and lung condition (healthy (x), low compliance (o) and high resistance (squares)).

## P150

### Appropriate ventilator settings for the TrachFlush automated tracheal secretion removal technology: preliminary results

#### DS Karbing^1^, N Dey^2^, CG Sølling^3^, AH Nielsen^2^, SE Rees^1^, R Winding^2^

##### ^1^Aalborg University, Department of Health Science and Technology, Respiratory and Critical Care Group, Aalborg, Denmark, ^2^Gødstrup Hospital, Department of Anaesthesia and Intensive Care, Herning, Denmark, ^3^Regions Hospital Viborg, Department of Anaesthesia and Intensive Care, Viborg, Denmark

*Critical Care* 2022, **26(Suppl 1):** P150

**Introduction:** Endotracheal suctioning (ETS) is performed in patients on invasive mechanical ventilation to maintain airway patency and prevent infection, but is associated with side-effects and pain [1,2]. TrachFlush (AW Technologies) is a device automating an artificial cough (AC) method [3]. The AC is produced by rapid cuff deflation/inflation during inspiration. At respiratory rate (RR) of 7 1/min and Peak inspiratory pressure (PIP) of 30–40 cmH_2_O the original method was shown effective [3]. This study evaluated the feasibility of using bedside selected or similar PIP and RR for AC.

**Methods:** Patients were ≥ 18 years, orally intubated and ventilated in pressure control (PC) or pressure support (PS). In each patient, on clinical indication for ETS, 1–3 procedures each with 3 ACs were performed at PIP and RR as close to bedside settings as possible, allowing 1 s inspiratory time (VENT_low_). If ETS remained indicated, three ACs were performed at increased PIP and decreased RR (VENT_high_).

**Results:** Preliminary results from 18 procedures in 8 patients. Mean ± SD age, SAPS 3 and tube size were 79 ± 6 years; 75 ± 24 and 7.4 ± 0.5, respectively. Proportions of male patients, active humidification and PC/PS ventilation were 50%, 63%, 28%/72%, respectively. Fifteen AC procedures were successful, 5 out of 18 VENT_low_ (28%) and 10 out of 12 VENT_high_ (83.3%), see settings in Table. All failed procedures were in a single patient with RR 25–40 1/min. Critical-Care Pain Observation Tool (CPOT) agitation, coughing, pain and discomfort showed good tolerance to AC with improvement following AC in several procedures and deterioration following a single procedure (Table 1).

**Conclusions:** TrachFlush ACs successfully prevented ETS in 7/8 patients, using less excessive ventilator settings than originally proposed. Patients tolerated the ACs well.

**References**

1. AARC. Respir Care 55:758–64, 2010.
2. van de Leur JP et al. Crit Care 8:R467-73, 2004.
3. Zanella A et al. Respir Care 64:372–83, 2019.

**Table 1 (abstract P150):** Ventilator settings for VENTlow and VENThigh artificial coughs.

	PEEP, cmH_2_O	PC/PS, cmH_2_O	PIP, cmH_2_O	RR, 1/min
VENTlow	7.5±2.1	12.7±2.5	21.3±3.3	21.6±9.0
VENThigh	8.3±1.6	17.1±2.6	25.9±2.8	19.8±8.6

## P151

### Feasibility study on novel usage of airway ultrasound to detect subglottic secretion above endotracheal tube (ETT) cuff—SUBSUS

#### A Azma Haryaty^1^, O Adi^1^, PF Chan^1^, R Sallehuddin^2^, Z Md Yusof^3^, V Gabrielle^4^

##### ^1^Raja Permaisuri Bainun Hospital, Resuscitation and Emergency Critical Care Unit, Emergency and Trauma Department, PERAK, Malaysia, ^2^Sultanah Bahiyah Hospital, Emergency and Trauma Department, Alor Setar,Kedah, Malaysia, ^3^Raja Permaisuri Bainun Hospital, Radiology Department, PERAK, Malaysia, ^4^Cardiocentro Ticino, Cardiac Anaesthesia and Critical Care, Lugano, Switzerland

*Critical Care* 2022, **26(Suppl 1):** P151

**Introduction:** Subglottic secretion was proven as one of the causes of micro-aspiration and increase risk of ventilator-associated pneumonia (VAP). Currently, the method to detect subglottic has not been well established [1–3]. The purpose of this study is to determine the sensitivity and specificity of upper airway ultrasound (US) in the detection of subglottic secretion, in comparison to CT scan.

**Methods:** This prospective study on the upper airway US findings of 50 intubated and mechanically ventilated patients were reviewed and compared with CT scans. The CT scan findings of the presence or absence of subglottic secretion were examined by a single radiologist. The sensitivities, specificities and positive/negative predictive values (PPV, NPV) of the upper airway US findings detection of subglottic secretion were then calculated and compared with CT scan findings.

**Results:** Subglottic secretions were detected on upper airway US in 31 patients. The sensitivity and specificity of upper airway US in detecting subglottic secretion was 96.7% and 90%, respectively (PPV 93.5%, NPV 94.7%). Eighteen (58%) patients with subglottic secretions developed VAP (*p* = 0.01). The area under the receiver operating curve (AUROC) is 0.977 (95% CI 0.936–1.00) (Fig. 1).

**Conclusions:** Upper airway ultrasound is a useful tool for detecting subglottic secretion with high sensitivity and specificity.

**References**

1. Osman A et al. J Intensive Care 4:52, 2016
2. Osman A et al. Am J Emerg Med 53:23–28, 2022
3. Mahul P et al. Intensive Care Med 18:20–5, 1992

**Fig. 1 (abstract P151):**
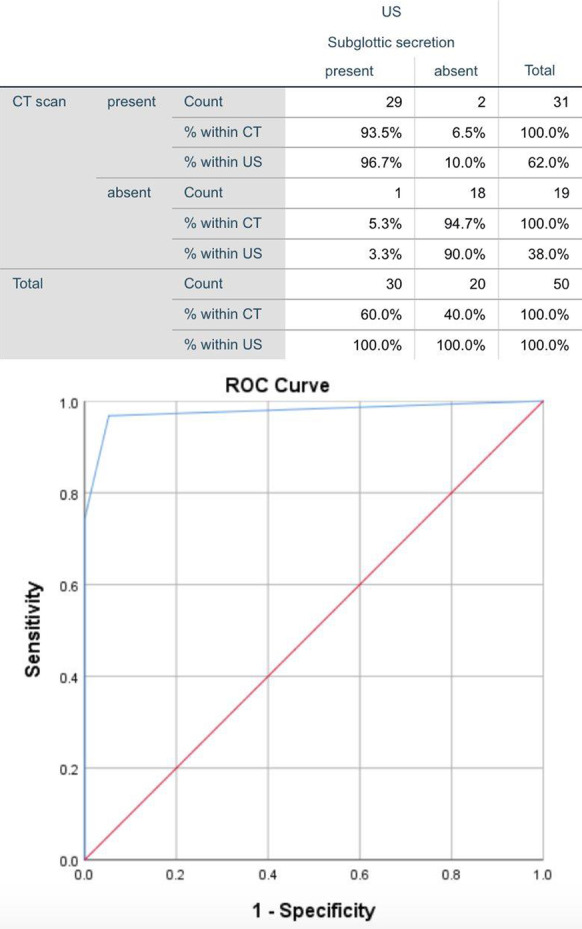
Figure shows a sensitivity, specificity, NPV, PPV US subglottic secretion with CT neck (gold standard). Area under the receiver operating curve (ROC) estimates the value of 0.977 between US Subglottic Secretion Score versus CT scan.

## P152

### McGRATH™ video laryngoscope versus Macintosh for intubation in hypoxemic COVID-19 ICU patients

#### M Hmida^1^, A Ben Souissi^1^, M Sboui^1^, S Raies^1^, M Hamdi^1^, S Ferchiche^1^, D Dallel^1^, H Cherif^1^, S Dammak^1^, MS Mebazaa^2^

##### ^1^Mongi Slim University Hospital, Anesthesiology and ICU Department, La Marsa, Tunisia, ^2^Mongi Slim University Hospital, Anesthesiolgy and ICU, La Marsa, Tunisia

*Critical Care* 2022, **26(Suppl 1):** P152

**Introduction:** Airway management and intubation are challenging in the ICU especially for COVID-19 patients with severe hypoxemia. Although recommended for COVID-19 patients, because of their capacity to reduce transmission to healthcare providers, there is no evidence that video laryngoscopes improve airway management and reduce time for intubation. The purpose of this study was to compare the McGRATH video laryngoscope and the Direct Laryngoscope (DL) in COVID-19 ICU patients with acute respiratory failure.

**Methods:** Forty patients meeting tracheal intubation criteria for respiratory failure were enrolled and equally randomized into 2 groups according to the used device: McGRATH Group and DL group. All patients had pre oxygenation with noninvasive ventilation with FiO_2_ = 1, Pep and pressure support levels were set to achieve a tidal volume of 6 ml/kg of ideal body weight. Demographic data, difficult intubation criteria were recorded. Our primary outcome was time to intubation defined as the time from the introduction of the blade in patient's mouth until the first efficient breath delivered. Secondary outcomes were the lowest SpO_2_ recorded during the procedure, the drop in SpO_2_, the number of attempts, the use of alternative methods for intubation and the experience of the operators.

**Results:** The 2 groups were comparable concerning demographic data, BMI and difficult intubation criteria (*p* = 0.091). Time to intubation was shorter in the McGRATH group with no significant difference (*p* = 0.597). The Delta SpO_2_ and the lowest SpO_2_ were similar (*p* = 0.546 and 0.458 respectively). No difference was noticed concerning the number of attempts (*p* = 0.378), the use of alternative methods (*p* = 0.276) and the operator’s skills (*p* = 0.076).

**Conclusions:** These results show that the DL is as effective as the recommended McGRATH video laryngoscope for intubation in COVID patients with severe hypoxemia.

## P153

### Airways management in SARS-CoV-2 related respiratory failure: a prospective observational multi-center study

#### L Cattin^1^, F Ferrari^2^, S Mongodi^2^, G Bettini^2^, E Pariani^2^, F Daverio^2^, F Mojoli^2^, V Danzi^1^, S De Rosa^1^

##### ^1^Ospedale San Bortolo, Anestesia e Rianimazione, Vicenza, Italy, ^2^Ospedale San Matteo, Anestesia e Rianimazione, Pavia, Italy

*Critical Care* 2022, **26(Suppl 1):** P153

**Introduction:** Emergency intubation of COVID-19 patients is a high-risk procedure and a challenge to intensivists [1,2]. The aim was to determine major adverse events related to tracheal intubation in COVID-19 patients: severe hypoxemia, hemodynamic instability and cardiac arrest.

**Methods:** This is a prospective, observational, dual-center study of COVID-19 patients undergoing advanced airway management for respiratory failure and admitted in ICU from November 2020 to May 2021. We reported data about demographics, comorbidities and parameters related to the intubation and expertise. Within 30 min from the intubation, we recorded the occurrence of severe hypoxia, cardiac arrest, hemodynamic instability. We collected data about difficult airways, the need of front of neck airways position, death within 30 min from the intubation, arrhythmia, esophageal intubation, pneumomediastinum and pneumothorax recognized within 6 h from the intubation.

**Results:** Within 142 patients considered for our analysis, 73.94% experienced at least 1 major adverse peri-intubation event. The predominant event was cardiovascular instability in 65.49% of patients, followed by severe hypoxemia (43.54%) and cardiac arrest (2.82%). First-pass success was achieved for 90.84% of patients. The rate of major adverse events was significantly lower with first-pass intubation success than for 2 attempts. No difference was found in ICU LOS between patients with a major adverse periintubation event and patients without events.

**Conclusions:** In this observational study of intubation practices in critically ill patients with COVID-19, major adverse peri-intubation events were observed frequently.

**References**

1. Yao W et al. Br J Anaesth 125:e28-e37, 2020
2. Zhang L et al. J Anesth 34:599–606, 2020

## P154

### High flow nasal oxygen and continuous positive airway pressure therapy for COVID-19: an observational study of outcomes

#### B Collins, M O’Sullivan, A Revill

##### Torbay & South Devon NHS Foundation Trust, Department of Critical Care, Torquay, UK

*Critical Care* 2022, **26(Suppl 1):** P154

**Introduction:** High flow nasal oxygen (HFNO) and continuous positive airway pressure (CPAP) therapy are recognised treatments for hypoxia which were widely used throughout the COVID-19 pandemic. Large scale studies such as RECOVERY-RS [1] compared HFNO and CPAP to conventional oxygen therapy in patients suitable for mechanical ventilation. TSDFT had capacity to offer ward based HFNO/CPAP to patients deemed both suitable and not suitable for mechanical ventilation. We set out to review the outcomes of all patients who received HFNO/CPAP for COVID-19 pneumonitis at our trust.

**Methods:** A retrospective observational study of all patients with COVID-19 Pneumonitis who received CPAP/HFNO was conducted at a district general hospital in South West England. Electronic records and ICD10 diagnostic codes were reviewed between September 2020 and October 2021.

**Results:** 90 patients received HFNO or CPAP. The median age was 68 years. 50 (55%) survived to hospital discharge. Survival to hospital discharge was greater in females (71%) than males (42%). Survival decreased from 100% in the 21–30 years age group, to 33.3% in the > 70 years age group. On review of co-morbidities the overall survival rate was similar, except for patients with cardiac failure or valvular disease, of which only 4 of 19 patients survived (21%) All patients under 40 years survived to hospital discharge. There was no relationship between number of days of therapy and survival to discharge.

**Conclusions:** Among this cohort, survival to hospital discharge after HFNO or CPAP for COVID-19 Pneumonitis was greater in younger patients, females and those without cardiovascular failure.

**Reference**

1. Perkins GD et al. medRxiv 2021.08.02.212613792021

## P155

### Non-invasive ventilation in COVID-19 patients, the experience of a level 2 unit

#### J Rua^1^, AR Nogueira^2^, JE Mateus^1^, AF Costa^3^, A Ribeiro^4^, C Silva^5^, J Trêpa^6^

##### ^1^Centro Hospitalar Universitário de Coimbra, Serviço de Medicina Intensiva, Coimbra, Portugal, ^2^Centro Hospitalar Universitário de Coimbra, Medicina Intensiva, Coimbra, Portugal, ^3^Centro Hospitalar Universitário de Coimbra, Serviço de Pneumologia, Coimbra, Portugal, ^4^Centro Hospitalar Universitário de Coimbra, Serviço de Hematologia Clínica, Coimbra, Portugal, ^5^Centro Hospitalar Universitário de Coimbra, Serviço de Medicina Interna, Coimbra, Portugal, ^6^Centro Hospitalar Universitário de Coimbra, Serviço de Doenças Infecciosas, Coimbra, Portugal

*Critical Care* 2022, **26(Suppl 1):** P155

**Introduction:** COVID-19 has a broad spectrum of severity and, although the majority of those infected are asymptomatic or have mild disease, many need hospitalization and organ support for respiratory failure. The approach to this dysfunction varied across the pandemic, influenced by retrospective data and centre experience. After initial unfavorable data, NIV resumed prominence during the 2nd wave, having been the modality of choice in our intermediate care unit (IU). We describe our NIV cohort and the results of our ventilatory strategy.

**Methods:** Descriptive retrospective study. Data were collected from electronic medical records of 202 COVID-19 patients (PTS) under NIV at the IU between September/20 and March/21. Categorical data are presented as frequency (percentage) and were compared using χ2 -test. Continuous variables were compared using Mann–Whitney U test. Statistical significance was set at *p* < 0.05.

**Results:** 202 of 469 PTS were submitted to NIV. Mean age was 66 years and 62.8% were male. Most common comorbidities were hypertension, dyslipidemia, obesity and diabetes. Mean admission SOFA score was 3.6. Most PTS underwent corticosteroid therapy, 86.7% in > 1 mg/kg dosage equivalent. Remdesivir was used in 50%. In 88.6% NIV was the initial modality of ventilatory support, 11.4% after HFNC failure (23). The preferred mode was CPAP with mean maximum pressure of 13 (6–16), titrated to normalization of the work of breathing (WOB). Mean PaO_2_/FiO_2_ ratio at start of NIV was 122, < 100 in 43% of PTS. NIV failure occurred in 35.6%, intra-unit mortality was 25.6%. 35 PTS were submitted to invasive mechanical ventilation (IMV), 41% died. Advanced age, intolerance to awake prone and delirium were associated with higher mortality.

**Conclusions:** NIV is a valid option for the management of respiratory failure secondary to COVID-19 ARDS, reducing the need for IMV. Elevated CPAP values, titrated to WOB control, complemented with prolonged periods of awake prone are essential for success.

## P156

### Regulation of a device for emergency transtracheal lung ventilation

#### D Pavlovic

##### Dalhousie University, Halifax, Dept. of Anesthesia, Pain Management and Perioperative Medicine, Halifax, Canada

*Critical Care* 2022, **26(Suppl 1):** P156

**Introduction:** In cannot-intubate, cannot-ventilate situations, a lung ventilation through a thin transtracheal cannula may be attempted [1]. However, it may be impossible to achieve sufficient ventilation if the lungs are spontaneously emptying through a thin transtracheal cannula and dangers of barotrauma may occur. Here we present a program for an automatically controlled version of a valve—a bi-directional manual respiratory pump—where a combination of low flow during inspiration, by reducing gas supply to the valve, and increased flow during expiration, by increasing gas supply to the valve, permits more effective Venturi effect and efficient expiration, with low total gas consumption.

**Methods:** The theoretical performance of the valve was modeled mathematically. The effectiveness of the valve that was predicted by the mathematical model was tested *in* *vitro* with a standard valve but by variable flow rates.

**Results:** (See the Figure) Timer–Trigger comprises of Solenoid valve 1 Oxygen, Solenoid valve 2 Output, Solenoid valve 3 Air Compressor.

1. General mode—controlled Inspiration, During expiration, the solenoid valve 1 (oxygen) is closed (to save oxygen). With the solenoid valve 3 (air) open, the compressor air at the constant start ensures expiratory aid.
2. Security—Assist mode (Trigger) Safety—Over-Pression: Forced Expiration
3. Safety—Negative Pressure—Trigger: +—Forced Inspiration, Safety—Apnea—Forced Inspiration
4. CICV mode the solenoid valve 2 and 3 (air compressor tube—ensures extra start during expiration) are the same (Fig. 1).

**Conclusions:** Satisfactory lung ventilation can be assured with transtracheal ventilation with a bidirectional manual respiration valve with variable gas flow.

**Reference**

1. Meissner K et al. Anesthesiology 109:251–9, 2008

**Fig. 1 (abstract P156):**
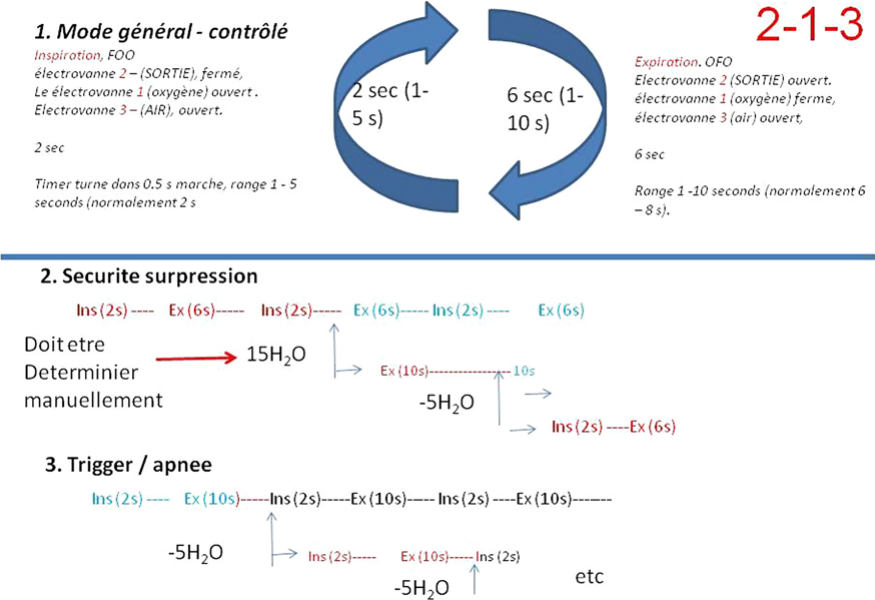
Algorithm

## P157

### Use of ROX index in predicting the failure of non-invasive respiratory support for patients with COVID-19

#### JT Thompson^1^, TS Sage^2^, AM Morrison^2^, AR Roy^2^, PM McAndrew^2^, AR Rostron^3^

##### ^1^Sunderland Royal Hospital, Integrated Critical Care Unit, Sunderland, UK, ^2^Sunderland Royal Hospital, Sunderland, UK, ^3^Translational and Clinical Research Institute, Newcastle University, Newcastle upon Tyne, UK

*Critical Care* 2022, **26(Suppl 1):** P157

**Introduction:** COVID-19 patients are at risk of respiratory deterioration requiring higher level of care. Decisions around timing of intubation and invasive ventilation remain a challenge. NEWS2 is a well-established physiology scoring system used to detect the deteriorating patient [1]. New evidence suggests ROX index may be more reliable than NEWS2 to identify patients at risk of treatment failure of non-invasive respiratory support (NIRS) [2]. Another study has suggested the use of a nomogram for predicting NIRS failure [3].

**Methods:** Data were collected retrospectively from 81 COVID-19 patients admitted to a general critical care unit. Vasopressor use, comorbidities and worst physiological parameters in the first 24 h of instituting NIRS were recorded and used to calculate NEWS2, ROX index, nomogram scores and P/F ratio. NIRS failure, length of therapy and survival status at the end of critical care admission were recorded.

**Results:** Area under the receiver operating characteristic (AUROC) curves were calculated for NIRS failure prediction. For nomogram AUROC was 0.701 (95% CI 0.584–0.818) *p* = 0.0033, ROX index AUROC 0.810 (95% CI 0.708–0.908) *p* =  < 0.0001, NEWS2 AUROC 0.688 (95 CI 0.574–0.802) *p* = 0.0051, P/F AUROC 0.748 (95% CI 0.638–0.858).

**Conclusions:** ROX index is an easily calculated score and a better predictor of NIRS failure than nomogram, NEWS2 scores and P/F ratio. NEWS2 is not calibrated for this patient population and is not specific for those requiring respiratory support. The ROX index is easier to calculate than a recently developed nomogram [3] and performs better. Patients in respiratory support units (RSU) in the United Kingdom do not have arterial lines sited routinely. ROX-index would therefore be a useful score to help predict treatment failure of NIRS in RSU’s and facilitate decision making for escalation of care.

**References**

1. Pimental M et al. Resuscitation 134:147–156, 2018.
2. Prower E et al. E Clinical Medicine 35:100,828, 2021.
3. Liu L et al. Lancet 3:e166–174, 2021.

## P158

WITHDRAWN

## P159

### Optimizing the effect of inhaled nitric oxide therapy of COVID-19 patients with acute respiratory failure

#### F Almutairi

##### King Fahd Armed Forces Hospital, Respiratory Care, Jeddah, Saudi Arabia

*Critical Care* 2022, **26(Suppl 1):** P159

**Introduction:** Inhaled nitric oxide (NO) is a pulmonary vasodilator that is inhaled and works by relaxing smooth muscle to dilate blood vessels in the lungs. It is used together with a ventilator to treat acute respiratory failure. Severe acute respiratory syndrome coronavirus, which is responsible for COVID-19 pandemic, caused a massive influx of patients presenting with acute respiratory distress syndrome (ARDS). This study aims to improved percentage of oxygenation of COVID-19 patients with acute respiratory failure using nitric oxide therapy by more than 50% by the end of 2021.

**Methods:** The data were collected using ISBAR (Introduction—Situation—Background—Assessment—Recommendation) hand over tool and arterial blood gas results from RAPIDCOMM blood gas server from the months of May 2021 to October 2021. Data collected includes date of intubation and extubation, start date and end date of iNO treatment, blood gases measurements before, during and after iNO treatment, dose of inhaled nitric oxide in PPM (parts per million), total number of days in iNO therapy.

**Results:** Based on the patients data collected from May to October 2021, 91–95% of COVID-19 patients who were treated with iNO in the months of May, June, July showed improvement in oxygenation and were wean-off from ventilator (Fig. 1). During the month of August 2021, influx of ARDS due to COVID-19 became twice as much the number compared from the previous months and it was observed during the months of August and September 2021. Number of patients without improvement in oxygenation has increased due to several factors such as availability of nitric oxide (NO) machine and co-morbidities like chronic renal failure.

**Conclusions:** Our data indicates that the utilization of iNO is useful in improving arterial oxygenation of COVID-19 patients and could provide immediate help and delay of respiratory deterioration and further complications.

**Fig. 1 (abstract P159):**
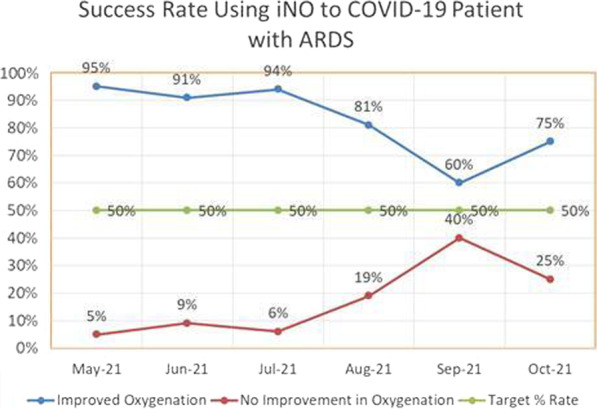
Results

## P160

### High flow nasal oxygen in the intensive care setting during the COVID-19 pandemic at Mater Dei Hospital, Malta

#### C Mizzi, M Drake, S Sciberras, M Buttigieg

##### Mater Dei Hospital, Intensive Care, Msida, Malta

*Critical Care* 2022, **26(Suppl 1):** P160

**Introduction:** The aim of this study was to describe the use of high flow nasal oxygen (HFNO) in COVID-19 intensive care unit (ICU) patients [1] locally, and establish their demographics and outcomes. Mater Dei Hospital is the only main acute general hospital on the island. It houses a 20-bedded adult ICU catering for a population of 500,000.

**Methods:** We conducted a single-centre prospective observational cohort study at the ICU at Mater Dei Hospital in Malta between March 2020 and May 2021. Data collected included use of HFNO, mechanical ventilation (MV), duration of MV, length of stay, and 28-day survival.

**Results:** 240 COVID-19 ICU patients were included. 108 (45%) received HFNO for a median of 3 days, the rest received MV for a median of 12 days. No major differences in demographics were noted (age: 66.5 vs 68 years, *p* = 0.225; 70% male, 30% female vs 79% male, 21% female, *p* = 0.191). Forty-two (38.2%) patients failed HFNO after a median of 2 days, needing MV for a median of 10 days (*p* < 0.001). Median length of stay was lower in HFNO patients (6 vs 13 days; *p* < 0.001). 28-day survival was highest in the HFNO-only group (94%), followed by the HFNO + MV group (61%), and finally the MV-only group (52%; *p* < 0.0001). This is not simply due to severity since FiO_2_ was higher for HFNO patients and PaO_2_ tended to be lower. Cox proportional hazards analysis showed that respiratory support was more significant than admission P/F ratios, PaO_2_s, or SOFA, with MV being linked to a hazards ratio of 8.4 (*p* < 0.001) when adjusted for the above criteria.

**Conclusions:** HFNO offers considerable practical advantages over MV. Avoiding MV might be linked to a reduced incidence of ventilator-associated pneumonias, shorter ICU stay and lower mortality. It is also a safe tool to use and the risk of aerosolization should not deter from its use.

**Reference**

1. Mellado-Artigas R et al. Crit Care 25:58, 2021

## P161

### Duration of noninvasive continuous positive airway pressure as a risk factor for mortality in patients with COVID-19 acute respiratory failure.

#### N De Vita^1^, L Scotti^1^, F Racca^2^, G Airoldi^3^, C Olivieri^4^, E Santangelo^1^, L Castello^1^, F Della Corte^1^, P Navalesi^5^, R Vaschetto^1^

##### ^1^Eastern Piedmont University, Translational Medicine, Novara, Italy, ^2^Azienda Ospedaliera SS. Antonio e Biagio e Cesare Arrigo, Department of Anesthesia and Intensive Care, Alessandria, Italy, ^3^Ospedale Ss. Trinità, Medicina Interna, Borgomanero, Italy, ^4^Azienda Ospedaliera Sant’Andrea, Department of Anesthesia and Critical Care, Vercelli, Italy, ^5^Università di Padova, Dipartimento di Medicina—DIMED, Padova, Italy

*Critical Care* 2022, **26(Suppl 1):** P161

**Introduction:** Recent experiences suggest that noninvasive continuous positive airway pressure (CPAP) ventilation may be an effective alternative to mechanical ventilation in COVID-19 respiratory failure. However, in patients who failed CPAP, delayed intubation may increase the risk of mortality. We made a comparison between the patients admitted during the first and the second wave of the pandemic who failed CPAP and required mechanical ventilation.

**Methods:** We retrospectively included all consecutive patients admitted to one of the four participating hospitals from March 1st to April 15th, 2020, and from November 1st to December 15th, 2020, with the following inclusion criteria: (1) age ≥ 18 years, (2) diagnosis of moderate to severe COVID-19 pneumonia treated with CPAP outside ICU, (3) intubation after CPAP failure. Patients who received post-extubation CPAP were excluded. We collected data about CPAP duration prior intubation, hospital length of stay and in-hospital mortality.

**Results:** A total of 193 COVID-19 patients received intubation after CPAP failure during the first (n = 127) and the second (n = 66) wave. During the second wave, CPAP treatment was longer (4 (2–8) vs. 3 (2–5) days; *p* < 0.05) as well as hospital length of stay (20 (15–29) vs. 12 (6–30) days; *p* < 0.05) with an increased in-hospital mortality (62% vs. 35%; *p* < 0.001). The univariable analysis showed that CPAP duration was a risk factor for mortality in patients failing CPAP during the second wave and in the overall population [HR 1.080 (95% C.I. 1.007–1.159) and HR 1.071 (95% C.I. 1.032–1.112), respectively. The multivariable model adjusted for centre, wave, age, gender, comorbidity, white blood cell count, and creatinine confirmed this results HR 1.117 (95% C.I. 1.029–1.214) during the second wave, and HR 1.077 (95% C.I. 1.025–1.131) in the overall patients.

**Conclusions:** Our results confirmed that in COVID-19 patients failing CPAP performed outside ICU the risk of death increased with the days spent on noninvasive ventilation.

## P162

### Tracheostomy experience during SARS-CoV-2 pandemic in a hospital from Bogotá, Colombia

#### EE Rodríguez^1^, NA Pedraza Lopez^1^, R Toro Manotas^2^, E Valencia Morera^2^, J Perez Pinzon^2^, E Reyes^1^, F Medina^1^, YR Cárdenas^1^

##### ^1^Fundación Santa Fe de Bogotá, Intensive Care Department, Bogotá, Colombia, ^2^School of Medicine, Universidad de los Andes, Intensive Care Department, Bogota, Colombia

*Critical Care* 2022, **26(Suppl 1):** P162

**Introduction:** Viral pneumonia is the main complication of SARS-Cov-2 infection. Many patients require prolonged invasive mechanical ventilation and subsequent tracheostomy [1]. There is no standard recommendation about the optimal timing for the procedure [2,3]. The aim of this study was to describe the clinical outcomes in COVID-19 patients who underwent early versus late tracheostomy.

**Methods:** A retrospective single-center observational descriptive study was performed at a fourth level hospital on patients with confirmed diagnosis of COVID-19 and admission to the ICU who required mechanical ventilation and subsequent tracheostomy between January and July of 2021. Group analysis by the timing of tracheostomy since the start of mechanical ventilation was done in two sets: until day 14 (group 1) and from day 15 onward (group 2). The measured outcomes were hospital length of stay (LOS), ICU length of stay (ICLOS) and overall mortality.

**Results:** 151 patients were included, almost all patients required ICU due to respiratory failure (96%). 42 patients conformed group 1 and 109 patients were included in group 2. Baseline characteristics are shown in Table 1. Mortality (50% vs 56,8%; *p* = *0.4*) was not statistically different between groups. However, LOS (33.5 IQR 27.3 vs 43 IQR 20; *p* = 0.003) and ICLOS (25 IQR 19 vs 32 IQR 19; *p* < 0.001) were shorter in group 1.

**Conclusions:** Optimal timing for tracheostomy in critically ill COVID-19 patients is still undefined. Our study showed similar results found in other populations [4,5]. ICLOS and LOS seem to be shorter in early tracheostomy group. Other outcomes such as morbidity, time to decannulation and the possibility of early pulmonary rehabilitation should be included in future studies.

**References**

1. Rappoport W et al. Rev Cirugia 72:449–54, 2020.
2. Cheung N et al. Respir Care 59:895–915, 2014.
3. Freeman B. Crit Care Clin 33:311–22, 2017.
4. Kuno T et al. Indian J Otolaryngol Head Neck Surg, 2021 https://doi.org/10.1007/s12070-021-02966-2.
5. Kwak P et al. JAMA Otolaryngol Head Neck Surg 147:239–44, 2021.

**Table 1 (abstract P162):** Baseline characteristics

	Group 1 (≤14 days) n = 42	Group 12 (≥15 days) n = 109	*p* Value
Age	56.6 (IQR 29.8)	63 (IQR 12)	0.015*
BMI	27.6 (IQR 4.83)	27.8 (IQR 5.78)	0.7
APACHE II score	13.5 (IQR 7)	12 (IQR 7)	0.3
SOFA score	6 (IQR 3.75)	4 (IQR 4)	0.023*
PAFI	180 (IQR 69)	184 (IQR 78)	0.9
Sex	Women: 16 (38%) Men: 26 (62%)	Women: 42 (38.5 %) Men: 67 (61.5%)	0.9
Hemodynamic support at tracheostomy	9 (21.5%)	40 (36.5%)	0.07

## P163

WITHDRAWN

## P164

### Experience in performing a tracheostomy in patients with COVID-19 in the intensive care unit of a high complexity hospital in the south of Colombia

#### LE Sanabria^1^, AM Luna-Florez^1^, MC Sanabria^2^, JD Charry^3^, A Muñoz^1^

##### ^1^Universidad SurColombiana, Critical Care, Neiva, Colombia, ^2^Universidad De Los Andes, Medicine, Bogotá DC, Colombia, ^3^Universidad SurColombiana, Neiva, Colombia

*Critical Care* 2022, **26(Suppl 1):** P164

**Introduction:** COVID-19 is an infectious disease caused by the coronavirus SARS-CoV-2. It mainly affects the respiratory tract and may progress to acute respiratory distress syndrome (ARDS), which requires management through orotracheal intubation and mechanical ventilation which, in many cases, requires performing a tracheostomy during the patient’s stay in the Intensive Care Unit. The objective of this study was to describe the indications, completion times, complications and outcomes of adult patients with SARS-CoV-2 COVID-19 who underwent tracheostomy in the Intensive Care Units of the Hospital Universitario de Neiva.

**Methods:** A descriptive observational study was carried out in 164 patients with history of PCR-confirmed SARS-CoV-2 COVID-19 infection, who underwent tracheostomy in the Intensive Care Units of the University Hospital of Neiva. The information was collected through epidemiological and clinical criteria using an electronic form. 3 groups of intervention were established at 21, 14 and 10 days post-intubation; a general analysis was performed, as were univariate and multivariate analysis per subgroups.

**Results:** Of the 164 patients with COVID-19 diagnosis, 62% were men, the mean age was 68.4 ± 2.6 years. The main comorbidities described were Obesity, HBP, COPD and DM II. The times of completion of the tracheostomy were at 21 (n = 61), 14 (n = 49), and 10 (n = 54) days post-intubation. 39 (23.8%) patients presented complications, 82% of which were from the 21 and 14 days groups. It was found that the patients who underwent tracheostomy at 10 days survival was higher compared to the other two groups with a *p* value < 0.05.

**Conclusions:** In patients with COVID-19 infection that require prolonged mechanical ventilation during their stay in the Intensive Care Unit, it is suggested to perform a tracheostomy in the 10-day protocol since it is associated with higher survival and a lower rate of complications.

## P165

### Lateral positioning as a new lung recruitment maneuver: an experimental ARDS model study

#### G Alcala^1^, M Tucci^2^, M Mlček^3^, M Otáhal^2^, JB Borges^3^, M Ricl^3^, R Roldán^2^, S Gomes^2^, M Amato^2^

##### ^1^University of São Paulo, Pulmonary Division, Heart Institute (INCOR), Sao Paulo, Brazil, ^2^University of São Paulo, Sao Paulo, Brazil, ^3^Charles University, Prague, Czech Republic

*Critical Care* 2022, **26(Suppl 1):** P165

**Introduction:** Recruitment maneuvers (RM) are designed to open collapse lungs and keep them opened, based on the application of high pressures during a period of time. However, they expose the patient to hemodynamic instability and their beneficial effects are debatable. Lateral positioning does not require the application of higher airway pressures and could promote alveolar recruitment due to changes in regional transpulmonary pressure.

**Methods:** Six pigs were submitted to invasive mechanical ventilation under general anesthesia. A lung injury model was produced by sequentially applying lung lavage followed by injurious ventilation to obtain a PF ratio < 100 mmHg. After injury, a RM was performed and open-lung-PEEP (lowest PEEP with collapse < 1%) was chosen during a decremental PEEP titration guided by EIT. After observing massive collapse at PEE *p* = 5 cmH_2_O, animals were ventilated with VT = 6 ml/kg, RR 25 bpm and optimal PEEP without recruitment maneuver. All animals were sequentially positioned at (1) supine position, (2) tilted to the left (down), (3) back to supine, (4) tilted to right (down) during 20 min, and finally back to (5) supine. We evaluated the end expiratory lung impedance (EELZ), arterial blood gases, and respiratory mechanics. Paired T tests were performed to compare the first supine with the last supine position.

**Results:** all animals presented an increase in respiratory compliance (*p* = 0.008; Fig. 1A) and PaO_2_/FiO_2_ ratio (*p* = 0.009; Fig. 1C). In five of six animals EELZ increased (*p* = 0.07), while one animal lost aeration probably due to insufficient PEEP (Fig. 1B). The amount of mass of collapsed lung measured by EIT decreased after the lateral positioning (*p* = 0.02; Fig. 1D). Adverse hemodynamics effects were not observed.

**Conclusions:** Sequential lateral positioning effectively works as a recruitment maneuver, opening collapsed lung areas, improving respiratory mechanics, oxygenation and EELZ, without requiring high airway pressures and presenting no adverse hemodynamics effects.

**Fig. 1 (abstract P165):**
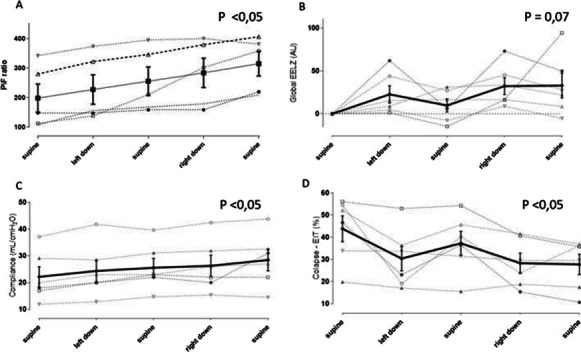
Changes, during sequential lateral position, in PaO_2_/FiO_2_ ratio (1A), global end expiratory lung impedance (EELZ) (1B), respiratory compliance (1C) and mass of collapsed lung (%) estimated by EIT (1D). A significant improvement in PaO_2_/FiO_2_ ratio, respiratory compliance and mass of collapsed lung was observed when comparing the first supine position with the final supine position. The gray lines represent each of the 6 animals and the black line show the mean values and standard error (SE) of the 6 animals. Paired T tests were performed to compare the first supine with the last supine position.

## P166

### Lateralization can work as a recruitment maneuver without hemodynamic side effects; but its success depend on PEEP

#### G Alcala^1^, AC Dos Santos^2^, M Tucci^2^, C Lima^2^, E Rodrigues^2^, S Gomes^2^, M Mlček^3^, E Kuriscak^3^, JB Borges^3^, M Amato^2^

##### ^1^University of São Paulo, Pulmonary Division, Heart Institute (INCOR), Sao Paulo, Brazil, ^2^University of São Paulo, Sao Paulo, Brazil, ^3^Charles University, Prague, Czech Republic

*Critical Care* 2022, **26(Suppl 1):** P166

**Introduction:** Deleterious hemodynamic effects of recruitment maneuvers (RM) has caused some concern. Lateral positioning changes the distribution of forces within the lung, increasing the transpulmonary pressures of the lung repositioned upwards, potentially working as a RM for this upper lung. By alternating this procedure between both lungs, we could promote bilateral recruitment without the need of high airway pressures.

**Methods:** We enrolled six animals with lung injury and mechanically ventilated. The open-lung-PEEP level was chosen by decremental PEEP titration guided by EIT (lowest PEEP generating < 3% collapse). After promoting massive lung collapse at low PEEP (5 cmH_2_O), all animals were kept in supine position, with PEEP slowly raised up to open-lung-PEEP without RM. Keeping the same PEEP, they were tilted to left lung down, back to supine, tilted to right lung down, and back to supine, 20 min in each position. Four of the six animals repeated the same sequence at a lower PEEP (6 cmH_2_O lower than open-lung PEEP). P/F ratio were measured at the end of each 20-min period and lung volume (EELZ) was continuously recorded by EIT.

**Results:** Sequential lateral turns at open-lung PEEP resulted in significant improvements in P/F ratio (mmHg) (*p* < 0.05; Fig. 1A) and EELZ (*p* = 0.07; Fig. 1B). In contrast sequential lateral turns at lower PEEP did not increase P/F ratio (mmHg) (*p* > 0.05; Fig. 1C). and resulted in decreased EELZ after each step of the lateralization (*p* = 0.04; Fig. 1D).

**Conclusions:** Sequential turns of lateral position can work as a traditional recruitment maneuver, resulting in better oxygenation and lung aeration, and not causing any deleterious hemodynamic effect. The success of the procedure depends on some interaction with PEEP levels: lateralization at lower PEEP seems to promote more collapse at the lung repositioned downwards. In contrast, lateralization at open-lung PEEP promotes progressive recruitment of the lung repositioned upwards (but keeping the lower lung stable).

**Fig. 1 (abstract P166):**
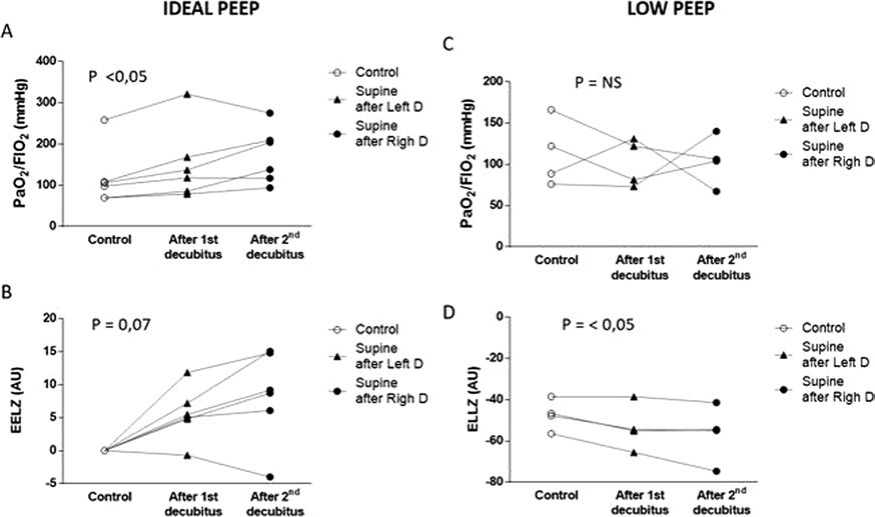
Changes, during sequential lateral position, in PaO_2_/FIO_2_ ratio (1A; 1C), global end expiratory lung impedance (EELZ) (1B; 1 D) estimated by EIT in ideal PEEP and low PEEP. Paired T tests were performed to compare the first supine with the last supine position. IDEAL PEEP presented increase in P/F ratio and EELZ. The LOW PEEP presented decrease in EELZ in P/F ratio and EELZ.

## P167

### A systematic review and meta-analysis of respiratory system mechanics and outcomes in mechanically ventilated patients with COVID-19 related acute respiratory distress syndrome

#### RR Ling^1^, MP Reddy^2^, A Subramaniam^3^, C Chua^3^, C Anstey^4^, K Ramanathan^5^, K Shekar^6^

##### ^1^Yong Loo Lin School of Medicine, National University of Singapore, Singapore, Singapore, ^2^Calvary Hospital, City: ACT, Department of Intensive Care Medicine, ACT, Australia, ^3^Peninsula Health, Australia, Department of Intensive Care Medicine, VIC, Australia, ^4^University of Queensland, Brisbane QLD, Australia, ^5^National University Heart Centre, National University Hospital, Cardiothoracic Intensive Care Unit, Singapore, Singapore, ^6^The Prince Charles Hospital, Adult Intensive Care Services and Critical Care Research Group, Brisbane, QLD, Australia

*Critical Care* 2022, **26(Suppl 1):** P167

**Introduction:** The respiratory mechanics, particularly static compliance of the respiratory system (C_RS_) in COVID-19 acute respiratory distress syndrome (CARDS) is poorly understood. Whether or not distinct ARDS phenotypes based on C_RS_ exist is still widely debated.

**Methods:** We conducted a systematic review and meta-analysis, searching three international databases from 1st December 2019 to 15th July 2021 for studies reporting on the respiratory mechanics of patients with CARDS. The primary outcome was the C_RS_ of both COVID-19 ARDS. Secondary outcomes included the mortality rates, lengths of stay, and ventilator free days. Random-effects (DerSimonian and Laird) meta-analyses were conducted.

**Results:** 45 studies (13,334 patients) were included for analysis. The pooled C_RS_ in patients mechanically ventilated for COVID-19 was 34.6 (95%-CI: 33.4–35.8), and displayed a normal distribution (Shapiro–Wilk test: *p* = 0.35). C_RS_ was significantly associated with an PaO_2_/FiO_2_ ratio, positive end-expiratory pressure, and tidal volume; driving pressure was negatively associated with C_RS_. The pooled mortality rate was 36.2% (95%-CI: 30.3–42.4%, ICU) and 38.9% (95%-CI: 32.3–45.7%, 28-day).

**Conclusions:** The respiratory mechanics of CARDS at the time closest to the initiation of invasive mechanical ventilation was normally distributed and did not reveal any distinct C_RS_-based phenotypes. However, to what extent the proposed unique pathophysiology of CARDS affects the current definition of ARDS and “exposes” its potential limitations remains a question for a high-quality, large prospective dataset to answer. Nonetheless, from our study-level analysis, C_RS_ appears to be a heterogenous metric affected by both disease and intervention factors (Fig. 1) and physicians should treat patients with personalised and precise interventions in this context.

**Fig. 1 (abstract P167):**
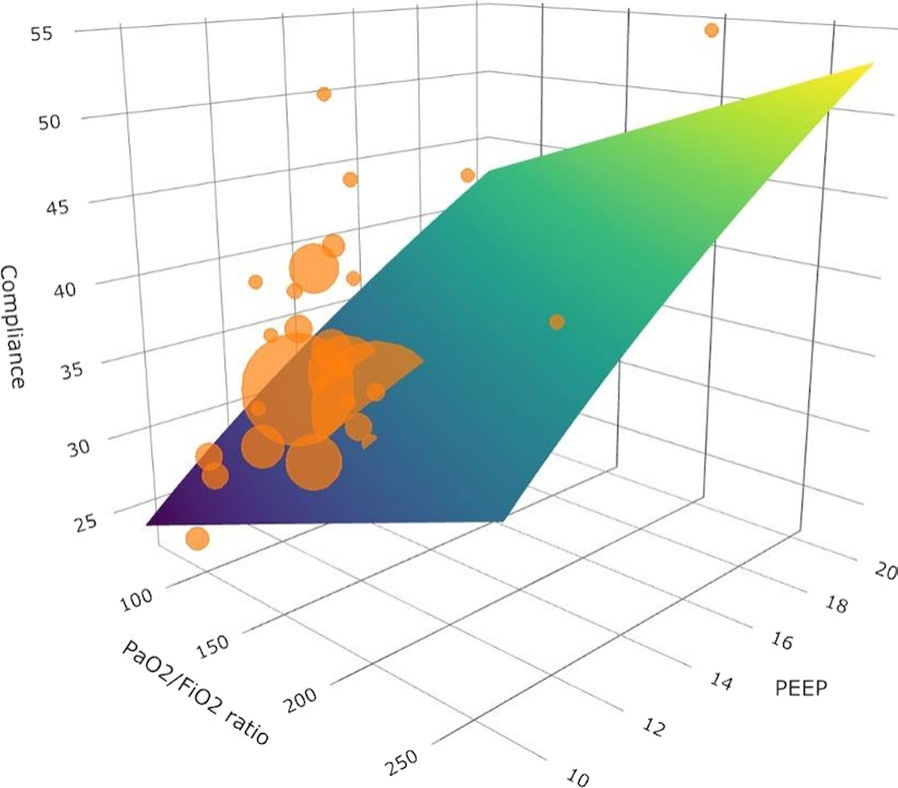
Respiratory static compliance is significantly associated with PaO_2_/FiO_2_ ratio and positive end-expiratory pressure

## P168

### A novel definition and treatment of hyperinflammation in COVID-19 based on purinergic signalling

#### D Hasan^1^, A Shono^2^, CK Van Kalken^1^, PJ Van der Spek^3^, T Kotani^2^

##### ^1^Independent researcher, Kasterlee, Belgium, ^2^School of Medicine, Showa University, Anaesthesiology and Critical Care Medicine, Tokyo 142–8666, Japan, ^3^Erasmus MC, Erasmus Universiteit Rotterdam, Pathology & Clinical Bioinformatics, 3015 CE Rotterdam, Netherlands

*Critical Care* 2022, **26(Suppl 1):** P168

**Introduction:** Hyperinflammation plays an important role in severe COVID-19. Using inconsistent criteria, researchers define hyperinflammation as a form of very severe inflammation with cytokine storm. Our paper gives a novel definition. Subsequently, we describe the treatment of ICU-patients with COVID-19 requiring ECMO and/or mechanical ventilation.

**Methods:** We searched scientific articles on P2X7 purinergic receptors (P2X7Rs) to underpin our definition of hyperinflammation. We found that lidocaine can block P2X7Rs. The issue is that the half-maximal effective concentration of lidocaine for P2X7R inhibition is much higher than the maximal tolerable plasma concentration. To overcome this, we selectively inhibit the P2X7Rs of the cells of the lymph nodes. We do this by subdermal infusion of lidocaine HCL inducing clonal expansion of Tregs in local lymph nodes. Secondarily, these Tregs migrate throughout the body suppressing systemic hyperinflammation (Fig. 1). We treated six COVID-19 ICU-patients with subdermal lidocaine infusion (1 mg/kg/h).

**Results:** We found 437 articles to underpin our definition of hyperinflammation. The essence is that hyperinflammation is initiated when SARS-CoV-2 infection causes prolonged and vigorous activation of the P2X7Rs of the immune cells. This leads to cytokine storm and desensitisation of purinergic receptors of immune cells other than the P2X7Rs, resulting in immune paralysis with secondary infections. The six ICU-patients with COVID-19 we treated with lidocaine all recovered completely.

**Conclusions:** Applying consistent criteria, we defined hyperinflammation as prolonged and vigorous activation of P2X7Rs of the immune cells and established that selective inhibition of these receptors can calm down cytokine storm in COVID-19. Our experience with subdermal administration of lidocaine in the ICU made clear that this method may not be suitable outside hospitals. Therefore, we developed a novel oral transmucosal administration route using xylocaine 10% spray, as shown in the Figure.

**Fig. 1 (abstract P168):**
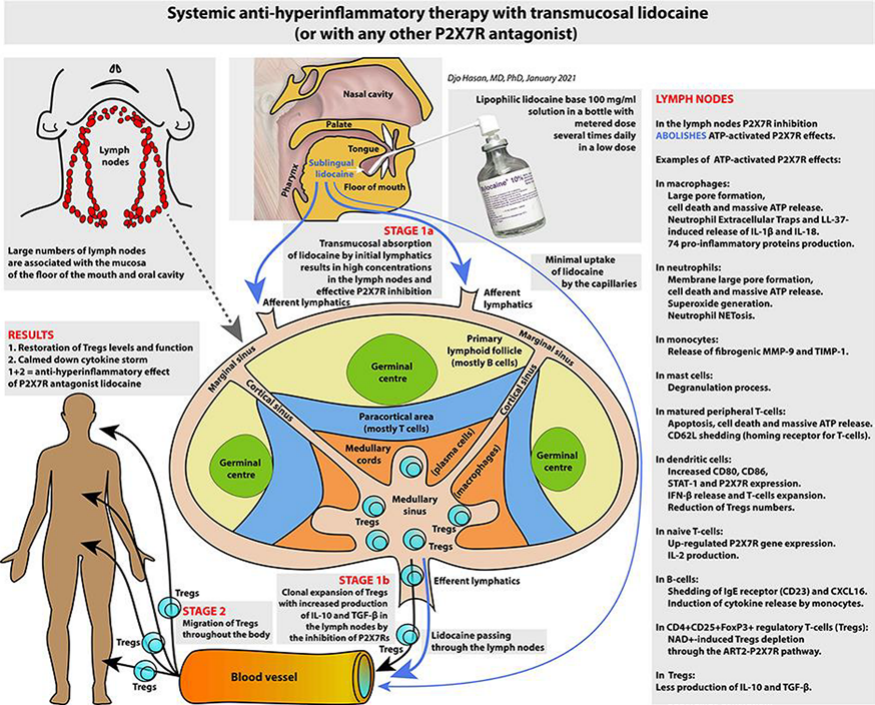
A graphical summary of the future development of the administration of lipophilic lidocaine base in the sublingual region or elsewhere in the oral cavity. We postulate that selective inhibition of the P2X7Rs of the immune cells of the lymphatic system by lidocaine suppresses hyperinflammation in two stages. Stage 1: The selective inhibition of the P2X7Rs of the immune cells residing in the lymph nodes (stage 1a) induces clonal expansion of Tregs with improved function in these lymph nodes (stage 1b); Stage 2: Subsequently, these Tregs migrate throughout the body exerting anti-inflammatory activities reducing systemic and (distant) local hyperinflammation. Source: Hasan D, et al. (2021) https://doi.org/10.1007/s11302-021-09814-6, with permission.

## P169

### Non-acidotic hypercapnia limits the loss of diaphragm force at single fibers level in mechanically ventilated rats for 5 days

#### N Cacciani^1^, A Addinsall^2^, L Larsson^3^

##### ^1^Karolinska Institutet, Department of Physiology and Pharmacology (FyFa), Stockholm, Sweden,^2^Karolinska Institutet, Department of Clinical Neuroscience, Karolinska Institutet, Stockholm, Sweden, ^3^Viron Molecular Medicine Institute, Boston, MA, USA

*Critical Care* 2022, **26(Suppl 1):** P169

**Introduction:** Acute exposure to hypercapnia has shown benefit to diaphragm function in porcine and rat ICU models of mechanical ventilation (MV), within 72 h. Here, we hypothesize that normoxic and non-acidotic hypercapnic conditions (NAHCs) would limit the loss of diaphragm function during MV for longer duration.

**Methods:** Adult Sprague–Dawley rats were deeply sedated, pharmacologically paralyzed, hydrated, nourished and controlled mechanical ventilated (CMV) for 5 days in normoxic-normocapnic or normoxic and NAHCs (EtCO2: 55–70 mmHg with normal blood pH). Diaphragm fibers from euthanized rats were prepared for contractile function assessment and downstream signaling pathways analyses.

**Results:** Our results show that: (1) cross sectional area (CSA) was decreased by 40% following normocapnic CMV when compared with control sham operated (CTR) (*p* < 0.001), which was unaltered by NAHCs; (2) Specific force (SF: force normalized to CSA) was decreased to 58% of CTR following normocapnic CMV (*p* < 0.001). NAHCs increased SF by 15% compared with normocapnic rats (*p* < 0.05); (3) Muscle E3 ligases Murf1 and Atrogin-1 protein expression is unchanged following 5 days of CMV, irrespective of C02 conditions. LC3B, a marker for autophagy, with LC3B II:I ratio signifying activation. Following 5 days normocapnic CMV LC3B II:I ratio (*p* < 0.05) was reduced by 70% in diaphragm. NAHCs failed to alter the activation of LC3B or these muscle degradation pathways; (4) NAHCs however increased TNFα and IL-1β transcript expression in the diaphragm compared with normocapnic CMV and CTR groups; (5) As expected, NAHCs increased stability of respiratory peak pressure, peripheral oxygenation and perfusion, hemodynamic conditions.

**Conclusions:** Our results suggest that NAHCs have beneficial effects on single diaphragm fibers function irrespective of significant increase in muscle inflammation in non-septic animals. This, combined with the positive respiratory and hemodynamic effects, encourage further study of the therapeutic potential of CO_2_ in ICU.

## P170

### Mitigation of cellular apoptosis by diaphragm neurostimulation, and correlation between homovanillic acid and apoptotic markers in a moderate-ARDS preclinical model

#### T Bassi^1^, E Rohrs^2^, K Fernandez^2^, M Nicholas^2^, J Wittmann^2^, M Ornowska^2^, M Gani^1^, D Evans^1^, S Reynolds^2^

##### ^1^Lungpacer Medical Inc., Burnaby, Canada, ^2^Fraser Health Authority, New Westminster, Canada

*Critical Care* 2022, **26(Suppl 1):** P170

**Introduction:** Dopamine production and hippocampal apoptosis in ARDS subjects undergoing mechanical ventilation (MV) have not been investigated. We explored correlation between these variables, and whether a hybrid ventilatory therapy affects them.

**Methods:** Pigs undergoing MV (volume control, PEEP 5 cmH_2_O, tidal volume 8 ml/kg) with moderate ARDS (PaO_2_/FiO_2_ 100–200) achieved by injecting oleic acid via catheter into the pulmonary artery. Subjects were assigned to three groups (n = 6 per group): lung injury (LI) with MV only (LI-MV), LI with temporary transvenous diaphragmatic neurostimulation (TTDN) every other breath (LI-TTDN50% + MV), and LI with TTDN every breath (LI-TTDN100% + MV). TTDN was delivered to achieve 15–20% reduction in ventilator pressure–time product. The hippocampus was harvested from each subject at study end, and terminal deoxynucleotidyl transferase dUTP nick end labeling (TUNEL) assay was used to stain for cellular apoptosis. TUNEL-positive percentage was determined using machine-learning software. Serum concentration of homovanillic acid at study end was analyzed. Spearman test was run to investigate any association between TUNEL percentage and homovanillic acid serum concentration. P values < 0.05 are considered statistically significant.

**Results:** TUNEL-positive cell percentages were [median (IQR)]: 26 (18–27) for LI-MV, 8 (7–14) for LI-TTDN50% + MV, and 6 (4–9) for LI-TTDN100% + MV. Homovanillic acid serum concentrations (ng/ml) were: 8 (7–13) for LI-MV, 6 (3–7) for LI-TTDN50% + MV, and 3 (3–4) for LI-TTDN100% + MV. Spearman correlation test showed a positive, linear, and moderate correlation between TUNEL-positive cell percentage and homovanillic acid serum concentration, r = 0.75, *p* = 0.0003 (Fig. 1).

**Conclusions:** We found a moderate correlation between the TUNEL-positive cell percentages and dopamine production in our moderate-ARDS preclinical model, and that TTDN subjects showed lower cellular apoptosis and dopamine production compared to MV only with LI.

**Fig. 1 (abstract P170):**
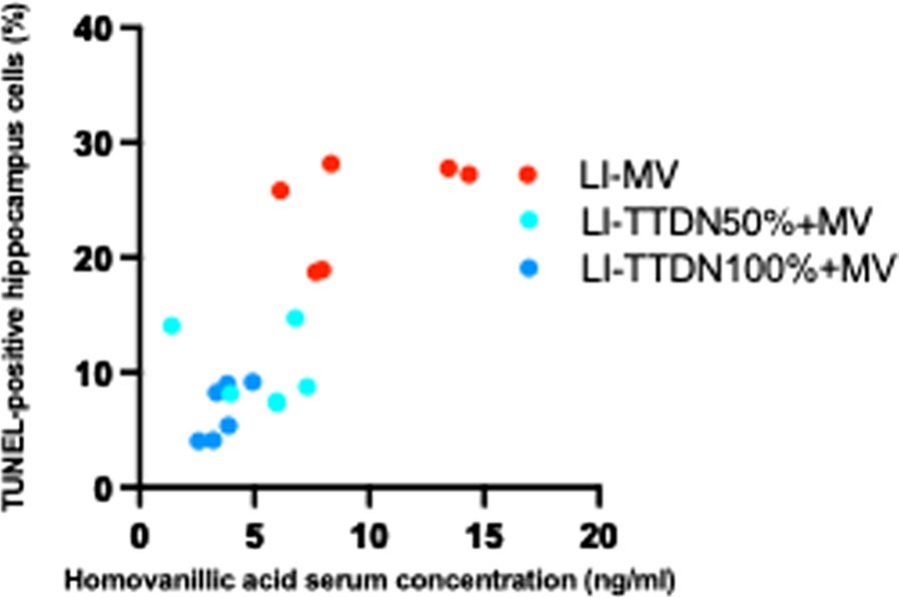
Correlation between TUNEL-positive hippocampus cells and homovanillic acid serum concentration

## P171

### Mitigation of neuroinflammation by diaphragm neurostimulation, and correlation between neuroinflammatory biomarkers in a moderate-ARDS preclinical model

#### T Bassi^1^, E Rohrs^2^, K Fernandez^2^, M Nicholas^2^, J Wittmann^2^, M Ornowska^3^, M Gani^1^, D Evans^1^, S Reynolds^2^

##### ^1^Lungpacer Medical Inc., Burnaby, Canada, ^2^Fraser Health Authority, New Westminster, Canada, ^3^Simon Fraser University, Burnaby, Canada

*Critical Care* 2022, **26(Suppl 1):** P171

**Introduction:** Correlation between microglia and astrocytes in ARDS subjects undergoing mechanical ventilation (MV) has not previously been demonstrated. We investigated correlation between these cells in subjects with various degrees of ventilation-induced neuroinflammation.

**Methods:** Juvenile pigs were assigned to four groups (n = 6 per group): lung injury (LI) with MV only (LI-MV), LI with MV plus temporary transvenous diaphragmatic neurostimulation (TTDN) every other breath (LI-TTDN50% + MV), LI with MV plus TTDN every breath (LI-TTDN100% + MV), and never ventilated (NV). Subjects undergoing MV (volume control, PEEP 5 cmH_2_O, tidal volume 8 ml/kg) had oleic acid injected into the pulmonary artery via Swan-Ganz catheter to achieve moderate ARDS (PaO_2_/FiO_2_ between 100 and 200). TTDN was delivered via a central line catheter embedded with electrodes, targeting a 15–20% reduction in ventilator pressure–time product. Hippocampus was harvested at study end, and ionizing binding adaptor molecule-1 (IBA-1) and glial fibrillary acid protein (GFAP) assays were used to stain microglia and astrocyte cells respectively. Positive-stained hippocampal cell percentages were determined using machine-learning software (ImageJ). Spearman test was run to investigate association between IBA-1 and GFAP hippocampal percentages. P-values < 0.05 are considered statistically significant.

**Results:** IBA-1- and GFAP-positive cell percentages found were [median (interquartile range)]: 18 (17–32) and 18 (14–24) for LI-MV, 12 (11–13) and 12 (9–15) for LI-TTDN50% + MV, 8 (7–10) and 9 (8–10) for LI-TTDN100% + MV, and 10 (8–10) and 10 (9–11) for NV. Spearman correlation test showed positive, linear, and moderate correlation between hippocampal IBA-1 and GFAP percentages, r = 0.57, *p* = 0.0031 (Fig. 1).

**Conclusions:** We found a moderate correlation between the percentages of hippocampal microglia and astrocytes after 12 h of MV with moderate ARDS, and that TTDN mitigated neuroinflammation in our injured-lung model.

**Fig. 1 (abstract P171):**
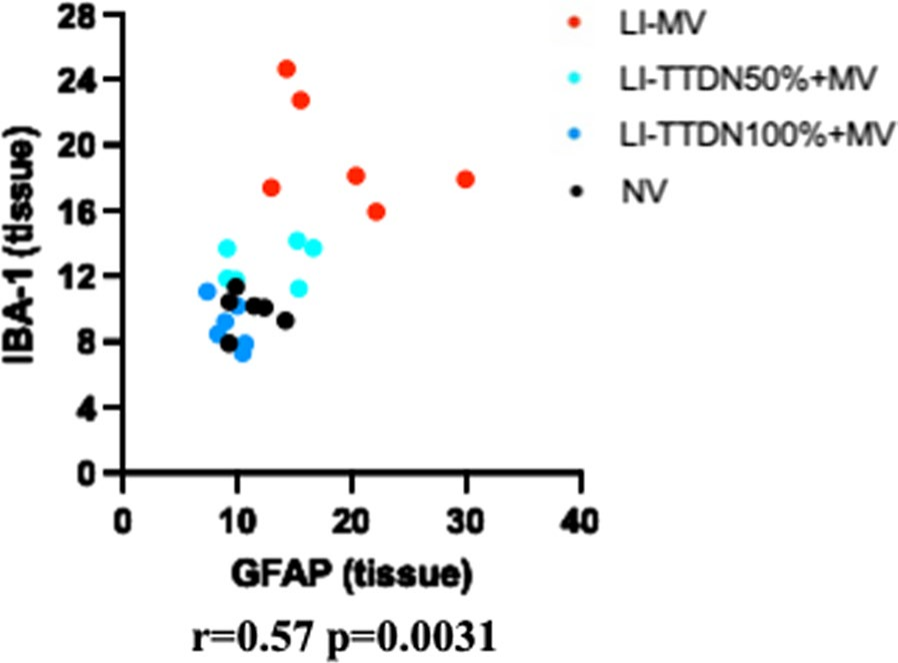
Correlation between GFAP-positive cells and IBA-1-positive cells

## P172

### Relationship among the electrical activity of the diaphragm, respiratory muscle ultrasound and indices of neuroventilatory coupling

#### M Umbrello^1^, E Tognacci^2^, E Antonucci^2^, A Arena^2^, S Cereghini^2^, S Muttini^2^

##### ^1^Ospedale San Carlo Borromeo, ASST Santi Paolo e Carlo—Polo Universitario, SC Anestesia e Rianimazione II, Milano, Italy, ^2^Ospedale San Carlo Borromeo, ASST Santi Paolo e Carlo—Polo Universitario, Milano, Italy

*Critical Care* 2022, **26(Suppl 1):** P172

**Introduction:** The electrical activity of the diaphragm (Edi), the main inspiratory muscle, reflects the neural control of respiration. There is limited knowledge of the correlation between Edi, the pressure generated during inspiration (ΔPes) and the ultrasonographic extent of its thickening (TFdi). The main aim of the current study was to assess the correlation between Edi, ΔPes and TFdi.

**Methods:** Patients > 18 years were enrolled if they were undergoing weaning in NAVA and with a catheter for ΔPes measurement. A stepwise NAVA level reduction was performed (-25%, -50%, -75% from baseline). During each step, an arterial sample was taken for gas analysis, neuroventilatory coupling parameters were recorded and respiratory muscle ultrasound was performed. Comparison of data was performed with one- or two-way ANOVA and linear mixed models were built to assess the relationship between variables. Two-tailed, *p* < 0.05 was considered statistically significant.

**Results:** We enrolled 16 patients (10 males, age 64 ± 17 years, BMI 24 ± 5 kg/m^2^), after an average of 8 ± 3 days from ICU admission. At enrolment, the PaO_2_/FIO_2_ ratio was 300 ± 91 mmHg, patients were ventilated with PEEP 7.7 ± 2.2 cmH_2_O and FIO_2_ 0.35 ± 0.1. Baseline NAVA level was 1.1 ± 0.7 cmH2O/uV, and Edi was 12.3 ± 8.5 uV. We found a significant correlation between Edi and ΔPes (R^2^ = 0.32, *p* < 0.001), while no significant correlation was found between Edi and TFdi (R^2^ = 0.01, *p* = 0.105). After analysis of individual patient data, we found two different behaviours of the Edi-TFdi relationship: a group in which the two variables were linearly correlated (preserved coupling), and one in which such coupling was not preserved (Fig. 1). A higher rapid shallow breathing index, a higher Edi, a lower respiratory system compliance, and a lower neuroventilatory and neuromechanical efficiency were found in patients with non-preserved coupling.

**Conclusions:** Edi is not always associated with TFdi; several physiological parameters were associated with the presence of a preserved or non-preserved coupling.

**Fig. 1 (abstract P172):**
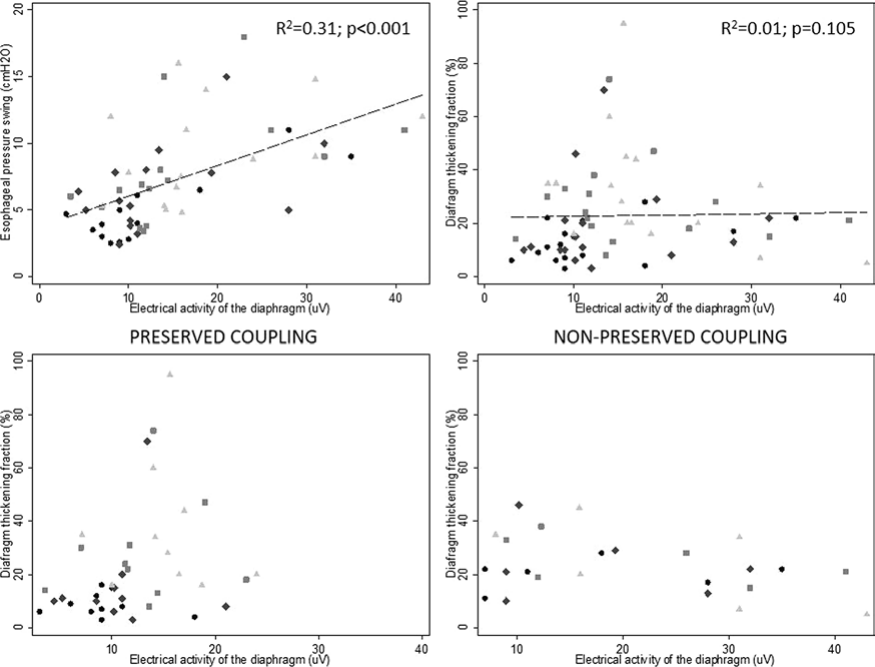
Correlation between the Edi and the esophageal pressure swing (upper left), the diaphragm thickening fraction (upper right) and relationship in patients with preserved (lower left) and non-preserved (lower right) coupling

## P173

### Diaphragmatic function and weaning failure from mechanical ventilation (MV): study using ultrasound (US) technique.

#### F Righetti^1^, E Colombaroli^2^

##### ^1^Intensive Care Unit, Emergency Department, Fracastoro Hospital, Emergency Department, San Bonifacio, Verona, Italy, ^2^Intensive Care Unit, Emergency Department, Fracastoro Hospital, San Bonifacio, Verona, Italy

*Critical Care* 2022, **26(Suppl 1):** P173

**Introduction:** The complex of dysfunctional effects induced by MV on the diaphragm is called Ventilator Induced Diaphragm Dysfunction(VIDD) [1]. The aim of this work is the study of the association between the diaphragmatic excursion(DE) measured with US and the weaning from MV.

**Methods:** 105 patients have been enrolled and they started pressure support ventilation(PSV) weaning trial. In all, DE was evaluated by viewing the right hemi-diaphragm using a 10 MHz linear probe in the right intercostal area. The US examination was performed at the same time as blood gas sampling and the measurement of ventilatory parameters. After 12 h of PSV, the patients started spontaneously breathe. If they were able to maintain spontaneous ventilation for the next 12 h, they were assigned to the successful weaning group (SW) otherwise to the failed weaning group (FW). The product between DE and respiratory rate, the DER, was calculated for each patient. Using the Mann–Whitney U test, the DER parameter was compared between SW and FW groups and in the subgroups of overweight/obese patients (*p* = 0.05).

**Results:** 57 patients are in SW group and 48 patients in FW group. There are not statistically differences in age, sex and weight class. The mean DER in SW group was 28.5 ± 27.7 cm; in FW group was 15.2 ± 8.6 cm; this difference is statistically significant (*p* = 0.028). Comparing the subpopulations of overweight and obese patients, the mean DER is 32.47 ± 3.2 cm in the SW, while in the FW is 13.9 ± 7.4 cm, with a statistically significant difference (*p* = 0.012).

**Conclusions:** Patients who were successfully weaned from MV had higher DER values during the weaning trial. These results allow us to hypothesize the use of DER as a predictive index of weaning from MV.

**Reference**

1. Peñuelas O et al. Intensive Care Med Exp 7:48, 2019

## P174

### Transvenous diaphragm neurostimulation lowers transpulmonary driving pressure in a preclinical ARDS model

#### E Rohrs^1^, TG Bassi^2^, M Nicholas^3^, J Wittmann^3^, M Ornowska^4^, K Fernandez^3^, M Gani^2^, S Reynolds^3^

##### ^1^Fraser Health Authority, Respiratory Therapy, New Westminster, Canada, ^2^Lungpacer Medical, Vancouver, Canada, ^3^Fraser Health Authority, New Westminster, Canada, ^4^Simon Fraser University, Burnaby, Canada

*Critical Care* 2022, **26(Suppl 1):** P174

**Introduction:** Acute respiratory distress syndrome (ARDS) drives morbidity and mortality, and exacerbates mechanical ventilator-induced lung injury (VILI). Mauri et al. recommend transpulmonary driving pressure be limited to < 10–12 cmH_2_O in patients with inhomogeneous lung parenchyma, to mitigate VILI [1]. Amato et al. reported that relative risk of death increases with every 7 cmH_2_O increase in driving pressure [2]. Current practice aims to reduce VILI in ARDS patients by limiting driving pressure. We previously showed that temporary transvenous diaphragm neurostimulation (TTDN) reduces driving pressure in normal lungs ventilated for 50 h; here we investigate whether TTDN reduces driving pressure in a moderate-ARDS preclinical model [3].

**Methods:** Moderate ARDS was induced in deeply sedated pigs, mechanically ventilated using volume-control mode at 8 ml/kg, PEEP 5 cmH_2_O, with respiratory rate and FiO_2_ set to achieve normal arterial blood gas values. ARDS was induced using oleic acid, delivered via the pulmonary artery until PaO_2_/FiO_2_ < 200. Animals were then ventilated for 12 h post-injury. MV + TTDN100% group (n = 6) received TTDN synchronized to inspiration on every breath, targeting a reduction in ventilator pressure–time-product of 15–20%; MV group (n = 6) received volume-control ventilation only.

**Results:** Transpulmonary driving pressure was lower both at post-injury and at study-end, and total study exposure to transpulmonary driving pressure was 36% lower in the MV + TTDN100% group than the MV group. TTDN on every breath limited transpulmonary driving pressure below 10 cmH_2_O post-injury and at study-end. Median transpulmonary driving pressure was 6.2 cmH_2_O greater in MV than MV + TTDN100% at study-end (Fig. 1).

**Conclusions:** TTDN reduces transpulmonary driving pressure by 36%, reducing the risk of VILI and the relative risk of death in a moderate-ARDS model.

**References**

1. Mauri et al. Intensive Care Med 42:1360–1373, 2016
2. Amato et al. N Engl J Med 372:747–755, 2015
3. Rohrs et al. J Appl Physiol 131:290–301, 2021

**Fig. 1 (abstract P174):**
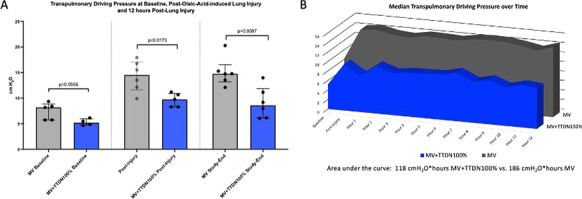
**A**: Median (IQR) driving pressure was lower in the MV+TTDN100% group post-injury and at study end. **B** Median transpulmonary driving pressure over time, showing 36% less driving pressure over the experiment for the MV+TTDN100% group.

## P175

### Transvenous diaphragm neurostimulation improves PaO_2_/FiO_2_ in a preclinical ARDS model

#### E Rohrs^1^, TG Bassi^2^, M Nicholas^3^, J Wittmann^3^, M Ornowska^4^, K Fernandez^3^, M Gani^2^, S Reynolds^3^

##### ^1^Fraser Health Authority, Respiratory Therapy, New Westminster, Canada, ^2^Lungpacer Medical, Vancouver, Canada, ^3^Fraser Health Authority, New Westminster, Canada, ^4^Simon Fraser University, Burnaby, Canada

*Critical Care* 2022, **26(Suppl 1):** P175

**Introduction:** PaO_2_/FiO_2_ is the main clinical tool used to categorize the level of acute lung injury in ventilated patients and has been shown to be well correlated with atelectasis in the context of the utilization of lung recruitment strategies [1]. It is also used to categorize the degree of severity of lung injury. We have previously shown that temporary transvenous diaphragm neurostimulation (TTDN) mitigates PaO_2_/FiO_2_ loss in pigs with healthy lungs ventilated for 50 h [2]. This study investigated the impact of TTDN on PaO_2_/FiO_2_ in a moderate-ARDS preclinical model, with mechanical ventilation (MV) for 12 h post-oleic-acid-induced lung injury.

**Methods:** MV was delivered to deeply sedated pigs using volume-control mode at 8 ml/kg, PEEP 5 cmH_2_O, with respiratory rate and FiO_2_ set to achieve normal arterial blood gas values. Moderate ARDS was induced using oleic acid, delivered via the pulmonary artery until PaO_2_/FiO_2_ < 200. Animals were then ventilated with the same strategy for 12 h post-injury. MV + TTDN100%-group (n = 6) received TTDN synchronized to inspiration on every breath, targeting a neurostimulation-induced reduction in ventilator pressure–time-product of 15–20%; MV-group (n = 6) received volume-control MV only.

**Results:** PaO_2_/FiO_2_ was not statistically different at baseline or post-injury and was higher at study-end in MV + TTDN100% (432 (383–490)) than MV (364 (327–376), *p* = 0.0360). PaO_2_/FiO_2_ recovery over time was better in MV + TTDN100%, and was affected by time (*p* < 0.0001), and interaction of TTDN and time (*p* = 0.0275) (Fig. 1).

**Conclusions:** PaO_2_/FiO_2_ recovery was faster with TTDN in a moderate-ARDS model, compared to lung-protective MV alone. We previously published that TTDN, using the same neurostimulation approach as this study, mitigated PaO_2_/FiO_2_ loss in healthy-lung pigs ventilated for 50 h. These findings indicate that TTDN results in better PaO_2_/FiO_2_ both in homogenous and heterogenous lungs.(5).

**References**

1. Nakahashi et al. J Crit Care 4:534.e1-534.e5, 2013
2. Rohrs et al. J Appl Physiol 131:290–301, 2021

**Fig. 1 (abstract P175):**
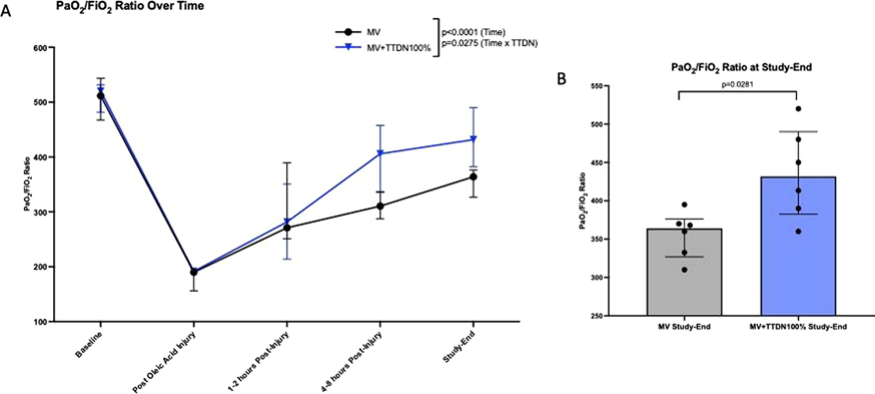
**A** Median (IQR) PaO_2_/FiO_2_ over time. **B** Median (IQR) PaO_2_/FiO_2_ at study-end.

## P176

### Neither R/I ratio nor recruitable volume differentiates recruiters from nonrecruiters by staircase recruitment maneuver

#### P Aramareerak, P Watcharasint

##### Phramongkutklao Hospital, Pulmonary and Critical Care Medicine, Bangkok, Thailand

*Critical Care* 2022, **26(Suppl 1):** P176

**Introduction:** A single-breath method allows bedside measurement of recruitment-to-inflation (R/I) ratio and recruited volume (Vrec). To date, there is a limit number of studies regards to these parameters compare between recruiters vs. nonrecruiters identified by staircase recruitment maneuver (SRM). Using SRM, we hypothesize whether recruiters would have a higher R/I ratio and higher Vrec than nonrecruiters.

**Methods:** We conducted a prospective experimental study in patient with acute respiratory distress syndrome (ARDS) who were admitted to a medical intensive care unit. All patients were measured for R/I ratio and Vrec. After that, SRM was performed to identify patients as recruiters (improvement of respiratory compliance (Crs) ≥ 30%) and nonrecruiters (improvement of Crs < 30%). Arterial blood gas parameters and respiratory mechanics were recorded at the baseline, end of SRM, 1 h, and 4 h following SRM. Primary outcome was difference in R/I ratio between recruiters vs. nonrecruiters. Secondary outcome was difference in Vrec between both groups.

**Results:** Twenty-two ARDS patients were enrolled. The most common cause of ARDS was pneumonia (n = 16). Median PaO_2_/FiO_2_ ratio at the baseline was 176 (interquartile range 93–230). Using improvement of Crs criteria following SRM, there were 17 nonrecruiters and 5 recruiters. No difference in R/I ratio was found between recruiters and nonrecruiters (0.33 vs. 0.23, *p* = 1.0) (Fig. 1 upper panel). Compared to nonrecruiters, there was a trend of higher Vrec in recruiters but not achieved statistically significant (330 ml vs. 380 ml, *p* = 0.31). While there was no different in baseline plateau and driving pressure, Recruiters had significantly lower delta plateau and driving pressure following SRM (Fig. 1 lower panel).

**Conclusions:** In patients with ARDS, using R/I ratio may not help differentiate recruiters and nonrecruiters identified by Crs improvement. However, there was a trend of higher Vrec in recruiters. These findings warrant further study.

**Fig. 1 (abstract P176):**
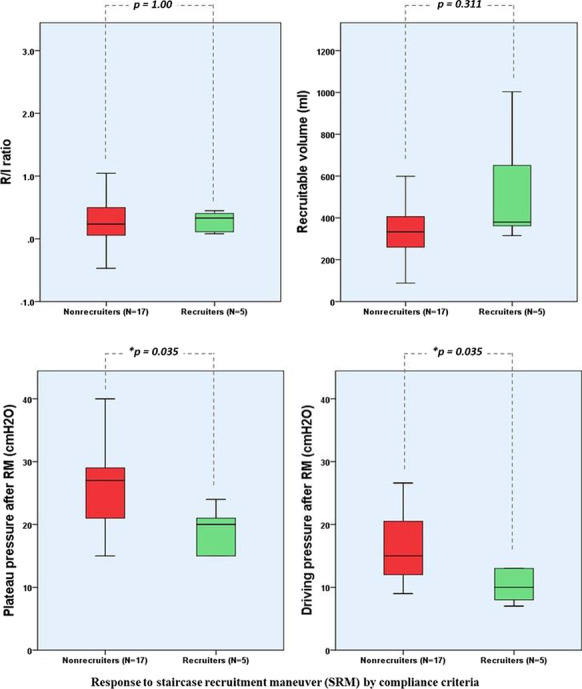
Response to staircase recruitment maneuver

## P177

### The use of VV-ECMO in patients with COVID 19 (is the first experience)

#### M.A. Petrushin^1^, E.V. Tereshchenko^2^, V.A. Alekseeva^2^, P.I. Melnychenko^1^, E.A. Kudryashova^1^, A.N. Grosheva^1^, M.A. Bachyrina^1^, M.A. Babaev^2^

##### ^1^Tver Regional Hospital, Intensive Care Unit, Tver, Russian Federation, ^2^Petrovsky National Research Centre of Surgery, Intensive Care Unit, Moscow, Russian Federation

*Critical Care* 2022, **26(Suppl 1):** P177

**Introduction:** Purpose of the study: to analyze the effectiveness of the use of intravenous ECMO as part of the complex therapy of patients with confirmed SARS-Cov-2 infection.

**Methods:** VV-ECMO was used in 19 patients (11 men, 8 women) over 18 years old (50 ± 12.5) with body mass index 32 ± 3.4 and severity of organ dysfunctions SOFA 3 points. Previously, all patients underwent therapy with interleukin-6 receptor inhibitors and glucocorticoids, non-invasive ventilation. The duration of mechanical ventilation before the start of ECMO was 1 ± 0.75 days, PaO_2_/FiO_2_ index—68 ± 12 mmHg, pHa were 7.25 ± 0.06, lactate 2.5 ± 0.6 mmol/l. ECMO was connected according to the femoral vena-jugular vein scheme, volumetric flow rate 3–4 l/min, oxygen 3 l/min.

**Results:** Disconnected from ECMO 37% (7), discharged 26% (5), 60—day survival—26%. Patients with an unfavorable outcome, compared with those who were discharged, had pronounced leukocytosis 14(10–17) versus 8 (4–8) × 10^9^/l, *p* = 0.009) and lymphocytopenia 7 ± 4% versus 12 ± 2%, *p* = 0.007). After initiation of the procedure, by day 3 in the lethal group: SOFA scores increased from 3 to 9 (in the group of survivors from 3 to 4 (*p* = 0.027)); the level of procalcitonin from 0.2 to 1.45 ng/ml; the number of platelets decreased by 48% (in the group of survivors it increased by 42% (*p* = 0.011); PaO_2_/FiO_2_ index did not change significantly (an increase of 200% in the group of survivors up to 206 mmHg (*p* = 0.011). The following complications developed in the group of patients with an adverse outcome: bleeding from cannulation sites (26.3%, n = 5), nosebleeds (26.3%, n = 5), circuit thrombosis (10.5%, n = 2) and pneumothorax (5.2%, n = 1).

**Conclusions:** VV-ECMO is an effective method of supporting lung function in patients with confirmed SARS-Cov-2 infection. The presence and progression of a bacterial infection is a predictor of an adverse outcome.

## P178

### Extracorporeal membrane oxygenation for critically ill patients with COVID-19 pneumonia: a retrospective cohort study

#### K Nijs, J Dubois, L Heremans, M Van Tornout, I De Pauw, B Stessel

##### Jessa Hospital, Department of Intensive Care and Anaesthesiology, Hasselt, Belgium

*Critical Care* 2022, **26(Suppl 1):** P178

**Introduction:** In patients with severe respiratory failure from COVID-19, extracorporeal membrane oxygenation (ECMO) can facilitate lung-protective ventilation and may improve outcome. The aim of this study is to investigate the clinical course, characteristics and outcomes of patients supported with ECMO for COVID-19 pneumonia.

**Methods:** All adult patients with a confirmed diagnosis of COVID-19 pneumonia admitted to the ICU of Jessa Hospital, Belgium and treated with ECMO between March 13, 2020, and June 30, 2021, were included. Data were prospectively entered into a database that included medical history, demographic data, laboratory results, ventilator settings, ventilator-derived parameters, therapeutic interventions and clinical outcomes. This database was retrospectively reviewed. The primary endpoint is ICU mortality. The study population is categorized based on primary endpoint. A Student t test or Mann Whitney U test and a Chi Square or Fisher's Exact tests were used to evaluated differences between survivors and non-survivors. A *p* < 0.05 is considered statistically significant.

**Results:** A total of 295 COVID-19 patients were admitted to the ICU, 24 needed ECMO and were analysed. Medical history, demographic data, laboratory results, ventilator settings, ventilator-derived parameters, therapeutic interventions and clinical outcomes, stratified for ICU mortality were analysed. ICU mortality was 45.8% (11/24). Only the following variables were significantly associated with ICU mortality: lower hospital length of stay (*p* = 0.01), need of continuous veno-venous hemofiltration (CVVH) during ECMO (*p* = 0.01), higher incidence of stroke (*p* = 0.04) and major bleeding (*p* = 0.004) (Table 1).

**Conclusions:** We were not able to identify baseline variables, treatment and/or ventilator strategies nor laboratory results that are associated with ICU mortality in this small cohort of COVID-19 patients supported with ECMO. Our results suggest that CVVH, stroke or major bleeding during EMCO treatment may increase the risk of ICU mortality.

**Table 1:** Clinical outcomes stratified for ICU mortality.

Outcomes	COVID-19 ECMO Survivors (n = 13)	COVID-19 ECMO Non-survivors (n = 11)	*p* value
Length of stay in ICU (days)	39.00 ± 28.10	26.36 ± 18.42	0.15
Length of stay in hospital (days)	49.33 ± 27.50	26.36 ± 18.42	0.01
CVVH during ECMO	1 (7.69%)	6 (54.55%)	0.01
Length of ECMO (days)	13.66 ± 10.73	16.64 ± 17.17	0.93
Stroke	0 (0.00%)	3 (27.3%)	0.04
Major bleeding	6 (46.15%)	11 (100%)	0.004
Heparin-induced thrombocytopenia	1 (7.70%)	1 (9.10%)	0.90

Data are expressed as mean ± standard deviation or as frequencies. A *p* value < 0.05 is considered statistically significant.

## P179

### Pneumomediastinum in ARDS (acute respiratory distress syndrome) caused by COVID-19 (coronavirus disease 2019): is protective lung ventilation really a weapon to our advantage?

#### F Righetti^1^, E Colombaroli^2^

##### ^1^Intensive Care Unit, Emergency Department, Fracastoro Hospital, Emergency Department, San Bonifacio, Verona, Italy, ^2^Intensive Care Unit, Emergency Department, Fracastoro Hospital, San Bonifacio, Verona, Italy

*Critical Care* 2022, **26(Suppl 1):** P179

**Introduction:** In mechanically ventilated patients suffering from ARDS as a consequence of COVID-19 interstitial pneumonia, we have often noted pneumomediastinum development despite the use of protective mechanical ventilation [1]. The purpose of this study is to determine whether the incidence of pneumomediastinum in patients with COVID-19 ARDS was higher than in ARDS patients without COVID-19 and whether this difference could be attributed to barotrauma or pulmonary fragility.

**Methods:** We divided the patients into two groups: Group A (patients with ARDS from COVID-19), Group B (patients with ARDS from other causes). All patients were admitted to ICU (intensive care unit) and treated with protective mechanical ventilation—tidal volume 4–6 ml/kg of IBW (ideal body weight), plateau pressure ≤ 28 cmH_2_O, driving pressure ≤ 12–14 cmH_2_O.

**Results:** In group A, pneumomediastinum occurred in 8 of 59 patients(13.5%) while in group B in 1 of 59 (1.6%) (*p* < 0.001). Mortality was 58% in group A patients while 48% in group B patients (*p* = 0.32). In group A the mean of tidal volume used was 5.6 ± 0.7 ml/kg of IBW, the mean of plateau pressure 22 ± 5 cmH_2_O and driving pressure 11 ± 4 cmH2O. In group B the mean of tidal volume used was 5.9 ± 0.5 ml/kg of IBW, the mean of plateau pressure 23 ± 4 cmH_2_O and driving pressure 10 ± 3 cmH_2_O.

**Conclusions:** The incidence of pneumomediastinum was approximately 8 times higher in the group of patients with COVID-19 ARDS despite the use of protective ventilation. This complication could be the consequence of greater lung fragility in patients with COVID-19 ARDS rather than in barotrauma which refers to elevated transpulmonary pressure.

**Reference.**

1. McGuinnes G et al. Radiology 297: E252-E262, 2020.

## P180

### Veno-venous extracorporeal membrane oxygenation (ECMO) in non-intubated patients with COVID-19 acute respiratory distress syndrome (ARDS): a non-inferiority study

#### R Attou, K Kaefer, A Gallerani, L Barreto Guttierez, P Honore, M Abou Iebdeh, E Waterplas, J Massaut, D Debels, C Pierrakos

##### CHU Brugmann, Intensive Care Unit, Laeken, Belgium

*Critical Care* 2022, **26(Suppl 1):** P180

**Introduction:** Invasive ventilation initiation after a prolonged period of non-invasive ventilation (NIV) trial can be associated with poor outcome in coronavirus disease 2019 (COVID-19) ARDS patients. This study aimed to document our center’s experience with COVID-19 ARDS patients treated with veno-venous ECMO (VV-ECMO) after a prolonged NIV trial period to avoid intubation. We speculated that VV-ECMO support is not associated with a worse outcome than invasive ventilation in these patients.

**Methods:** We retrospectively reviewed 6 patients with COVID-19 ARDS who presented severe hypoxemia and pneumomediastinum after NIV (ECMO group). Twenty patients with COVID − 19 and age less than 70 years old were treated in the first wave of the national outbreak and underwent NIV trials for more than 24 h before intubation (Control group). The primary outcome was intensive care unit (ICU) survival and secondary ECMO or mechanical ventilation weaning at 28 days.

**Results:** The age of the patients in the ECMO group was 59 years (IQR: 46 − 65) and SAPS II score 47 (IQR: 46 − 52), compared to 60 years (IQR: 51 − 66) (*p* = 0.71) and 48 (IQR: 45 − 54) (*p* = 0.63) in the control group. NIV duration before ECMO or invasive ventilation initiation was 5 days (IQR: 2 − 8) and 3 days (IQR: 1 − 5), respectively (*p* = 0.13). Drainage multistage femoral cannula 25 F and internal jugular infusion cannula 21 F were placed percutaneously. After cannulation, the patients received light sedation that permitted communication, active physiotherapy and oral feeding. None of the patients in the ECMO group died within 28 days after ECMO initiation (Fig. 1, Panel A) or received invasive ventilation. VV-ECMO was not associated with longer mechanical support than invasive ventilation (HR: 1.26 95%CI: 0.24 − 6.55, *p* = 0.77) (Fig. 1, Panel B).

**Conclusions:** VV-ECMO can be a not inferior strategy to invasive ventilation for treating patients with COVID-19 ARDS and severe hypoxemia not responding to long trials of NIV.

**Fig. 1 (abstract P180):**
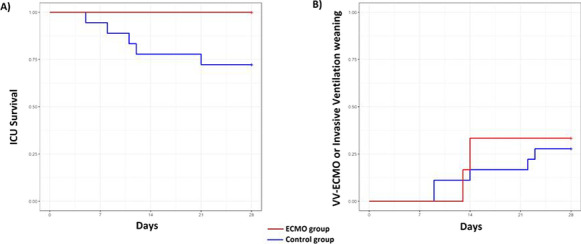
VV-ECMO (ECMO group) and Invasive ventilation strategy (Control group) and probability of survival (**A**) and successful liberation from ECMO or mechanical ventilation (**B**) at 28 days.

## P181

### Time dependent covariate analysis of liver parameters for VV-ECMO survival

#### SFE Ehrentraut, TS Stiehl, MS Schmandt, SM Münster, CP Putensen, JCS Schewe

##### University Hospital Bonn, Department of Anaesthesiology and Intensive Care Medicine, Bonn, Germany

*Critical Care* 2022, **26(Suppl 1):** P181

**Introduction:** Extracorporeal membrane oxygenation (ECMO) provides a rescue strategy in treatment of acute respiratory distress syndrome (ARDS). However, its use can be associated with serious complications and hepatic dysfunction is a commonly observed phenomenon. Due to several limitations of studies addressing liver dysfunction results are conflicting and to date there is no conclusive evidence of impact of altered liver dysfunction in prognostication of VV-ECMO support survival. This is further limited by a lack of data regarding sequential alterations of liver parameters during ECMO support [1]. We aimed for detection in trends of liver dysfunction as a prognostic value to predict survival of VV-ECMO support in a large cohort.

**Methods:** Retrospective, monocentric evaluation of VV-ECMO patients from the years 2013–2021. Liver dysfunction was assessed by sequential measurements of bilirubin and liver enzymes (ASAT, ALAT). Patients were evaluated in regard to mortality, disease severity (SOFA-Score on ICU admission, duration of ECMO support, ICU/hospital length of stay). The study has been approved by the local ethics committee (#492/20). All values are mean ± standard deviation(SD) or median ± interquartile range (IQR). Univariate time dependent Cox proportional hazard (CoxPH) analyses were performed using R.

**Results:** 366 (262 male) patients underwent VV-ECMO for ARDS. Grouped (by in-hospital survival status, survivors vs. non-survivors) stats: age 51 ± 12 vs. 56 ± 12 years (*p* < 0.001), weight 102 ± 33 vs. 94 ± 32 kgs (*p* = 0.02), height 175 ± 10 vs. 174 ± 9 cm. Median ECMO-support was 13 ± 9 vs. 16.7 ± 27 days (no differences between groups), SOFA Score on ICU admission was 8 ± 2. vs 9 ± 3 (*p* = 0.005). Hazard ratios for liver parameters are depicted in the Figure (Fig. 1).

**Conclusions:** Time dependent CoxPH analysis revealed bilirubin and ASAT as independently increase the risk of death on VV ECMO support.

**Reference**

1. Lazzeri C et al. J Artif Organs 21:61–67, 2018

**Fig. 1 (abstract P181):**
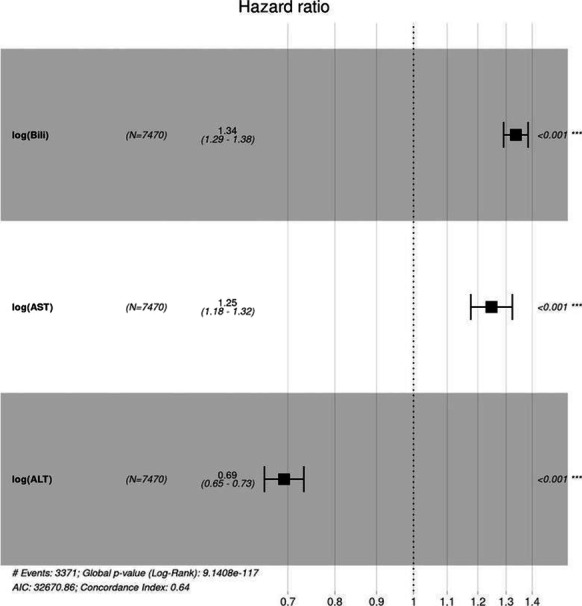
Hazard ratios of liver parameters. Bilirubin and ASAT independently increase risk of death on VV-ECMO

## P182

### The survival of patients treated with extracorporeal hemoadsorption and ECMO analysed in a nation wide registry.

#### AH Heidenreich^1^, KK Kaier^2^, CVZM Von zur Mühlen^1^, DS Dürschmied^3^, MZ Zehender^1^, CB Bode^1^, PS Stachon^3^, AS Supady^1^

##### ^1^University Heart Center Freiburg, Medical Faculty, University of Freiburg, Department of Cardiology and Angiology I, Freiburg, Germany, ^2^Faculty of Medicine and Medical Center, University of Freiburg, Institute of Medical Biometry and Statistics, Freiburg, Germany, ^3^University Medical Centre Mannheim, Medical Faculty Mannheim, Heidelberg University, Department of Cardiology, Angiology, Haemostaseology and Medical Intensive Care, Mannheim, Germany

*Critical Care* 2022, **26(Suppl 1):** P182

**Introduction:** Extracorporeal hemoadsorption has received increasing attention in recent years. In particular, therapy with the CytoSorb adsorber has been used more and more frequently. The frequency of use and outcomes of the application in combination with extracorporeal membrane oxygenation (ECMO) have not been assessed in larger cohorts to date. We therefore analyzed all patients treated with veno-venous (VV) ECMO either with or without CytoSorb in Germany from 2017 to 2019.

**Methods:** Using German national records from a nationwide claim data set collected by the Research Data Center of the Federal Bureau of Statistics, all ECMO treatments with and without CytoSorb in Germany were analysed. Between-group differences of patient characteristics and in-hospital outcomes were calculated using unpaired t-test and chi-squared test. To assess the impact of CytoSorb on in-hospital mortality, uni- and multivariable logistic regression analyzes were carried out.

**Results:** From 2017 to 2019, out of 7,699 ECMO treatments, 7,015 were done without CytoSorb and 684 ECMO runs were performed with CytoSorb. When combined with CytoSorb, ECMO was initiated later than in patients without CytoSorb (8.41 vs. 6.98 days after admission to hospital; *p* = 0.02). Mortality was significantly lower in patients supported with ECMO without CytoSorb as compared to patients supported with ECMO and CytoSorb (51.23% vs. 72.08%; *p* < 0.001). Bleeding events were significantly more frequent in patients with CytoSorb (82.89% vs. 61.34%; *p* < 0.001). In univariate and multivariate logistic regression analyses the additional use of CytoSorb in ECMO patients was associated with higher in-hospital mortality (univariate analysis: OR 2.46; 95% CI: 1.95 to 3.09; *p* < 0.001; multivariate analysis: OR 2.62; 95% CI: 2.09 to 3.29; *p* < 0.001).

**Conclusions:** In this retrospective analysis, ECMO therapy with CytoSorb was associated with higher in-hospital mortality and an increased rate of bleeding events compared to ECMO runs without CytoSorb.

## P183

### Relationship of lung oxygenation to timing of hemoadsorption therapy initiation in COVID-19 patients requiring extracorporeal mechanical oxygenation (ECMO): an observational analysis from the CytoSorb therapy in COVID-19 (CTC) Registry

#### J Hayanga^1^, T Song^2^, L Durham^3^, L Garrison^4^, P Nelson^5^, H Kroger^5^, Z Molnar^6^, E Deliargyris^5^, N Moazami^7^

##### ^1^West Virginia University School of Medicine, Morgantown, USA, ^2^University of Chicago Medicine, Chicago, USA, ^3^Medical College of Wisconsin, Milwaukee, USA, ^4^Franciscan Health Indianapolis, Indianapolis, USA, ^5^CytoSorbents Corporation, Princeton, USA, ^6^CytoSorbents Europe, Berlin, Germany, ^7^New York University School of Medicine, New York, USA

*Critical Care* 2022, **26(Suppl 1):** P183

**Introduction:** The multicenter CTC Registry study collected patient-level data in COVID-19 patients receiving CytoSorb therapy under FDA Emergency Use Authorization. An earlier report on the first 52 CTC patients on ECMO treated with CytoSorb showed 69% overall survival [1]. The current analysis focuses on changes in pulmonary function relative to the time of CytoSorb therapy.

**Methods:** A total of 56 patients from 5 U.S. centers were included. Data on demographics, mechanical ventilation (MV), ECMO, and arterial blood gases during CytoSorb therapy were analyzed. Linear regression was used to evaluate the relationship between the timing of initiation of CytoSorb therapy to lung oxygenation according to changes in PaO_2_/FiO_2_ ratio.

**Results:** In the current analysis, 71% (40/56) overall survival was observed. For these survivors, time to CytoSorb start after ICU admission, MV start, and ECMO start was 138 ± 171.3 h, 83 ± 111.0 h, and 55 ± 156.5 h, respectively, with mean duration of CytoSorb therapy of 83 ± 29.1 h. At the first 24 h following CytoSorb therapy, oxygenation was improved evidenced by decreased MV FiO_2_ and ECMO FdO_2_ requirements and an increased PaO_2_/FiO_2_ ratio (90.2 ± 58.13 mmHg to 166.3 ± 98.67 mmHg, *p* < 0.001, N = 21). Linear regression analysis suggested that earlier initiation of CytoSorb therapy following ICU admission may be correlated to greater improvements in PaO_2_/FiO_2_ ratio (r = -0.37, *p* = 0.103), however, this trend did not achieve statistical significance.

**Conclusions:** High survival rates have been observed with adjunct CytoSorb therapy in critically ill COVID-19 patients on ECMO. The current analysis suggests that early initiation of hemoadsorption following ICU admission may contribute to earlier improvements in native lung oxygenation.

**Reference**

1. Song T et al. Front Med (Lausanne) 8:773,461, 2021

## P184

### Excess oxygen administration and adverse outcomes in patients with SARS-CoV-2 pneumonia requiring invasive mechanical ventilation: a retrospective study

#### E Damiani^1^, R Giorgetti^2^, S Vannicola^1^, P Giaccaglia^1^, M Cerioni Canone^1^, ME Bagnarelli^1^, G Danieli^1^, S Marzialetti^1^, P Gregori^1^, A Donati^1^

##### ^1^Università Politecnica delle Marche, Biomedical Sciences and Public Health, Ancona, Italy, ^2^Università Politecnica delle Marche, Biomedical Sciences, Ancona, Italy

*Critical Care* 2022, **26(Suppl 1):** P184

**Introduction:** Even if life-saving in most cases, excess O_2_ may have adverse effects. We described the prevalence of hyperoxemia and excess O_2_ administration in patients with severe acute respiratory syndrome due to novel coronavirus (SARS-CoV-2) and explored the association with mortality in the intensive care unit (ICU) or ventilator-associated pneumonia (VAP).

**Methods:** Retrospective single-centre study on 134 patients with SARS-CoV-2 requiring mechanical ventilation for ≥ 48 h. We calculated the excess O_2_ administered based on an ideal arterial O_2_ tension (PaO_2_) target of 55–80 mmHg. We defined hyperoxemia as PaO_2_ > 100 mmHg and hyperoxia + hyperoxemia as an inspired O_2_ fraction (FiO_2_) > 60% + PaO_2_ > 100 mmHg. Risk factors for ICU-mortality and VAP were assessed with multivariate analyses.

**Results:** Each patient received an excess O_2_ of 1121 [829–1449] l per day of mechanical ventilation. Hyperoxemia was found in 38 [27–55] % of arterial blood gases, hyperoxia + hyperoxemia in 11 [5–18] %. The FiO_2_ was not reduced in 69 [62–76] % of cases of hyperoxemia. Adjustments were more frequent with higher PaO_2_ or initial FiO_2_ levels. ICU-mortality was 32%. VAP was diagnosed in 48.5% of patients. Hyperoxemia (odds ratio [OR] 1.300 95% confidence interval [1.097–1.542]) and hyperoxia + hyperoxemia (OR 1.144 [1.008–1.298]) were associated with higher risk for ICU-mortality, independently of age, Sequential Organ failure Assessment score at ICU-admission and mean PaO_2_/FiO_2_. Hyperoxemia (OR 1.033 [1.006–1.061]), hyperoxia + hyperoxemia (OR 1.038 [1.003–1.075]) and daily excess O_2_ (OR 1.001 [1.000–1.001]) were identified as risk factors for VAP, independently of body mass index, blood transfusions, days of neuromuscular blocking agents before VAP, prolonged prone positioning and mean PaO_2_/FiO_2_ before VAP.

**Conclusions:** Excess O_2_ administration and hyperoxemia were common in mechanically ventilated patients with SARS-CoV-2 and may be associated with ICU-mortality and greater risk for VAP.

## P185

### The use of mechanical power and driving pressure during mechanical ventilation for ARDS in an ICU in a lower income country (Sri Lanka)

#### K Indraratna, H Yapa, D Jayasekera

##### Sri Jayewardenepura General Hospital, Anaesthesia and Intensive Care, Nugegoda, Sri Lanka

*Critical Care* 2022, **26(Suppl 1):** P185

**Introduction:** To determine the use of mechanical power [1]and driving pressure [2] during ventilation for ARDS in a lower income country. To assess whether these measurements affect outcomes in patients with ARDS being treated in a lower middle income country.

**Methods:** Patients who fulfilled the Berlin criteria for ARDS [3] were recruited. The arterial blood gas immediately prior to ventilation, the 1st post ventilation blood gas, the ventilator settings on which the patient was stabilized on the first day were considered. The 1st post ventilation blood gas should have fulfilled the Berlin criteria as well to remain in the study. Patients with ejection fractions less than 30, and GCS of 3 prior to ventilation were excluded. Mechanical power and driving pressure were calculated from the first day’s finalized ventilator settings. The ventilator settings, blood gas reports and chest X-rays were collected by an observer who was not privy to the measurements being made. The staff in the ICU were not aware of the measurements being taken.

**Results:**

- There were 20 patients. Mortality was 60%.
- 45% had mechanical power of over 12. Mortality 88.9%.
- 55% had mechanical power less than 12. Mortality 45%.
- 50% had driving pressure of over 14. Mortality 100%.
- 50% had driving pressure of less than 14. Mortality 20%.
- 33% had driving pressure over 14 and mechanical power over 12. Mortality 83%.
- 20% had driving pressure less than 14 and mechanical power less than 12. Mortality 0%.
- Documentation of values of mechanical power and driving pressure-0%.

**Conclusions:** The mortality for ARDS was high. Due attention has not been paid to these parameters. Mortality was high when the values were high. Driving pressure correlates better with mortality rates than mechanical power. When both mechanical power and driving pressure were normal mortality was 0.

**References**

1. Coppola S et al. Crit Care 24:246, 2020
2. Amato MBP et al. N Engl J Med 372:747–755, 2015
3. Ranieri VM et al. JAMA 3,072,526–2533, 2012

## P186

### Utility of dead-space-to-tidal-volume ratio in predicting extubation failure: a systematic review and meta-analysis

#### M Van Haute, KC Jimenez, S Kumar, Z Libozada, PD Lim, MB Llamzon, PK Llantada, AC Lopez, MA Loyola

##### San Beda University, College of Medicine, Metro Manila, Philippines

*Critical Care* 2022, **26(Suppl 1):** P186

**Introduction:** Despite many advances made with weaning protocols, the need for reintubation still arises in 10%-20% of patients extubated after successful weaning [1]. Such extubation failure (EF) is associated with prolonged mechanical ventilation and mortality [2]. Dead-space-to-tidal-volume ratio (Vd/Vt) as an indicator of extubation outcome was investigated in several studies, however, the results were inconsistent. We hypothesize that higher Vd/Vt values are associated with EF. Thus, we aimed to assess the utility of Vd/Vt in predicting EF among mechanically ventilated ICU patients via a systematic review and meta-analysis of observational studies.

**Methods:** A systematic search (PubMed, Scopus and ScienceDirect; in March 2021) was performed for published papers that investigated the relationship between Vd/Vt and outcome of extubation following successful spontaneous breathing trial. Pooled Vd/Vt mean difference (MD) between extubation outcomes, and pooled Vd/Vt predictive accuracy measures (sensitivity [Sn], specificity [Sp], diagnostic odds ratio [dOR] and summary area under the curve [AUC]) were determined using a random-effects model. Subgroup analysis based on age (pediatric vs adult) was likewise done.

**Results:** Eight studies (684 patients) met the inclusion criteria. Optimal Vd/Vt cutoffs ranged from 0.50 to 0.65. EF patients had higher mean Vd/Vt relative to extubation successes (MD = 0.12; 95% CI 0.06–0.18; *p* < 0.01). Pooled Sn, Sp and dOR were 0.79 (95% CI 0.66–0.88), 0.74 (95% CI 0.67–0.81) and 9.72 (95% CI 3.89–24.32), respectively. The summary AUC (Fig. 1) is 0.83 (95% CI 0.79–0.86). No significant differences in pooled estimates between the age subgroups were noted.

**Conclusions:** Vd/Vt is a potential predictor for EF given its modest Sn and Sp, high dOR and moderate-to-high summary AUC. However, further studies are needed to determine a single optimal Vd/Vt cutoff, above which EF is expected.

**References**

1. Thille AW et al. Am J Respir Crit Care Med 187:1294–302, 2013
2. Epstein SK et al. Chest 112:186–192, 1997

**Fig. 1 (abstract P186):**
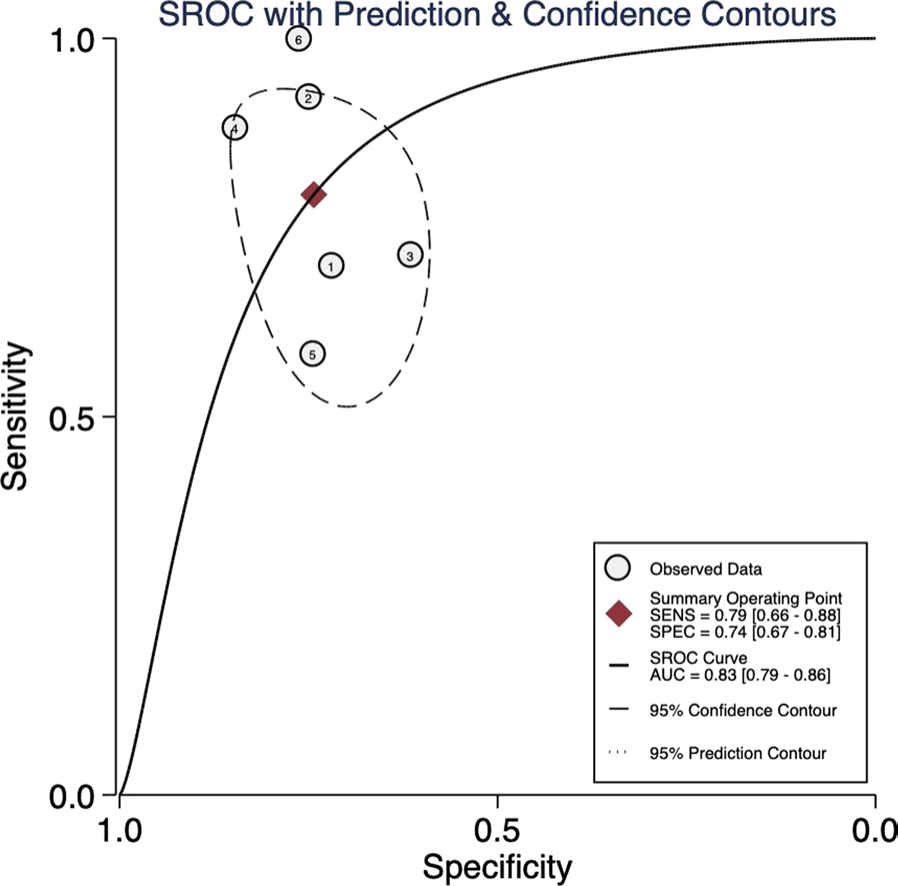
Summary area under the curve with prediction and confidence contours

## P187

### Effects of anterior external chest wall compression on respiratory mechanics and gas exchange in COVID-19 ARDS patients

#### B De Dreuker^1^, AH Jonkman^2^, LMA Heunks^2^

##### ^1^UZA, ICU, Edegem, Belgium, ^2^AMC VUmc, ICU, Amsterdam, Netherlands

*Critical Care* 2022, **26(Suppl 1):** P187

**Introduction:** Beneficial effects of prone position on outcome in ARDS may be related to decreased anterior chest wall compliance, which facilitates lung recruitment. The effects of anterior external chest wall compression (AEC) in supine position on respiratory mechanics and gas exchange are less well known [1]. We aimed to evaluate the effects of AEC in invasively ventilated COVID-19 patients.

**Methods:** In 10 sedated and paralyzed COVID-19 patients ventilated in volume control mode, airway and esophageal pressures, driving pressure (DP) and lung compliance (Clung) were recorded before (baseline) and during (5 kg, 10 kg) AEC (10-min epochs). AEC was performed by placing one or two 5 l fluid bags on anterior chest. Repeated arterial blood gas analysis was available in 6 patients.

**Results:** Patients’ (9/1 M/F, age 65 [range 53–74] yrs, BMI 29.2 [range 23.2–50.5] kg/m^2^, PEEP median 11.8 (IQR 9.7–13.9) cmH_2_O), Clung at baseline (median 21.3; IQR 15.0–32.6 ml/cmH_2_O) increased > 10% with 5 kg AEC (mean 20.7 ± 7.3%, max. increase 30.9%) and additionally increased with 5.9% (range -2.9–19.3%) with 10 kg AEC (Fig. 1a, *p* < 0.001). Better response was related to an anteriorly located baby lung on CT imaging. At baseline, median transpulmonary DP (Pl DP) was 17.46 (IQR 11.50–25.07) cmH_2_O and decreased with 16.2 ± 4.9% and 20.6 ± 8.2% during 5 kg and 10 kg AEC, respectively (Fig. 1b, p < 0.001). pCO_2_ decreased in 4 patients and remained equal in 2 patients (Fig. 1c). The latter group also had minimal change in Pl DP and Clung. In all patients PO_2_ decreased with need for increasing FiO_2_. Median P/F ratio of 124 (IQR 104–143) mmHg at baseline decreased to 105 (IQR 92–124) mmHg and 86 (IQR 78–114) mmHg during 5 kg and 10 kg AEC respectively.

**Conclusions:** This preliminary data demonstrates improved compliance of the aeriated lung by decreasing hyperinflation. No evidence for recruitment was found in contrast to existing literature showing improved oxygenation [2].

**References**

1. Marini JJ et al. Crit Care 25:264, 2021
2. Carteaux G et al. Crit Care. 25:187, 2021

**Fig. 1 (abstract P187):**
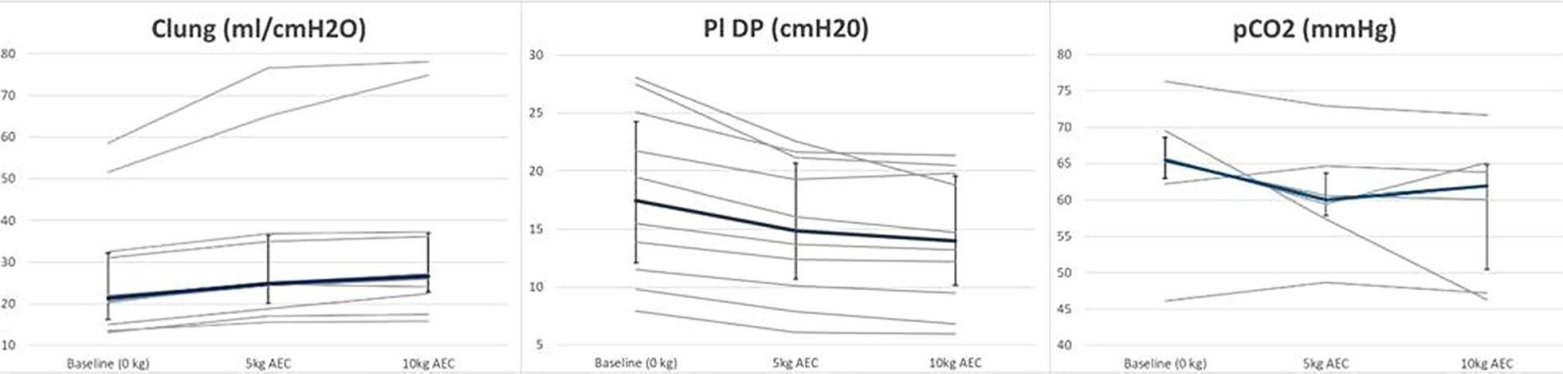
**a** Clung during baseline, 5 kg AEC and 10 kg AEC (n = 10), **b** Pl DP (n = 10), **c** PCO_2_ (n = 6). Gray: individual patients. Blue line: median values + IQR.

## P188

### Association of sedation level and 28-day mortality: a COVID-19 cohort multicentre study sub analysis

#### BM Monleon^1^, SM Martinez^2^, JAC Carbonell^2^, RF Florea^2^, MGP Garcia Perez^2^, RB Badenes^2^

##### ^1^Hospital Clinico Universitario, Anesthesiology, Intensive Care and Pain Management, Valencia, Spain, ^2^Hospital Clinico Universitario, Valencia, Spain

*Critical Care* 2022, **26(Suppl 1):** P188

**Introduction:** COVID-19 started in Wuhan (China) in December 2019 [1]. World pandemic was declared by the WHO in March 2020 [2]. Since then, millions of patients have been infected worldwide. Our group published in March 2021 a multicentre study analysing the prevalence and risk factors for delirium in critically ill patients with COVID-19 infection [3]. In this sub analysis of the main study, the primary outcome was the association of sedation level since ICU admission with 28-day mortality in patients admitted to ICU due to COVID-19.

**Methods:** The exposure tested against mortality was excessive sedation (in the coma range) defined as ‘Did patients have a documented sedation score in the coma range at any point this day? RASS =  − 4 or − 5; SAS = 2 or 1; MAAS = 0 or 1; Ramsay 5 or 6″. Data from 2017 patients was available for analysis, collected after patients’ admission to ICU. Other covariates analyzed were baseline patient characteristics, medical history and treatment applied in the ICU. Logistic regression was used in all analyses and results presented as odd ratios (OR) with 95% confidence intervals.

**Results:** Deep sedation (RASS =  − 4 or − 5, SAS = 2 or 1, MAAS = 0 or 1, Ramsay 5 or 6) was positively and significantly associated to mortality within 28 days since ICU admission. P value was 0.012 and the OR was 2.00 with a 95% confidence interval of 1.16–3.45.

**Conclusions:** As shown by this sub analysis, deep sedation increases mortality rates in critically ill COVID 19 patients. We should try to decrease sedation levels to avoid RASS of − 4 and − 5 to favor patients’ outcomes admitted to the ICU.

**References**

1. Zhu N et al. N Engl J Med 382**:**727–33, 2020.
2. https://www.who.int/emergencies/diseases/novel- coronavirus-2019/situation-reports (accessed May 25, 2020).
3. Badenes R et al. Lancet Respir Med 9:239–250, 2021.

## P189

### Sedation requirements in patients receiving venovenous extracorporeal membrane oxygenation in the era of COVID-19

#### LE Pratt, KA Considine, TS Lam, JT Jancik

##### Hennepin Healthcare, Department of Clinical Pharmacy, Minneapolis, United States

*Critical Care* 2022, **26(Suppl 1):** P189

**Introduction:** Current literature regarding sedative dosing in venovenous extracorporeal membrane oxygenation (VV-ECMO) is sparse, with information pertaining to those with SARS-CoV-2 (COVID) requiring VV-ECMO being even rarer. The purpose of this quality improvement project was to examine the sedation practices and requirements of patients who received VV-ECMO in the year prior to and the first year of the COVID epidemic.

**Methods:** This was a single-center, observational study. Adults who received VV-ECMO from January 1, 2019 through December 31, 2020 were included. Those who received VV-ECMO in 2019 were considered the pre-COVID cohort, while those who received VV-ECMO during 2020 were the COVID cohort. Demographics, clinical and lab parameters, and doses of oral and intravenous (IV) opioids, benzodiazepines (BZD), and other sedatives were recorded.

**Results:** Twelve pre-COVID and 13 COVID patients were included with a mean duration of VV-ECMO of 10 and 27 days, respectively. Mean medication doses during the first week of VV-ECMO and mean duration of use are described in Table 1. Patients who received IV opioids (11 vs. 13), IV BZD (12 vs. 13), and propofol (10 each) were similar between groups. Median number of IV sedation agents used was similar between groups (3.5 vs. 4), while the number of oral agents (opioids, BZD, phenobarbital) was higher in the COVID group (0 vs. 2).

**Conclusions:** There were a similar number of IV agents used between the two cohorts. Oral medications were used more often to augment sedation in the COVID cohort of this study.

**Table 1 (abstract P189):** Mean medication doses during the first week of VV-ECMO and mean duration of use. *Doses are in mcg fentanyl, mg midazolam, or mg propofol

		**Opioids**	**BZD**	**Propofol**
Mean Dose Days 1–7*	Pre-COVID	3935	275	4419
	COVID	4750	292	6396
Mean Days, (% ECMO time)	Pre-COVID	10 (100)	9 (96)	6 (73)
	COVID	25 (95)	20 (76)	21 (81)

## P190

### Effect of ramelteon on reducing in-hospital mortality in critically ill adults: a nationwide observational cohort study in Japan

#### T Takeuchi^1^, N Inoue^1^, T Masuda^2^, K Fushimi^1^

##### ^1^Tokyo Medical and Dental University, Department of Health Policy and Informatics, Tokyo, Japan, ^2^Tokyo Medical and Dental University, Department of Intensive Care Medicine, Tokyo, Japan

*Critical Care* 2022, **26(Suppl 1):** P190

**Introduction:** Delirium is a common complication in intensive care units (ICU) and is considered to be an independent risk factor for mortality [1]. Some studies have attempted to show preventative effects of pharmacological treatments including ramelteon, melatonin receptor agonists, but none have been proven to improve major outcomes [2]. Recent small studies reported that ramelteon may prevent critically ill patients from developing delirium [3], but the data are limited and the effect of ramelteon on mortality is still unknown. Our aim is to determine whether ramelteon reduces mortality in critically ill adults.

**Methods:** We conducted a cohort study using the Diagnosis Procedure Combination database in Japan. We enrolled adult patients admitted to the ICU from April 2018 to March 2021 in Japan. We excluded patients who were admitted to the ICU after 48 h of hospital admission and were discharged or died within 2 days of ICU admission. After multiple imputation (MI) for missing values, propensity score (PS)-based fine stratification weighting (FSW) analysis was performed to compare in-hospital mortality between patients who were administered ramelteon within 48 h of admission (ramelteon group) and those who did not (control group).

**Results:** Of 39,869 ICU patients, 1,755 were in the ramelteon group and 38,144 were in the control group. After MI, PS-based FSW improved the balance of background factors between the two groups. Administration of ramelteon was associated with decreased days of haloperidol use (-0.101 days; 95% CI, − 0.190 to − 0.012) and reducing in-hospital mortality (10.2% vs 12.0%; odds ratio 0.832; 95% CI, 0.695 to 0.996).

**Conclusions:** For critically ill adults, treatment with ramelteon within 48 h of admission was associated with reducing days of haloperidol use and in-hospital mortality.

**References**

1. Balas MC et al. Chest 135:18–25, 2009
2. JW Devlin et al. Crit Care Med 46:e825-e873, 2018
3. Nishikimi M et al. Crit Care Med 46:1099–1105, 2018

## P191

### Dexmedetomidine increases central venous oxygen saturation in patients undergoing neurosurgery

#### MC Niño^1^, D Cohen^1^, AL Delgadillo^2^, EE Rodríguez^3^, SS Valencia^2^, GA Madrid^2^

##### ^1^Fundación Santa Fe de Bogotá, Neuroanesthesia Service, Bogotá, Colombia, ^2^Fundación Santa Fe de Bogotá, Anesthesiology Department, Bogotá, Colombia, ^3^Fundación Santa Fe de Bogotá, Intensive Care Department, Bogotá, Colombia

*Critical Care* 2022, **26(Suppl 1):** P191

**Introduction:** Dexmedetomidine decreases sympathetic tone and reduces cerebral metabolic rate [1,2], important benefits in neurosurgery. We aimed to evaluate the effect of this medication on systemic metabolic markers that reflect the oxygen supply and demand relationship. Our hypothesis was that dexmedetomidine will diminish oxygen consumption in patients under general anesthesia and we would see an increase in central venous oxygen saturation (ScvO_2_).

**Methods:** A quasi-experimental design, including patients at least18 years old programmed for craniotomy between April 2019 and March 2020 was conducted. We excluded patients with hemodynamic instability, ventilator support, hemodynamic support, anemia, oxygen requirement, sepsis, systemic inflammatory response, and pregnancy. A sample size of 66 was calculated. After induction, a central venous catheter was placed, a sample of blood was obtained (T0), and dexmedetomidine infusion (0.3 μg/kg/h) was started. A second sample was obtained 60 min later (T1) before incision was made. ScvO_2_ and lactate were measured in both samples. Global and stratified statistical analysis for paired samples were carried out.

**Results:** A sample of 75 was analyzed. Overall, dexmedetomidine increased ScvO_2_ (81.2% in T0 to 82.9% in T1; p 0.05). When stratified analysis was conducted, the increase in ScvO_2_ was significant in patients older than 65 years (*p* = 0.02) and patients with tumor (vs vascular lesion, *p* = 0.03). Lactate levels were constant across time.

**Conclusions:** Dexmedetomidine increases ScvO_2_ under general anesthesia without modifying lactate levels, especially among patients older than 65 years. Even though the difference was small, further studies are needed to elucidate clinical benefits of dexmedetomidine in neurosurgery.

**References**

1. Ebert TJ et al. Anesthesiology 93:382–94, 2000.
2. Drummond JC et al. Anesthesiology 108:225–32, 2008.

## P192

### Effect of sevoflurane on the activation of human neutrophiles in *ex vivo* models

#### D Starostin

##### V.A. Negovsky Research Institute of General Reanimatology of the Federal Research and Clinical Center of Intensive Care Medicine and Rehabilitology, Intensive Care & Emergency Medicine, Moscow, Russian Federation

*Critical Care* 2022, **26(Suppl 1):** P192

**Introduction:** Objective is to study the effect of different concentrations of sevoflurane on the activation of human neutrophils in an *ex* *vivo* model.

**Methods:** *T*he study was carried out on a cell culture of venous blood neutrophils of 5 healthy men. LPS and chemotaxis peptide fMLP as stimulants, was assessed by the expression level of CD11b and CD66b, IL-1b, IL-6 and IL-8, the level of phosphorylation of glycogen synthase GSK-3β. Annexin V and propidium iodide were used to assess apoptosis.

**Results:** Incubation of neutrophils with LPS and fMLP statistically significantly increased the expression of these molecules, namely, when LPS was treated at a dose of 200 ng/ml, the expression of CD11b and CD66b increased 2.3 and 2.2 times (*p* = 0.002 and *p* = 0.001, respectively), and upon treatment with fMLP at a dose of 100 nm., the expression of CD11b and CD66b increased by 1.7 and 2.0 times (*p* = 0.025 and *p* = 0.03, respectively). Upon incubation of neutrophils with the same LPS concentration after exposure to sevoflurane at a dose of 1.5 MAC, the expression level of CD11b and CD66b increased compared to intact neutrophils. The change in CD11b expression in this experiment was statistically insignificant (*p* = 0.055), the change in CD66b expression was statistically significant (*p* = 0.007). Thus, exposure to sevoflurane at a dose of 1.5 MAC decreases the pro-inflammatory activation of neutrophils by LPS. Stimulation of neutrophils by LPS was accompanied by dephosphorylation of GSK-3β, and exposure to 1.5 MAC of sevoflurane resulted in its phosphorylation. Thus, phosphorylation of GSK-3β in neutrophils by sevoflurane reduces the expression of CD11b and CD66b.

**Conclusions:** *S*evoflurane has a pronounced anti-inflammatory effect due to the suppression of neutrophil hyperactivation; one of the possible mechanisms of the effect of sevoflurane on the function of neutrophils is the phosphorylation of GSK-3β.

## P193

### Intravenous lidocaine during craniotomy does not blunt inflammatory response to surgery

#### MC Niño^1^, D Cohen^1^, P Aguilar^2^, EE Rodríguez^3^, GA Madrid^2^

##### ^1^Fundación Santa Fe de Bogotá, Neuroanesthesia Service, Bogotá, Colombia, ^2^Fundación Santa Fe de Bogotá, Anesthesiology Department, Bogotá, Colombia, ^3^Fundación Santa Fe de Bogotá, Intensive Care Department, Bogotá, Colombia

*Critical Care* 2022, **26(Suppl 1):** P193

**Introduction:**

Intravenous lidocaine has demonstrated to attenuate the proinflammatory effects associated with surgery, in addition to its analgesic properties [1]. Effective modulation of the inflammatory response may improve outcomes for patients undergoing craniotomy. We hypothesized that intravenous lidocaine would attenuate the inflammatory response to craniotomy, measured using widely available biomarkers (neutrophil to lymphocyte ratio [NLR] and platelet to lymphocyte ratio [PLR]).

**Methods:** A retrospective cohort study was conducted included patients at least 18 years old undergoing craniotomy under general anesthesia form January 2015 to December 2019. Exclusion criteria were simultaneous procedures, fever, sepsis, and intraoperative vasopressors. Anesthesia technique was standardized. Lidocaine use was at the discretion of the anesthesiologist. NLR and PLR were measured before and 24 h after surgery. The primary outcome was a reduction in biomarkers in the patients exposed to lidocaine. Secondary outcomes were ICU stay, postoperative septic shock, and mortality 20 days after surgery.

**Results:** A sample of 165 patients was analyzed, and 84 (50.9%) of them were exposed to lidocaine. Preoperative and 24-h postoperative values of NLR and PLR were similar between groups of patients. There were no differences in ICU length of stay, opioid consumption, incidence of septic shock, and mortality. Patients who received intraoperative lidocaine had less opioid consumption than those who were not exposed (*p* < 0.01).

**Conclusions:** Intravenous lidocaine during craniotomy did not blunt the inflammatory response associated with this procedure. However, less opioid consumption was associated with intraoperative infusion of this local anesthetic.

**Reference**

1. Dunn LK et al. Anesthesiology 126:729–737, 2017.

## P194

### Dose comparisons of fentanyl vs. morphine when used as infusion for analgosedation in mechanically ventilated adult intensive care unit patients.

#### A Casamento^1^, A Ghosh^2^, A Serpa Neto^1^, R Bellomo^1^

##### ^1^Austin Hospital, ICU, Heidelberg, Australia, ^2^The Northern Hospital, ICU, Epping, Australia

*Critical Care* 2022, **26(Suppl 1):** P194

**Introduction:** We aimed to study the dose equivalency of fentanyl and morphine when used for analgosedation in adult mechanically ventilated ICU patients.

**Methods:** This is a post hoc analysis of a previously published prospective cluster-crossover trial of fentanyl vs. morphine infusion for analgosedation in two university-affiliated hospital ICUs in Melbourne, Australia [1]. Patient height and weight were collected as part of routine nursing observations. Body mass index (BMI), body surface area (BSA), ideal body weight (IBW) and lean body weight (LBW) were calculated using these observations by validated formulae. In one center, hourly data on narcotic dose were extracted from electronic information. In the other center, narcotic dose data were manually extracted from observation charts at 4-h intervals and missing data was linearly imputed.

**Results:** In the initial trial, the median (IQR) ages were 59 (44–68; n = 344; male = 63%) in the fentanyl group and 59 (45–72; n = 337; male = 62%) in the morphine group. The median (IQR) weights (kg) were 84 (70–100; n = 317) in the fentanyl group and 82 (70–95; n = 309) in the morphine group. There were 261 patients in the fentanyl group and 273 patients in the morphine group in which the height and weight were both recorded. Of these, the median (IQR) BMI (kg/m^2^) was 30 (25–34) and 29 (25–33), the median (IQR) BSA (m^2^) was 2.0 (1.8–2.2) and 2.0 (1.8–2.1), the median IBW (kg) was 63 and 65, and the median LBW (kg) was 57 and 58 for fentanyl and morphine respectively. Table 1 provides the doses and ratios of fentanyl to morphine. The ratios of fentanyl to morphine ranged from 1:48 to 1:59.

**Conclusions:** The dose equivalency of IV fentanyl to IV morphine has traditionally been described as 1:100. We have shown when used for analgosedation, the dose equivalency of fentanyl to morphine is approximately 1:55 and doesn’t vary significantly when adjusting for weight, BMI, BSA, IBW and LBW.

**Reference**

1. Casamento AJ et al. Am J Respir Crit Care Med 204:1286–1294, 2021.

**Table 1 (abstract P194):** Results

	Fentanyl (µg)	Morphine (mg)	Dose ratio Fentanyl:Morphine
Hourly Dose*/ Total Cumulative dose	57.9 (40.0–88.0)/1930.0 (622.5–4520.0)	3.4 (2.2–4.9)/ 92.0 (41.5–275.0)	1:59/1:48
Dose (Total/hourly*)			
Per weight (kg)$	23.6 (8.5–53.9)/ 0.7 (0.4–1.0)	1.3 (0.5–3.5)/ 0.04 (0.02–0.06)	1:55/1:55
Per BMI (kg/m2)#	70.5 (28.7–173.3)/ 1.9 (1.3–3.1)	4.0 (1.7–12.6)/ 0.11 (0.07–0.18)	1:56/1:55
Per BSA (m2)#	1156.9 (403.2–2622.3)/ 29.1 (19.3–46.6)	61.2 (24.3–166.9)/ 1.7 (1.0–2.5)	1:53/1:57
Per IBW (kg)#	35.8 (11.8- 87.5)/ 0.9 (0.6–1.4)	2.0 (0.7–4.9)/ 0.05 (0.03–0.08)	1:55/1:58
Per LBW (kg)#	39.3 (14.3–95.3)/1.1 (0.7–1.6)	2.3 (0.9–5.7)/ 0.06 (0.03–0.09)	1:59/1:57

All data presented as median (IQR); *: hourly dose calculated by total amount divided by number of hours drug administered; $: n = 317 (fentanyl) and 309 (morphine); # n = 261 (fentanyl) and 273 (morphine)

## P195

### Dynamics of blood cytokines in neurosurgical patients under conditions of sedation-analgesia with opioids and alpha-2-adrenoagonists

#### NL Lesteva, A Kondratyev, ND Dryagina

##### ^1^Almazov National Medical Research Centre, Anesthesiology and Intensive Care, Saint-Petersburg, Russian Federation

*Critical Care* 2022, **26(Suppl 1):** P195

**Introduction:** The clinical significance of the inflammatory response in patients with brain tumors operated under general anesthesia is of interest. Postoperative delirium and postoperative cognitive impairment are associated with the intensity of the systemic inflammatory response and neuroinflammation.

**Methods:** 217 patients (74 (34.10%)—men and 143—women) operated for brain tumors. Anesthesia: propofol to a 3.5–6 mg/kg-hr, fentanyl 1—the 2.8 mcg/kg-h, clonidine 0.4–0.7 mcg/kg-h or dexmedetomidine 0.2–0.5 mcg/kg-h. We analysed cytokine levels (IL-8, IL-6, IL-10, TNF) at the four times: before induction, after induction, after elimination of the tumor (hemostasis) and the first day after surgery.

**Results:** On the first postoperative day, IL-6 values were on average eight times higher than the average IL-6 values during other stages of the study (Table 1). For IL-8 and IL-10, the highest average value is achieved at the stage of hemostasis, while the value of IL-8 is on average one and a half times higher, and the value of IL-10 is four times higher.

**Conclusions:** When analyzing the concentrations of pro-inflammatory and anti-inflammatory interleukins, depending on the stage of the perioperative period, the balance of their changes is shown. Pharmacological effect on the opioid and alpha-2-adrenergic systems of the brain provides modulation of the response to stress.

**Table 1 (abstract P195):** Cytokine levels during neurosurgery

	I	II	III	IV
IL-8, pg/ml	9,641 ± 2,038	9,357 ± 1,541	15,659 ± 4,113	10,968 ± 2,019
IL-6, pg/ml	3,114 ± 0,842	2,877 ± 0,692	6,550 ± 0,784	30,890 ± 4,557
IL-10, pg/ml	4,118 ± 0,751	4,618 ± 0,890	16,158 ± 4,126	5,142 ± 0,732
TNF, pg/ml	8,391 ± 0,450	7,915 ± 0,387	8,316 ± 0,435	7,005 ± 0,347

## P196

### ICU-free and ventilator-free days with isoflurane or propofol as a primary sedative: a *post-hoc* analysis of the Sedaconda study

#### H Bracht^1^, A Meiser^2^, J Wallenborn^3^, R Knafelj^4^, P Sackey^5^, J Nilsson^6^, M Bellgardt^7^

##### ^1^University Hospital Ulm, Dept. Emergency Medicine, Ulm, Germany, ^2^University Hospital Homburg/Saar, Department of Anesthesiology, Homburg, Germany, ^3^HELIOS Klinikum Aue, Department of Anesthesiology, AUE, Germany, ^4^University Medical Center Ljubljana, Klinični oddelek za interno intenzivno medicine, KOIIM, Ljubljana, Slovenia, ^5^Sedana Medical, Dept of Physiology and Pharmacology, Karolinska institutet, Stockholm, Danderyd, Sweden, ^6^Sedana Medical, Sedana Medical, Danderyd, Sweden, ^7^Katholisches Klinikum Bochum, Department of Anesthesiology, Bochum, Germany

*Critical Care* 2022, **26(Suppl 1):** P196

**Introduction:** The Sedaconda study was an RCT in invasively ventilated patients, comparing inhaled isoflurane (Iso) via the Sedaconda Anaesthetic Conserving Device (ACD) to iv propofol (iP) [1]. 150 patients received Iso and 151 iP, for up to 48 ± 6 h or extubation, whichever was first. Continued sedation, if needed, was at the physician’s discretion. We compared ICU-free days (ICU-FD) and ventilator-free days (VFD) in patients receiving the initial drug and never converting to the other drug in the 30 days from randomisation.

**Methods:** 69 Iso patients not switching to iP and 109 iP patients not switching to Iso after the study period were analysed. Study groups had similar demographic and clinical baseline characteristics (Table 1).

**Results:** The Iso group had significantly more ICU-FD than the iP group (15.9 vs 11.6, *p* = 0.008). VFD in Iso and iP were 18.6 and 16.6 respectively (*p* = 0.231). Additional analyses:

1. Comparing only patients that were further sedated after the study period (43 Iso patients vs 81 iP patients); the Iso group had more ICU-FD (14.7 vs. 9.6, *p* = 0.017) than the iP group. VFD for Iso vs iP were 18.7 and 14.8 respectively (*p* = 0.103).
2. Controlling for SAPS II at baseline; the Iso group had more ICU-FD than the iP group (15.6 vs 12.4 ICU-free days, *p* = 0.043). VFD for Iso vs iP were 18.5 vs 17.8 respectively (*p* = 0.672).
3. Controlling for SOFA score 48 h after randomisation; ICU-FD were more with Iso than with iP: 17.9 vs 13.3, *p* = 0.005. VFD for Iso vs iP were 20.4 and 17.6, respectively (*p* = 0.086).

**Conclusions:** Isoflurane, via Sedaconda ACD, as the primary sedative was associated with more ICU-free days than intravenous propofol. Ventilator-free days favoured isoflurane but differences did not reach statistical significance.

**Reference**

1. Meiser A et al. Lancet Respir Med 9:1231–1240, 2021

**Table 1 (abstract P196):** Demographic and clinical baseline characteristics

	Isoflurane (n = 69)	Propofol (n = 109)	*p* Value
Age, mean (SD) years	66 (11.8)	66 (13.15)	0.885
Female sex n (%)	17 (24.6)	38 (34.9)	0.150
BMI, Mean (SD) kg/m2	27.5 (6.1)	27.7 (7.2)	0.958
Emergency admission n (%)	39 (56.5)	71 (65.1)	0.299
Infection at admission, n (%)	33 (47.8)	57 (52.3)	0.480
SAPS II score, mean (SD)	40.1 (17.3)	44.3 (18.3)	0.191
SOFA score Mean (SD) 48 hours after randomisation	7.5 (4.3)	8.1 (3.7)	0.378

## P197

### IgM/IgA-enriched immunoglobulins: more than an IVIg: about multimeric IgA, IgM & J-chain

#### FB Bohländer^1^, SW Weißmüller^2^, JS Schüttrumpf^3^, STF Faust^1^

##### ^1^Biotest AG, Analytical Development and Validation, Dreieich, Germany, ^2^Biotest AG, Translational Research, Dreieich, Germany, ^3^Biotest AG, Corporate R&D, Dreieich, Germany

*Critical Care* 2022, **26(Suppl 1):** P197

**Introduction:** Severe infections are still a major health burden. Beneficial effects of treatment with IgM/IgA-enriched immunoglobulins compared to standard IVIg were reported and attributed to the additional IgM and IgA component [1]. Nevertheless, the molecular structure of such complex preparations is not fully understood. Especially in focus of respiratory diseases are multimeric IgM and IgA species that could be transported to mucosa [2]. The aim of our work was to characterize an IgM/IgA-enriched immunoglobulin with focus on IgA, IgM and the presence of J-chain which enables immunoglobulin transport to mucosa.

**Methods:** We characterized the IgM/IgA-enriched immunoglobulin Pentaglobin® (76% IgG, 12% IgA, 12% IgM) by measuring the ratio of IgG and IgA subclasses. Relative distribution of monomeric and multimeric immunoglobulins was analyzed by size exclusion chromatography. Specific detection of IgG, IgA, IgM and J-chain was performed by western-blot. J-chain was quantified by ELISA.

**Results:** The immunoglobulin distribution reveal the presence of several immunoglobulin isotypes (IgG/IgA/IgM) as well as IgG and IgA subclasses. Molecular size distribution reveal multiple multimeric species. Subsequent western-blot analysis shows pentameric IgM and dimeric IgA with J-chain, as well as monomeric IgG and IgA (Fig. 1). J-chain ELISA quantifies a notable portion of IgA and IgM molecules that is associated with J-chain.

**Conclusions:** The results of this study show the biochemical complexity of polyvalent IgM/IgA-enriched immunoglobulins. The data highlight a substantial portion of multimeric IgM and IgA species. The presence of J-chain indicates the potential secretion of IgA and IgM onto mucosa. These observed biochemical properties could explain the beneficial effects of IgM/IgA-enriched immunoglobulins compared to standard IVIg in therapy of severe infections.

**References**

1. Kakoullis L et al. J Crit Care 47:30–35, 2018.
2. Sterlin D et al. Pharmacology 106:9–19, 2021.

**Fig. 1 (abstract P197):**
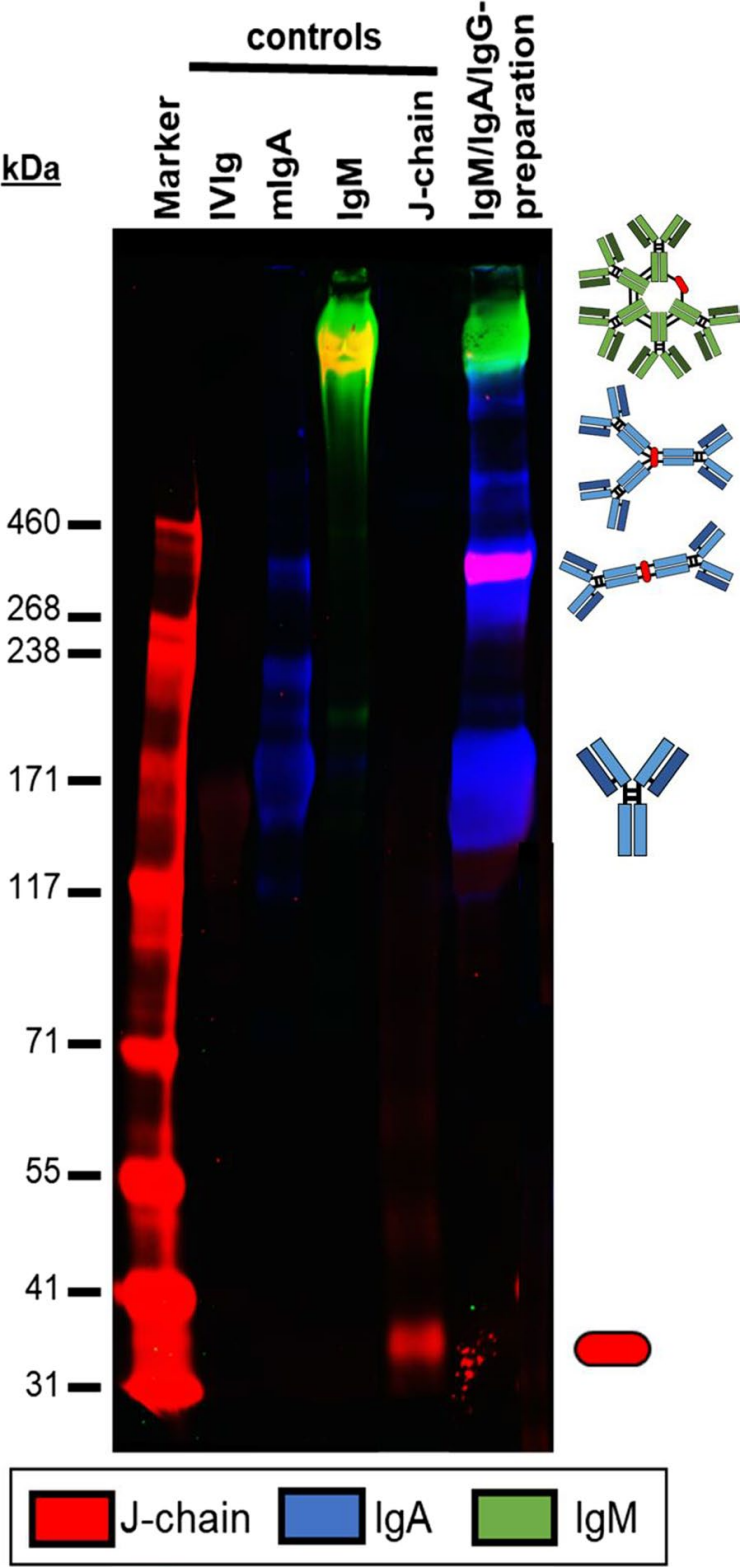
Non-reducing Western-Blot analysis of IgM/IgA-enriched immunoglobulin preparation. Indicated samples were separated by SDS-PAGE and transferred to nitrocellulose membrane. Specific detection antibodies and fluorophore labeled secondary antibodies were used for detection of J-chain (red), IgA (blue) and IgM (green). Presumed structure of different immunoglobulin species is indicated. Abbreviations: Marker—HiMark™ Protein-Standard; IVIg—Intravenous Immunoglobulin (Intratect®); mIgA—monomeric IgA; IgM/IgA/IgG-preparation—IgM/IgA enriched immunoglobulin (Pentaglobin®).

## P198

### Immunoglobulins combined with antibiotics improve outcome of patients with severe pneumococcal pneumonia. A retrospective observational study

#### M Benlabed^1^, S Benlabed^2^, R Gaudy^3^, S Nedjari^4^, A Ladjouze^5^, S Aissaoui^5^

##### ^1^Lille University, Anesthesiology, Lille, France, ^2^Free University of Brussels, Internal Medicine, Brussels, Belgium, ^3^Lille University, Internal Medicine, Lille, France, ^4^Algiers University, Algiers, Algeria, ^5^Algiers University, Anesthesiology, Algiers, Algeria

*Critical Care* 2022, **26(Suppl 1):** P198

**Introduction:** The role of intravenous immunoglobulin (IVIG) as an adjunctive treatment for severe sepsis remains controversial [1]. We hypothesized that IVIG associated with antibiotics could be more effective than antibiotics alone for treating patients with severe pneumococcal pneumonia.

**Methods:** We performed a retrospective study and analyzed the data of 2 groups of 20 patients presenting severe pneumoccocal pneumonia and admitted in an university ICU between 2015 and 2019. All The patients were 60 + -10 years old and mechanically ventilated. On admission, a first group receiving IVIG and antibiotics (study group) and a second group receving placebo(saline) and antibiotics (control group). IVIG were administered on the first hour of admission at a dose of 0.5 g/kg during 3 days and infused continuously at 3 ml/kg/h. IVIG were preceded by administration of ceftriaxon-levofloxacin. We recorded in the 2 groups: CRP, SOFA score Day 1 and Day 4, Time on mechanical ventilation(MV), PaO_2_/FiO_2_ ratio on day 1 and day 3, the incidence of septic shock, ICU stay, the hospital length of stay (LOS) and 28-day mortality.

**Results:** Statistical analysis used Mann–Whitney test and results expressed as mean with standard deviation (Table 1). We observed that SOFA score Day 3, time on MV, ICU stay and LOS were significantly reduced in study group compared to control group. PaO_2_/FiO_2_ Day 3 was more elevated in study group than in control group, respectively 220 + -5 vs 180 + -6 *p* < 0.001. Septic shock was present in 10 patients sur 20 in control group and in 6 patients sur 20 in study group. Day 28 mortality was significantly decreased in study group compared to control group respectively 30% vs 50%.

**Conclusions:** Early administration of high doses IVIG combined with antibiotics improved outcome of patients with severe pneumoccocal pneumonia. A randomized controlled study, including a large population of patients is needed to confirm these interesting results.

**Reference**

1. De Hennezel L et al. Antimicrob Agents Chemother 45:316–8, 2001

**Table 1 (abstract P198):** Variables related to outcome in study and control groups

	Study group	Control group	*p*
SOFA Score Day 4	4.53±0.71	7.22±072	< 0.0001
ICU STAY (days)	11.93±0.45	16.86±0.55	< 0.0001
LOS (days)	23.06±0.88	29.4±1.12	< 0.004
Septic shock %	30%	50%	< 0.001
Time on MV (days)	6.76±0.41	9.86±0.26	< 0.0007
CRP day 2 (mg/l)	14±2	47±5	< 0.0001
PaO_2_/FiO_2_ ratio Day 3	220±5	180±6	< 0.001

## P199

### Neutralization of circulation histone with sodium-β-O-methyl cellobioside sulphate in sepsis (a prospective randomized double-blinded placebo-controlled preclinical trial)

#### BG Garcia^1^, YW Wang^2^, N Li^2^, A Khaldi^1^, A Moiroux^1^, FS Taccone^3^, JL Vincent^3^, J Creteur^3^, L Shi^2^, F Su^3^

##### ^1^Erasme Hospital, Brussels, Belgium, ^2^ Grandpharma Ltd, Wuhan, China, ^3^Erasme Hospital, Department of Intensive Care, Université libre de Bruxelles, Brussels, Belgium

*Critical Care* 2022, **26(Suppl 1):** P199

**Introduction:** High circulating levels of histones were correlated to sepsis severity and worse outcome. Our hypothesis was that the neutralization of histones with sodium-β-O-methyl cellobioside sulphate (STC3141, from Grandpharma, China) in sepsis might improve sepsis outcome.

**Methods:** Sepsis was induced by fecal peritonitis in twenty-four mechanically ventilated, hemodynamically monitored female sheep. Animals were randomized to three groups control, concurrent treatment (CuT) and post treatment (PT) group (N = 8 each) after surgical preparation and stabilization. STC3141 was given as bolus (1 mg/kg) + continuous (1 ml/kg/h). It was started at sepsis inducement in the CuT group and 4 h after in the PT group. During the first 4 h, fluid was maintained at 2 ml/kg/h. 4 h after sepsis, fluid resuscitation (maintain pulse pressure variation < 13%), antibiotics and peritoneal lavage were administrated, and norepinephrine was given to maintain mean arterial blood pressure > 65 mmHg if necessary. Experiment lasted for 24 h.

**Results:** During the first 4 h, MAP was maintained in the CuT group, while dropped significantly in the PT and Control group. Significantly lower dose of NE and lactate levels was observed in the two treatment groups compared to the control group. Impaired sublingual microcirculation significantly improved at 6 h in the two treatment groups at 18 h in the PT group. Survival benefit tended to be longer in the two treatment groups (*p* = 0.075).

**Conclusions:** Neutralization of histones with STC3141 in sepsis quickly stabilized hemodynamics with less NE utilization, ameliorated impaired microcirculation and improved tissue perfusion, which might provide a new therapeutic approach for sepsis.

## P200

### A novel virotherapy encoding human interleukin 7 enhances *ex vivo* lymphocyte functions in immunosuppressed septic shock and critically ill COVID-19 patients

#### M Crausaz^1^, G Monneret^2^, P Martin^1^, F Conti^2^, A Lukaszewicz^3^, G Inchauspé^1^, F Venet^4^

##### ^1^Transgene, Department of Infectious Diseases, Lyon, France, ^2^EA 7426 Pathophysiology of injury-induced immunosuppression (PI3), Lyon 1 University/ Hospices Civils de Lyon / bioMérieux, Hôpital Edouard Herriot, Lyon, France, ^3^Hospices Civils de Lyon, Hôpital Edouard Herriot, Service d’anesthésie-réanimation, Lyon, France, ^4^Hospices Civils de Lyon, Hôpital Edouard Herriot, Laboratoire d’Immunologie, Lyon, France

*Critical Care* 2022, **26(Suppl 1):** P200

**Introduction:** After viral or bacterial sepsis, most intensive care unit (ICU) patients enter a state of profound immunosuppression contributing to patients’ worsening. Transgene has developed an immunotherapy based on a viral vector encoding human interleukin-7 (hIL-7) to restore both innate and adaptive immune responses. Here, we assessed the capacity of hIL-7 to improve *ex* *vivo* T lymphocyte function from septic shock and COVID-19 patients.

**Methods:** Primary human hepatocytes were transduced with MVA-hIL-7-Fc, a recombinant Modified Vaccinia virus Ankara (MVA) encoding the hIL-7 fused to the human IgG2 Fc fragment, or with empty MVA as control. Cell culture supernatants were harvested for further assays. T cells were collected from ICU patients (septic shock = 11, COVID-19 = 29) and healthy donors (n = 21). STAT5 phosphorylation, cytokine production (ELISpot and intracellular staining) and cell proliferation were assessed upon TCR stimulation with supernatants containing or not hIL-7 produced after MVA transduction or with the counterpart recombinant hIL-7 (rhIL-7).

**Results:** Patients with viral and bacterial sepsis display T lymphocyte alterations compared to healthy donors with a decreased production of cytokines and a decreased proliferation capacity. Supernatant containing hIL-7 induces STAT5 phosphorylation in CD3 lymphocytes of all patients. With 90% of responders, hIL-7 boosts cytokines production (single and double IFN-TNF) and T lymphocytes proliferation capacity at the same level as rhIL-7 in both cohorts whereas empty MVA has no effect.

**Conclusions:** This study indicates that hIL-7-Fc produced after MVA transduction initiates IL-7 signaling through the phosphorylation of STAT5 and restores *ex* *vivo* human lymphocyte functions in cells from septic patients with acquired immunosuppression. This proof-of-concept study, along with experimental results in animal models, supports the clinical development of the MVA-hIL-7-Fc in sepsis immunosuppressed patients.

## P201

### Extracorporeal immune cell therapy of sepsis—*ex vivo* one-way results

#### G Klinkmann^1^, T Wild^2^, B Heskamp^2^, F Doss^2^, S Doss^2^, M Milej^2^, DA Reuter^1^, S Mitzner^3^, J Altrichter^2^

##### ^1^University Medical Center Rostock, Department of Anaesthesiology and Intensive Care Medicine, Rostock, Germany, ^2^Artcline GmbH, Artcline GmbH, Rostock, Germany, ^3^Division of Nephrology, Medical Faculty, Department of Medicine, University of Rostock, Rostock, Germany

*Critical Care* 2022, **26(Suppl 1):** P201

**Introduction:** Immune cell dysfunction is a crucial part in sepsis. Granulocyte concentrate (GC) transfusions, as the only available immune cell concentrates, potentially induce tissue damage via local effects of neutrophils. Therefore, using the donor immune cells purely extracorporeally is an attractive option. Clinical trials with standard GC in an extracorporeal plasma treatment achieved beneficial effects. In this *ex* *vivo* study, purified GC with longer storability were investigated in a simplified extracorporeal plasma treatment system.

**Methods:** Purified GC (pGC) were stored up to 3 days and used in a plasma perfusion therapy model simulating a 6 h treatment. The extracorporeal circuit consists of a blood circuit and a plasma circuit with 3 plasma filters (PF) (Fig. 1). PF1 is separating the plasma from the patient’s blood, plasma is perfused through PF2 containing the donor immune cells and only the treated plasma is re-transfused. A PF3 is included in the plasma backflow as a redundant safety measure. 1000 ml donor plasma was used to simulate patients. Granulocyte efficacy information on phagocytosis, oxidative burst and cell viability as well as cytokine release and metabolic parameters were assessed.

**Results:** Cells were viable throughout the study period and exhibited well-preserved functionality and efficient metabolic activity. No indication of immune cell impairment was detected. Also, cytokines are actively secreted during the extracorporeal treatment simulation. Of particular interest is equivalence in performance of the granulocytes on day 1 and day 3, demonstrating sustained shelf life of pGC.

**Conclusions:** Results demonstrate that cells are highly active in removing toxic or inflammatory compounds from plasma and secreting cytokines into plasma. Furthermore, granulocytes remain viable and active even after storage for 3 days supporting the use of the system in clinical trials.

**Fig. 1 (abstract P201):**
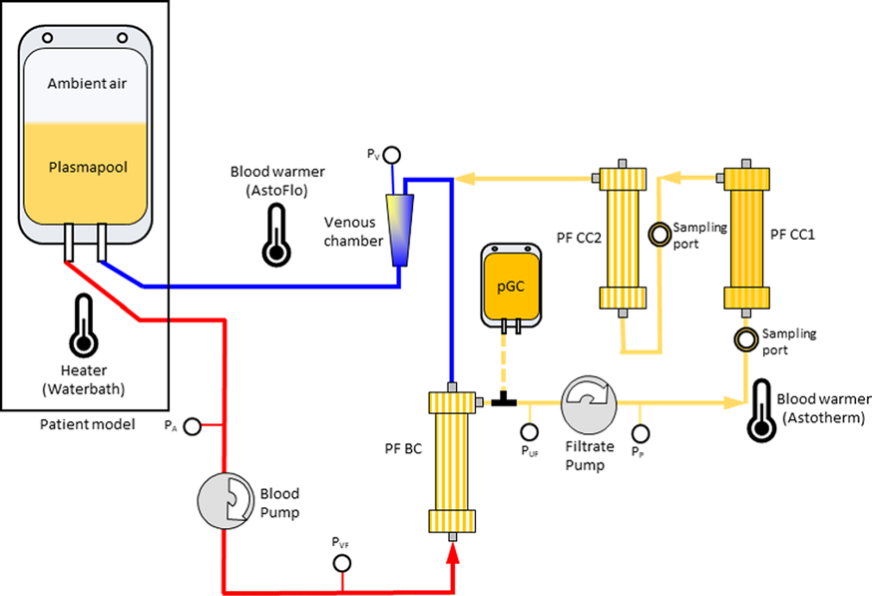
Schematic of the treatment simulation experiments of the extracorporeal immune cell therapy. Plasma is continuously filtered from the patient’s extracorporeal blood circuit and transferred into a closed-loop ‘cell circuit’ (CC), where the plasma is brought into direct contact with therapeutically effective, human-donor immune cells (i.e. the purified granulocyte concentrate pGC).

## P202

### Recovery from acute immune failure in septic shock by immune cell extracorporeal therapy: REACTIF-ICE

#### G Klinkmann^1^, T Wild^2^, B Heskamp^2^, F Doss^2^, S Doss^2^, M Milej^2^, DA Reuter^1^, J Altrichter^2^, S Mitzner^3^

##### ^1^University Medical Center Rostock, Department of Anaesthesiology and Intensive Care Medicine, Rostock, Germany, ^2^Artcline GmbH, Artcline GmbH, Rostock, Germany, ^3^Division of Nephrology, Medical Faculty, Department of Medicine, University of Rostock, Rostock, Germany

*Critical Care* 2022, **26(Suppl 1):** P202

**Introduction:** Immune cell dysfunction is a crucial part in sepsis and in particular in septic shock. Granulocyte concentrate (GC) transfusions, as the only available immune cell concentrates, potentially induce tissue damage via local effects of neutrophils. Therefore, using donor immune cells purely extracorporeally is an attractive option. Clinical trials with standard GC in an extracorporeal plasma treatment achieved beneficial effects. In this clinical trial, purified GC with longer storability will be investigated in a streamlined extracorporeal plasma treatment.

**Methods:** We describe a prospective, phase II, multicenter, randomized controlled parallel-group clinical trial in patients with refractory septic shock. Subjects suffering from septic shock according to Sepsis-3-Definition who additionally require norepinephrine at a dose of ≥ 0.2 mcg/kg/min (and/or vasopressin at any dose) for a minimum of 6 h (within the last 48 h will be randomized. Patients are randomized to receive either standard of care therapy or extracorporeal immune cell therapy on top of standard of care, in a 1:1 ratio. A total of 120 evaluable patients will be enrolled at 4 sites within Germany. Primary endpoint is safety and tolerability consisting of overall mortality and new onset of serious adverse events. A key secondary endpoint is showing recovery from immune dysfunction after extracorporeal immune cell treatment.

**Results:** This study has been submitted to independent ethics committees and responsible agencies in Q4/2021 and is expected to start in Q1/2022 (Fig. 1).

**Conclusions:** The extracorporeal immune cell plasma perfusion therapy may on one hand provide immune support and may avoid unwanted local side effects on the other hand. Recovery from immune dysfunction is a prerequisite for avoiding secondary infections and ultimately also for recovery from sepsis.

**Fig. 1:**
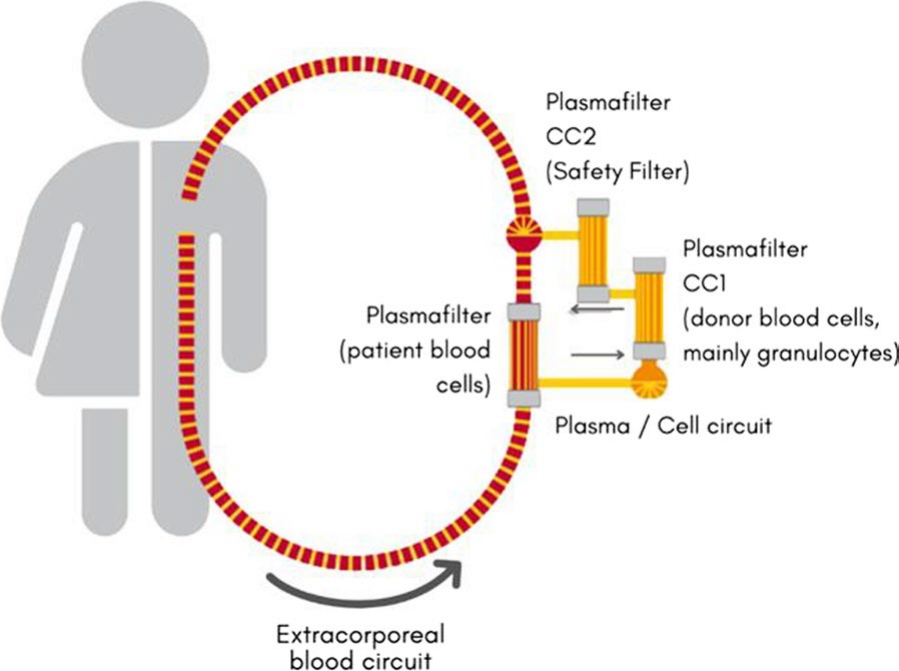
Scheme of the extracorporeal immune cell plasma perfusion therapy. Plasma is continuously filtered from the patient’s extracorporeal blood circuit and transferred into a closed-loop ‘cell circuit’, where the patient’s plasma is brought into direct contact with therapeutically effective, human-donor immune cells (i.e. granulocyte concentrate).

